# Asymmetric synthesis of multiple quaternary stereocentre-containing cyclopentyls by oxazolidinone-promoted Nazarov cyclizations†
†Electronic supplementary information (ESI) available: Experimental details and NMR spectra of all newly synthesized compounds. X-ray crystal data for (3*S*)-**23** and *E*-**24**. Details and further discussion of computational studies. CCDC 1814626 and 1814627. For ESI and crystallographic data in CIF or other electronic format see DOI: 10.1039/c8sc00031j


**DOI:** 10.1039/c8sc00031j

**Published:** 2018-04-20

**Authors:** Rohan Volpe, Romain J. Lepage, Jonathan M. White, Elizabeth H. Krenske, Bernard L. Flynn

**Affiliations:** a Monash Institute of Pharmaceutical Sciences , Monash University , 381 Royal Parade , Parkville , Victoria 3052 , Australia . Email: bernard.flynn@monash.edu; b School of Chemistry and Molecular Biosciences , The University of Queensland , Brisbane , QLD 4072 , Australia; c Bio21 Institute , School of Chemistry , University of Melbourne , Parkville , VIC 3010 , Australia

## Abstract

Carbometalation of oxazolidinone (**Ox**)-substituted ynamides is used to generate highly substituted **Ox**-divinyl (and aryl vinyl) ketones for use in Nazarov cyclizations. The **Ox**-group serves as a remarkably effective chiral activating group, enabling the torquoselective Nazarov cyclization of these sterically congested substrates to be performed under mild conditions. It also serves as a charge-stabilizing group in the intermediate oxyallyl cation, suppressing undesired [1,2]-sigmatropic shifts of neighboring substituents and facilitating the regio- and stereoselective incorporation of nucleophiles to yield cyclopentanoids containing up to three contiguous all-carbon quaternary (4°) stereocentres.

## Abstract 2

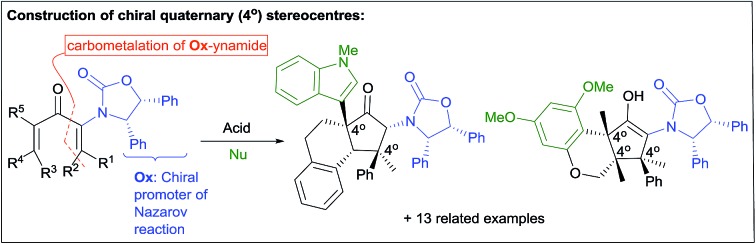
 **Ox**-activated divinyl ketones undergo torquoselective Nazarov cyclization to give cyclopentanoids containing up to three new contiguous quaternary (4°) stereocentres.

## Introduction

The enantioselective synthesis of quaternary (4°) stereocentres is a major challenge in organic synthesis, hindering access to sp^3^-rich scaffolds in drug discovery and natural products synthesis.^1^,^2^ Particularly problematic is the enantioselective formation of multiple 4°-stereocentres, which requires control over both relative and absolute stereochemistry.

**Scheme 1:**
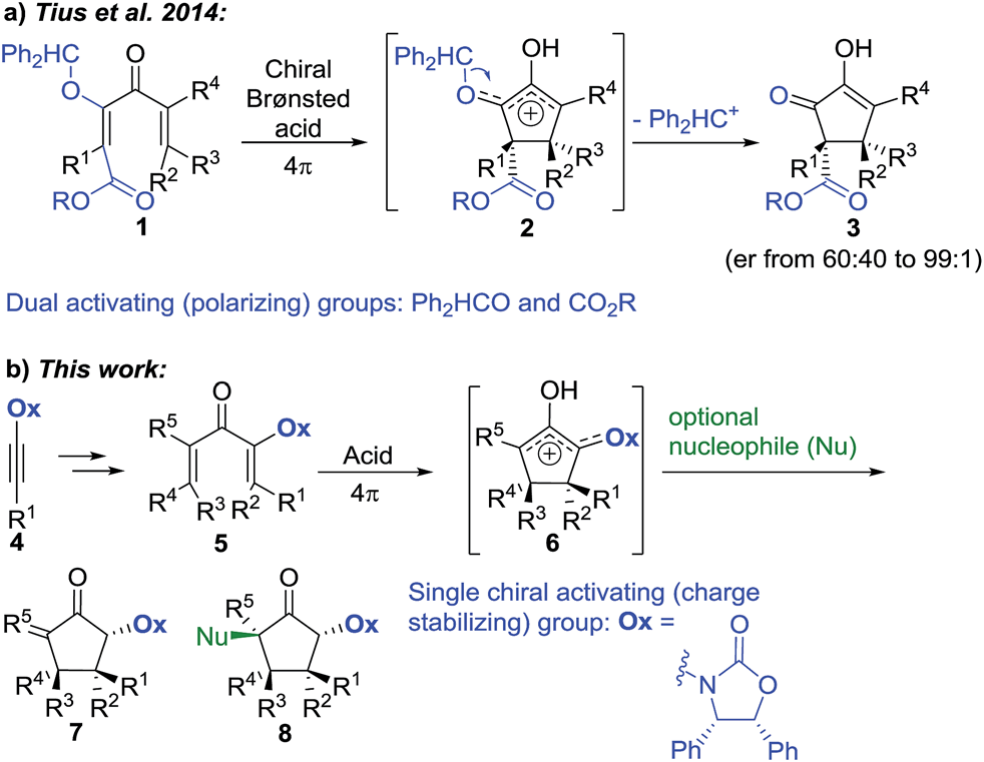
Nazarov substrate activation modes for the enantioselective synthesis of 4°-stereocentres.

The Nazarov cyclization offers inherent control over relative stereochemistry through conservation of orbital symmetry and constitutes an attractive route to multistereocentre-containing cyclopentanoids.^3^ However, the potential of the Nazarov cyclization for 4°-stereocentre formation has not yet been fully realized due to two significant challenges: (i) stereoselective access to highly substituted divinyl (and aryl vinyl) ketone substrates^4^ and (ii) torquoselective^5^ ring closure. In a landmark study, Tius and co-workers^6^ reported chiral Brønsted acid-catalyzed Nazarov cyclizations of divinyl ketones **1** (Scheme 1a) leading to cyclopentenols **3** containing two new vicinal 4°-stereocentres (R^1–3^ ≠ H) with high enantioselectivities (often er > 97 : 3). Careful design of the divinyl ketone **1** with dual-activating electron donor (OCHPh_2_) and acceptor (CO_2_R) elements was key to attaining efficient cyclization.^6^ Electrofugal release of Ph_2_HC^+^ from the intermediate oxyallyl cation **2** further promoted the cyclization and suppressed competing Wagner–Meerwein rearrangements ([1,2]-sigmatropic shifts of R^1–3^ within **2**). Herein, we report that highly substituted aryl vinyl and divinyl ketones **5** can be readily accessed through carbometalations of oxazolidinone (**Ox**)-substituted ynamides **4** (Scheme 1b).^7^ The **Ox**-group proves to be remarkably effective as a single chiral activating group for the Nazarov cyclizations of these highly substituted and sterically congested substrates **5**, giving *exo*-methylene cyclopentanones **7** under remarkably mild conditions, with excellent and predictable enantiocontrol. Furthermore, since no electrofugal release is required for substrate activation or suppression of Wagner–Meerwein rearrangements, the oxyallyl cation **6** can be exploited in nucleophilic trapping^7^,^8^ to afford multistereocentre-centre-containing products **8** with up to three all-carbon 4°-stereocentres. The rapid assembly of such levels of complexity from a prochiral starting material highlights the powerful activating and stereocontrolling influence of the **Ox** group. Using theoretical calculations, we show that the exceptional activating properties of **Ox** originate from a combination of covalent and non-covalent transition-state stabilizing effects.

## Results and discussion

Two different carbometalation strategies were developed to give access to **Ox**-containing divinyl and aryl vinyl ketones **5** (R^2^ = alkyl/aryl, Table 1). Firstly, Cu-catalyzed carbomagnesiation of **Ox**-ynamides **4** with Grignard reagents gave **9** (M = MgBr);^9^ alternatively, Rh-catalyzed carbozincation of **4** with ZnEt_2_ gave **9** (M = ZnEt).^10^ Addition of iodine to organometallics **9** (M = MgBr or ZnEt) gave the key building block alkenyliodides **10a** (68%) and **10b** (79%). Carbonylative Stille coupling (Method A) of **10a** and **10b** with tributyl(cyclohexen-1-yl)stannane afforded divinyl ketones **5a**/**a′** and **5b**/**b′**, respectively, each as a 5 : 1 mixture of *E/Z*-isomers about the **Ox**-substituted double bond (entries 1–4).^11^ Despite this partial isomerization, the major isomers, **5a** and **5b**, were isolated in 55% and 52% yield, respectively. All other divinyl and aryl vinyl ketones **5** shown in Table 1 were accessed by reaction of **9** (M = MgBr) with the corresponding aldehyde followed by Dess–Martin periodinane oxidation of the crude alcohols (Method B) giving **5c–j** in yields of 31–91% (entries 5–12).

**Table 1:** Synthesis of Nazarov substrates **5** and their cyclization to 4°-stereocentre-containing cyclopentanoids **7**

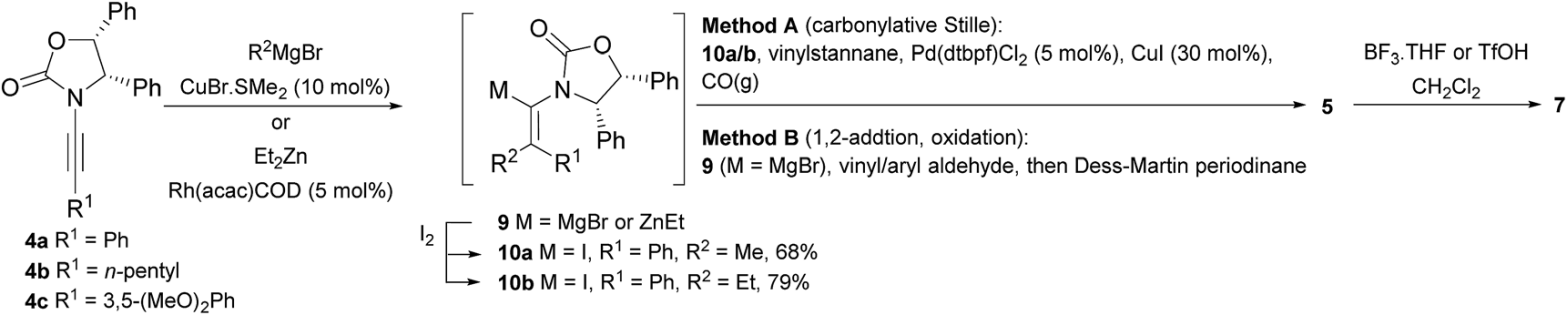								
	**4**/**10** → **5**^*a*^	**5** → **7**^*c*^ (dr)^*d*^		**4**/**10** → **5**^*a*^	**5** → **7**^*c*^ (dr)^*d*^		**4**/**10** → **5**^*a*^	**5** → **7**^*c*^ (dr)^*d*^
1	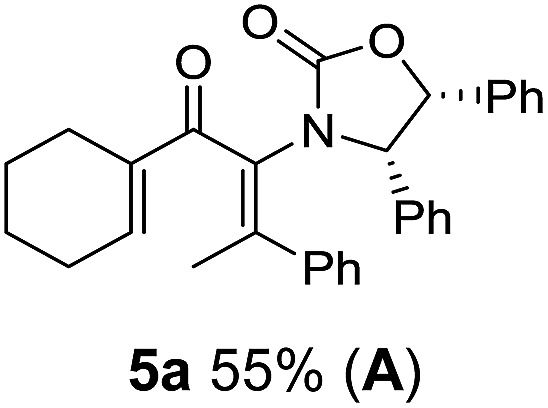	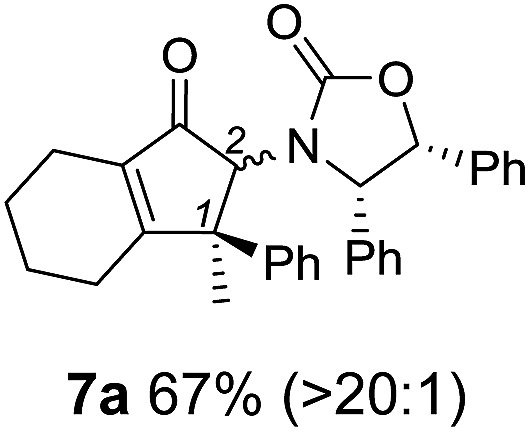	5	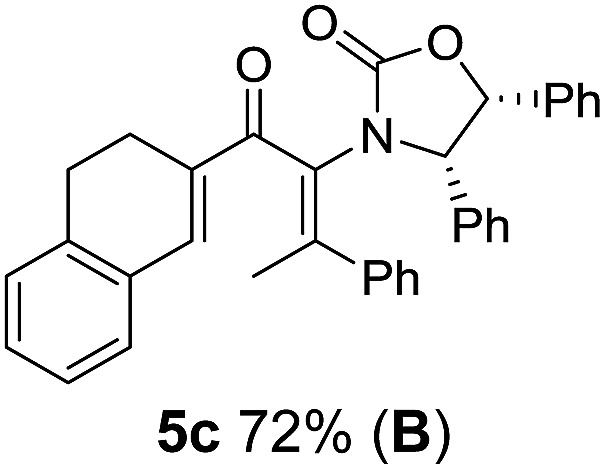	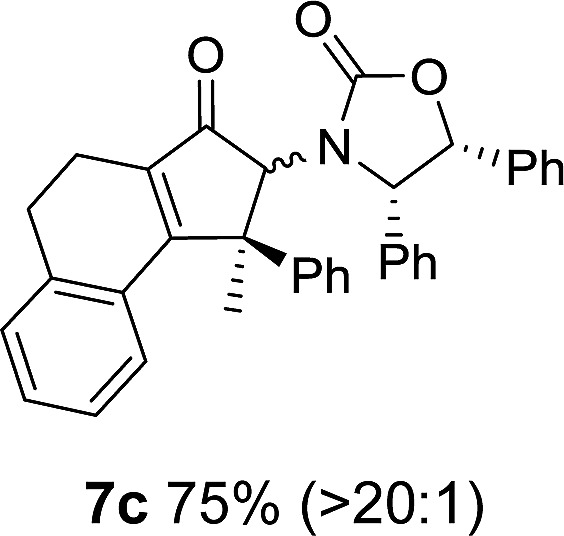	9	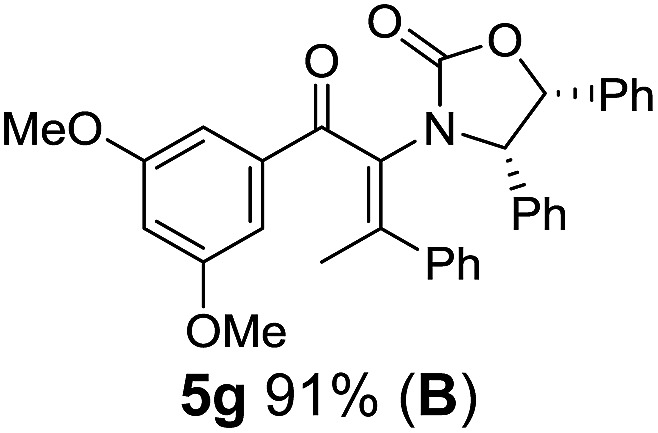	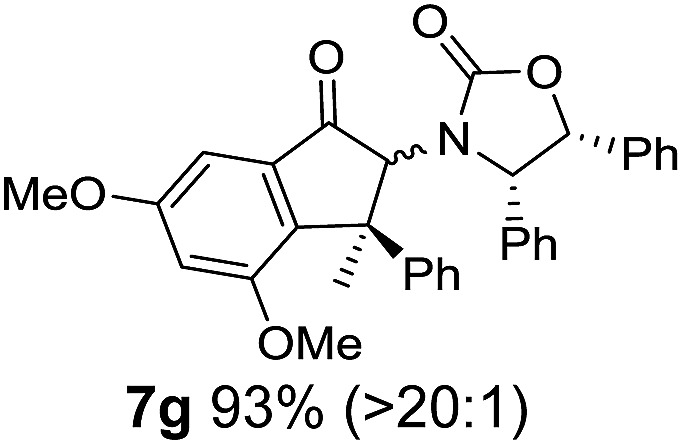
2	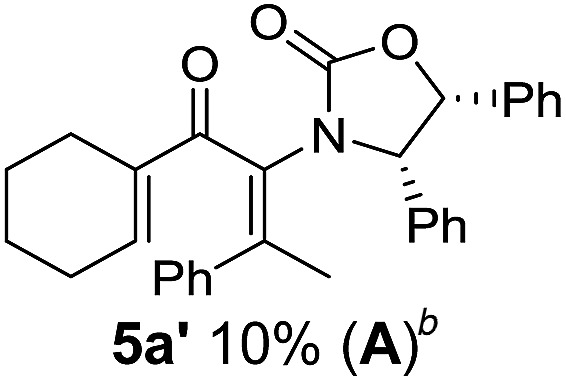	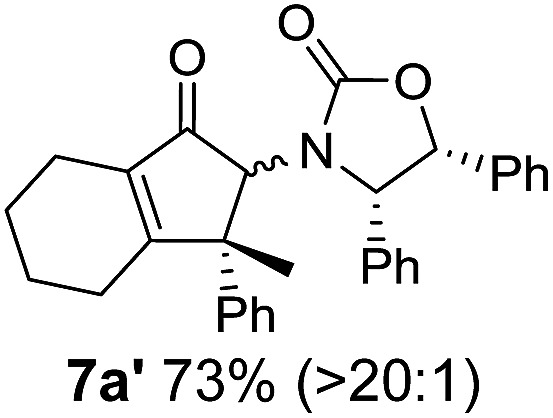	6	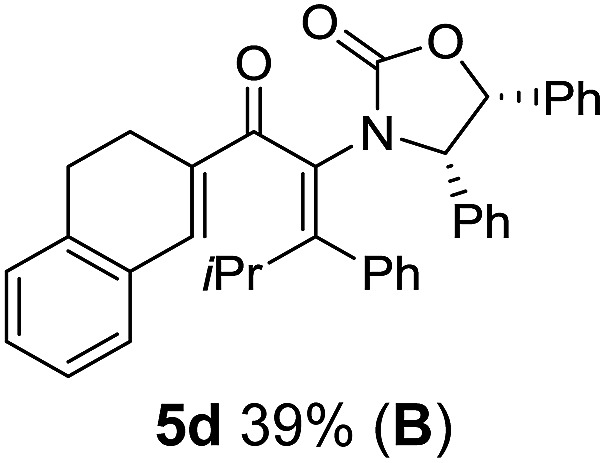	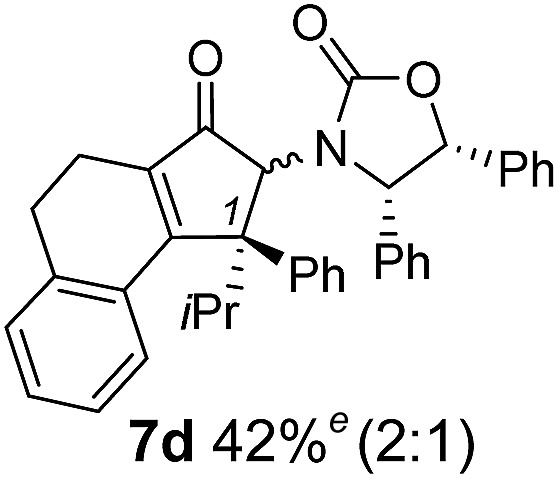	10	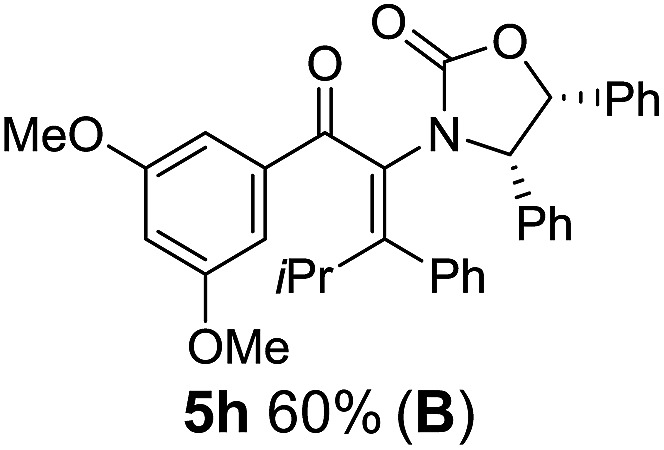	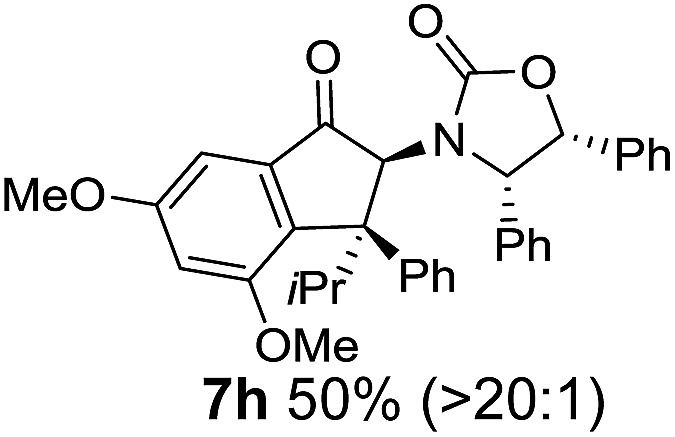
3	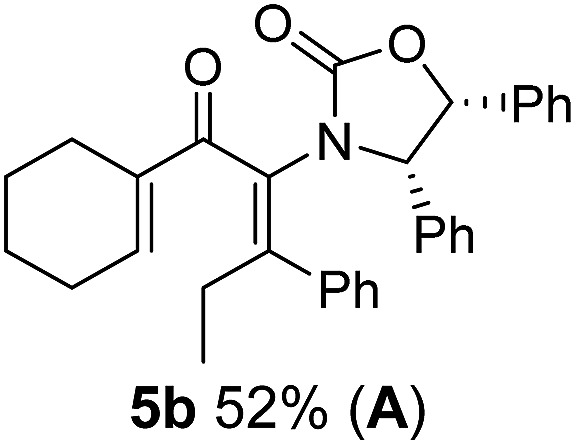	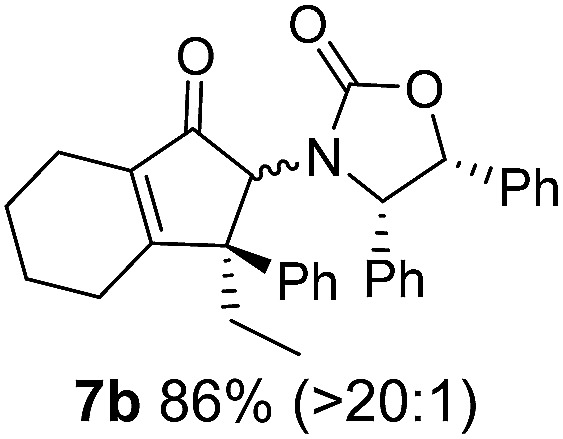	7	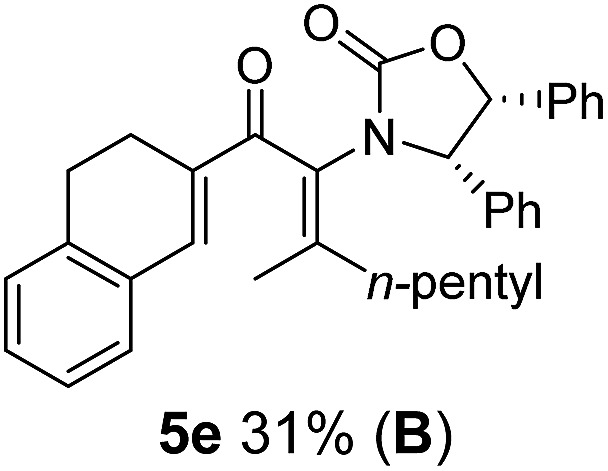	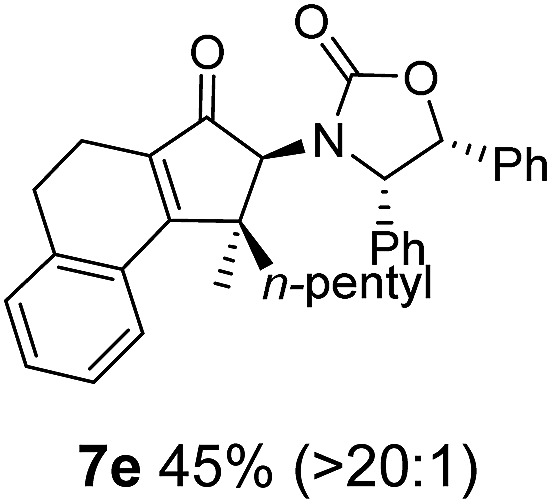	11	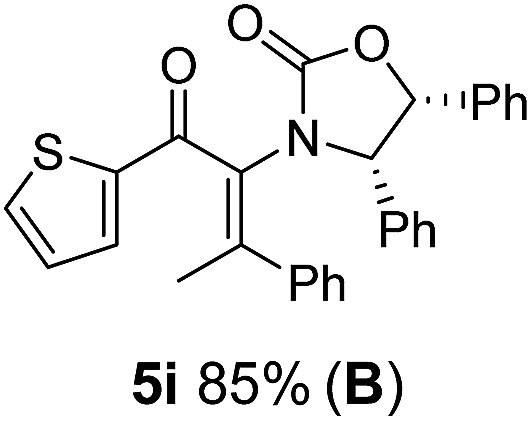	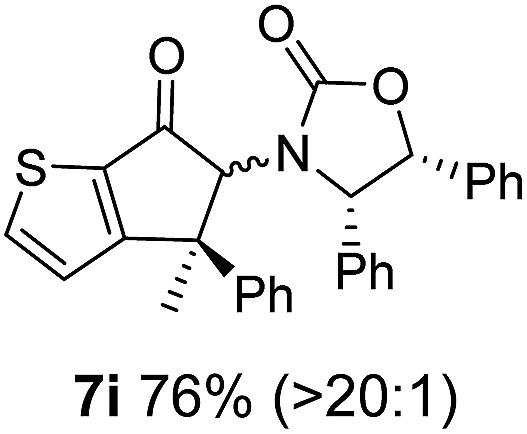
4	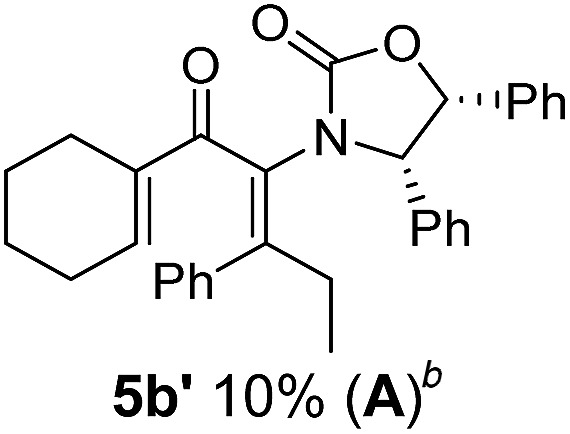	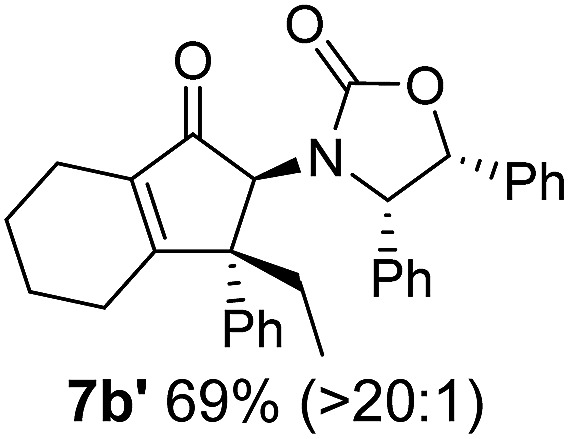	8	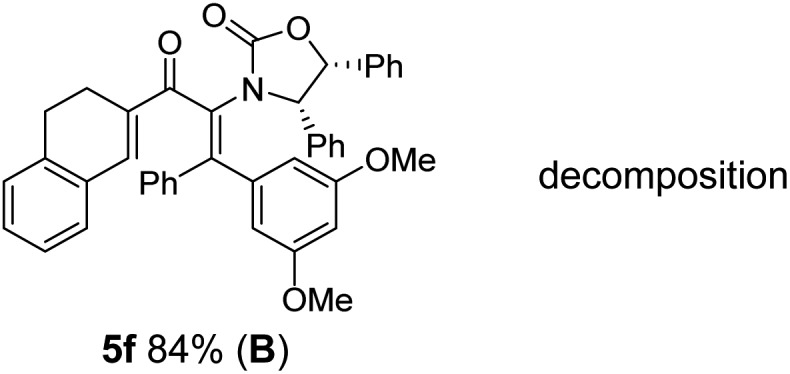		12	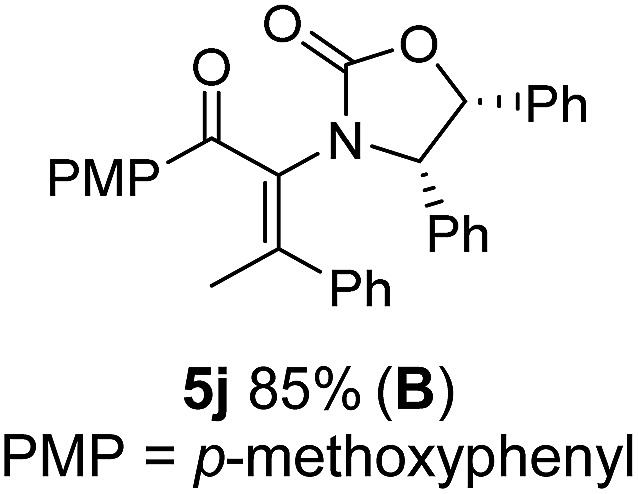	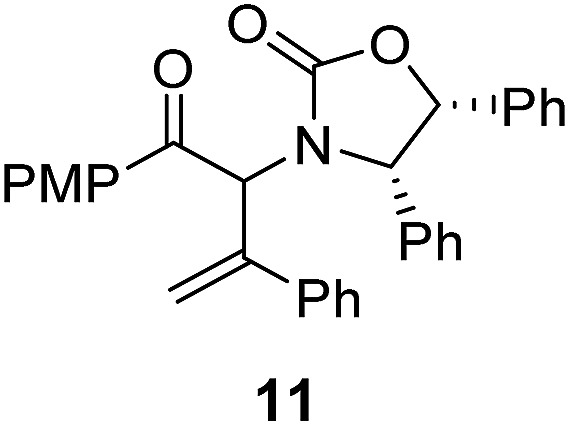

^*a*^Nazarov substrates **5** formed from **4** using Method A (**A**) or Method B (**B**), as indicated.

^*b*^Isolated as a minor isomer using Method A.

^*c*^Cyclized with BF_3_·THF or TfOH in CH_2_Cl_2_ at various temperatures (ranging from –78 °C to 40 °C) depending on acid and substrate; see text and ESI for details.

^*d*^Diastereomeric ratio (dr) refers to stereochemistry at C1 relative to **Ox** (determined by ^1^H NMR). Some products **7** were isolated as a mixture of C2-epimers, indicated by a wavy bond (see ESI for ratio), these give a single enantiomer upon **Ox** removal (eqn (1), ref. 7).

^*e*^Isolated yield of C1-(*S*) isomer, an additional 24% was isolated as a 3 : 1 (*R*) : (*S*)-C1 mix.

Nazarov cyclizations of divinyl and aryl vinyl ketones **5a–j** were performed using either BF_3_·THF or TfOH as catalyst in CH_2_Cl_2_, giving cyclopentanoids **7a–i** (**5f** and **5j** did not cyclize) containing one new 4°-stereocentre (Table 1). Broadly speaking, these Nazarov cyclizations performed very well, particularly where the “inner” substituent (R^2^) in **5** was Me, Et or Ph (BF_3_·THF or TfOH). Use of TfOH as catalyst allowed the Nazarov cyclization to be conducted at temperatures as low as –78 °C, but generally the reactions were performed at 0 °C to rt or in refluxing CH_2_Cl_2_ (40 °C) using either TfOH or BF_3_·THF.‡
‡See the ESI.†
 The torquoselectivities were very high (dr > 20 : 1 for C1 relative to **Ox**), with the sole exception of **7d** (dr = 2 : 1 (*S*) : (*R*)-C1, entry 6). X-ray crystal structure and density functional theory (DFT) studies have shown that **Ox** auxiliaries of this configuration consistently favor anticlockwise conrotation leading to R^1^-β stereochemistry (see below);7*b* we have therefore assigned this stereochemistry to each product in Table 1. Most likely, the cyclization of **5d**, which required heating to 40 °C due to the sterically encumbering isopropyl group (R^2^ = *i*Pr), gave lower selectivity due to partial *Z*/*E*-isomerization of the oxazolidinyl-alkene prior to cyclization, rather than because of poor stereoinduction by the auxiliary (see also below).

The presence of two aliphatic substituents on the tetrasubstituted alkene terminus, as in **5e**, led to slower cyclization, but the stereoinduction remained high (entry 7). Diaryl-substituted alkene **5f** underwent undesired side reactions to give multiple minor products along with return of starting material (entry 8). In a number of cases, the presence of epimers at C2 (the carbon bearing **Ox**) was apparent, but both epimers lead to the same product once the auxiliary is removed by reductive cleavage (see below). Cyclizations of electron-rich aryl vinyl ketones were successful (entries 9–11), even for the very hindered substrate **5h** where R^2^ = *i*Pr. For the less activated aryl vinyl ketone **5j**, alkene isomerization to form β,γ-unsaturated ketone **11** became the dominant pathway and no Nazarov cyclization was observed.

As has been demonstrated in our previous study utilizing a diverse array of less substituted Nazarov products **7** (R^2^ = R^3^ = H), the oxazolidinone can be removed by reductive-cleavage using lithium naphthalenide (LiNph).7*c* Two examples are given as part of this work (eqn (1)): reductive cleavage of the **Ox** group in **7c** and **7i** gave **12** (79%) and **13** (55%), respectively, both in high enantiomeric purity (er > 98 : 2).1
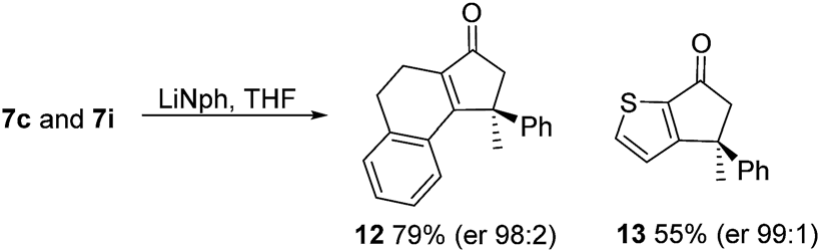



Also, as per our previous work, additional stereochemical complexity can be built up by nucleophilic trapping of the intermediate oxyallyl cations **6**.7*c* Accordingly, the highly substituted divinyl ketone **5c** was converted into the indole-trapped product **14** (75%) (eqn (2)). Notably, this tandem sequence generates four new contiguous stereocentres, including two 4°-centres, with excellent control over both relative and absolute stereochemistry: only a single isomer was observed.2
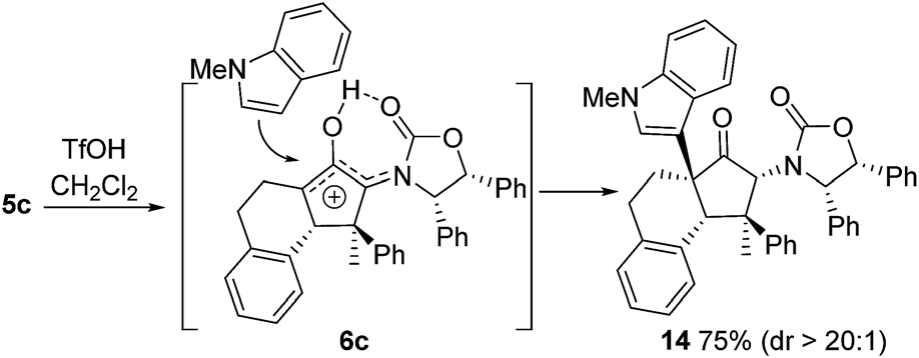



**Scheme 2:**
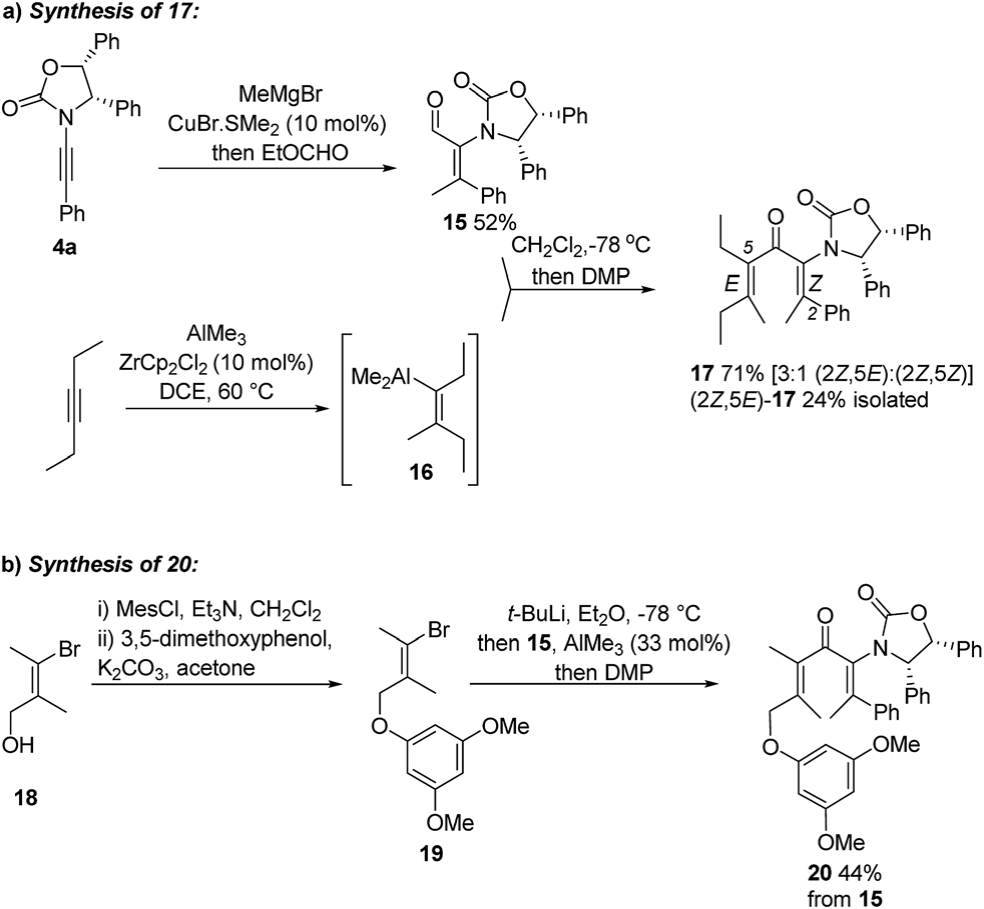
Syntheses of fully substituted **Ox**-divinyl ketones **17** and **20**.

**Scheme 3:**
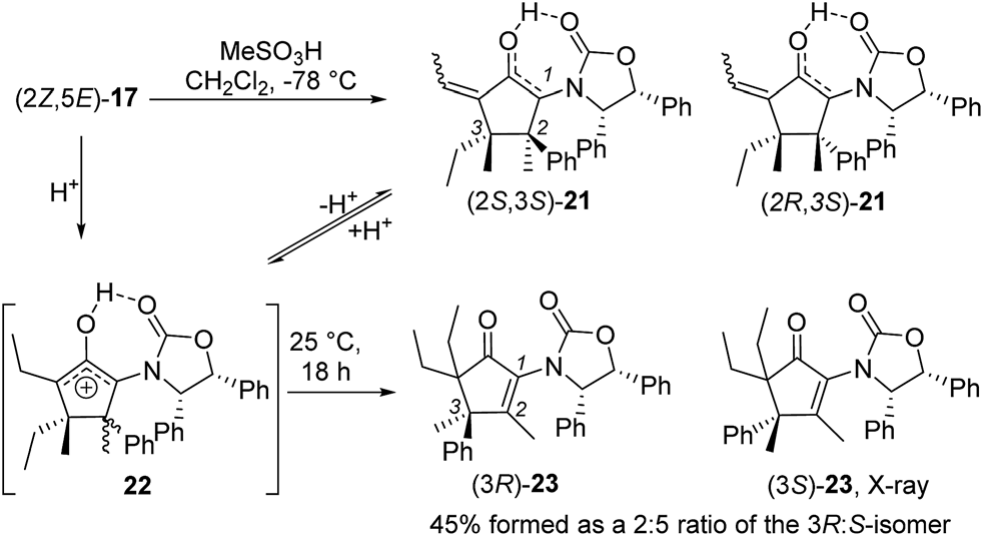
Nazarov cyclization of **17** in CH_2_Cl_2_.

Having achieved stereoselective Nazarov cyclizations leading to products with adjacent 3° and 4°-stereocentres, we next addressed the formation of vicinal 4°-stereocentres. To prepare the fully substituted Nazarov substrate **17** we developed a convergent carbometalation approach starting from two alkynes: ynamide **4a** and 3-hexyne (Scheme 2a). Cu-catalyzed addition of MeMgBr to **4a**, followed by *in situ* formylation with ethylformate, afforded **15** (52%) stereoselectively. Carboalumination of 3-hexyne to give **16**,^11^ followed by 1,2-addition of **16** to **15** and oxidation with DMP, gave divinyl ketone **17** (71%). The C2–C3 double bond retained its *Z* stereochemistry while the C5–C6 double bond was formed as a 3 : 1 *E* : *Z* mixture.^12^§
§The basis of the isomerization of the C5-alkenyl unit has not yet been fully discerned. Treatment of **16** with I_2_ gave only the expected *E-*iodoalkene, whereas 1,2-addition of **16** to **15** gives the corresponding carbinol (not shown) as ∼3 : 1 mixture of the *E*- and *Z*-isomer. Separation of these isomers proved challenging; however, a pure sample of (2*Z*,5*E*)-**17** was isolated in 24% yield (from **15**). We also prepared the fully substituted ketone **20** (Scheme 2b) bearing a tethered nucleophile (electron-rich aryl group). Access to **20** commenced with formation of vinyl bromide **19** from bromoalcohol **18**.^13^ Lithiation of **19**, followed by addition to a solution of **15** and AlMe_3_ (Lewis acid) and DMP oxidation of the crude carbinol (not shown) afforded **20** (44%) as a single alkene-stereoisomer.¶
¶In the absence of AlMe_3_ the reaction affords mostly an acyl migration product involving ring opening of the oxazolidinone:
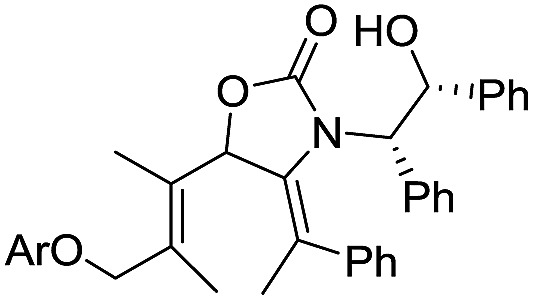




Nazarov cyclization of (2*Z*,5*E*)-**17** with MeSO_3_H (CH_2_Cl_2_, –78 °C) gave **21** as a complex mixture of C2,3-diastereomers, keto/enol-tautomers and *E*/*Z*-isomers (Scheme 3). Warming the mixture to ambient temperature resulted in a double Wagner–Meerwein shift of the C3-ethyl and C2-phenyl substituents in the reversibly formed oxyallyl cation **22** to give (3*R*)-**23** and (3*S*)-**23** in a 2 : 5 ratio (Scheme 3).^14^ The stereochemistry of these products was confirmed by X-ray crystallography of (3*S*)-**23**.‡ We believe that the origin of this epimeric mixture is partial double-bond isomerization of (2*Z*,5*E*)-**17** to (2*E*,5*E*)-**17** under the acidic conditions prior to Nazarov cyclization. While this isomerization was undesired, the rapid (<2 h) cyclisation of both isomers of **17** at –78 °C demonstrates the remarkable ability of the **Ox** group to activate the Nazarov reaction. Upon further experimentation with reaction conditions (acids and solvents) to avoid double-bond isomerization of (2*Z*,5*E*)-**17** to (2*E*,5*E*)-**17**, we found that treatment of (2*Z*,5*E*)-**17** with MeSO_3_H in 1,4-dioxane with mild heating gave cyclopentanone **24** stereoselectively in 52% isolated yield (eqn (3)). The stereochemistry of (*E*)- and (*Z*)-**24** were confirmed by X-ray crystallography and 2D NMR, respectively.‡ Replacing CH_2_Cl_2_ with 1,4-dioxane as solvent appears to exert different effects on the rates of the various competing reactions involved in the formation of **21**, **23** and **24** (Scheme 3 and eqn (3)). Solvation of MeSO_3_H by 1,4-dioxane likely reduces the rates of all of these reactions, however, its strongest effects appear to be the suppression of C2–C3 double-bond isomerization in **17** and Wagner–Meerwein rearrangement in **22**, leading to the observed stereo- and chemoselective formation of **24**.^15^ Cyclization of **20** (eqn (4)) under these conditions was also successful, yielding the intramolecularly trapped product **25** as the only product discernable by ^1^H-NMR (53% isolated yield). Conversion of **20** to **25** forms two new rings and three contiguous 4°-stereocentres, underscoring the effectiveness of the **Ox**-controlled Nazarov reaction for synthesis of structurally complex, 4°-stereocentre-containing scaffolds. The asymmetric formation of three contiguous 4°-stereocentres entirely from prochiral carbons is a rare transformation; a Diels–Alder reaction reported by Nicolaou *et al.* is the only other example known to us.^16^3
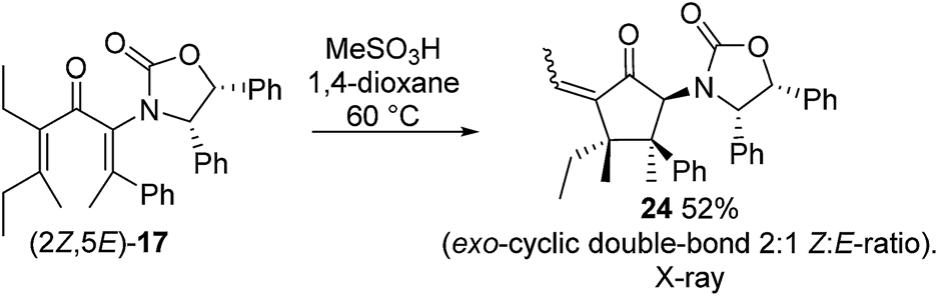

4
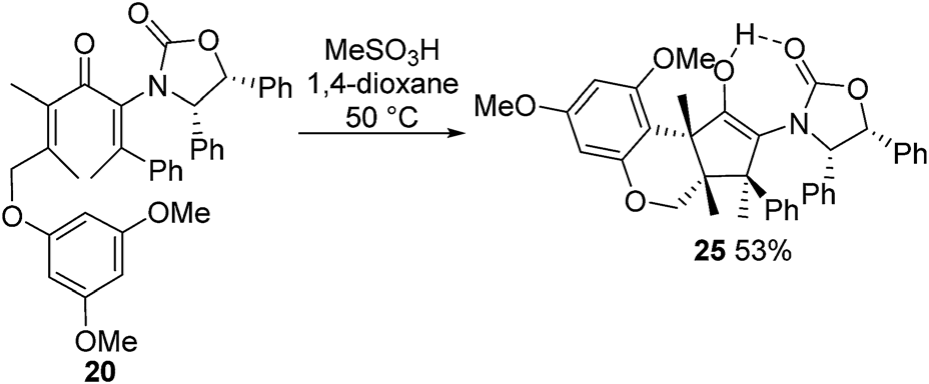



**Fig. 1:**
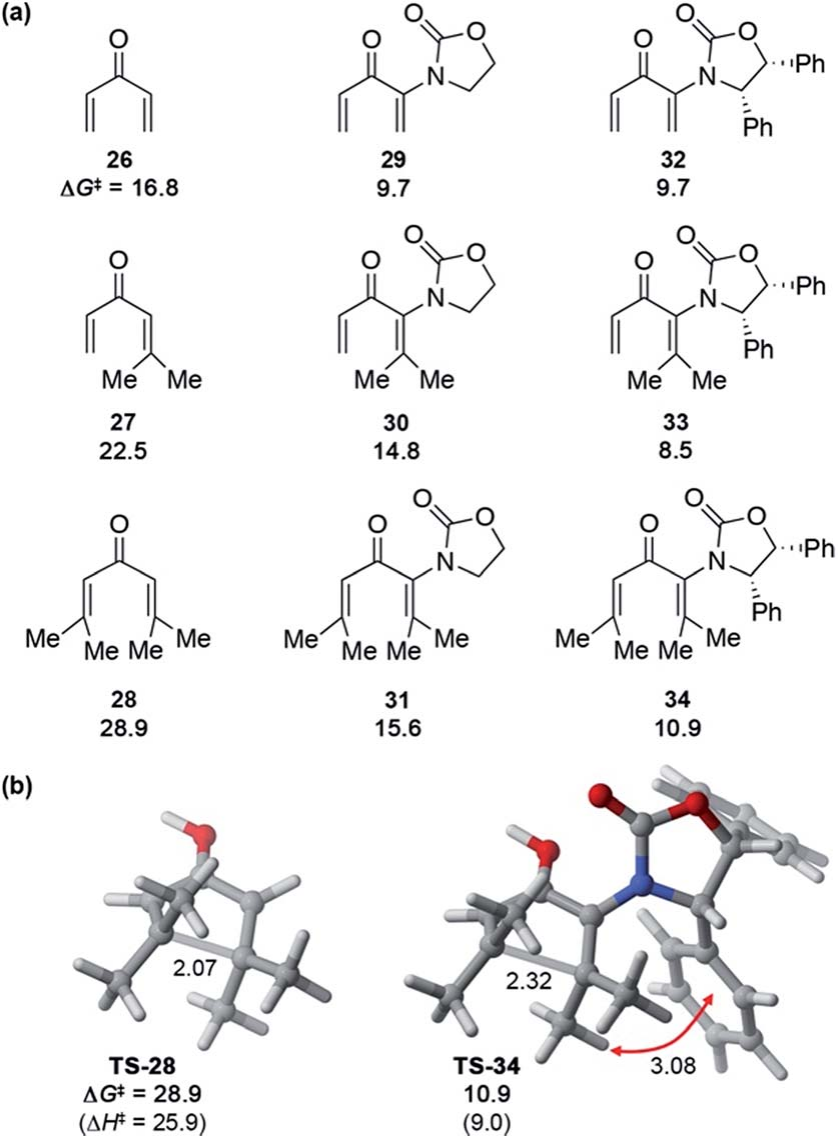
(a) Activation barriers for H^+^-catalyzed Nazarov cyclizations of model divinyl ketones **26–34** and (b) transition states for cyclizations of **28** and **34**, calculated with M06-2X/6-311+G(d,p)//M06-2X/6-31G(d) in implicit (SMD) dichloromethane. Distances in Å, Δ*H*^‡^ and Δ*G*^‡^ in kcal mol^–1^.

These **Ox**-promoted Nazarov cyclizations are remarkably facile, allowing efficient generation of sterically congested products at temperatures as low as –78 °C. This points to a powerful activating influence of the **Ox** auxiliary. In order to determine the origins of this activation, we performed DFT calculations (Fig. 1).‡ Calculations with M06-2X show that in the absence of an oxazolidinone, the activation energies (Δ*G*^‡^) for Nazarov cyclizations of **26–28** leading to zero, one, or two 4°-centres are 16.8, 22.5, and 28.9 kcal mol^–1^, respectively. Each new 4°-stereocentre raises the barrier by 6 kcal mol^–1^.‡ An achiral oxazolidinone devoid of Ph substituents (**OxH_2_**, see **29–31**) lowers the cyclization barrier by 7–13 kcal mol^–1^ (Δ*G*^‡^ = 9.7–15.6 kcal mol^–1^) relative to the oxazolidinone-free substrates, while the diphenyl-oxazolidinone (**Ox**, see **32–34**) provides further activation still, leading to cyclization barriers of only 8.5–10.9 kcal mol^–1^. These very low barriers are consistent with the facile ring closures observed for **5**, **17**, and **20**.

The transition states (TSs) for **OxH_2_**- and **Ox**-promoted cyclizations benefit from several stabilizing effects. Firstly, the nitrogen lone pair affords resonance stabilization of the incipient oxyallyl cation. Secondly, the oxazolidinone-containing TSs feature a longer forming C–C bond than the corresponding oxazolidinone-free TSs, leading to reduced steric repulsion between the Me groups about the forming C–C bond (see Fig. 1b). A third activating influence of **Ox** is evident from a comparison of the cyclizations of **33** and **34** (containing **Ox**) with those of **30** and **31** (containing **OxH_2_**). The two **Ox**-substituted TSs have Δ*G*^‡^ values about 6 kcal mol^–1^ lower than those of the corresponding **OxH_2_** derivatives. The additional activation by **Ox** can be traced to a CH–π interaction in the TS between the “inner” substituent on C2 (R^2^, rotating downwards) and the nearby Ph substituent on **Ox** (see red arrow in Fig. 1). Together, these three TS-stabilizing influences of **Ox** make it an exceptionally powerful activating group, capable of reducing the barrier for vicinal 4°-centre formation by almost 18 kcal mol^–1^ (**28***vs.***34**). Indeed, computations predict that when the R^1^ substituent is an aryl group, like in many of our substrates (**5**, **17**, and **20**) (with R^2^ = alkyl) the barrier for cyclization is even lower still.‡

## Conclusions

To conclude, carbometalation of **Ox**-ynamides affords direct access to highly substituted **Ox**-divinyl and -aryl vinyl ketones, which undergo exceptionally facile Nazarov cyclizations leading to 4°-stereocentre-containing cyclopentanoids. In addition to the powerful activating and stereodirecting influence of **Ox** in the Nazarov cyclization, the **Ox** auxiliary helps suppress undesired Wagner–Meerwein rearrangements in the intermediate oxyallyl cations, and facilitates nucleophilic trapping of these intermediates enabling rapid assembly of multiple stereocentres (including vicinal 4°-stereocentres) with excellent stereochemical control. Theoretical studies allowed us to discover the electronic origin of the strong activating effect of the **Ox**, which is traced to a combination of covalent (lone pair donation to the incipient oxyallyl cation and reduced steric crowding about the newly forming bond) and non-covalent (CH–π interaction) effects which are generally applicable across most of the divinyl (or aryl vinyl) ketones reported here.

## Conflicts of interest

There are no conflicts to declare.

## Supplementary Material

Supplementary information

Crystal structure data
