# Supplementary material for: Asymmetric synthesis of multiple quaternary stereocentre-containing cyclopentyls by oxazolidinone-promoted Nazarov cyclizations
Source: Chem Sci. 2018 Apr 20;9(20):4644–9. doi: 10.1039/c8sc00031j (PMC5969496; doi:10.1039/c8sc00031j)

This journal is © The Royal Society of Chemistry 2018

Electronic Supplementary Information

Asymmetric Synthesis of Multiple Quaternary Stereocentre Containing Cylopentyls by Oxazolidinone-Promoted Nazarov Cyclizations

Rohan Volpe, Romain J. Lepage Jonathan M. White, Elizabeth H. Krenske, and Bernard L. Flynn*

Table of Contents:

Crystallography (3*S*)-23 and (*E*)-24 .... S2

Notes on the Computational Investigation of the Ox-Promoted Nazarov Cyclization S4

Synthesis of Compounds S22

^{1}H, ^{13}C and 2D NMR Spectra S48

Crystallography

Intensity data were collected with an Oxford Diffraction SuperNova CCD diffractometer using Cu- Ka radiation, the temperature during data collection was maintained at 130.0(1) using an Oxford Cryosystems cooling device.

The structure was solved by direct methods and difference Fourier synthesis.[1] Thermal ellipsoid plots were generated using the program ORTEP-3[2] integrated within the WINGX[3] suite of programs.

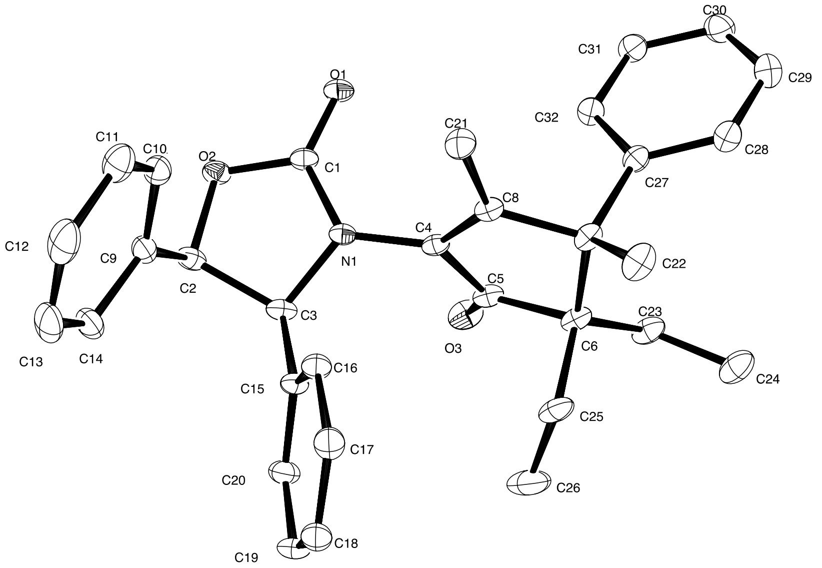

Crystal data for (*3S*)-**23**. $C_{32}H_{33}NO_3$, M = 479.59 T = 130.0(2) K, λ = 0.71073 Å, Trigonal, space group $P3_12_12$ a = 12.0961(4), c = 30.6664(11) Å, V = 3885.8(3) Å^3, Z = 6, D_c = 1.230 Mg M^{-3} μ(Mo-Kα) = 0.078 mm^{-1}, *F(000)* = 1536, crystal size 0.66 x 0.64 x 0.16 mm. θ_{max} = 36.46°, 52026 reflections measured, 12232 independent reflections (R_{int} = 0.051) the final R = 0.0546 [I > 2σ(I), 8765 data] and $wR(F^2)$ = 0.144 (all data) GOOF = 1.033, Absolute structure parameter 0.3(4).

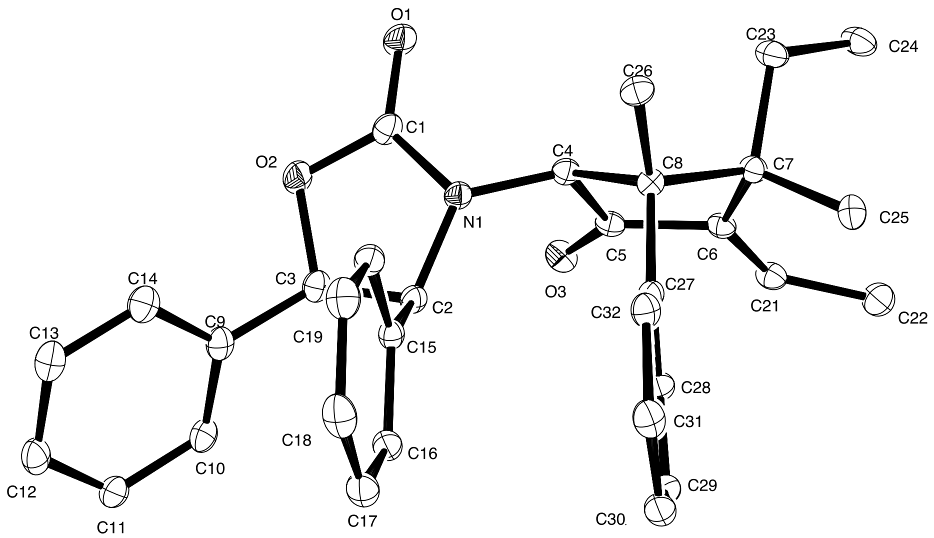

Crystal data for (*E*)-**24**. $C_{32}H_{33}NO_3$, *M* = 479.59 *T* = 130.0(2) K, λ = 1.54184 Å, Monoclinic, space group *P2₁* *a* = 10.2041(2), *b* = 12.0967(2), *c* = 10.8841(2) Å, β = 109.609(2)°, *V* = 1265.57(4) Å^3, *Z* = 2, D_c = 1.259 Mg M^{-3} μ(Cu-Kα) = 0.630 mm^{-1}, *F(000)* = 512, crystal size 0.50 x 0.35 x 0.23 mm. θ_{max} = 77.16°, 11875 reflections measured, 5308 independent reflections (R_{int} = 0.019) the final R = 0.0289 [I > 2σ(I), 5254 data] and *w*R(F^2) = 0.0742 (all data) GOOF = 1.081, Absolute structure parameter 0.0

---

[1] G. Sheldrick, *Acta Crystallogr. Section C*, **2015**, **71**, 3-8.

[2] Farrugia, L. J.; *J. Appl. Cryst.* **1997**, *30*, 565.

[3] Farrugia, L. J.; *J. Appl. Cryst.* **1999**, *32*, 837.

Notes on the Computational Investigation of the Ox-Promoted Nazarov Cyclization

The geometries of the transition states for H$^+$-catalyzed Nazarov cyclizations of divinyl ketones **26**–**34**, discussed in the paper, are shown in Figure S-1(a). Figure S-1(b) shows the TSs for cyclizations of additional substrates **35**–**37**, in which one substituent of the divinyl ketone bears Me and Ph substituents.

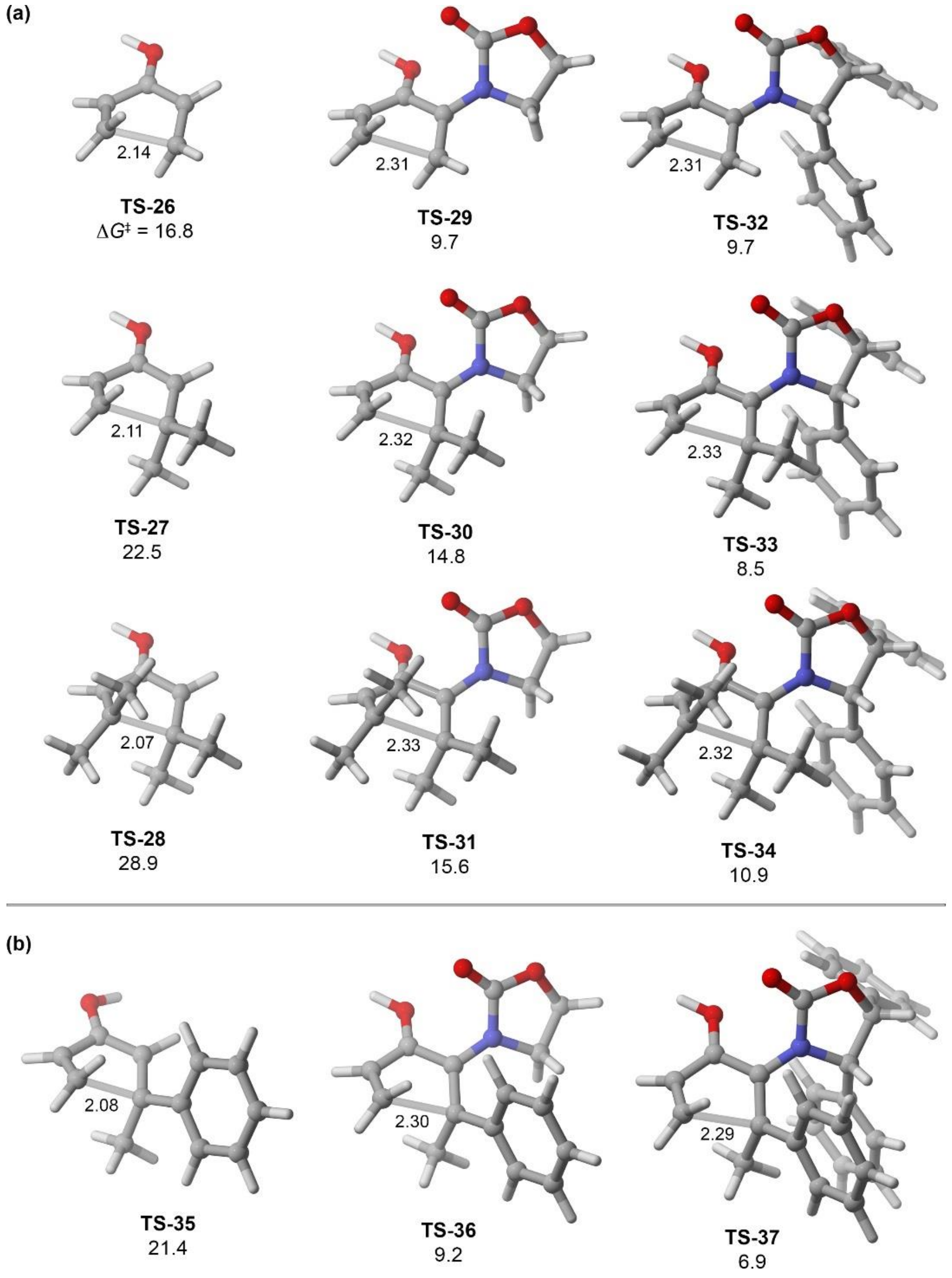

Figure S-1. Transition structures for Nazarov cyclizations of divinyl ketones **26**–**37**, calculated with M06-2X/6-311+G(d,p)//M06-2X/6-31G(d) in SMD implicit dichloromethane. Distances in Å, Δ*G*‡ in kcal/mol.

Experimentally, the Nazarov cyclizations of **Ox**-substituted divinyl ketones are performed under either Brønsted acid (TfOH) or Lewis acid (BF_3.THF) catalysis. To mimic both types of conditions, the calculations on the H^+-catalyzed cyclizations of **26**–**37** were performed with the OH group adopting a conformation that does not form a hydrogen bond to the oxazolidinone carbonyl oxygen. We have previously shown[1] that the lowest-energy TSs for Brønsted acid-catalyzed Nazarov cyclizations of other oxazolidinone-substituted divinyl ketones do not contain such an OH···O hydrogen bond, and in the TSs for BF_3-catalyzed cyclizations the BF_3 group likewise points away from the oxazolidinone. Moreover, our previous calculations showed that a range of divinyl ketone substituents and a range of different oxazolidinone auxiliaries (related to **Ox**) consistently favor anticlockwise conrotation by the divinyl ketone termini during ring closure. Thus, the TSs for cyclizations of **26**–**37** were calculated assuming a similar direction of conrotation.

The Nazarov cyclizations of **26**–**34** shown in Figure S-1(a) illustrate the effects of alkyl substitution on the cyclization barrier. In general, increasing substitution of the divinyl ketone termini by alkyl (e.g. Me) groups increases the cyclization barrier due to the increased steric crowding about the newly-forming bond. Two other factors also contribute to the higher barriers: (i) loss of the hyperconjugative stabilization of the pentadienyl cation reactant, and (ii) destabilization of the TS due to closed-shell electron repulsion between the inner methyl groups and the high-lying sigma orbital of the forming bond.[2]

Figure S-1(b) shows the effect of replacing a Me substituent at the “outer” (R^1) position with a Ph substituent (**35**–**37**). Many of our experimental substrates (**5**, **17**, and **20**) contain an aryl group *trans* and an alkyl group *cis* to the divinyl ketone carbonyl group, similar to this. In the absence of an oxazolidinone, the Ph substituent is predicted to lower the barrier by 1.1 kcal/mol relative to a Me group at the same position (**TS-35** vs **TS-27**). In principle, the Ph group might be expected to stabilize the reacting pentadienyl cation via conjugation, which might be expected to raise the barrier for electrocyclization, but the calculations indicate the opposite result. This is because the Ph group does not achieve full coplanarity with the pentadienyl cation in **35**; instead it lies at an angle of 28° relative to the plane of the pentadienyl cation. In the presence of an oxazolidinone substituent (either **OxH₂** or **Ox**), the Me/Ph-substituted divinyl ketones have very low electrocyclization barriers ($\Delta G^‡$ = 9.2 and 6.9 kcal/mol for **TS-36** and **TS-37**, respectively). In these two cases, reactant stabilization by the Ph substituent is further reduced, because the Ph group is twisted even further out of coplanarity with the pentadienyl cation (dihedrals 48–51°) due to steric clashing with the oxazolidinone.

1 B. L. Flynn, N. Manchala, E. H. Krenske, *J. Am. Chem. Soc.* **2013**, *135*, 9156–9163.
2 W. Kirmse, N. G. Rondan, K. N. Houk, *J. Am. Chem. Soc.* **1984**, *106*, 7989–7991.

Computational Methods

Density functional theory calculations were performed in Gaussian 09.[3] Geometries were optimized with M06-2X/6-31G(d)[4] in implicit solvent (dichloromethane) as modeled with the SMD solvation model.[5] Vibrational frequency calculations at this level were performed to confirm the nature of each species (local minimum or first-order saddle point) and to obtain thermochemical quantities. Subsequently, single-point calculations were performed with M06-2X/6-311+G(d,p) in SMD implicit dichloromethane. The thermochemical corrections obtained from the M06-2X/6-31G(d) frequencies were added to the M06-2X/6-311+G(d,p) potential energies to give Gibbs free energies in solution, corrected to a standard state of 298.15 K and 1 mol/L. The M06-2X calculations employed the ultrafine integration grid of Gaussian 09. Cartesian coordinates of reactants and transition states are given below, along with the following energies (all in CH_2Cl_2 at 298.15 K and 1 mol/L, and given in Hartree):

E: Sum of M06-2X/6-31G(d) electronic potential energy and free energy of solvation

G: M06-2X/6-31G(d) Gibbs free energy

E_{soln}: Sum of M06-2X/6-311+G(d,p) electronic potential energy and free energy of solvation

G_{soln}: Total Gibbs free energy in CH_2Cl_2 at the M06-2X/6-311+G(d,p)-SMD(CH_2Cl_2)//M06-2X/6-31G(d)-SMD(CH_2Cl_2) level of theory

3 M. J. Frisch, G. W. Trucks, H. B. Schlegel, G. E. Scuseria, M. A. Robb, J. R. Cheeseman, G. Scalmani, V. Barone, B. Mennucci, G. A. Petersson, H. Nakatsuji, M. Caricato, X. Li, H. P. Hratchian, A. F. Izmaylov, J. Bloino, G. Zheng, J. L. Sonnenberg, M. Hada, M. Ehara, K. Toyota, R. Fukuda, J. Hasegawa, M. Ishida, T. Nakajima, Y. Honda, O. Kitao, H. Nakai, T. Vreven, J. A. Montgomery, Jr., J. E. Peralta, F. Ogliaro, M. Bearpark, J. J. Heyd, E. Brothers, K. N. Kudin, V. N. Staroverov, T. Keith, R. Kobayashi, J. Normand, K. Raghavachari, A. Rendell, J. C. Burant, S. S. Iyengar, J. Tomasi, M. Cossi, N. Rega, J. M. Millam, M. Klene, J. E. Knox, J. B. Cross, V. Bakken, C. Adamo, J. Jaramillo, R. Gomperts, R. E. Stratmann, O. Yazyev, A. J. Austin, R. Cammi, C. Pomelli, J. W. Ochterski, R. L. Martin, K. Morokuma, V. G. Zakrzewski, G. A. Voth, P. Salvador, J. J. Dannenberg, S. Dapprich, A. D. Daniels, O. Farkas, J. B. Foresman, J. V. Ortiz, J. Cioslowski, and D. J. Fox, Gaussian 09, Revision E.01, Gaussian, Inc., Wallingford CT, 2013.

4 Y. Zhao, D. G. Truhlar, *Theor. Chem. Acc.* **2008**, *120*, 215–241.

5 A. V. Marenich, C. J. Cramer, D. G. Truhlar, *J. Phys. Chem. B* **2009**, *113*, 6378–6396.

Calculated Geometries and Energies

Protonated divinyl ketones

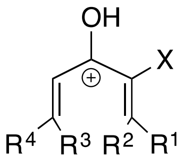

X = H, **OxH_2**, **Ox**

26

```
O   -0.601252    1.493342   -0.003706
C    0.008885    0.352471   -0.017695
C   -0.833970   -0.817923   -0.044370
C   -2.169429   -0.704141    0.030413
C    1.449052    0.333777   -0.008379
C    2.162366   -0.803446    0.027339
H    0.016644    2.256802    0.029145
H   -2.664134    0.259058    0.113704
H   -2.796553   -1.589511    0.014525
H   -0.357006   -1.786257   -0.120668
H    1.713662   -1.791206    0.049736
H    3.246757   -0.759268    0.040191
H    1.949230    1.299222   -0.020837
0 imaginary frequencies
E = -269.607375
G = -269.523868
```

E_{soln} = -269.687422
G_{soln} = -269.603916

27

```
O    1.723718   -1.518516    0.133653
C    0.879774   -0.541347   -0.056683
C    1.497387    0.752025   -0.279933
C    2.781804    0.967062    0.038708
C   -0.508167   -0.833380   -0.091910
C   -1.545822    0.053832    0.007126
C   -1.438192    1.528353    0.226166
C   -2.938091   -0.471941   -0.073981
H    0.892352    1.530592   -0.724131
H    3.398569    0.200528    0.498254
H    3.238579    1.933937   -0.146340
H   -0.595982    1.807875    0.860773
H   -2.362705    1.902826    0.671429
H   -1.321679    2.028175   -0.745043
H   -3.500438    0.112119   -0.812881
H   -3.436074   -0.308261    0.890202
H   -2.979614   -1.531361   -0.329243
H    1.266981   -2.366699    0.318947
H   -0.761889   -1.889223   -0.168138
0  imaginary frequencies
E = -348.211992
G = -348.076565
```

E_{soln} = -348.313561
G_{soln} = -348.178134

28

O	0.575843	-1.977449	0.020527
C	-0.024916	-0.822982	-0.136666
C	0.802412	0.324102	-0.290828
C	2.148635	0.379086	-0.059730
C	2.994588	-0.745827	0.446001
C	2.868803	1.658220	-0.324573
C	-1.455791	-0.822107	-0.227531
C	-2.298931	0.213821	0.030241
C	-3.768919	0.002530	-0.140999
C	-1.912379	1.574394	0.521965
H	-0.069258	-2.703938	0.152019
H	3.157598	-1.469284	-0.362527
H	3.966725	-0.371664	0.772631
H	2.513696	-1.288488	1.262921
H	3.706221	1.465234	-1.006540
H	3.312266	2.021294	0.611053
H	2.222566	2.429797	-0.745137
H	0.308506	1.216810	-0.652586
H	-0.994005	1.577151	1.110389
H	-2.725313	1.986680	1.125688
H	-1.778290	2.247831	-0.335026
H	-4.262267	0.072721	0.836043
H	-4.005114	-0.960523	-0.596220
H	-4.182149	0.810545	-0.756531
H	-1.898935	-1.781987	-0.487674

0 imaginary frequencies
E = -426.817416
G = -426.629355
E_{soln} = -426.939927
G_{soln} = -426.751866

29

C	2.126717	1.148239	0.238860
N	0.900096	0.364205	0.146965
C	1.182488	-0.890745	-0.380816
O	2.502880	-1.097924	-0.325559
C	3.143010	0.010506	0.342542
C	-0.363708	0.916579	-0.046215
C	-0.597410	2.210509	-0.322629
O	0.378678	-1.670547	-0.828882
C	-1.502621	0.047237	0.258753
C	-2.759607	0.188448	-0.435667
C	-3.876775	-0.458272	-0.066802
O	-1.291837	-0.802500	1.200441
H	-3.946249	-1.128402	0.786965
H	-4.792612	-0.319502	-0.632211
H	-2.757653	0.847784	-1.295313
H	-1.608222	2.594340	-0.394492
H	0.218781	2.919436	-0.420082
H	2.272652	1.751021	-0.664895
H	4.081539	0.216762	-0.168309
H	3.327111	-0.278580	1.379469
H	2.119591	1.791953	1.119605
H	-2.045944	-1.411486	1.364356

0 imaginary frequencies
E = -590.836420
G = -590.689902
E_{soln} = -591.010760
G_{soln} = -590.864242

30

C	1.510411	-1.213651	1.100292
N	0.922010	-0.213362	0.213392
C	1.793541	0.139147	-0.794869
O	2.989086	-0.431102	-0.555390
C	2.991360	-1.021980	0.757433
C	-0.419491	0.217197	0.207686

C	-0.700303	1.565880	0.305775
C	-2.031291	2.195172	0.065103
O	1.558222	0.844759	-1.744798
C	-1.408738	-0.793945	0.025985
C	-2.830145	-0.657714	0.309487
C	-3.745420	-1.356799	-0.374380
O	-0.942453	-1.947755	-0.356795
C	0.397741	2.526493	0.598646
H	-3.493836	-2.017312	-1.202220
H	-4.800565	-1.244597	-0.147799
H	-3.119312	0.047205	1.080353
H	-2.698999	1.610595	-0.565464
H	-1.872590	3.175313	-0.394884
H	-2.519790	2.380173	1.032036
H	0.786737	2.902112	-0.358148
H	0.000777	3.384339	1.148649
H	1.224243	2.067936	1.143842
H	1.295844	-0.988413	2.146243
H	3.480606	-0.325079	1.442546
H	1.157298	-2.219622	0.856522
H	3.549013	-1.955683	0.710031
H	-1.628334	-2.649447	-0.336543

0 imaginary frequencies
E = -669.437629
G = -669.241194
E_{soln} = -669.633478
G_{soln} = -669.437044

31

C	1.838808	-1.273590	1.251887
N	1.508473	-0.240175	0.275490
C	2.423982	-0.212419	-0.753521
O	3.448225	-1.033013	-0.447900
C	3.316064	-1.492600	0.909302
C	0.288380	0.464009	0.199965
C	0.296164	1.835256	0.157330
C	1.575366	2.578226	0.352814
O	2.358104	0.432096	-1.771310
C	-0.897048	-0.346952	0.076106
C	-2.205505	0.080049	0.460791
C	-3.385586	-0.457117	0.038134
C	-4.664327	0.087486	0.579500
O	-0.653349	-1.566053	-0.331919
C	-3.539129	-1.554639	-0.972334
C	-0.880661	2.703051	-0.157889
H	-1.432651	-2.151144	-0.247046
H	-3.572371	-2.527071	-0.458783
H	-2.743803	-1.573443	-1.722579
H	-4.495381	-1.446935	-1.489914
H	-5.223910	0.564734	-0.234744
H	-5.287503	-0.739408	0.940725
H	-4.508202	0.809056	1.382599
H	-1.673494	2.200074	-0.710005
H	-2.238432	0.921765	1.143078
H	-0.531195	3.561906	-0.739211
H	-1.297197	3.103982	0.775965
H	2.011868	2.789113	-0.632431
H	1.371360	3.539640	0.833126
H	2.305249	2.012251	0.934037
H	1.700669	-0.910802	2.271754
H	3.973136	-0.887484	1.539083
H	1.240297	-2.176098	1.095147
H	3.619368	-2.537719	0.947294

0 imaginary frequencies
E = -748.042781
G = -747.792282
E_{soln} = -748.259729
G_{soln} = -748.009230

32

C	3.116567	-0.931353	-0.446462
C	1.795248	-1.383054	-0.465716
C	1.300328	-2.112151	0.616110
C	2.120148	-2.376206	1.712101
C	3.433228	-1.912015	1.734444
C	3.931789	-1.189665	0.651119
C	0.916864	-0.997575	-1.629970
C	0.151281	0.352684	-1.447968
N	-1.086585	-0.169542	-0.873504
C	-1.290164	-1.479897	-1.279344
O	-0.148282	-1.954632	-1.800416
C	0.843172	1.380624	-0.591151
C	0.760242	1.343799	0.803193
C	1.465421	2.267564	1.568231
C	2.258955	3.232102	0.947261
C	2.338343	3.275556	-0.442549
C	1.626186	2.355583	-1.209762
O	-2.316487	-2.104937	-1.193466
C	-2.182498	0.614820	-0.504800
C	-2.373650	1.883768	-0.906374
C	-3.049447	0.057760	0.529086
C	-4.422881	0.475014	0.692112
C	-5.092912	0.288159	1.839951
O	-2.486156	-0.811430	1.292742
H	-3.194305	2.478390	-0.521880
H	-4.660419	-0.165959	2.728767
H	-1.681621	2.366032	-1.589270
H	-6.120099	0.626386	1.929547
H	-4.882882	0.975974	-0.151318
H	-0.047582	0.775055	-2.441796
H	1.491173	-0.978116	-2.557045
H	1.683287	2.389688	-2.295260
H	2.949288	4.028056	-0.931530
H	2.810256	3.950394	1.546341
H	1.398494	2.232928	2.651307
H	0.152814	0.583194	1.287876
H	3.502952	-0.366375	-1.291543
H	4.956886	-0.832332	0.658129
H	4.068979	-2.118858	2.589951
H	1.730932	-2.948617	2.548661
H	0.280229	-2.486637	0.609437
H	-3.102212	-1.227513	1.936210

0 imaginary frequencies
E = -1052.769212
G = -1052.472473
E_{soln} = -1053.057526
G_{soln} = -1052.760788

33

C	0.699570	2.821113	-0.971086
C	0.544348	1.525399	-0.476736
C	0.736488	1.287601	0.888945
C	1.081865	2.331729	1.740593
C	1.244101	3.623260	1.237777
C	1.055422	3.866293	-0.119759
C	0.185600	0.421864	-1.436833
C	1.302535	-0.604274	-1.806635
O	0.550389	-1.790411	-2.120870
C	-0.631688	-1.773070	-1.463171
N	-0.819965	-0.519578	-0.933844
C	2.299544	-0.890064	-0.709834
C	3.400036	-0.042715	-0.561850
C	4.296001	-0.229168	0.486252
C	4.101116	-1.270746	1.392915
C	3.013556	-2.127292	1.239541

C	2.114085	-1.940162	0.190578
C	-1.974816	-0.190797	-0.182837
C	-1.996321	-0.717722	1.146833
O	-1.074196	-1.599704	1.393259
O	-1.370482	-2.721136	-1.379739
C	-3.006612	0.520832	-0.757117
C	-2.897150	0.901681	-2.194819
C	-4.246016	0.976683	-0.054777
C	-2.918577	-0.357705	2.216295
C	-3.361358	-1.280405	3.079670
H	-3.105047	-2.334936	2.996655
H	-4.041234	-1.003339	3.878357
H	-3.237890	0.675399	2.277698
H	-0.166328	0.876501	-2.371227
H	1.812363	-0.285085	-2.716747
H	0.542664	3.011894	-2.030291
H	1.177835	4.868809	-0.517979
H	1.516673	4.436063	1.903970
H	1.232748	2.136574	2.797986
H	0.640917	0.278145	1.280339
H	3.546288	0.773770	-1.265587
H	5.147298	0.436061	0.593542
H	4.800716	-1.419193	2.209922
H	2.865642	-2.948739	1.934292
H	1.279361	-2.625181	0.072432
H	-3.888060	0.965940	-2.647387
H	-2.449264	1.905910	-2.251287
H	-2.271119	0.214791	-2.766241
H	-4.702486	0.181176	0.539767
H	-4.011548	1.808961	0.620653
H	-4.973987	1.334709	-0.782730
H	-1.071851	-1.875175	2.335921

0 imaginary frequencies
E = -1131.366243
G = -1131.017332
E_{soln} = -1131.676529
G_{soln} = -1131.327618

34

C	-1.443070	-2.769280	1.053586
C	-1.110373	-1.504383	0.567576
C	-0.846939	-0.472041	1.475536
C	-0.921818	-0.705288	2.844679
C	-1.264609	-1.970526	3.323172
C	-1.527626	-3.001661	2.425958
C	-1.038771	-1.291270	-0.922060
C	-2.157043	-0.434949	-1.594535
O	-1.484888	0.114694	-2.741568
C	-0.152762	0.186550	-2.504956
N	0.125931	-0.517094	-1.360756
C	-2.723681	0.668967	-0.735589
C	-3.750851	0.363572	0.160221
C	-4.244600	1.336316	1.023685
C	-3.718010	2.627351	0.992901
C	-2.703997	2.939815	0.090502
C	-2.206612	1.964614	-0.773431
C	1.442803	-0.631346	-0.845969
C	1.905684	0.524114	-0.114037
O	1.220803	1.608375	-0.360507
O	0.622395	0.779461	-3.211882
C	2.190638	-1.754837	-1.086616
C	1.576223	-2.863812	-1.881565
C	3.587075	-1.994878	-0.605685
C	2.954098	0.512973	0.860640
C	3.742739	1.573834	1.188667
C	4.757541	1.420108	2.272088
C	3.725969	2.913073	0.515436
H	1.452054	2.340960	0.246919

H	3.119762	-0.434290	1.360771
H	-1.038176	-2.272264	-1.413277
H	-2.953601	-1.085840	-1.957994
H	-1.636897	-3.577889	0.352650
H	-1.791862	-3.989550	2.790497
H	-1.324842	-2.150036	4.392281
H	-0.719008	0.103313	3.540401
H	-0.606427	0.522214	1.107595
H	-4.155245	-0.645830	0.187641
H	-5.042177	1.088359	1.717310
H	-4.104293	3.388822	1.663671
H	-2.301579	3.947727	0.051998
H	-1.425858	2.223624	-1.482755
H	2.342969	-3.482567	-2.349137
H	0.999804	-3.505897	-1.199320
H	0.889986	-2.489896	-2.645113
H	5.761885	1.487417	1.835727
H	4.668311	2.255971	2.976132
H	4.660090	0.475688	2.809260
H	3.362449	2.884176	-0.514728
H	3.101533	3.607224	1.096240
H	4.734503	3.334469	0.514722
H	4.179070	-1.084304	-0.513779
H	3.555325	-2.484571	0.377164
H	4.092174	-2.679716	-1.289157

0 imaginary frequencies
E = -1209.971347
G = -1209.569999
E_{soln} = -1210.302662
G_{soln} = -1209.901314

35

C	-4.544566	0.569370	-0.020264
C	-3.233975	0.618467	0.256162
C	-2.405318	-0.568989	0.150621
C	-0.992690	-0.624100	0.107443
C	-0.118140	0.403122	-0.163427
C	1.325365	0.131294	-0.087351
O	-3.076740	-1.691534	0.173978
C	-0.535005	1.792269	-0.545563
H	-2.761410	1.536494	0.577316
H	-5.027689	-0.342142	-0.358854
H	-5.159272	1.458635	0.073257
H	-1.452526	1.790308	-1.134550
H	0.247417	2.276787	-1.130219
H	-0.704679	2.398073	0.354417
H	-2.496253	-2.470753	0.043148
H	-0.575265	-1.603318	0.328125
C	2.204603	1.175636	0.250996
C	3.565941	0.936388	0.383768
C	4.077854	-0.338201	0.144613
C	3.221038	-1.376379	-0.220188
C	1.855566	-1.149437	-0.326788
H	1.818475	2.170949	0.444758
H	4.228764	1.746948	0.668512
H	5.144276	-0.520739	0.233665
H	3.619098	-2.364316	-0.427648
H	1.208944	-1.961314	-0.643884

0 imaginary frequencies
E = -539.880105
G = -539.694843
E_{soln} = -540.026643
G_{soln} = -539.841382

36

C	-1.996544	0.083362	-0.932619
C	-1.569947	-0.955834	-0.089738

C	-2.518387	-1.668375	0.662223
C	-3.859978	-1.310022	0.614213
C	-4.275835	-0.276588	-0.224277
C	-3.345810	0.405541	-1.008493
C	-0.156280	-1.361440	-0.008754
C	0.104138	-2.836378	-0.081154
C	0.823970	-0.396813	0.140166
C	2.230626	-0.651051	0.191456
O	2.959389	0.427905	0.201797
N	0.459084	0.964793	0.287520
C	0.323253	1.605542	1.592045
C	0.007858	3.033608	1.139735
O	0.572053	3.127384	-0.182487
C	0.741724	1.891591	-0.688717
O	1.070401	1.663683	-1.828096
C	2.911512	-1.939765	0.245800
C	4.063949	-2.129956	-0.409262
H	4.513715	-1.371587	-1.047219
H	4.570146	-3.088108	-0.358671
H	2.456792	-2.734867	0.822401
H	0.909099	-3.062363	-0.785075
H	-0.793278	-3.360451	-0.406899
H	0.394494	-3.228273	0.901313
H	-0.492690	1.163269	2.166974
H	-1.069232	3.202190	1.062279
H	1.257551	1.540567	2.162035
H	0.466115	3.800397	1.762192
H	-2.197951	-2.482235	1.306496
H	-4.582621	-1.845961	1.220940
H	-5.327136	-0.010819	-0.276393
H	-3.671529	1.191242	-1.682445
H	-1.280115	0.595126	-1.566691
H	3.912823	0.226014	0.320673

0 imaginary frequencies
E = -861.101988
G = -860.853415
E_{soln} = -861.343043
G_{soln} = -861.094470

37

C	3.048118	-0.185067	-1.493073
C	2.950774	-0.209536	-0.092749
C	3.485281	-1.289343	0.624398
C	4.064500	-2.355233	-0.056356
C	4.159520	-2.326502	-1.446720
C	3.666609	-1.233615	-2.161697
C	2.303743	0.896868	0.632855
C	3.072298	1.471405	1.782952
C	1.046741	1.304025	0.224033
C	0.365584	2.464936	0.708665
C	0.788964	3.377748	1.765874
C	0.606075	4.697218	1.630658
N	0.316241	0.546169	-0.722561
C	-0.041412	-0.870387	-0.593178
C	-1.107109	-0.946128	-1.730153
O	-0.654528	0.063885	-2.651994
C	0.068150	0.996940	-1.995561
C	-0.468264	-1.273888	0.792788
C	-0.029028	-2.495924	1.302954
C	-0.423928	-2.915174	2.572858
C	-1.249583	-2.104662	3.346925
C	-1.682356	-0.875564	2.847282
C	-1.295212	-0.461912	1.576972
C	-2.527288	-0.670034	-1.299751
C	-3.289225	-1.719373	-0.780202
C	-4.574393	-1.485495	-0.301052
C	-5.111385	-0.199187	-0.344502
C	-4.360469	0.846596	-0.875909

C	-3.071285	0.613842	-1.353669
O	0.432429	2.036602	-2.486735
O	-0.755463	2.720471	0.100530
H	0.199962	5.147345	0.727060
H	0.911105	5.375290	2.420684
H	1.248709	2.962914	2.652829
H	0.809345	-1.494956	-0.894701
H	-1.038253	-1.908708	-2.238699
H	0.626037	-3.121911	0.701438
H	-0.080021	-3.870698	2.956750
H	-1.554959	-2.426403	4.337964
H	-2.327391	-0.241068	3.447510
H	-1.653212	0.485693	1.181463
H	-2.867036	-2.720976	-0.740869
H	-5.158016	-2.307189	0.102652
H	-6.115251	-0.015768	0.026386
H	-4.779040	1.847402	-0.925102
H	-2.501113	1.434392	-1.779486
H	3.217745	2.548609	1.653863
H	2.546651	1.309647	2.730469
H	4.053791	1.003122	1.845618
H	3.412887	-1.309334	1.708351
H	4.450122	-3.203356	0.500101
H	4.626260	-3.152201	-1.974675
H	3.760546	-1.200816	-3.242315
H	2.668304	0.669480	-2.046734
H	-1.222892	3.483336	0.505112

0 imaginary frequencies
E = -1323.034980
G = -1322.635230
E_{soln} = -1323.390017
G_{soln} = -1322.990268

Transition states for H^+-catalyzed Nazarov cyclizations

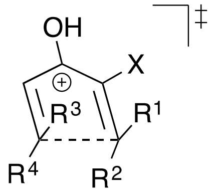

X = H, **OxH$_2$**, **Ox**

TS-26

O	2.069746	-0.127606	-0.011565
C	0.752313	-0.024798	-0.000007
C	-0.061860	-1.168324	0.112694
C	-1.391442	-1.011933	-0.249372
C	0.000328	1.159139	-0.109631
C	-1.338207	1.067459	0.249728
H	2.493431	0.748142	0.074592
H	0.309624	-2.082747	0.566899
H	-1.678417	-0.517591	-1.176281
H	-2.148840	-1.670435	0.170443
H	-1.650591	0.597539	1.180401
H	-2.060334	1.759513	-0.178454
H	0.410372	2.057173	-0.565562

1 imaginary frequency
E = -269.579733
G = -269.496499

E_{soln} = -269.660451
G_{soln} = -269.577217

TS-27

```
O   -2.552097   -0.804581    0.208702
C   -1.366010   -0.243086   -0.001363
C   -1.114222    1.134306   -0.221849
C    0.110970    1.415865   -0.804840
C   -0.193274   -0.985921   -0.027006
C    1.029822   -0.265609    0.082237
C    2.214382   -0.810646   -0.664005
C    1.404601    0.490659    1.348300
H   -3.274074   -0.186469   -0.010624
H   -1.780351    1.909644    0.150649
H    0.443478    0.878977   -1.689487
H    0.561888    2.396601   -0.668529
H    0.534892    0.860869    1.891245
H    1.933283   -0.236670    1.977592
H    2.096251    1.308516    1.138255
H    2.942890   -0.016476   -0.849383
H    2.703924   -1.560609   -0.030053
H    1.930748   -1.278288   -1.608583
H   -0.193764   -2.052856   -0.239540
```

1 imaginary frequency
E = -348.175982
G = -348.039978
E_{soln} = -348.278228
G_{soln} = -348.142225

TS-28

```
O   -2.989313   -0.138987   -0.009558
C   -1.659588   -0.039807   -0.001751
C   -0.890297    1.075887    0.335318
C    0.464768    1.038055   -0.095674
C    0.805176    1.074769   -1.581572
C    1.468708    1.779591    0.744262
C   -0.836008   -1.118796   -0.341551
C    0.511407   -1.026296    0.095475
C    0.845836   -1.044399    1.583663
C    1.552717   -1.722129   -0.738586
H   -3.399875    0.741558    0.070020
H   -1.265081    1.880241    0.967124
H    0.808867    2.132273   -1.870640
H    0.076791    0.544325   -2.195910
H    1.807229    0.681359   -1.765663
H    1.500039    2.823763    0.409563
H    1.224197    1.759104    1.807849
H    2.470782    1.364828    0.593540
H    0.092160   -0.544534    2.192961
H    1.829550   -0.608938    1.771630
H    0.892076   -2.100238    1.874749
H    2.531331   -1.253536   -0.590923
H    1.308241   -1.721668   -1.802422
H    1.639413   -2.760829   -0.397100
H   -1.177534   -1.927061   -0.985818
```

1 imaginary frequency
E = -426.772840
G = -426.582190
E_{soln} = -426.896457
G_{soln} = -426.705807

TS-29

```
C    1.750048   -1.394794   -0.323073
N    0.719488   -0.373607   -0.137487
C    1.269379    0.755847    0.487381
O    2.601283    0.643887    0.463213
```

C	2.993775	-0.506287	-0.316048
C	-0.630146	-0.567449	-0.244344
C	-1.233320	-1.808068	-0.110556
O	0.659978	1.659354	0.991581
C	-1.508287	0.554827	-0.477055
C	-2.798326	0.346111	0.009377
C	-2.963136	-0.678317	0.932034
O	-1.070555	1.577241	-1.176495
H	-1.673049	2.343830	-1.103151
H	-2.266776	-0.844450	1.745932
H	-3.938096	-1.148729	1.041253
H	-3.649434	0.872166	-0.415648
H	-2.109492	-2.073248	-0.690186
H	-0.670069	-2.635348	0.315568
H	3.852211	-0.964921	0.170453
H	1.748063	-2.103425	0.510503
H	3.258688	-0.163055	-1.318318
H	1.605970	-1.922649	-1.266678

1 imaginary frequency
E = -590.820481
G = -590.674814
E_{soln} = -590.994445
G_{soln} = -590.848778

TS-30

O	0.183489	-2.121217	-1.122166
C	0.940659	-1.266114	-0.458008
C	2.240931	-1.484198	0.025695
C	2.678639	-0.617303	1.007411
H	2.031922	-0.290898	1.814035
H	3.745336	-0.477734	1.170922
H	2.899320	-2.213747	-0.439958
H	0.508772	-3.035732	-1.015203
C	0.465894	0.046729	-0.196985
C	1.423632	1.072396	0.029631
C	2.532127	1.384529	-0.947197
C	1.046923	2.211166	0.923812
H	2.799968	0.536804	-1.577368
H	3.417443	1.767113	-0.434912
H	2.154973	2.191347	-1.589984
H	1.948715	2.587893	1.416613
H	0.309426	1.923978	1.676539
H	0.645935	3.041167	0.326644
N	-0.883923	0.271408	-0.073714
C	-1.738095	-0.647581	0.547104
O	-1.407677	-1.623949	1.164055
O	-3.001534	-0.242903	0.367281
C	-3.042538	0.847715	-0.575568
C	-1.635107	1.445051	-0.522111
H	-1.568658	2.258444	0.205805
H	-3.818817	1.540196	-0.256525
H	-1.282206	1.784496	-1.496554
H	-3.277275	0.437038	-1.560124

1 imaginary frequency
E = -669.417595
G = -669.217798
E_{soln} = -669.613313
G_{soln} = -669.413517

TS-31

O	-0.328154	-1.927543	-1.578001
C	0.477192	-1.132858	-0.882943
C	1.804727	-1.331073	-0.557442
C	2.340324	-0.513369	0.467274
C	3.790130	-0.153079	0.394615
C	1.753281	-0.468159	1.856023
C	-0.004224	0.144110	-0.441568

C	0.937081	1.185003	-0.301004
C	0.657825	2.274417	0.687790
C	1.899242	1.575038	-1.397210
H	2.448018	-1.988767	-1.139182
H	0.016630	-2.840088	-1.591412
H	0.697051	-0.741909	1.880829
H	2.313154	-1.204570	2.448421
H	1.907567	0.504970	2.329745
H	4.360976	-0.883079	0.984215
H	4.171877	-0.150023	-0.627926
H	3.960585	0.824924	0.857717
H	2.099666	0.766806	-2.101007
H	1.436147	2.405567	-1.945684
H	2.838596	1.951323	-0.983487
H	1.610429	2.655109	1.073385
H	0.158156	3.121291	0.198669
H	0.040753	1.932843	1.522266
N	-1.320016	0.283480	-0.080608
C	-2.037621	-0.746722	0.539060
O	-3.328477	-0.400661	0.586705
O	-1.581021	-1.764826	0.987436
C	-3.553040	0.786219	-0.202275
C	-2.178085	1.453209	-0.279615
H	-2.037195	2.185637	0.519272
H	-4.300063	1.392158	0.306584
H	-1.985944	1.914814	-1.248758
H	-3.916062	0.476461	-1.184749

1 imaginary frequency
E = -748.020741
G = -747.767532
E_{soln} = -748.237543
G_{soln} = -747.984335

TS-32

C	1.502988	-2.069392	0.536925
C	1.829648	-1.288224	-0.572193
C	3.104214	-0.725687	-0.669493
C	4.039447	-0.926202	0.340814
C	3.708485	-1.699413	1.452853
C	2.442974	-2.273659	1.545906
C	0.815880	-0.962477	-1.640052
C	-0.038574	0.314213	-1.364614
N	-1.172100	-0.314462	-0.677823
C	-1.296345	-1.650742	-1.077337
O	-0.180303	-2.001726	-1.730272
C	0.635205	1.405629	-0.575327
C	1.190057	2.483637	-1.265813
C	1.879543	3.479528	-0.576570
C	2.004502	3.407640	0.808481
C	1.438799	2.338630	1.503706
C	0.756957	1.339678	0.816484
O	-2.244377	-2.365211	-0.902498
C	-2.171640	0.342443	-0.009508
C	-2.504676	1.666268	-0.253591
C	-2.936551	-0.343155	0.997741
C	-4.204386	0.208412	1.186517
C	-4.683018	1.040558	0.183950
O	-2.372346	-1.314247	1.682026
H	-2.812392	2.326295	0.548931
H	-4.573053	0.805675	-0.868745
H	-3.035630	-1.814698	2.197779
H	-2.161294	2.149058	-1.166194
H	-5.432902	1.790963	0.424469
H	-4.726532	0.096344	2.133421
H	-0.376639	0.713357	-2.327823
H	1.293481	-0.884449	-2.617300
H	1.085202	2.539491	-2.346744
H	2.312282	4.312532	-1.121769

H	2.537723	4.184753	1.347484
H	1.531905	2.280843	2.583751
H	0.327442	0.501521	1.360551
H	3.359069	-0.119808	-1.535912
H	5.027176	-0.483414	0.257721
H	4.437875	-1.859257	2.241026
H	2.184036	-2.884891	2.405065
H	0.522111	-2.529721	0.621714

1 imaginary frequency
E = -1052.753035
G = -1052.457467
E_{soln} = -1053.040909
G_{soln} = -1052.745342

TS-33

C	-2.214549	-1.853132	-0.426305
C	-2.326223	-0.941372	0.623962
C	-3.398630	-0.046802	0.649819
C	-4.341092	-0.050303	-0.373505
C	-4.222049	-0.955142	-1.427897
C	-3.161579	-1.857483	-1.449494
C	-1.274889	-0.843135	1.701734
C	-0.130338	0.180291	1.415296
N	0.830410	-0.723022	0.770839
C	0.592227	-2.045758	1.143757
O	-0.563847	-2.091193	1.825991
C	-0.478956	1.381191	0.573980
C	-0.659273	2.613667	1.202591
C	-0.997864	3.742533	0.457697
C	-1.146139	3.644584	-0.923377
C	-0.962568	2.414995	-1.557511
C	-0.632808	1.286540	-0.813934
O	1.297210	-2.992604	0.925006
C	1.866260	-0.358155	-0.058092
C	2.665989	0.785828	0.217816
C	2.187044	-1.155423	-1.180656
C	3.517733	-0.994139	-1.607475
C	4.399151	-0.486510	-0.675384
O	1.253979	-1.913800	-1.731176
C	2.885800	1.887865	-0.788402
H	4.372105	-0.791284	0.365231
H	1.644320	-2.578463	-2.331026
C	2.830337	1.202448	1.644650
H	5.317433	-0.004446	-1.004035
H	3.793433	-1.141042	-2.649208
H	0.272133	0.517535	2.377863
H	-1.729652	-0.629652	2.669695
H	-0.531245	2.689011	2.280006
H	-1.138034	4.696252	0.956885
H	-1.403563	4.523521	-1.506432
H	-1.080671	2.334076	-2.633629
H	-0.503921	0.328391	-1.312629
H	-3.487426	0.662784	1.469018
H	-5.170251	0.650031	-0.346129
H	-4.958007	-0.960679	-2.226157
H	-3.069957	-2.571342	-2.262467
H	-1.398988	-2.570311	-0.452153
H	3.818232	1.658146	1.767716
H	2.094737	1.979666	1.893199
H	2.722178	0.366684	2.339300
H	3.860232	2.362990	-0.655838
H	2.761159	1.559472	-1.820330
H	2.118628	2.645050	-0.568948

1 imaginary frequency
E = -1131.352989
G = -1131.004886
E_{soln} = -1131.662196
G_{soln} = -1131.314094

TS-34

C	2.543581	-1.872245	0.551446
C	2.702175	-1.053464	-0.566986
C	3.827898	-0.233063	-0.668666
C	4.777267	-0.215193	0.348175
C	4.611771	-1.025936	1.470635
C	3.498138	-1.856662	1.567279
C	1.647271	-0.969273	-1.641541
C	0.556476	0.127952	-1.414176
N	-0.466450	-0.689047	-0.749764
C	-0.283538	-2.042434	-1.035216
O	0.878007	-2.187839	-1.689296
C	0.969525	1.337246	-0.613979
C	1.253593	2.525234	-1.287888
C	1.667446	3.654757	-0.583141
C	1.787511	3.603385	0.803029
C	1.497734	2.419452	1.482651
C	1.092542	1.289712	0.779204
O	-1.039619	-2.939616	-0.774564
C	-1.485926	-0.234254	0.050910
C	-2.219395	0.934886	-0.247833
C	-1.874528	-0.990198	1.198968
C	-3.206316	-0.801395	1.519834
C	-4.042251	-0.300734	0.492480
O	-0.955336	-1.697281	1.847683
C	-2.443341	2.031276	0.764171
C	-4.202989	-1.005118	-0.833323
H	-1.371493	-2.335070	2.457792
C	-2.297088	1.387828	-1.672586
C	-5.204676	0.557158	0.881651
H	-3.573171	-0.905080	2.539398
H	0.190424	0.449117	-2.395751
H	2.104357	-0.835691	-2.622604
H	1.147066	2.565563	-2.369372
H	1.888318	4.573368	-1.117807
H	2.103755	4.483241	1.354916
H	1.591216	2.374761	2.563296
H	0.880864	0.366831	1.314771
H	3.952680	0.401979	-1.542636
H	5.647846	0.427783	0.262961
H	5.353018	-1.014911	2.263929
H	3.369966	-2.497436	2.434327
H	1.682555	-2.529315	0.637644
H	-3.268161	1.869529	-1.837072
H	-1.532533	2.150737	-1.871563
H	-2.176360	0.566206	-2.382189
H	-5.409252	1.290240	0.093452
H	-6.098347	-0.075656	0.962147
H	-5.047799	1.072386	1.830893
H	-4.413805	-0.301462	-1.643167
H	-3.347225	-1.632889	-1.087664
H	-5.086197	-1.649928	-0.731511
H	-3.394154	2.543616	0.594175
H	-2.385651	1.681199	1.795424
H	-1.644470	2.768594	0.603381

1 imaginary frequency
E = -1209.955583
G = -1209.553966
E_{soln} = -1210.285629
G_{soln} = -1209.884013

TS-35

C	-2.014373	1.081079	-0.307227
C	-0.978156	0.152186	-0.132011
C	-1.301156	-1.178596	0.180739
C	-2.625714	-1.579021	0.290192

C	-3.648298	-0.649431	0.098475
C	-3.340582	0.677429	-0.197731
C	0.447406	0.535624	-0.250154
C	0.739866	1.998791	-0.569907
C	1.338684	-0.483336	-0.744619
C	2.603324	-0.520383	-0.182261
C	2.672478	0.277630	0.987431
C	1.443782	0.553111	1.572239
O	3.670101	-1.183598	-0.618833
H	0.998687	-1.212912	-1.476232
H	3.516786	-1.537129	-1.515018
H	1.330841	1.429608	2.206657
H	0.752287	-0.256373	1.784884
H	3.603285	0.739909	1.307135
H	-1.792227	2.115759	-0.544298
H	-4.135910	1.401101	-0.343707
H	-4.685167	-0.958260	0.188867
H	-2.861786	-2.609383	0.535447
H	1.811071	2.183372	-0.640005
H	0.285618	2.203537	-1.546351
H	0.294357	2.674403	0.161663
H	-0.502221	-1.895340	0.360627

1 imaginary frequency
E = -539.846481
G = -539.660010
E_{soln} = -539.993804
G_{soln} = -539.807333

TS-36

C	1.601404	-0.319540	-1.088145
C	1.631905	0.460937	0.083519
C	2.819851	0.521310	0.828096
C	3.937895	-0.202452	0.426155
C	3.891130	-0.978570	-0.729357
C	2.721272	-1.031015	-1.491048
C	0.433917	1.220671	0.480039
C	-0.848437	0.607775	0.275375
N	-1.037067	-0.747860	0.366483
C	-1.849743	-1.450911	-0.532636
O	-1.921607	-2.725652	-0.131751
C	-1.295185	-2.875133	1.159374
C	-0.342361	-1.683148	1.251279
O	-2.371489	-1.007369	-1.519683
H	-0.261139	-1.278736	2.260805
C	-1.942295	1.450734	-0.042464
C	-1.547392	2.690902	-0.567631
C	-0.268983	2.752786	-1.090901
O	-3.176466	1.054183	0.226926
H	-3.831626	1.596725	-0.251470
H	0.241983	3.710703	-1.161298
H	0.130153	1.960987	-1.712626
H	-2.164305	3.576288	-0.431341
C	0.603992	2.282714	1.545841
H	-2.075491	-2.838421	1.922865
H	0.651524	-1.926517	0.862292
H	-0.787794	-3.837517	1.177962
H	2.870539	1.117580	1.732740
H	4.846905	-0.158754	1.017151
H	4.766837	-1.538554	-1.042603
H	2.687590	-1.622481	-2.400134
H	1.411137	2.975822	1.302431
H	-0.319952	2.830772	1.724989
H	0.871502	1.764537	2.475749
H	0.696279	-0.349064	-1.691796

1 imaginary frequency
E = -861.088259
G = -860.839002
E_{soln} = -861.329013

G_{soln} = -861.079756

TS-37

C	-2.996537	-0.442403	-1.295226
C	-2.762941	0.305606	-0.125279
C	-3.275268	1.609751	-0.040254
C	-3.980065	2.158040	-1.106752
C	-4.194781	1.410157	-2.262564
C	-3.706254	0.104913	-2.353530
C	-2.016872	-0.302823	0.988829
C	-0.931967	-1.189627	0.656791
N	-0.130589	-0.975021	-0.439515
C	0.267579	-1.997934	-1.301103
O	1.100087	-1.477709	-2.217158
C	1.451806	-0.120492	-1.878098
C	0.254567	0.330966	-0.985257
O	-0.087536	-3.144579	-1.270064
C	0.516272	1.353743	0.089923
C	0.023305	2.648937	-0.068229
C	0.227853	3.606822	0.924383
C	0.919006	3.268339	2.085260
C	1.411991	1.972773	2.249368
C	1.212125	1.018639	1.257507
C	-0.690099	-2.292424	1.502179
C	-1.791741	-2.615242	2.312758
C	-3.023144	-2.158977	1.882440
O	0.508754	-2.857573	1.520333
H	0.477565	-3.728948	1.958817
H	-3.831424	-2.028446	2.598860
H	-3.324147	-2.222135	0.843937
H	-1.647340	-3.066908	3.291828
C	-2.022490	0.427796	2.315220
C	2.807388	-0.072390	-1.218292
H	-0.540248	0.696371	-1.647784
H	1.454828	0.453195	-2.805561
H	1.593499	0.008688	1.390398
H	1.951543	1.705283	3.152713
H	1.072499	4.010415	2.862865
H	-0.159762	4.612293	0.792447
H	-0.533498	2.904809	-0.966666
H	-3.104903	2.208028	0.848691
H	-4.361467	3.171356	-1.034324
H	-4.748034	1.840046	-3.091793
H	-3.885497	-0.484681	-3.246634
H	-3.036779	0.638173	2.659393
H	-1.471482	-0.119309	3.079537
H	-1.510043	1.386513	2.152521
C	3.516816	1.130554	-1.239895
C	4.734856	1.239647	-0.576479
C	5.256813	0.141917	0.106905
C	4.558688	-1.063135	0.117924
C	3.335970	-1.172960	-0.543102
H	3.106151	1.986834	-1.770029
H	5.278539	2.179123	-0.596542
H	6.208503	0.224477	0.622943
H	4.966100	-1.923926	0.639382
H	2.806854	-2.121783	-0.534186
H	-2.635035	-1.466581	-1.361667

1 imaginary frequency
E = -1323.025119
G = -1322.625184
E_{soln} = -1323.379282
G_{soln} = -1322.979348

Synthesis of Compounds

General Experimental

All reactions were performed under an inert atmosphere of anhydrous N_2 in glassware dried with heating under vacuum, unless otherwise stated. CH_2Cl_2, Et_2O, toluene, dimethylsulfoxide (DMSO) and *N*,*N*-dimethylformamide (DMF) were dried using a commercial solvent purification system. Tetrahydrofuran (THF) and 1,4-dioxane (dioxane) were purchased in an anhydrous form and stored under nitrogen. Solvents used in reaction extractions and chromatography and all other reagents were used as supplied by commercial vendors unless otherwise indicated. PS refers to commercial petroleum spirits with a boiling point range of 40-60 °C. Column (flash) chromatography was performed on silica gel (40-63 µm). Analytical TLC was performed using aluminium backed 0.2 mm thick silica gel 60 GF254 plates. The TLC plates were visualised using a 254 nm UV lamp, and/or stained using reagent solutions of *p*-anisaldehyde (EtOH (214 mL), H_2SO_4 (8 mL), AcOH (2.4 mL), *p*-anisaldehyde (5.9 mL)), $KMnO_4$ ($KMnO_4$ (1.5g), K_2CO_3 (10g), and 10% aq. NaOH (1.25mL), H_2O (200 mL)) or phosphomolybdic acid (phosphomolybdic acid:95% ethanol (4g:100mL)) followed by heating. ^{1}H NMR spectra were recorded at 400 MHz, ^{13}C NMR at 101 MHz, for selected compounds the number of attached hydrogens to each carbon atom was determined using Distortionless Enhancement by Polarization Transfer with detection of quaternary carbons (DEPT-Q 135), as indicated. Chemical shifts were calibrated using residual nondeuterated solvent as an internal reference and are reported in parts per million (δ) relative to trimethylsilane (δ = 0). High-resolution mass spectra (HR-ESI) were recorded on a time of flight mass spectrometer fitted with an electrospray (ESI) ion source, the capillary voltage was 2400 V. All data was acquired and mass corrected using a dual-spray Leucine Enkephaline reference sample. Melting points were measured using an electrothermal melting point apparatus. Chiral HPLC was performed using an Agilent infinity 1260 system with a solvent system as indicated on a Phenomonex Lux 5µm Cellulose-2 column (150 x 4.6 mm) and processed using ChemStation for LC systems.

SI-1 (4*S*,5*R*)-4,5-diphenyloxazolidin-2-one

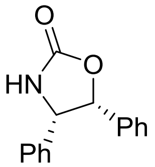

Procedure adapted from that reported by Wagman and Moser.[1] (1*R*,2*S*)-2-amino-1,2-diphenylethan-1-ol (6.0 g, 28 mmol) and K_2CO_3 (0.386 g, 2.4 mmol) were placed in a magnetic stirrer equipped rbf which was evacuated and back filled with N_2. Anhydrous diethylcarbonate (20.4 mL, 168 mmol) was added and the reaction was heated to reflux for two six hour periods (12 h in total), heating for more extended periods resulted in formation of what may be an *N*-ethyl oxazolidinone by product. Water (100 mL) and dichloromethane (200 mL) were added and the organic layer separated, the aq. layer was extracted with dichloromethane (2 x 50 mL). The combined organic layers were washed with brine, dried ($MgSO_4$), filtered and concentrated to yield (4*S*,5*R*)-4,5-diphenyloxazolidin-2-one (6.2 g, 27.7 mmol, 98%) which was used without further purification. Trituration with hot toluene allows removal of non-polar side products when present. ^{1}H NMR in accordance with that previously reported.[2] ^{1}H NMR (400 MHz, $CDCl_3$) δ 7.17 – 7.05 (m, 6H), 6.99 – 6.90 (m, 4H), 5.96 (d, *J* = 8.2 Hz, 1H), 5.64 (s, br, 1H), 5.19 (d, *J* = 8.2 Hz, 1H).

SI-2 Tributyl(cyclohex-1-en-1-yl)stannane

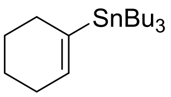

In a dried two necked flask with magnetic stirrer, 1-bromo-cyclohex-1-ene[3] (1.93 g, 12.0 mmol) was dissolved in anhydrous THF (20 mL) and cooled to -78 °C. *t*-BuLi (1.4 M in pentane, 17.1 mL, 24 mmol) was added dropwise over 10 minutes, the reaction was stirred for 1 h at -78 °C, warmed to 0 °C over 0.5 h, cooled to -78 °C and Bu_3SnCl (3.29 mL, 3.95 g, 12.12 mmol) was added dropwise resulting in formation of a white precipitate. The reaction was let warm to room temperature and sat. aq. K_2CO_3 (10 mL) was added followed by Et_2O (20 mL), the organic layer was separated and the aqueous layer extracted with Et_2O (2 x 20 mL). The combined organic layers were washed sat. aq. K_2CO_3 (2 x 20 mL), dried ($MgSO_4$), filtered and concentrated to give tributyl(cyclohex-1-en-1-yl)stannane **SI-2** (4.59 g, quantitative) as a colourless oil which was in accordance with previously reported data[4] and used without further purification.

^{1}H NMR (400 MHz, $CDCl_3$) δ 5.79 (m, $J_{Sn\text{-}H}$ = 68.6 Hz, 1H), 2.20 – 2.10 (m, 2H), 2.09 – 1.99 (m, 2H), 1.66 – 1.57 (m, 4H), 1.53 – 1.42 (m, 6H), 1.37 – 1.25 (m, 6H), 0.92 – 0.81 (m, 15H).

<u>General Method A</u>: Formation of ynamide

A mixture of oxazolidinone **SI-1** (1.0 eq.), ground K_2CO_3 (2.0 eq.), ground $CuSO_4.H_2O$ (0.1 Eq.), 1,10-phenanthroline (0.2 Eq.) in a dried two necked flask fitted with a condenser and magnetic stir bar were placed under vacuum and backfilled with N_2. Bromoalkyne (1.2-1.3 eq.) and anhydrous toluene (0.25 M) were added and heated at 90 °C until ^{1}H NMR indicates complete consumption of the oxazolidinone (typically 48 h). After this time the reaction was cooled to rt, diluted with EtOAc, filtered through celite and purified as indicated below.

4a (4S,5*R*)-4,5-diphenyl-3-(phenylethynyl)oxazolidin-2-one

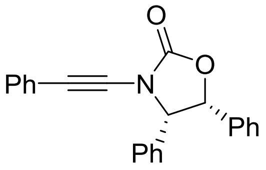

Prepared from **SI-1** (1.2 g, 5.01 mmol) and (bromoethynyl)benzene[5] (1.19 g, 6.00 mmol) following General Method A. The filtered organic layer was washed with 10% aq. citric acid, followed by water and brine, dried ($MgSO_4$), filtered and concentrated. The resulting brown solid was triturated with hot cyclohexane and filtered to yield **4a** (1.31 g, 3.85 mmol, 77%) as a brown solid of sufficient purity for use in subsequent steps. R_f = 0.46 (1:4 EtOAc:PS); MP = 158.2-162.3°C; ^{1}H NMR (400 MHz, $CDCl_3$) δ 7.31 – 7.27 (m, 2H), 7.25 – 7.20 (m, 3H), 7.14 (ddd, *J* = 9.7, 5.2, 2.1 Hz, 6H), 6.98 – 6.93 (m, 4H), 5.99 (d, *J* = 8.1 Hz, 1H), 5.44 (d, *J* = 8.1 Hz, 1H); ^{13}C NMR (101 MHz, $CDCl_3$) δ 155.9, 133.5, 132.8, 131.7, 128.8, 128.6, 128.4, 128.3, 128.24, 128.21, 127.6, 126.3, 122.2, 81.1, 78.6, 72.5, 67.4; HRMS calcd. for $C_{23}H_{18}NO_2$ $(M+H)^+$: 340.1332, Found: 340.1335.

4b (4*S*,5*R*)-3-(hept-1-yn-1-yl)-4,5-diphenyloxazolidin-2-one

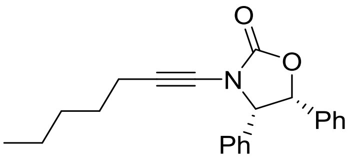

Prepared from (4S,5*R*)-4,5-diphenyloxazolidin-2-one **SI-1** (1 g, 4.184 mmol) and 1-bromohept-1-yne (0.946 g, 5.439 mmol) following General Method A.[6] The filtered organic layer was concentrated and purified by flash chromatography (2:48:48, Et_2O:CH_2Cl_2:PS) to give (4*S*,5*R*)-3-(hept-1-yn-1-yl)-4,5-diphenyloxazolidin-2-one **4b** (1.142 g, 3.425 mmol, 82%) as a thick yellow oil. ^{1}H NMR (400 MHz, $CDCl_3$) δ 7.18 – 7.05 (m, 6H), 6.95 – 6.85 (m, 4H), 5.91 (d, *J* = 8.2 Hz, 1H), 5.29 (d, *J* = 8.2 Hz, 1H), 2.18 (t, *J* = 7.0 Hz, 2H), 1.42 – 1.31 (m, 2H), 1.20 – 1.10 (m, 4H), 0.79 (t, *J* = 6.9 Hz, 3H); ^{13}C NMR (101 MHz, $CDCl_3$) δ 156.7, 133.6, 133.0, 128.6, 128.4, 128.2, 128.1, 127.6, 126.2, 80.7, 72.6, 69.6, 67.2, 30.8, 28.3, 22.1, 18.4, 14.0; HRMS calcd for $C_{22}H_{24}NO_2^+$: 334.1802. Found: 334.1804.

10a (*4S,5R*)-3-((*E*)-1-iodo-2-phenylprop-1-en-1-yl)-4,5-diphenyloxazolidin-2-one

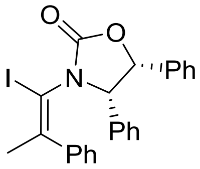

(4*S*,5*R*)-4,5-diphenyl-3-(phenylethynyl)oxazolidin-2-one **4a** (0.6 g, 1.77 mmol) was placed in a dried rbf and evacuated and backfilled with N_2, $CuBr.SMe_2$ (36.4 mg, 0.177 mmol) was added followed by CH_2Cl_2 (5 mL) and then Et_2O (28 mL). The reaction was cooled to -50 °C, MeMgBr (2.9 M in Et_2O, 1.22 mL, 3.54 mmol) was added and the reaction stirred for 0.5 h resulting in a clear brown solution. I_2 (0.99 g, 3.89 mmol) was added, the reaction was let warm to rt and stirred overnight. Sat. aq. NH_4Cl (5 mL) was added followed by 10% aq. $Na_2S_2O_3$ (5 mL), the organic layer was separated and the aq. Layer extracted with Et_2O (2x20 mL), the combined organic layers were washed with brine, dried ($MgSO_4$), filtered and concentrated to provide a yellow solid. Recrystallisation from hot cyclohexane provides (4*S*,5*R*)-3-((*E*)-1-iodo-2-phenylprop-1-en-1-yl)-4,5-diphenyloxazolidin-2-one **10a** (0.672 g, 1.40 mmol, 79%).

Note: Some peaks missing from NMR due to broadening

R_f = 0.5 (10% EtOAc in PS); 138-140 °C; ^{1}H NMR (400 MHz, $CDCl_3$) δ 7.36 (br s, 3H), 7.26 – 7.24 (m, 1H), 7.05 (t, *J* = 7.3 Hz, 2H), MP = 6.94 (br s, 2H), 6.79 (br s, 2H), 6.63 (br s, 2H), 6.12 (br s, 2H), 5.64 (br s, 1H), 5.19 (br s, 1H), 2.15 (s, 3H); ^{13}C DEPT-Q NMR (101 MHz, $CDCl_3$) δ 158.3 (C), 149.0 (C), 139.7 (C), 134.4 (C), 131.3 (C), 129.3 (CH), 128.5 (CH), 128.5 (CH), 128.1 (CH), 127.7 (CH), 127.6 (CH), 126.2 (CH), 79.9 (CH), 68.8 (CH), 29.7 (CH_3); HRMS calcd. for $C_{24}H_{21}INO_2$ $(M+H)^+$: 482.0624, found 482.0611.

10b (4*S*,5*R*)-3-((*E*)-1-iodo-2-phenylbut-1-en-1-yl)-4,5-diphenyloxazolidin-2-one

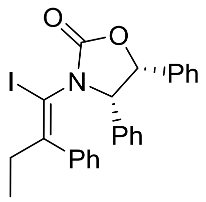

(4*S*,5*R*)-4,5-diphenyl-3-(phenylethynyl)oxazolidin-2-one **4a** (0.2 g, 0.581 mmol) was placed in a dried rbf and evacuated and backfilled with N_2, Rh(acac)(COD) (9.1 mg, 0.0295 mmol) was added followed by THF (6 mL). The reaction was cooled to 0 °C, Et_2Zn (1 M in hexanes, 0.589 mL, 0.589 mmol) was added and the reaction stirred for 0.5 h resulting in a clear red solution. TLC indicates consumption of **4a**. I_2 (0.507 g, 1.178 mmol) was added and the reaction stirred overnight. 10% aq. $Na_2S_2O_3$ (2 mL) was added followed by Et_2O (10 mL), the organic layer was separated and the aq. layer extracted with Et_2O (2x10 mL), the combined organic layers were washed with brine, dried ($MgSO_4$), filtered through a short pad of silica and concentrated to provide a yellow solid. Recrystallisation from hot cyclohexane provides (4*S*,5*R*)-3-((*E*)-1-iodo-2-phenylbut-1-en-1-yl)-4,5-diphenyloxazolidin-2-one **10b** (0.199 g, 0.402 mmol, 68%) as a yellow solid. Note: Some peaks missing from NMR due to broadening. R_f = 0.42 (10% EtOAc in PS); MP = 176-178 °C; ^{1}H NMR (400 MHz, $CDCl_3$) δ 7.36 (br s, 3H), 7.22 (dd, *J* = 6.9, 2.7 Hz, 2H), 7.05 (t, *J* = 7.2 Hz, 2H), 6.94 (br s, 2H), 6.83 (br s, 2H), 6.58 (br s, 2H), 6.17 (br s, 2H), 5.62 (br s, 1H), 5.19 (br s, 1H), 2.55 (br s, 1H), 2.36 (br s, 1H), 0.93 (t, *J* = 7.5 Hz, 3H). ^{13}C NMR DEPT-Q (101 MHz, $CDCl_3$) δ 154.2 (C), 138.5 (C), 134.4 (C), 131.4 (C), 129.4 (CH), 128.5 (CH), 128.2 (CH), 128.1 (CH), 128.0 (CH), 127.8 (CH), 127.6 (CH), 126.3 (CH), 79.9 (CH), 68.4 (CH), 36.1 (CH_2), 11.8 (CH_3). HRMS calcd. for $C_{25}H_{23}INO_2$ $(M+H)^+$: 496.0768, found 496.0771.

General Method B: Formation of divinyl ketones 5 – Carbonylative Stille coupling

In a dried rbf ca. 10 times the volume of the amount of solvent to be used, equipped with a large magnetic stir bar, in line vent adaptor and a stopcock fitted cone to tube adaptor, vinyl iodide **6** (1 eq.) and CuI (0.3 eq.) are added and dissolved in DMF (0.08 - 0.1 M in iodide). Vinyl stannane (2 eq.) and [1,1′-Bis(di-*tert*-butylphosphino)ferrocene]dichloropalladium(II) (5 mol%) were added, the reaction was stirred rapidly, evacuated and backfilled with CO three times. The reaction was stirred at room temperature for 3 days to 1 week until significant consumption of vinyl iodide had occurred (>80%) and prior to alkene isomerisation proceeding beyond 20-35%, then diluted with Et_2O and sat. aq. K_2CO_3. The aqueous layer was separated and extracted with Et_2O twice, the combined organic layers were washed with sat. aq. K_2CO_3, water, brine, dried ($MgSO_4$), filtered and concentrated. The crude residue was purified by column chromatography using silica mixed with 10% powdered K_2CO_3 by weight to remove residual tin compounds.

General Method C: Formation of dienyl ketones 5 – Carbomagnesiation and addition to aldehydes

Ynamide **4** (1 eq.) was placed in a dried rbf equipped with a magnetic stir bar, evacuated and backfilled with N_2 and dissolved in anhydrous CH_2Cl_2 (0.6 M in ynamide), $CuBr.SMe_2$ (0.1 eq.) was added followed

by anhydrous Et_2O (0.1M in ynamide). The reaction was cooled to -40 °C and the Grignard reagent (2 eq.) was added, usually resulting in formation of a brown suspension. The reaction was let warm to the indicated temperature and stirred for the indicated time, resulting in a clear brown solution and consumption of the alkyne. The aldehyde (2.2 eq) was added at -78 °C, let warm to rt and stirred for 2 h. Sat. aq. NH_4Cl was added followed by Et_2O, the organic layer was separated, aq. layer extracted twice with Et_2O, the organic layers combined, washed with brine, dried ($MgSO_4$), filtered and concentrated to provide the carbinol as a yellow oil which was analysed by ^1H NMR. Based on the level of aldehyde conversion as judged by ^1H NMR, Dess-Martin periodinane (1.1-2 eq.) was added to a solution of the crude carbinol in CH_2Cl_2 and stirred at rt for 1 h or until TLC indicates consumption of the carbinol. 10% aq. $Na_2S_2O_3$ was added followed by 1M aq. K_2CO_3 and the reaction stirred for 10 min. CH_2Cl_2 and H_2O were added and the organic layer separated, the aq. layer was extracted twice with CH_2Cl_2, combined organic layers were washed with brine, dried ($MgSO_4$), filtered and concentrated. Column chromatography provided the dienylketone.

General Method D: Removal of oxazolidinone auxiliary

A ~1 M solution of lithium naphthalenide was prepared by addition of finely cut lithium metal to a slight excess of naphthalene in THF and stirring at rt for 3 hours or until no more metal was visible, resulting in a dark green solution. ~2 eq. of this solution was added to the cyclopentanoid **7** (1 eq.) in THF (0.1 M) at -78 °C until the dark colour persisted, the reaction was immediately quenched at -78 °C with sat. aq. NH_4Cl. The reaction was diluted with Et_2O and H_2O, warmed to rt, the organic layer was separated and the aqueous layer extracted with Et_2O. The combined organic layers were washed with brine, dried ($MgSO_4$), filtered and concentrated. The residue was purified by column chromatography to give the desired cyclopentanone.

5a (4*S*,5*R*)-3-((*Z*)-1-(cyclohex-1-en-1-yl)-1-oxo-3-phenylbut-2-en-2-yl)-4,5-diphenyloxazolidin-2-one

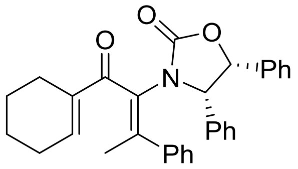

Prepared by General Method B from **10a** and **SI-2** over 3 days. Purified by column chromatography (crude residue dissolved in toluene and PS, 2.5% step gradient, 5% - 10% EtOAc in PS).To give **5a** (0.240 g, 0.518 mmol, 55%) and (4*S*,5*R*)-3-((*E*)-1-(cyclohex-1-en-1-yl)-1-oxo-3-phenylbut-2-en-2-yl)-4,5-diphenyloxazolidin-2-one **5a'** (41 mg, 0.088 mmol, 10%) as clear oils.

5a R_f = 0.2 (10% EtOAc in PS); ^1H NMR (400 MHz, $CDCl_3$) δ 7.43 – 7.37 (m, 3H), 7.25 – 7.21 (m, 2H), 7.21 – 7.17 (m, 1H), 7.04 – 6.98 (m, 4H), 6.92 (t, *J* = 7.5 Hz, 2H), 6.82 – 6.77 (m, 2H), 6.66 – 6.62 (m, 2H), 5.54 (d, *J* = 8.4 Hz, 1H), 4.74 (d, *J* = 8.4 Hz, 1H), 2.29 – 2.23 (m, 2H), 2.21 – 2.16 (m, 2H), 1.86 (s, 3H), 1.66 – 1.59 (m, 4H); ^{13}C DEPT-Q NMR (101 MHz, $CDCl_3$) δ 195.3 (C), 156.7 (C), 145.1 (C), 140.2 (C), 139.6 (C), 139.5 (C), 134.7 (C), 133.5 (C), 128.8 (CH), 128.6 (CH), 128.4 (CH), 128.1 (CH), 128.0 (C), 127.9 (CH), 127.8 (CH), 127.8 (CH), 127.5 (CH), 126.1 (CH), 80.4 (CH), 66.2 (CH), 26.6 (CH_2), 22.8 (CH_2), 22.1 (CH_3), 22.0 (CH_2), 21.6 (CH_2); HRMS calcd. for $C_{31}H_{30}NO_3$ $(M+H)^+$: 464.2220, found 464.2188;

5a' (4*S*,5*R*)-3-((*E*)-1-(cyclohex-1-en-1-yl)-1-oxo-3-phenylbut-2-en-2-yl)-4,5-diphenyloxazolidin-2-one

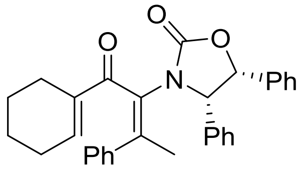

R_f = 0.16 (10% EtOAc in PS); ^{1}H NMR (400 MHz, $CDCl_3$) δ 7.21 – 7.13 (m, 6H), 7.09 – 6.98 (m, 5H), 6.98 – 6.94 (m, 2H), 6.86 (dd, *J* = 7.9, 1.4 Hz, 2H), 5.89 (d, *J* = 8.7 Hz, 1H), 5.59 (s, 1H), 5.47 (d, *J* = 8.7 Hz, 1H), 2.27 (s, 3H), 2.06 – 1.95 (m, 1H), 1.56 – 1.50 (m, 1H), 1.49 – 1.43 (m, 2H), 1.24 – 1.16 (m, 1H), 1.16 – 1.05 (m, 1H), 1.05 – 0.91 (m, 2H). ^{13}C DEPT-Q NMR (101 MHz, $CDCl_3$) δ 195.3 (C), 156.0 (C), 145.0 (C), 142.7 (CH), 141.5 (C), 139.3 (C), 135.5 (C), 134.5 (C), 129.3 (C), 128.9 (CH), 128.5 (CH), 128.4 (CH), 128.2 (CH), 128.1 (CH), 128.0 (CH), 126.2 (CH), 79.6 (CH), 65.0 (CH), 25.8 (CH_2), 23.1 (CH_2), 21.8 (CH_3), 21.6 (CH_2), 21.3 (CH_2); HRMS calcd. for $C_{31}H_{30}NO_3$ $(M+H)^+$: 464.2220, found 464.2221;

5b (4*S*,5*R*)-3-((*Z*)-1-(cyclohex-1-en-1-yl)-1-oxo-3-phenylpent-2-en-2-yl)-4,5-diphenyloxazolidin-2-one

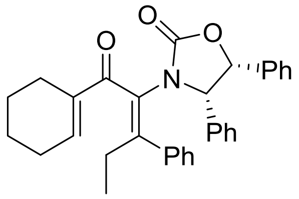

Prepared by General Method B from **10b** and tributyl(cyclohex-1-en-1-yl)stannane **SI-2** over 7 days. Purified by column chromatography (crude residue dissolved in toluene and PS, 2.5% step gradient, 5% - 10% EtOAc in PS).To give **5b** (93 mg, 0.195 mmol, 52%) as a white solid and (4*S*,5*R*)-3-((*E*)-1-(cyclohex-1-en-1-yl)-1-oxo-3-phenylpent-2-en-2-yl)-4,5-diphenyloxazolidin-2-one **5b'** (13 mg, 0.0274 mmol, 7%) as a clear oil.

5b (4*S*,5*R*)-3-((*Z*)-1-(cyclohex-1-en-1-yl)-1-oxo-3-phenylpent-2-en-2-yl)-4,5-diphenyloxazolidin-2-one

R_f = 0.26 (10% EtOAc in PS); MP = 150-152 °C; ^{1}H NMR (400 MHz, $CDCl_3$) δ 7.44 – 7.37 (m, 3H), 7.25 – 7.22 (m, 1H), 7.20 – 7.16 (m, 2H), 7.04 – 6.98 (m, 4H), 6.93 (t, *J* = 7.5 Hz, 2H), 6.81 – 6.77 (m, 2H), 6.69 – 6.65 (m, 2H), 5.49 (d, *J* = 8.3 Hz, 1H), 4.68 (d, *J* = 8.3 Hz, 1H), 2.32 – 2.09 (m, 6H), 1.67 – 1.60 (m, 4H), 0.73 (t, *J* = 7.3 Hz, 3H); ^{13}C DEPT-Q NMR (101 MHz, $CDCl_3$) δ 195.6 (C), 156.6 (C), 145.0 (C), 144.9 (CH), 139.9 (C), 138.2 (C), 134.8 (C), 133.6 (C), 128.9 (C), 128.5 (CH), 128.37 (CH), 128.35 (CH), 128.1 (CH), 128.0 (C), 127.9 (CH), 127.8 (CH), 127.6 (CH), 126.2 (CH), 80.4 (CH), 66.2 (CH), 27.9 (CH_2), 26.3 (CH_2), 22.9 (CH_2), 22.0 (CH_2), 21.7 (CH_2), 12.1 (CH_3); HRMS calcd. for $C_{32}H_{32}NO_3$ $(M+H)^+$: 478.2377, found 478.2370.

5b' (4*S*,5*R*)-3-((*E*)-1-(cyclohex-1-en-1-yl)-1-oxo-3-phenylpent-2-en-2-yl)-4,5-diphenyloxazolidin-2-one

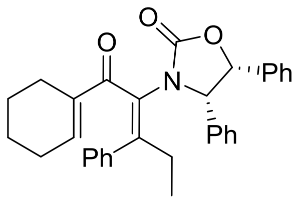

R_f = 0.21 (10% EtOAc in PS); ^{1}H NMR (400 MHz, $CDCl_3$) δ 7.21 – 7.13 (m, 6H), 7.10 – 7.00 (m, 5H), 6.96 – 6.88 (m, 4H), 5.91 (d, *J* = 8.7 Hz, 1H), 5.77 (s, 1H), 5.41 (d, *J* = 8.7 Hz, 1H), 2.78 (dq, *J* = 14.7, 7.4 Hz, 1H), 2.51 (dq, *J* = 15.0, 7.5 Hz, 1H), 1.98 – 1.87 (m, 1H), 1.61 (s, 1H), 1.30 – 1.01 (m, 6H), 0.82 (t, *J* = 7.4 Hz, 3H). ^{13}C DEPT-Q NMR (101 MHz, $CDCl_3$) δ 196.0 (C), 156.7 (C), 150.5 (C), 143.2 (CH), 139.5 (C), 139.2 (C), 135.4 (C), 134.7 (C), 128.9 (CH) 128.5 (CH), 128.42 (CH), 128.36 (CH), 128.2 (CH), 128.1 (CH), 128.03 (CH), 128.01 (s), 127.9 (CH), 126.1 (CH), 79.5 (CH), 65.4 (CH), 27.2 (CH_2), 25.8 (CH_2), 23.0 (CH_2), 21.6 (CH_2), 21.3 (CH_2), 11.7 (CH_3); HRMS calcd. for $C_{32}H_{32}NO_3$ $(M+H)^+$: 478.2377, found 478.2388.

5c (4*S*,5*R*)-3-((*Z*)-1-(3,4-dihydronaphthalen-2-yl)-1-oxo-3-phenylbut-2-en-2-yl)-4,5diphenyloxazolidin-2-one

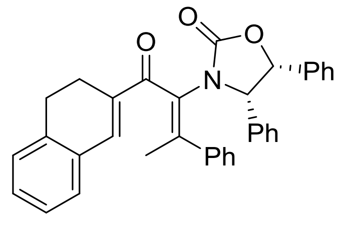

Prepared by General Method C from **4a**, MeMgBr (2.9 M in Et_2O), warmed to -30 °C for 0.5 h and 3,4-dihydronaphthalene-2-carbaldehyde.[7]

Purified by column chromatography (crude residue dissolved in toluene, 2.5% step gradient, 7.5% to 25% EtOAc in PS) to give **5c** (541 mg, 1.057 mmol, 72%) as an off white solid.

R_f = 0.43 (1:4 EtOAc:PS); MP = 186-188 °C; ^{1}H NMR (400 MHz, $CDCl_3$) δ 7.62 (s, 1H), 7.49 – 7.40 (m, 3H), 7.34 – 7.28 (m, 2H), 7.28 – 7.23 (m, 1H), 7.23 – 7.15 (m, 3H), 7.08 – 6.98 (m, 4H), 6.92 (t, *J* = 7.6 Hz, 2H), 6.83 – 6.78 (m, 2H), 6.66 (d, *J* = 7.3 Hz, 2H), 5.57 (d, *J* = 8.4 Hz, 1H), 4.79 (d, *J* = 8.4 Hz, 1H), 2.88 (td, *J* = 8.1, 1.2 Hz, 2H), 2.67 – 2.52 (m, 2H), 1.94 (s, 3H); ^{13}C DEPT-Q NMR (101 MHz, $CDCl_3$) δ 194.5 (C), 156.9 (C), 140.5 (CH), 140.2 (C), 140.1 (C), 138.2 (C), 137.7 (C), 134.7 (C), 133.4 (C), 132.8 (C), 130.2 (CH), 129.5 (CH), 128.9 (CH), 128.7 (CH), 128.5 (CH), 128.2 (CH), 127.9 (CH), 127.9 (C), 127.8 (CH), 127.6 (CH), 126.9 (CH), 126.2 (CH), 80.4 (CH), 66.2 (CH), 27.5 (CH_2), 22.3 (CH_3), 21.3 (CH_2); HRMS calcd. for $C_{35}H_{30}NO_3$ $(M+H)^+$: 512.2220, found 512.2220.

5d (4*S*,5*R*)-3-((*Z*)-1-(3,4-dihydronaphthalen-2-yl)-4-methyl-1-oxo-3-phenylpent-2-en-2-yl)-4,5-diphenyloxazolidin-2-one

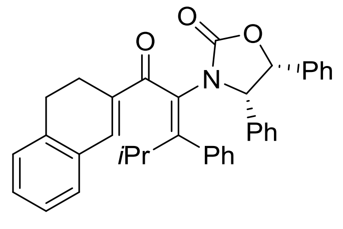

Prepared by General Method C from **4a**, *i*PrMgCl (1.6 M in THF), warmed to -30 °C for 0.5 h, and 3,4-dihydronaphthalene-2-carbaldehyde.[7]

Purified by column chromatography (crude residue dissolved in toluene and PS, 2.5% step gradient, 10-15% EtOAc in PS) to give **5d** (125 mg, 0.232 mmol, 39%) as a yellow solid.

R_f = 0.28 (15% EtOAc in PS); MP = 197-199 °C; ^{1}H NMR (400 MHz, $CDCl_3$) δ 7.81 (s, 1H), 7.45 (t, *J* = 6.3 Hz, 3H), 7.37 – 7.15 (m, 7H), 7.09 – 6.91 (m, 7H), 6.81 – 6.66 (m, 5H), 5.33 (d, *J* = 8.0 Hz, 1H), 4.76 (d, *J* = 8.0 Hz, 1H), 2.90 – 2.79 (m, 3H), 2.61 – 2.48 (m, 1H), 2.41 – 2.29 (m, 1H), 0.93 (d, *J* = 6.8 Hz, 3H), 0.90 (d, *J* = 6.8 Hz, 3H); ^{13}C DEPT-Q NMR (101 MHz, $CDCl_3$) δ 194.6 (C), 156.3 (C), 150.9 (C), 141.0 (CH), 137.9

(C), 137.7 (C), 135.5 (C), 134.3 (C), 134.0 (C), 132.6 (C), 130.2 (CH), 129.6 (CH), 129.1 (CH), 128.7 (CH), 128.2 (C), 128.2 (CH), 127.9 (CH), 127.8 (CH), 127.7 (CH), 127.6 (CH), 126.9 (CH), 126.0 (CH), 80.2 (CH), 66.7 (CH), 31.9 (CH), 27.4 (CH_2), 21.3 (CH_3), 20.9 (CH_2), 20.3 (CH_3); HRMS calcd. for $C_{37}H_{34}NO_3$ $(M+H)^+$: 540.2533, found 540.2540.

5e (4*S*,5*R*)-3-((*Z*)-1-(3,4-dihydronaphthalen-2-yl)-3-methyl-1-oxooct-2-en-2-yl)-4,5diphenyloxazolidin-2-one

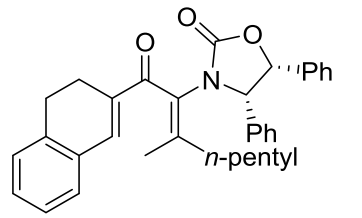

Prepared by General Method C from (4*S*,5*R*)-3-(hept-1-yn-1-yl)-4,5-diphenyloxazolidin-2-one **5b**, MeMgBr (2.9 M in Et_2O), warmed to 0 °C for 1 h, and 3,4-dihydronaphthalene-2-carbaldehyde.[7]

Purified by column chromatography (crude residue dissolved in toluene and PS, 2.5% step gradient, 10-15% EtOAc in PS) to give **5e** (86 mg, 0.17 mmol, 31%) as a thick yellow gum.

R_f = 0.3 (15% EtOAc in PS); ^{1}H NMR (400 MHz, $CDCl_3$) δ 7.24 (t, *J* = 7.5 Hz, 1H), 7.18 – 7.06 (m, 7H), 7.04 – 6.96 (m, 4H), 6.91 (d, J = 6.2 Hz, 2H), 6.80 (d, J = 7.2 Hz, 2H), 5.84 (d, J = 8.5 Hz, 1H), 5.17 (d, J = 8.5 Hz, 1H), 2.79 (t, J = 8.2 Hz, 2H), 2.50 – 2.43 (m, 2H), 2.39 – 2.26 (m, 2H), 1.69 (s, 3H), 1.40 – 1.17 (m, 6H), 0.93 (t, J = 6.9 Hz, 3H); ^{13}C DEPT-Q NMR (101 MHz, CDCl3) δ 194.8 (C), 156.5 (C), 144.4 (C), 140.4 (CH), 137.9 (C), 137.6 (C), 135.2 (C), 134.4 (C), 132.5 (C), 130.2 (CH), 129.4 (CH), 128.8 (CH), 128.55 (s), 128.07 (s), 128.0 (CH), 127.8 (CH), 126.8 (CH), 126.2 (C), 126.0 (CH), 79.5 (CH), 65.7 (CH), 34.2 (CH_2), 32.4 (CH_2), 27.5 (CH_2), 26.6 (CH_2), 22.7 (CH_2), 21.2 (CH_2), 19.0 (CH_3), 14.3 (CH_3); HRMS calcd. for $C_{34}H_{36}NO_3$ $(M+H)^+$: 506.2690, found 506.2690.

5f (4*S*,5*R*)-3-((*Z*)-3-(3,4-dihydronaphthalen-2-yl)-1-(3,5-dimethoxyphenyl)-3-oxo-1-phenylprop-1-en-2-yl)-4,5-diphenyloxazolidin-2-one

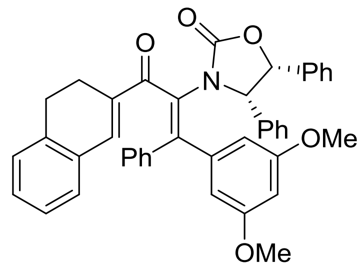

Prepared by General Method C from (4*S*,5*R*)-3-((3,5-dimethoxyphenyl)ethynyl)-4,5-diphenyloxazolidin-2-one,[8] PhMgBr (2.6 M in Et_2O), warmed to 0 °C for 1 h and 3,4-dihydronaphthalene-2-carbaldehyde.[7]

Purified by column chromatography (crude residue dissolved in toluene and PS, 2.5% step gradient, 15-17.5% EtOAc in PS) to give **5f** (0.2651 g, 0.42 mmol, 84%) as a yellow solid.

R_f = 0.2 (15% EtOAc in PS); MP = 119-121 °C; ^{1}H NMR (400 MHz, $CDCl_3$) δ 7.35 (s, 1H), 7.15 – 6.92 (m, 19H), 6.71 (d, *J* = 7.2 Hz, 2H), 6.56 (t, *J* = 2.2 Hz, 1H), 6.18 (s, 2H), 5.71 (d, *J* = 8.7 Hz, 1H), 4.97 (d, *J* = 8.8 Hz, 1H), 3.73 (s, 2H), 2.61 – 2.49 (m, 2H), 2.14 – 2.02 (m, 2H); ^{13}C DEPT-Q NMR (101 MHz, $CDCl_3$) δ 195.4 (C), 1607. (C), 156.6 (C), 143.3 (C), 141.1 (C), 140.2 (C), 139.0 (C), 137.7 (CH), 137.1 (C), 135.3

(C), 133.4 (C), 132.7 (C), 130.3 (CH), 129.7 (C), 129.2 (CH), 128.8 (CH), 128.7 (CH), 128.6 (CH), 128.2 (CH), 128.2 (CH), 128.1 (CH), 127.9 (CH), 127.4 (CH), 127.3 (CH), 126.4 (CH), 126.4 (CH), 108.4 (CH), 101.3 (CH), 81.2 (CH), 64.9 (CH), 55.5 (CH_3), 27.3 (CH_2), 22.8 (CH_2); HRMS calcd. for $C_{42}H_{36}NO_5$ $(M+H)^+$: 634.2588, found 634.2599.

5g (4*S*,5*R*)-3-((*Z*)-1-(3,5-dimethoxyphenyl)-1-oxo-3-phenylbut-2-en-2-yl)-4,5-diphenyloxazolidin-2-one

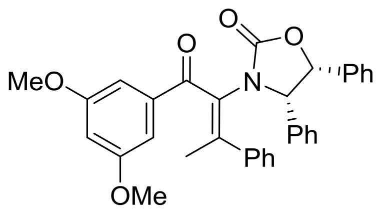

Prepared by General Method C from **4a**, MeMgBr (2.9 M in Et_2O), warmed to -30 °C for 0.5 h and 3,5-dimethoxybenzaldehyde.

Purified by column chromatography (crude residue dissolved in toluene and PS, 2.5% step gradient, 10-15% EtOAc in PS) to give **5g** (0.2775 g, 0.535 mmol, 91%) as a white solid.

R_f = 0.22 (15% EtOAc in PS); MP = 164-166 °C; ^{1}H NMR (400 MHz, $CDCl_3$) δ 7.46 – 7.40 (m, 3H), 7.27 – 7.23 (m, 2H), 7.16 (d, *J* = 2.3 Hz, 2H), 7.06 – 6.99 (m, 4H), 6.91 (t, *J* = 7.6 Hz, 2H), 6.80 (dd, *J* = 7.5, 1.9 Hz, 2H), 6.66 – 6.62 (m, 3H), 5.55 (d, *J* = 8.4 Hz, 1H), 4.70 (d, *J* = 8.4 Hz, 1H), 3.81 (s, 6H), 1.76 (s, 3H); ^{13}C DEPT-Q NMR (101 MHz, $CDCl_3$) δ 193.9 (C), 160.9 (C), 157.0 (C), 141.7 (C), 140.3 (C), 140.1 (C), 134.8 (C), 133.1 (C), 128.9 (C), 128.7 (CH), 128.2 (CH), 127.9 (CH), 127.8 (CH), 127.7 (CH), 127.5 (CH), 126.2 (CH), 106.9 (CH), 106.2 (CH), 80.6 (CH), 65.8 (CH), 55.6 (CH_3), 22.6 (CH_3); HRMS calcd. for $C_{33}H_{30}NO_5$ $(M+H)^+$: 520.2118, found 520.2120.

5h (4*S*,5*R*)-3-((*Z*)-1-(3,5-dimethoxyphenyl)-4-methyl-1-oxo-3-phenylpent-2-en-2-yl)-4,5-diphenyloxazolidin-2-one

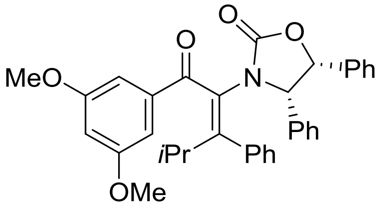

Prepared by General Method C from **4a**, *i*PrMgCl (1.6 M in THF), warmed to -30 °C for 0.5 h, and 3,5-dimethoxybenzaldehyde.

Purified by column chromatography (crude residue dissolved in toluene and PS, 2.5% step gradient, 10-15% EtOAc in PS) to give **5h** (0.0921 g, 0.1775 mmol, 60%) as a waxy yellow solid.

R_f = 0.18 (10% EtOAc in PS); ^{1}H NMR (400 MHz, $CDCl_3$) δ 7.47 – 7.41 (m, 3H), 7.24 – 7.20 (m, 2H), 7.17 (d, *J* = 2.3 Hz, 2H), 7.05 – 6.95 (m, 4H), 6.92 (t, *J* = 7.6 Hz, 2H), 6.73 – 6.63 (m, 5H), 5.32 (d, *J* = 8.2 Hz, 1H), 4.64 (d, *J* = 8.2 Hz, 1H), 3.82 (s, 6H), 2.77 – 2.65 (m, 1H), 0.85 (d, *J* = 6.9 Hz, 3H), 0.76 (d, *J* = 6.7 Hz, 3H). ^{13}C DEPT-Q NMR (101 MHz, $CDCl_3$) δ 194.0 (C), 160.8 (C), 156.5 (C), 151.9 (C), 139.6 (C), 135.6 (C), 134.5 (C), 133.5 (C), 129.2 (CH), 128.8 (CH), 128.4 (C), 128.2 (CH), 128.0 (CH), 128.0 (CH), 127.9 (CH),

127.8 (CH), 127.6 (CH), 126.1 (CH), 107.0 (CH), 106.7 (CH), 80.4 (CH), 66.4 (CH), 55.7 (CH_3), 31.8 (CH), 21.5 (CH_3), 20.4 (CH_3); HRMS calcd. for $C_{35}H_{34}NO_5$ $(M+H)^+$: 548.2431, found 548.2437.

5i (4*S*,5*R*)-3-((*Z*)-1-oxo-3-phenyl-1-(thiophen-2-yl)but-2-en-2-yl)-4,5-diphenyloxazolidin-2-one

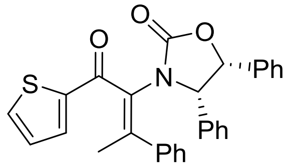

Prepared by General Method C from **4a**, MeMgBr (2.9 M in Et_2O), warmed to -30 °C for 0.5 h and thiophene-2-carbaldehyde.

Purified by column chromatography (crude residue dissolved in toluene and PS, 5% step gradient, 10-20% EtOAc in PS) to give **5i** (0.233 g, 0.5 mmol, 85%) as a yellow solid.

R_f = 0.43 (1:4 EtOAc:PS); MP = 170-172 °C; ^1H NMR (400 MHz, $CDCl_3$) δ 7.94 (dd, *J* = 3.8, 1.1 Hz, 1H), 7.69 (dd, *J* = 4.9, 1.1 Hz, 1H), 7.44 (dd, *J* = 5.0, 1.8 Hz, 3H), 7.27 – 7.23 (m, 2H), 7.14 (dd, *J* = 4.9, 3.8 Hz, 1H), 7.06 – 6.97 (m, 4H), 6.91 (t, *J* = 7.6 Hz, 2H), 6.80 (dd, *J* = 7.4, 2.1 Hz, 2H), 6.65 (dd, *J* = 8.1, 0.9 Hz, 2H), 5.55 (d, *J* = 8.5 Hz, 1H), 4.71 (d, *J* = 8.5 Hz, 1H), 1.86 (s, 3H); ^{13}C DEPT-Q NMR (101 MHz, $CDCl_3$) δ 185.9 (C), 156.8 (C), 144.6 (C), 140.5 (C), 139.8 (C), 134.9 (C), 134.9 (C), 134.7 (C), 133.0 (C), 128.8 (CH), 128.8 (CH), 128.7 (CH), 128.5 (CH), 128.3 (CH), 128.2 (CH), 128.0 (CH), 127.8 (CH), 127.8 (CH), 127.5 (CH), 80.6 (CH), 65.8 (CH), 22.6 (CH_3); HRMS calcd. for $C_{29}H_{24}NO_3S$ $(M+H)^+$: 466.1471, found 466.1471.

5j (4*S*,5*R*)-3-((*Z*)-1-(4-methoxyphenyl)-1-oxo-3-phenylbut-2-en-2-yl)-4,5-diphenyloxazolidin-2-one

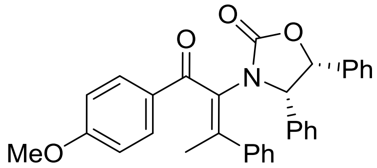

Prepared by General Method C from **4a**, MeMgBr (2.9 M in Et_2O), warmed to -30 °C for 0.5 h and 4-methoxybenzaldehyde.

Purified by column chromatography (crude residue dissolved in toluene and PS, 5% step gradient, 10-20% EtOAc in PS) to give **5j** (0.1315 g, 0.269 mmol, 65%) as a yellow solid.

R_f = 0.34 (1:4 EtOAc:PS); MP = 179-181 °C; 1H NMR (400 MHz, $CDCl_3$) δ 8.02 (d, *J* = 8.9 Hz, 2H), 7.43 (dd, *J* = 5.0, 1.8 Hz, 3H), 7.24 (d, *J* = 2.2 Hz, 2H), 7.06 – 6.98 (m, 4H), 6.93 (d, *J* = 8.9 Hz, 2H), 6.90 (d, J = 7.4 Hz, 2H), 6.80 (dd, *J* = 7.2, 2.0 Hz, 2H), 6.66 – 6.63 (m, 2H), 5.55 (d, *J* = 8.5 Hz, 1H), 4.73 (d, *J* = 8.5 Hz, 1H), 3.88 (s, 3H), 1.72 (s, 3H); ^{13}C DEPT-Q NMR (101 MHz, $CDCl_3$) δ 192.7 (C), 163.8 (C), 157.0 (C), 140.2 (C), 140.0 (C), 134.9 (C), 133.2 (C), 132.0 (CH), 131.3 (C), 128.9 (CH), 128.7 (CH), 128.6 (CH), 128.1 (CH), 128.0 (CH), 127.9 (CH), 127.9 (CH), 127.8 (CH), 127.5 (CH), 126.2 (CH), 114.0 (CH), 80.7 (CH), 65.8 (CH), 55.6 (CH_3), 22.5 (CH_3); HRMS calcd. for $C_{32}H_{28}NO_4$ $(M+H)^+$: 490.2013, found 490.2004.

7a (4*S*,5*R*)-3-((1*S*)-1-methyl-3-oxo-1-phenyl-2,3,4,5,6,7-hexahydro-1*H*-inden-2-yl)-4,5-diphenyloxazolidin-2-one

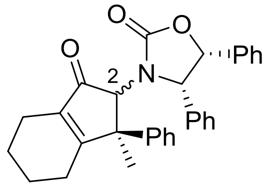

In a dried rbf with magnetic stir bar **5a** (0.085 g, 0.18 mmol) was dissolved in anhydrous CH_2Cl_2 (0.9 mL) and cooled to 0 °C. BF_3.THF (0.06 mL, 0.077 g, 0.55 mmol) was added and the reaction stirred for 0.25 h at which point TLC analysis indicated consumption of starting material. Sat. aq. $NaHCO_3$ (1 mL), H_2O (3 mL) and CH_2Cl_2 (5 mL) were added and the organic layer separated. The aq. layer was extracted with CH_2Cl_2 (2 x 5 mL) and the combined organic extracts were washed with brine, dried ($MgSO_4$), filtered and concentrated. The residue was dissolved in minimal toluene and purified by column chromatography (step gradient, 12.5-20% EtOAc in PS) to give **7a** (0.0570 g, 0.123 mmol, 68%) as a 5:1 mixture of *cis*(2*S*):*trans*(2*R*) isomers (α-C).

R_f = 0.25 (15% EtOAc in PS); ^{1}H NMR (400 MHz, $CDCl_3$) δ 7.50 – 7.34 (m, 5.4H), 7.13 – 7.05 (m, 3.8H), 7.05 – 6.93 (m, 5H), 6.94 – 6.86 (m, 0.8H) 6.78 – 6.73 (m, 2.6H), 6.57 (d, *J* = 7.2 Hz, 0.4H) *trans*, 5.87 (d, *J* = 8.4 Hz, 0.2H) *trans*, 5.25 (d, *J* = 8.5 Hz, 0.2H) *trans*, 4.83 (d, *J* = 8.5 Hz, 1H), 4.57 (d, *J* = 8.5 Hz, 1H), 3.52 (s, 0.2H) *trans*, 3.36 (s, 1H), 2.52 – 2.44 (m, 1.2H), 2.37 – 2.30 (m, 1.2H), 2.19 – 2.13 (m, 2H), 2.12 – 1.98 (m, 0.4H) *trans*, 1.89 – 1.82 (m, 1.2H), 1.80 – 1.73 (m, 2.6H), 1.68 – 1.62 (m, 1.8H), 1.59 (s, 3H); ^{13}C DEPT-Q NMR (101 MHz, $CDCl_3$) δ 199.6 (C), 175.1 (C), 155.7 (C), 139.5 (C), 139.1 (C), 134.5 (C), 133.6 (C), 128.5 (CH), 128.4 (CH), 128.4 (CH), 128.3 (CH), 128.0 (CH), 127.9 (CH), 127.8 (CH), 127.8 (CH), 126.3 (CH), 80.2 (CH), 67.4 (CH), 66.0 (CH), 53.4 (C), 25.3 (CH_3), 24.2 (CH_2), 22.3 (CH_2), 21.7 (CH_2), 20.3 (CH_2); HRMS calcd. for $C_{31}H_{30}NO_3$ $(M+H)^+$: 464.2220, found 464.2213.

7a' (4*S*,5*R*)-3-((1*R*)-1-methyl-3-oxo-1-phenyl-2,3,4,5,6,7-hexahydro-1*H*-inden-2-yl)-4,5-diphenyloxazolidin-2-one

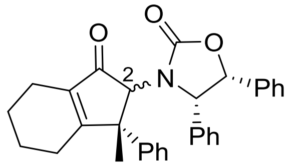

In a dried rbf with magnetic stir bar **5a'** (0.0114 g, 0.0246 mmol) was dissolved in anhydrous CH_2Cl_2 (0.123 mL) and cooled to 0 °C. A CH_2Cl_2 solution of BF_3.THF (0.6 M, 0.123 mL, 0.0738 mmol) was added and the reaction stirred for 2 h at which point sat. aq. $NaHCO_3$ (1 mL), H_2O (3 mL) and CH_2Cl_2 (5 mL) were added and the organic layer separated. The aq. layer was extracted with CH_2Cl_2 (2 x 5 mL) and the combined organic extracts were washed with brine, dried ($MgSO_4$), filtered and concentrated. The residue was dissolved in minimal toluene and purified by column chromatography (20% EtOAc in PS) to give **7a'** (8 mg, 0.0179 mmol, 73%) as a 7:1 mixture of *cis*(2*S*):*trans*(2*R*) isomers (α-C).

R_f = 0.22 (20% EtOAc in PS); ^{1}H NMR (400 MHz, $CDCl_3$) δ 7.54 – 7.31 (m, 5H), 7.27 (m, 0.16H) *trans*, 7.25 (m, 0.15H) *trans*, 7.22 – 7.17 (m, 0.24H) *trans*, 7.10-6.93 (m, 7.4H), 6.67 (br, 2H), 6.56 – 6.49 (m, 2.13H), 5.89 (d, *J* = 8.7 Hz, 0.13H) *trans*, 4.82 (d, *J* = 8.7 Hz, 0.14H) *trans*, 4.55 (s, 1H), 4.37 (d, *J* = 6.9

Hz, 1H), 3.99 (d, *J* = 6.6 Hz, 1H), 3.48 (s, 0.13H) *trans*, 2.37 – 2.09 (m, 4.4H), 1.90 – 1.59 (m, 8H); ^{13}C DEPT-Q NMR (101 MHz, $CDCl_3$) δ 199.6 (C), 175.2 (C), 159.3 (C), 141.1 (C), 138.6 (C), 135.8 (C), 133.9 (C), 128.9 (CH), 128.1 (CH), 127.9 (CH), 127.9 (CH), 127.8 (CH), 127.7 (CH), 127.6 (CH), 127.5 (CH), 126.2 (CH), 81.6 (CH), 69.1 (CH), 65.6 (CH), 54.5 (C), 24.5 (CH_3), 24.2 (CH_2), 22.3 (CH_2), 21.6 (CH_2), 20.2 (CH_2); HRMS calcd. for $C_{31}H_{30}NO_3$ $(M+H)^+$: 464.2220, found 464.2220.

7b (4*S*,5*R*)-3-((1*S*)-1-ethyl-3-oxo-1-phenyl-2,3,4,5,6,7-hexahydro-1*H*-inden-2-yl)-4,5-diphenyloxazolidin-2-one

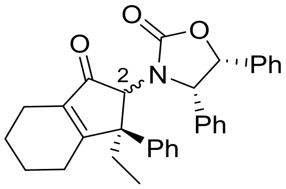

In a dried rbf with magnetic stir bar **5b** (0.0601 g, 0.1258 mmol) was dissolved in anhydrous CH_2Cl_2 (0.63 mL) and cooled to 0 °C. A CH_2Cl_2 solution of BF_3.THF (0.6 M, 0.629 mL, 0.3775 mmol) was added and the reaction stirred for 4 h at which point ^1H NMR analysis indicated completion of the reaction. Sat. aq. $NaHCO_3$ (1 mL), H_2O (3 mL) and CH_2Cl_2 (5 mL) were added and the organic layer separated. The aq. layer was extracted with CH_2Cl_2 (2 x 5 mL) and the combined organic extracts were washed with brine, dried ($MgSO_4$), filtered and concentrated. The residue was dissolved in minimal toluene and purified by column chromatography (5% step gradient, 15-20% EtOAc in PS) to give **7b** (51.7 g, 0.1083 mmol, 86%) as clear oil with a 3.5:1 mixture of *trans*(2*R*):*cis*(2*S*) isomers (α-C).

R_f = 07 (m, 4H), 7.07 – 7.02 (m, 3.9H), 7.02 – 6.98 (m, 1.3H), 6.98 – 6.89 (m, 4.3H), 6.77 – 6.72 (m, 0.6H) *cis*, 6.72 – 6.66 (m, 4H), 5.92 (d, *J* = 8.1 Hz, 1H), 5.02 (d, *J* = 8.1 Hz, 1H), 4.72 (d, *J* = 8.4 Hz, 0.3H) *cis*, 4.50 (d, *J* = 8.4 Hz, 0.3H) *cis*, 3.75 (s, 1H), 3.42 (s, 0.3H) *cis*, 2.50 – 2.10 (m, 6.3H), 2.10 – 1.97 (m, 1.6H), 1.83 – 1.72 (m, 3H), 1.70 – 1.59 (m, 1.3H), 1.59 – 1.48 (m, 1H), 0.97 (t, *J* = 7.5 Hz, 3H), 0.64 (t, *J* = 7.4 Hz, 0.9H) *cis*; ^{13}C DEPT-Q NMR (101 MHz, $CDCl_3$) δ 201.0 (C), 176.0 (C), 158.4 (C), 143.0 (C), 138.4 (C), 135.0 (C), 134.9 (C), 128.5 (CH), 128.4 (CH), 128.3 (CH), 128.2 (CH), 128.0 (CH), 127.9 (CH), 127.9 (CH), 127.8 (CH), 126.8 (CH), 126.3 (CH), 126.2 (CH), 126.1 (CH), 80.2 (CH), 73.5 (CH), 69.5 (CH), 56.7 (CH), 27.0 (CH_2), 24.5 (CH_2), 22.5 (CH_2), 21.5 (CH_2), 20.2 (CH_2), 10.7 (CH_3); HRMS calcd. for $C_{32}H_{32}NO_3$ $(M+H)^+$: 478.2377, found 478.2380.

7b' (4*S*,5*R*)-3-((1*R*,2*S*)-1-ethyl-3-oxo-1-phenyl-2,3,4,5,6,7-hexahydro-1*H*-inden-2-yl)-4,5-diphenyloxazolidin-2-one

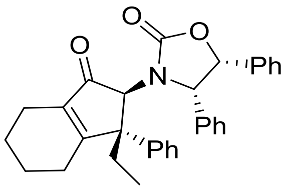

In a dried rbf equipped with a magnetic stir bar **5b'** (0.0303 g, 0.0635 mmol) was dissolved in CH_2Cl_2 (1 mL) and cooled to 0 °C. BF_3.THF (0.02 mL, 0.18 mmol) was added and the reaction stirred for 3 h. Sat. aq. $NaHCO_3$ (1 mL), H_2O (3 mL) and CH_2Cl_2 (5 mL) were added and the organic layer separated. The aq. layer was extracted with CH_2Cl_2 (2 x 5 mL) and the combined organic extracts were washed with brine, dried ($MgSO_4$), filtered and concentrated. The residue was dissolved in minimal toluene

and purified by column chromatography (5% step gradient, 15-20% EtOAc in PS) to give **7b'** (0.0209 g, 0.0438 mmol, 69%)

R_f = 0.22 (20% EtOAc in PS); MP = 203-205 °C; 1H NMR (400 MHz, $CDCl_3$) δ 7.30 (t, *J* = 7.5 Hz, 2H), 7.21 (t, *J* = 7.3 Hz, 1H), 7.11 – 7.02 (m, 5H), 7.00 – 6.89 (m, 5H), 6.80 – 6.65 (br s, 2H), 5.89 (d, *J* = 8.6 Hz, 1H), 4.77 (d, *J* = 8.6 Hz, 1H), 3.44 (s, 1H), 2.55 – 2.38 (m, 2H), 2.34 – 2.20 (m, 2H), 2.16 (dd, *J* = 6.7, 4.9 Hz, 2H), 1.84 – 1.73 (m, 2H), 1.73 – 1.63 (m, 1H), 1.58 – 1.49 (m, 1H), 1.07 (t, *J* = 7.4 Hz, 3H); ^{13}C DEPT-Q NMR (101 MHz, $CDCl_3$) δ 198.1 (C), 173.8 (C), 156.5 (C), 143.6 (C), 139.0 (C), 134.9 (C), 133.4 (C), 129.1 (CH), 128.4 (CH), 128.1 (CH), 128.0 (CH), 128.0 (CH), 126.8 (CH), 126.7 (CH), 126.2 (CH), 80.0 (CH), 71.2 (CH), 66.9 (CH), 56.8 (C), 26.6 (CH_2), 25.7 (CH_2), 22.5 (CH_2), 21.6 (CH_2), 20.4 (CH_2), 10.6 (CH_3); HRMS calcd. for $C_{32}H_{32}NO_3$ $(M+H)^+$: 478.2377, found 478.2382.

7c (4*S*,5*R*)-3-((1*S*,2*S*)-1-methyl-3-oxo-1-phenyl-2,3,4,5-tetrahydro-1*H*-cyclopenta[*a*]naphthalen-2-yl)-4,5-diphenyloxazolidin-2-one

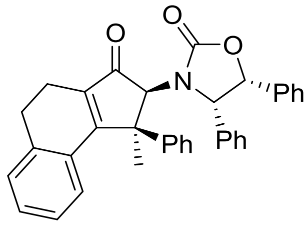

5c (0.1770 g, 0.3463 mmol) was placed in a dried rbf, dissolved in anhydrous CH_2Cl_2 (3.5 mL) and cooled to -78 °C. Neat TfOH (0.05 mL, 0.565 mmol) was added and the reaction was stirred for 2 h then quenched with sat. aq. $NaHCO_3$ (2 mL). CH_2Cl_2 (5 mL) and H_2O (5 mL) were added, the organic layer separated and the aqueous layer extracted with CH_2Cl_2 (2 x 5 mL), the combined organic layers were washed with brine, dried ($MgSO_4$), filtered and concentrated. The residue was dissolved in minimal CH_2Cl_2 and PS and purified by column chromatography (2.5% step gradient, 20-25% EtOAc in PS) to give **7c** (0.1327 g, 0.26 mmol, 75%) as a white solid.

R_f = 0.18 (15% EtOAc in PS); MP = 235-237 °C; ^1H NMR (400 MHz, $CDCl_3$) δ 7.75 (brs, 1H), 7.61 (brs, 1H), 7.43 – 7.27 (m, 3H), 7.26 – 7.20 (m, 2H), 7.17 – 6.93 (m, 9H), 6.82 – 6.75 (m, 3H), 4.82 (d, *J* = 8.5 Hz, 1H), 4.59 (d, *J* = 8.5 Hz, 1H), 3.53 (s, 1H), 3.04 (ddd, *J* = 15.8, 7.2, 5.4 Hz, 1H), 2.91 (ddd, *J* = 15.7, 12.9, 7.2 Hz, 1H), 2.84 – 2.66 (m, 2H), 1.81 (s, 3H); ^{13}C DEPT-Q NMR (101 MHz, $CDCl_3$) δ 198.6 (C), 167.6 (C), 155.7 (C), 140.6 (C), 140.1 (C), 138.0 (C), 134.5 (C), 133.6 (C), 130.5 (CH), 129.8 (C), 129.2 (CH), 128.8 (CH), 128.5 (CH), 128.4 (CH), 128.4 (CH), 128.0 (CH), 128.0 (CH), 127.8 (CH), 127.7 (CH), 126.4 (CH), 126.3 (CH), 80.2 (CH), 69.2 (CH), 66.1 (CH), 52.3 (C), 28.4 (CH_2), 26.8 (CH_3), 18.3 (CH_2); HRMS calcd. for $C_{35}H_{30}NO_3$ $(M+H)^+$: 512.2222, found 512.2232.

(2*R*) *trans* isomer also forms in minor amounts (~8%) but was not observed after chromatography. ^1H NMR (400 MHz, $CDCl_3$) δ 5.83 (d, *J* = 8.5 Hz, 1H), 5.15 (d, *J* = 8.5 Hz, 1H), 3.61 (s, 1H), 1.89 (s, 3H).

7d (4*S*,5*R*)-3-((1*S*)-1-isopropyl-3-oxo-1-phenyl-2,3,4,5-tetrahydro-1*H*-cyclopenta[*a*]naphthalen-2-yl)-4,5-diphenyloxazolidin-2-one

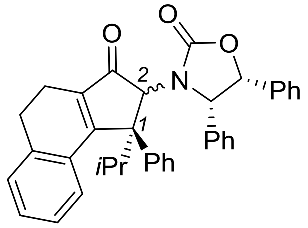

5d (58 mg, 0.107 mmol) was dissolved in 1 mL anhydrous CH_2Cl_2 . BF_3.THF (0.035 mL, 45 mg, 0.32 mmol) was added to the reaction mixture which was heated to reflux overnight. TLC and NMR analysis indicated completion of the reaction and formation of a mixture of diastereomers. 0.5 mL sat. aq. $NaHCO_3$, 2 mL H_2O and 5 mL CH_2Cl_2 were added, the organic layer was separated, aq. layer extracted with CH_2Cl_2 (2 x 5 mL), the organic extracts combined, washed with brine, dried with $MgSO_4$, filtered and concentrated. Column chromatography (step gradient - toluene: PS: EtOAc 50:45:5 to 45:45:10) yielded (1*S*)-**7d** (24 mg, 0.448 mmol, 42% yield) as a 4:1 mixture of *cis*(2*S*):*trans*(2*R*) isomers (α-C). A mixture of this product and a lower R_f product, most likely (1*R*)-**7d** was also isolated in later eluting fractions (14 mg, 24% (2.8:1 *R*:*S* at C1)).

MP = 110-113 °C; ^{1}H NMR (400 MHz, $CDCl_3$) δ 7.64 (br s, 2H), 7.49 (br s, 2H), 7.46 – 7.37 (m, 2H), 7.29 – 7.08 (m, 12H), 7.07 – 6.85 (m, 8H), 6.80 – 6.71 (m, 2H), 5.94 (d, *J* = 8.0 Hz, 0.15H) *trans*, 4.71 (d, *J* = 8.0 Hz, 0.15H) *trans*, 4.66 (d, *J* = 8.3 Hz, 1H) *cis*, 4.38 (d, *J* = 8.4 Hz, 1H) *cis*, 3.81 (s, 0.15H) *trans*, 3.63 (s, 1H) *cis*, 3.04 – 2.96 (m, 1.15H), 2.93 – 2.81 (m, 3.3H)2.72 – 2.58 (m, 1.15H), 1.14 (d, *J* = 6.7 Hz, 0.45H) *trans*, 1.04 (d, *J* = 5.9 Hz, 0.45H) *trans*, 0.97 (d, *J* = 6.2 Hz, 3H), 0.80 (d, *J* = 6.8 Hz, 3H); ^{13}C DEPT-Q NMR (101 MHz, $CDCl_3$) δ 199.7 (C), 165.8 (C), 156.3 (C), 140.2 (C), 139.9 (C), 134.4 (C), 133.6 (C), 131.8 (C), 130.3 (CH), 128.6 (CH), 128.6 (CH), 128.5 (CH), 128.0 (CH), 127.9 (CH), 127.9 (CH), 127.8 (CH), 127.7 (CH), 126.3 (CH), 126.0 (CH), 126.0 (CH), 80.3 (CH), 66.0 (CH), 64.6 (CH), 60.7 (CH), 32.8 (CH), 28.5 (CH_2), 20.6 (CH_3), 20.4 (CH_3), 18.2 (CH_2); HRMS calcd. for $C_{37}H_{33}NO_3$ (M^+): 539.2455, found 539.2415.

(1*R*)-**7d** (4*S*,5*R*)-3-((1*R*)-1-isopropyl-3-oxo-1-phenyl-2,3,4,5-tetrahydro-1*H*-cyclopenta[*a*]naphthalen-2-yl)-4,5-diphenyloxazolidin-2-one

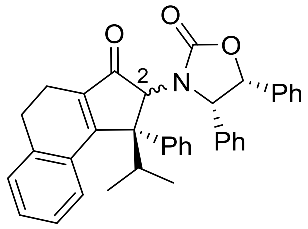

Resolved peaks of (1*R*)-**7d** reported from 2.8:1 mixture of (1*R*): (1*S*) **7d**

^{1}H NMR (400 MHz, $CDCl_3$) δ 7.83 (s, 1H), 7.61 (s, 1H), 7.48 (d, *J* = 7.0 Hz, 2H), 7.37 (s, 1H), 7.29 – 7.26 (m, 2H), 7.12 (d, *J* = 7.8 Hz, 1H), 7.06 – 6.88 (m, 8H), 6.60 (dd, *J* = 7.5, 1.5 Hz, 2H), 5.04 (s, 1H), 4.17 (d, *J* = 6.8 Hz, 1H), 3.89 (d, *J* = 7.0 Hz, 1H), 3.24 – 3.11 (m, 1H), 2.93 – 2.79 (m, 2H), 2.62 – 2.55 (m, 1H), 2.20 (ddd, *J* = 16.5, 14.9, 7.5 Hz, 1H), 1.01 (d, *J* = 6.9 Hz, 3H), 0.95 (d, *J* = 6.6 Hz, 3H).

7e (4*S*,5*R*)-3-((1*S*,2*S*)-1-methyl-3-oxo-1-pentyl-2,3,4,5-tetrahydro-1*H*-cyclopenta[*a*]naphthalen-2-yl)-4,5-diphenyloxazolidin-2-one

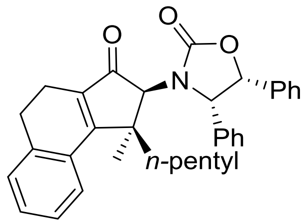

In a dried rbf with magnetic stir bar **5e** (0.0697 g, 0.138 mmol) was dissolved in anhydrous CH_2Cl_2 (1.35 mL) and cooled to -78 °C. TfOH (0.7 M in CH_2Cl_2, 0.24 mL, 0.168 mmol) was added and the reaction let warm to room temperature and stirred overnight. Sat. aq. $NaHCO_3$ (1 mL), H_2O (3 mL) and CH_2Cl_2 (5 mL) were added and the organic layer separated. The aq. layer was extracted with CH_2Cl_2 (2 x 5 mL) and the combined organic extracts were washed with brine, dried ($MgSO_4$), filtered and concentrated. The residue was dissolved in minimal toluene and purified by column chromatography (2.5% step gradient, 12.5-20% EtOAc in PS) to give **7e** (0.031 g, 0.061 mmol, 44%) as a yellow solid.

R_f = 0.35 (20% EtOAc in PS); MP = 208-211 °C; ^{1}H NMR (400 MHz, $CDCl_3$) δ 7.59 (d, *J* = 7.4 Hz, 1H), 7.34 – 7.21 (m, 4H), 7.18 – 6.96 (m, 9H), 5.99 (d, J = 8.2 Hz, 1H), 5.24 (d, *J* = 8.3 Hz, 1H), 3.53 (s, 1H), 2.93 – 2.66 (m, 2H), 2.63 – 2.41 (m, 2H), 2.00 (br s, 2H), 1.53 (s, 3H), 1.40 – 1.03 (m, 6H), 0.83 (br s, 3H). DEPT-Q ^{13}C NMR (101 MHz, $CDCl_3$) δ 167.3 (C), 140.5 (C), 136.4 (C), 134.7 (C), 130.9 (C), 130.4 (CH), 129.1 (CH), 128.7 (CH), 128.4 (CH), 128.1 (CH), 128.0 (CH), 126.7 (CH), 126.4 (CH), 126.3 (CH), 48.7 (C), 36.5 (CH_2), 28.5 (CH_2), 26.7 (CH_3), 25.7 (CH_2), 22.7 (CH_2), 18.0 (CH_2), 14.2 (CH_3); HRMS calcd. for $C_{34}H_{36}NO_3$ $(M+H)^+$: 506.2690, found 506.2700.

7g (4*S*,5*R*)-3-((1*S*)-5,7-dimethoxy-1-methyl-3-oxo-1-phenyl-2,3-dihydro-1*H*-inden-2-yl)-4,5-diphenyloxazolidin-2-one

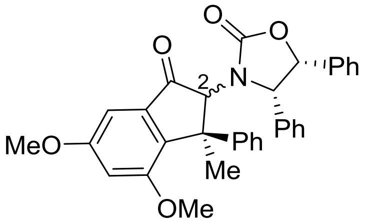

5g (0.1526 g, 0.2939 mmol) was placed in a dried rbf and dissolved in anhydrous CH_2Cl_2 (3 mL). BF_3.THF (0.034 mL, 0.306 mmol) was added and the reaction was stirred at rt for 20 h at which point completion of the reaction was determined by TLC analysis. Sat. aq. $NaHCO_3$ (2 mL), CH_2Cl_2 (5 mL) and H_2O (5 mL) were added, the organic layer was separated and the aqueous layer extracted with CH_2Cl_2 (2 x 5 mL). The combined organic layers were washed with brine, dried ($MgSO_4$), filtered and concentrated to yield **7g** (0.1426 g, 0.2746 mmol, 93%) as a 7:1 mixture of *cis*(2*S*):*trans*(2*R*) isomers (α-C).

R_f = 0.19 (20% EtOAc in PS); MP = 238-239 °C; ^{1}H NMR (400 MHz, $CDCl_3$) δ 7.45 – 7.27 (m, 4.6H), 7.19 – 7.06 (m, 5H), 7.05 – 6.99 (m, 4H), 6.98 – 6.89 (m, 1.4H), 6.85 – 6.77 (m, 2.2H), 6.72 (d, *J* = 2.2 Hz, 1H), 6.71 – 6.63 (m, 1.2H), 5.94 (d, *J* = 8.5 Hz, 0.15H) *trans*, 5.28 (d, *J* = 8.5 Hz, 0.14H) *trans*, 4.94 (d, *J* = 8.5 Hz, 0.13H), 4.77 (d, *J* = 8.5 Hz, 1H), 3.88 (s, 3H), 3.85 (s, 0.4H) *trans*, 3.79 (s, 0.15H) *trans*, 3.64 (d, *J* = 7.9 Hz, 1H), 3.62 (s, 3H), 3.47 (s, 0.4H) *trans*, 1.98 (s, 0.4H) *trans*, 1.87 (s, 3H). ^{13}C DEPT-Q NMR (101 MHz, $CDCl_3$) δ 198.7 (C), 161.7 (C), 157.5 (C), 155.6 (C), 141.4 (C), 140.2 (C), 134.4 (C), 133.7 (C), 128.4 (CH), 128.3 (CH), 127.9 (CH), 127.7 (CH), 127.2 (CH), 126.2 (CH), 107.3 (CH), 96.6 (CH), 80.3 (CH), 69.8

(CH), 66.1 (CH), 55.8 (CH_3), 55.6 (CH_3), 50.4 (C), 26.8 (CH_3); HRMS calcd. for $C_{33}H_{30}NO_5$ $(M+H)^+$: 520.2118, found 520.2131.

7h (4*S*,5*R*)-3-((1*S*,2*S*)-1-isopropyl-5,7-dimethoxy-3-oxo-1-phenyl-2,3-dihydro-1*H*-inden-2-yl)-4,5-diphenyloxazolidin-2-one

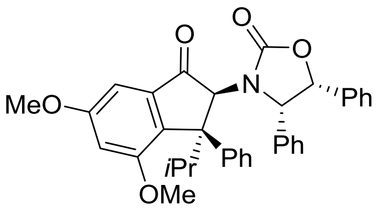

5h (95 mg, 0.173 mmol) was placed in a dried rbf equipped with magnetic stirrer and reflux condenser. CH_2Cl_2 (1.7 mL) was added followed by BF_3.THF (0.06 mL, 0.545 mmol) and the reaction was heated to reflux for 72 h at which point all starting material was consumed. The reaction was cooled, sat. aq. $NaHCO_3$ (2 mL) and CH_2Cl_2 (5 mL) were added, the organic layer was separated and the aqueous layer extracted with CH_2Cl_2 (2 x 5 mL). The combined organic layers were washed with brine, dried ($MgSO_4$), filtered and concentrated. The crude residue was dissolved in minimal toluene and purified by column chromatography (20% EtOAc in PS) provided the product in 79% yield as a 17:1 mixture of *cis*(2*S*):*trans*(2*R*) isomers with an additional isopropyl containing impurity, chromatography of this mixture (dissolved in toluene, 2.5% step gradient, 15-20% EtOAc in PS) avoiding the higher R_f impurity provided **7h** (48 mg, 0.087 mmol, 50%) as a yellow waxy solid.

R_f = 0.25 (20% EtOAc in PS); MP = 95-97 °C; ^{1}H NMR (400 MHz, $CDCl_3$) δ 7.44 (d, *J* = 4.0 Hz, 4H), 7.37 (dd, *J* = 8.3, 4.1 Hz, 1H), 7.17 – 7.07 (br, 4H), 7.05 – 6.95 (m, 5H), 6.74 – 6.66 (m, 3H), 4.55 (d, *J* = 8.2 Hz, 1H), 4.39 (d, *J* = 8.3 Hz, 1H), 3.91 (s, 3H), 3.68 (s, 1H), 3.57 (s, 3H), 2.85 (m, 1H), 0.91 (d, *J* = 6.8 Hz, 3H), 0.61 (d, *J* = 6.5 Hz, 3H); ^{13}C DEPT-Q NMR (101 MHz, $CDCl_3$) δ 200.1 (C), 161.6 (C), 158.0 (C), 156.2 (C), 140.7 (C), 140.3 (C), 136.3 (C), 134.3 (C), 133.7 (C), 129.0 (CH), 128.4 (CH), 128.4 (CH), 128.0 (CH), 127.8 (CH), 127.8 (CH), 127.0 (CH), 126.3 (CH), 107.1 (CH), 96.9 (CH), 80.6 (CH), 67.9 (CH), 66.4 (CH), 59.4 (C), 55.8 (CH_3), 55.3 (CH_3), 35.8 (CH), 20.8 (CH_3), 19.3 (CH_3); HRMS calcd. for $C_{35}H_{34}NO_5$ $(M+H)^+$: 548.2431, found 548.2432.

Note: The (2*R*) (*trans*) isomer appears to be formed initially in the reaction with isomerisation to the isolated *cis* isomer (2*S*) occurring prior to the completion of the reaction. Resolved peaks in ^{1}H NMR for (2*R*) *trans* isomer: ^{1}H NMR (400 MHz, $CDCl_3$) δ 6.79 (d, *J* = 2.2 Hz, 1H), 6.75 (d, *J* = 2.2 Hz, 1H), 5.97 (d, *J* = 6.9 Hz, 1H), 5.16 (d, *J* = 7.0 Hz, 1H), 4.96 (s, 1H), 3.79 (s, 3H), 3.63 (s, 3H), 3.16 (m, 1H), 1.09 (d, *J* = 6.7 Hz, 3H), 0.65 (d, *J* = 6.5 Hz, 3H).

7i (4*S*,5*R*)-3-((4*S*)-4-methyl-6-oxo-4-phenyl-5,6-dihydro-4*H*-cyclopenta[*b*]thiophen-5-yl)-4,5-diphenyloxazolidin-2-one

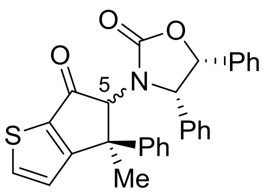

5i (0.138 mg, 0.298 mmol) was placed in a dried rbf and dissolved in anhydrous CH_2Cl_2 (3.3 mL). TfOH (0.034 mL, 0.328 mmol) was added and the reaction was stirred at rt for 48 h at which point completion of the reaction was determined by ^{1}H NMR analysis. Sat. aq. $NaHCO_3$ (2 mL), CH_2Cl_2 (5 mL) and H_2O (5 mL) were added, the organic layer was separated and the aqueous layer extracted with CH_2Cl_2 (2 x 5 mL). The combined organic layers were washed with brine, dried ($MgSO_4$) and concentrated. The residue was dissolved in minimal CH_2Cl_2 and PS and purified by column chromatography (2.5% step gradient, 15%-20% EtOAc in PS) to yield **7i** (0.105 g, 0.226 mmol, 76%) as a 4:1 mixture of *cis*(5*S*):*trans*(5*R*) isomers (α-C).

R_f = 0.18 (15% EtOAc in PS); MP = 237-239 °C; ^{1}H NMR (400 MHz, $CDCl_3$) δ 7.98 (d, *J* = 4.8 Hz, 1H), 7.90 (d, *J* = 4.8 Hz, 0.25H), 7.51 – 7.32 (m, 5.25H), 7.18 – 7.06 (m, 5.75H), 7.06 – 6.93 (m, 6H), 6.87 – 6.78 (m, 2.75H), 6.75 – 6.55 (m, 1.5H), 5.93 (d, *J* = 8.5 Hz, 0.25H) *trans*, 5.30 (d, *J* = 8.5 Hz, 0.25H) *trans*, 5.07 (d, *J* = 8.6 Hz, 1H), 4.89 (d, *J* = 8.6 Hz, 1H), 4.12 (s, 0.25H) *trans*, 3.94 (s, 1H), 1.96 (s, 0.75H) *trans*, 1.82 (s, 3H). ^{13}C DEPT-Q NMR (101 MHz, $CDCl_3$) δ 188.8 (C), 172.0 (C), 155.6 (C), 141.5 (CH), 140.7 (C), 139.2 (C), 134.4 (C), 133.5 (C), 128.5 (CH), 128.4 (CH), 128.3 (CH), 128.0 (CH), 127.9 (CH), 127.9 (CH), 127.8 (CH), 126.5 (CH), 126.2 (CH), 126.0 (CH), 125.8 (CH), 123.0 (CH), 80.2 (CH), 73.4 (CH), 66.1 (CH), 50.8 (C), 28.2 (CH_3); HRMS calcd. for $C_{29}H_{24}NO_3S$ $(M+H)^+$: 466.1471, found 466.1481.

Minor (5*R*) *trans* isomer (resolved peaks) ^{1}H NMR (400 MHz, $CDCl_3$) δ 7.90 (d, *J* = 4.8 Hz, 1H), 5.93 (d, *J* = 8.5 Hz, 1H), 5.30 (d, *J* = 8.5 Hz, 1H), 4.12 (s, 1H), 1.96 (s, 3H). ^{13}C NMR (101 MHz, $CDCl_3$) δ 191.3 (C), 174.3 (C), 158.5 (C), 145.2 (C), 141.7 (CH), 136.9 (C), 134.9 (C), 133.6 (C), 128.2 (CH), 127.9 (CH), 127.7 (CH), 127.5 (CH), 126.5 (CH), 126.0 (CH), 123.2 (CH), 79.8 (CH), 77.3 (CH), 69.9 (CH), 49.8 (C), 21.0 (CH_3).

11 (4*S*,5*R*)-3-(1-(4-methoxyphenyl)-1-oxo-3-phenylbut-3-en-2-yl)-4,5-diphenyloxazolidin-2-one

Formed in reactions using **5j** in CH_2Cl_2 with either TfOH or BF_3.THF as acid, the below NMR data is reported from a crude reaction mixture using BF_3.THF in CH_2Cl_2 at reflux after 24 h.

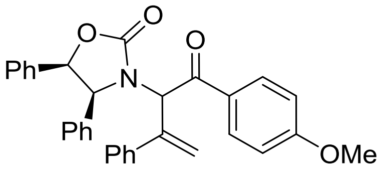

^{1}H NMR (400 MHz, $CDCl_3$) δ 7.56 – 7.51 (m, 1H), 7.37 (d, *J* = 8.8 Hz, 2H), 7.23 – 7.15 (m, 2H), 7.15 – 7.03 (m, 7H), 6.93 (dd, *J* = 5.2, 3.1 Hz, 3H), 6.82 (t, *J* = 7.7 Hz, 2H), 6.48 (d, *J* = 7.2 Hz, 2H), 6.16 (s, 1H), 5.92 (s, 1H), 5.79 (d, *J* = 8.6 Hz, 1H), 5.11 (d, *J* = 8.6 Hz, 1H), 3.94 (s, 3H), 3.01 (s, 1H); ^{13}C DEPT-Q NMR (101 MHz, $CDCl_3$) δ 160.1 (C), 143.0 (C), 140.68 (s), 135.0 (C), 134.3 (C), 133.4 (C), 130.1 (CH), 128.3 (CH), 128.3 (CH), 128.1 (CH), 128.0 (CH), 128.0 (CH), 127.7 (CH), 126.5 (CH), 126.0 (CH), 125.1 (C), 120.3 (CH), 120.1 (CH), 114.4 (CH), 112.6 (CH_2), 80.1 (CH), 66.7 (CH), 55.5 (CH_3), 37.6 (CH).

12 (+)-(*S*)-1-methyl-1-phenyl-1,2,4,5-tetrahydro-3*H*-cyclopenta[*a*]naphthalen-3-one

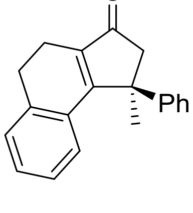

Prepared by General Method D from **7c** (0.133 g, 0.260 mmol). The crude residue was dissolved in minimal CH_2Cl_2 and PS and purified by column chromatography (2.5% step gradient, 10-15% EtOAc in PS) to give **12** (56 mg, 0.204 mmol, 79%) as a clear oil.

R_f = 0.42 (15% EtOAc in PS); $[\alpha]_D^{26}$=+72.1 degcm^{3}g^{-1}dm^{-1} (c=0.01 gcm^{-3}, $CHCl_3$); ^{1}H NMR (400 MHz, $CDCl_3$) δ 7.35 – 7.28 (m, 4H), 7.26 – 7.18 (m, 3H), 6.97 (ddd, *J* = 7.8, 7.3, 1.7 Hz, 1H), 6.80 (d, *J* = 7.7 Hz, 1H), 3.00 – 2.88 (m, 2H), 2.75 – 2.65 (m, 3H), 2.54 (ddd, *J* = 16.6, 11.5, 8.5 Hz, 1H), 1.86 (s, 3H); ^{13}C DEPT-Q NMR (101 MHz, $CDCl_3$) δ 206.6 (C), 170.8 (C), 146.2 (C), 140.3 (C), 138.1 (C), 130.3 (C), 130.2 (CH), 129.1 (CH), 128.6 (CH), 127.3 (CH), 126.8 (CH), 126.5 (CH), 125.7 (CH), 56.9 (CH_2), 47.0 (C), 28.6 (CH_2), 25.4 (CH_3), 18.3 (CH_2); HRMS calcd. for $C_{20}H_{19}O$ $(M+H)^+$: 275.1430, found 275.1438; Chiral HPLC (98:2 er, Lux Cellulose-2 column, 4.6 mm x 150 mm, UV detection at 214 nm, eluent: PS:EtOH (97:3), 1 mL/min) t_{R1} (major) =13.543 min, t_{R2} (minor) = 14.805).

13 (+)-(*S*)-4-methyl-4-phenyl-4,5-dihydro-6*H*-cyclopenta[*b*]thiophen-6-one

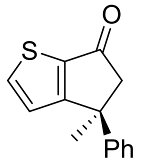

Prepared by General Method D from **7i** (41 mg, 0.0881 mmol). The crude residue was dissolved in toluene and PS and purified by column chromatography (2.5% step gradient, 7.5%-10% EtOAc in PS) to give **13** (11.0 mg, 0.0483 mmol, 55%) as a waxy solid.

R_f = 0.4 (10% EtOAc in PS); $[\alpha]_D^{26}$=+13.5 degcm^{3}g^{-1}dm^{-1} (c=0.01 gcm^{-3}, $CHCl_3$); ^{1}H NMR (400 MHz, $CDCl_3$) δ 7.91 (d, *J* = 4.8 Hz, 1H), 7.34 – 7.28 (m, 2H), 7.25 – 7.20 (m, 3H), 6.96 (d, *J* = 4.8 Hz, 1H), 3.22 (d, *J* = 18.4 Hz, 1H), 3.14 (d, *J* = 18.4 Hz, 1H), 1.82 (s, 3H); ^{13}C DEPT-Q NMR (101 MHz, $CDCl_3$) δ 196.0 (C), 175.8 (C), 146.2 (C), 141.0 (CH), 140.1 (C), 128.8 (CH), 126.8 (CH), 125.9 (CH), 123.3 (CH), 60.0 (CH_2), 45.2 (C), 28.1 (CH_3); HRMS calcd. for $C_{14}H_{13}OS$ $(M+H)^+$: 228.0676, found 228.0682; Chiral HPLC (>99:1 er, Lux Cellulose-2 column, 4.6 mm x 150 mm, UV detection at 254 nm, eluent: PS:EtOH (97:3), 1 mL/min) t_{R1} (major) =9.315 min, t_{R2} (minor) = not detected).

14 (4*S*,5*R*)-3-((1*R*,2*R*,3a*S*,9b*S*)-1-methyl-3a-(1-methyl-1*H*-indol-3-yl)-3-oxo-1-phenyl-2,3,3a,4,5,9b-hexahydro-1*H*-cyclopenta[*a*]naphthalen-2-yl)-4,5-diphenyloxazolidin-2-one

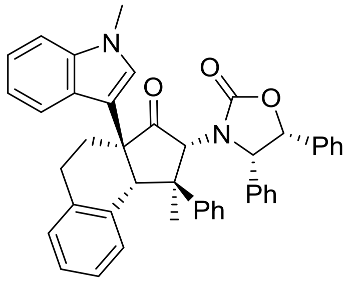

To a dried rbf equipped with a magnetic stirrer divinylketone **5c** (51 mg, 0.1 mmol) and 1-methylindole (0.13 mL, 1.0 mmol) were added, dissolved in anhydrous CH_2Cl_2 (1 mL) and cooled to -78 °C. A 0.6 M solution of TfOH (0.5 mL, 0.3 mmol) was added, the reaction was let warm to rt and stirred for 48 h resulting in formation of a product assigned as the C2-(*S*) isomer. Resolved peaks assigned to (2*S*)-**14** ^{1}H NMR (400 MHz, $CDCl_3$) δ 5.36 (d, *J* = 8.2 Hz, 1H), 4.83 (d, *J* = 8.4 Hz, 1H), 4.00 (s, 1H), 0.87 (s, 3H). Sat. aq. $NaHCO_3$ (2 mL), CH_2Cl_2 (5 mL) and H_2O (5 mL) were added, the organic layer separated and the aq. layer extracted with CH_2Cl_2 (2 x 5 mL). The combined organic layers were washed with brine, dried ($MgSO_4$), filtered and concentrated. The residue was dissolved in toluene and purified by column chromatography (2.5% step gradient, 12.5%-17.5% EtOAc in PS) on silica pretreated with 12.5% EtOAc, 1% Et_3N, 86.5% PS to give **14** (48 mg, 0.744 mmol, 74%) as a brown solid.

R_f = 0.29 (20% EtOAc in PS); MP = 142-145 °C; ^{1}H NMR (400 MHz, $CDCl_3$) δ 8.07 (d, *J* = 8.1 Hz, 1H), 7.28 (d, *J* = 8.1 Hz, 1H), 7.26 – 7.21 (m, 1H), 7.17 – 7.01 (m, 11H), 6.91 – 6.79 (m, 4H), 6.75 (s, 1H), 6.63 (t, *J* = 7.1 Hz, 2H), 6.37 (d, *J* = 7.1 Hz, 2H), 6.20 (d, *J* = 7.7 Hz, 1H), 5.73 (d, *J* = 8.4 Hz, 1H), 5.11 (d, *J* = 8.4 Hz, 1H), 4.64 (s, 1H), 4.37 (s, 1H), 3.66 (s, 3H), 2.74 – 2.56 (m, 3H), 2.48 – 2.38 (m, 1H), 1.46 (s, 3H); ^{13}C DEPT-Q NMR (101 MHz, $CDCl_3$) δ 211.5 (C), 158.9 (C), 146.8 (C), 138.0 (C), 136.9 (C), 135.0 (C), 133.6 (C), 133.4 (C), 129.1 (CH), 128.56 (CH), 128.3 (CH), 127.9 (CH), 127.8 (CH), 127.8 (CH), 127.6 (CH), 127.0 (CH), 126.7 (CH), 126.6 (CH), 126.4 (CH), 126.2 (CH), 125.8 (C), 125.7 (CH), 122.5 (CH), 122.0 (CH), 119.4 (CH), 116.2 (CH), 109.4 (CH), 79.9 (CH), 73.9 (CH), 69.4 (CH), 54.1 (CH_3), 50.0 (C), 49.6 (C), 32.9 (CH), 29.7 (CH_2), 26.4 (CH_2), 18.4 (CH_3); HRMS calcd. for $C_{44}H_{39}N_2O_3$ $(M+H)^+$: 643.2955, found 643.2959.

15 (*Z*)-2-((4*S*,5*R*)-2-oxo-4,5-diphenyloxazolidin-3-yl)-3-phenylbut-2-enal

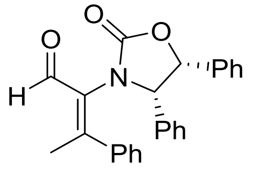

Ynamide **4a** (0.816 g, 2.404 mmol) was placed in a rbf, evacuated and backfilled with N_2 and dissolved in anhydrous CH_2Cl_2 (8 mL), CuBr.SMe_2 (0.0493 g, 0.24 mmol) was added followed by anhydrous Et_2O (48 mL). The reaction was cooled to -40 °C and MeMgBr (2.8 M in Et_2O, 1.72 mL, 4.8 mmol) was added, the resulting brown suspension was stirred at this temperature for 0.5 h. The reaction was cooled to -78 °C, ethyl formate (0.782 mL, 9.6 mmol) was added, the reaction was let warm to rt and stirred for 2 h. 10% aq. NH_4Cl (10 mL) was added followed by EtOAc (50 mL) and H_2O (20 mL), the organic layer was separated and the aq. layer extracted twice with EtOAc (2 x 50 mL). The organic layers were combined, washed with brine, dried ($MgSO_4$), filtered and concentrated. The residue was dissolved in toluene and purified by column chromatography (2.5% step gradient, 20-25% EtOAc in PS) to give **15** (0.478 g, 1.247 mmol, 52%) as an orange solid.

R_f = 0.24 (20% EtOAc in PS); MP = 129-131 °C; ^{1}H NMR (400 MHz, $CDCl_3$) δ 10.02 (s, 1H), 7.51 – 7.42 (m, 3H), 7.30 – 7.26 (m, 2H), 7.08 – 6.95 (m, 4H), 6.83 (dd, *J* = 9.8, 5.0 Hz, 4H), 6.34 (d, *J* = 7.5 Hz, 2H), 5.59 (d, *J* = 8.8 Hz, 1H), 4.71 (br d, *J* = 7.9 Hz, 1H), 2.42 (s, 3H); ^{13}C DEPT-Q NMR (101 MHz, $CDCl_3$) δ 186.1, 158.3, 158.1, 139.8, 135.0, 133.1, 131.0, 129.7, 128.8, 128.6, 128.3, 128.0, 127.8, 127.7, 127.1, 126.2, 80.4, 66.0, 19.7; HRMS calcd. for $C_{25}H_{22}NO_3$ $(M+H)^+$: 384.1594, found 384.1597

17 (4*S*,5*R*)-3-((2*Z*,5*E*)-5-ethyl-6-methyl-4-oxo-2-phenylocta-2,5-dien-3-yl)-4,5-diphenyloxazolidin-2-one

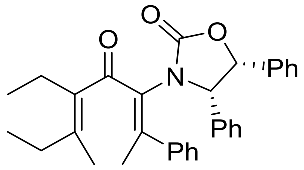

In a dried Schlenk tube equipped with a stirrer bar, $ZrCp_2Cl_2$ (76 mg, 0.2615 mmol) was added and suspended in anhydrous 1,2-dichlorethane (8 mL). $AlMe_3$ (2 M in Toluene, 1.74 mL, 3.49 mmol) was added dropwise and stirred for 2 min. 3-hexyne (0.3 mL, 2.62 mmol) was added and the clear yellow solution was heated to 60°C for 5 h. After this time the reaction was cooled to room temperature and in a separate dried rbf with stirrer bar, aldehyde **15** (0.227 g, 0.581 mmol) was dissolved in anhydrous CH_2Cl_2 (5.8 mL) and cooled to -78 °C. The vinylalane solution was added dropwise to the cooled aldehyde solution and stirred for 5 min, 5 mL of an aq. Rochelle's salt (9%) and K_2CO_3 (1%) solution was then added followed by H_2O (5 mL) and the reaction warmed to room temperature. The reaction

mixture was passed through a pad of celite, washed with CH_2Cl_2 (10 mL) the organic layer separated and the aq. layer extracted with CH_2Cl_2 (2 x 10 mL). The combined organic layers were washed with brine, dried ($MgSO_4$), filtered and concentrated to yield the intermediate carbinol as an apparent mixture of alkene isomers (indicated by a ~3:1 ratio of doublets at 5.44 and 5.48 ppm in $CDCl_3$ ^1H NMR). The crude mixture was dissolved in CH_2Cl_2 (6 mL), $NaHCO_3$ (0.146 g, 1.74 mmol) was added and the mixture cooled to 0 °C. Dess-Martin periodinane (0.246 g, 0.581 mmol) was added and the reaction stirred for 1 h. At this time H_2O (5 mL) and CH_2Cl_2 (10 mL) were added, the organic layer separated and the aq. layer extracted with CH_2Cl_2 (2 x 5 mL). The combined organic layers were washed with brine, dried ($MgSO_4$), filtered and concentrated, to give a 3:1 ratio of (2*Z*,5*E*:2*Z*,5*Z*)-**17**. The resulting oil was dissolved in minimal toluene and purified by column chromatography (1% step gradient, 10-12% EtOAc in PS). As no discernible difference between isomers could be detected by TLC in a range of solvent systems nor with analytical HPLC, ^1H NMR was used to assess the isomeric purity of fractions prior to combination and evaporation. Early fractions gave product enriched with the minor (*Z*,*Z*) isomer which were combined and re-chromatographed under the same conditions to give pure (4*S*,5*R*)-3-((2*Z*,5*Z*)-5-ethyl-6-methyl-4-oxo-2-phenylocta-2,5-dien-3-yl)-4,5-diphenyloxazolidin-2-one (2*Z*,5*Z*)-**17** (0.0192 g, 0.04 mmol, 7%) as a resin. Later fractions of (2*Z*,5*E*)-**17** free from the (2*Z*,5*Z*) isomer could be contaminated by a slightly lower R_f by-product assignable to a product of oxazolidinone rearrangement analogous to **SI-3** (see below) which could be observed by TLC (10% EtOAc in PS) when the TLC was rerun 2 or 3 times. Avoiding these impurities resulted in isolation of (2*Z*,5*E*)-**17** (60 mg, 0.125 mmol, 22%) as a pale solid. A 2:1 mixture of (*Z*,*E*):(*Z*,*Z*) isomers of **17** (0.109, 0.227 mmol, 39%) was also isolated.

(2*Z*,5*E*)-**17**

R_f = 0.26 (10% EtOAc in PS); MP = 64-65 °C; ^1H NMR (400 MHz, $CDCl_3$) δ 7.43 – 7.39 (m, 3H), 7.22 – 7.14 (m, 2H), 7.05 – 6.95 (m, 4H), 6.89 – 6.84 (m, 4H), 6.54 (d, *J* = 7.2 Hz, 2H), 5.54 (d, *J* = 8.7 Hz, 1H), 4.56 (d, *J* = 8.7 Hz, 1H), 2.38 (dd, *J* = 14.8, 7.4 Hz, 2H), 2.23 – 2.07 (m, 5H), 1.83 (s, 3H), 1.04 (m, 6H); ^{13}C DEPT-Q NMR (101 MHz, $CDCl_3$) δ 196.3 (C), 157.8 (C), 147.1 (C), 141.9 (C), 141.4 (C), 138.8 (C), 135.3 (C), 133.4 (C), 131.1 (C), 128.9 (CH), 128.7 (CH), 128.6 (CH), 128.0 (CH), 127.8 (CH), 127.7 (CH), 127.4 (CH), 126.3 (CH), 80.5 (CH), 65.8 (CH), 27.5 (CH_2), 23.2 (CH_2), 21.6 (CH_3), 19.5 (CH_3), 14.0 (CH_3), 11.8 (CH_3); HRMS calcd. for $C_{32}H_{34}NO_3$ $(M+H)^+$: 480.2533, found 480.2505.

(2*Z*,5*Z*)-**17**

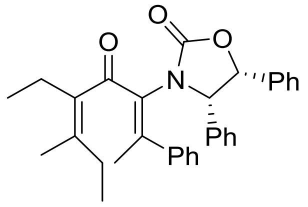

R_f = 0.26 (10% EtOAc in PS); ^1H NMR (400 MHz, $CDCl_3$) δ 7.43 – 7.36 (m, 3H), 7.20 – 7.16 (m, 2H), 7.05 – 6.94 (m, 4H), 6.89 – 6.79 (m, 4H), 6.49 (d, *J* = 7.2 Hz, 2H), 5.54 (d, *J* = 8.7 Hz, 1H), 4.63 (d, *J* = 8.7 Hz, 1H), 2.31 (q, *J* = 7.5 Hz, 2H), 2.23 – 2.07 (m, 2H), 2.12 (s, 3H), 1.75 (s, 3H), 1.02 (t, *J* = 7.5 Hz, 3H), 0.97 (t, *J* = 7.5 Hz, 3H); ^{13}C DEPT-Q NMR (101 MHz, $CDCl_3$) δ 195.8 (C), 158.0 (C), 148.2 (C), 141.6 (C), 141.5 (C), 138.8 (C), 135.3 (C), 133.5 (C), 131.2 (C), 128.9 (CH), 128.5 (CH), 128.5 (CH), 127.9 (CH), 127.7 (CH), 127.6 (CH), 127.3 (CH), 127.2 (CH), 126.3 (CH), 80.3 (CH), 65.9 (CH), 28.9 (CH_2), 23.6 (CH_2), 21.9 (CH_3), 17.5 (CH_3), 13.2 (CH_3), 12.6 (CH_3); HRMS calcd. for $C_{32}H_{34}NO_3$ $(M+H)^+$: 480.2533, found 480.2506.

18 (*E*)-3-bromo-2-methylbut-2-en-1-ol

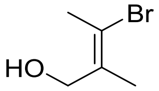

Adapted from the procedures of Campbell and Sammis,[12] Buckles and Mock,[13] and Curran and Kuo[14]

A solution of bromine (8.88 mL, 27.7 g, 173 mmol) in $CHCl_3$ (17 mL) was added dropwise to a solution of tiglic acid (17 g, 0.170 mol) in $CHCl_3$ (170 mL) at 45 °C under N_2 and stirred for 1.5 h. The mixure was cooled and sat. aq. $NaHCO_3$ (180 mL) was added, the organic layer was separated and washed with sat. aq. $NaHCO_3$ (2 x 100 mL). The combined aq. layers were then acidified with 37% aq. HCl cooled to 4 °C overnight and the solid filtered to give 2,3-dibromo-2-methylbutanoic acid (45 g, quantitative) ^1H NMR (401 MHz, $CDCl_3$) δ 4.85 (q, *J* = 6.7 Hz, 1H), 1.99 (s, 3H), 1.91 (d, *J* = 6.8 Hz, 3H). 2,3-dibromo-2-methylbutanoic acid (22.5 g, 0.085 mol) was dissolved in MeOH (12 mL) and 120g of a 25% KOH in MeOH solution (30 g, 0.534 mol) was added slowly with cooling in an ice bath, K_2CO_3 (2.35 g, 0.017 mol) was added and the reaction was heated to 55 °C for 2 h at which point the reaction mixture was poured into a 6M aq. HCl solution (100 mL) and diluted with H_2O (300 mL). The mixture was then cooled to 0 °C for 4 h and filtered to yield (*E*)-3-bromo-2-methylbut-2-enoic acid (9.5 g, 0.053 mmol, 62% from dibromide) as a white solid ^1H NMR (400 MHz, $CDCl_3$) δ 2.76 (q, *J* = 1.6 Hz, 1H), 2.12 (q, *J* = 1.6 Hz, 1H) in accordance with previously reported data.[12] In a dried two necked rbf containing a magnetic stir bar (*E*)-3-bromo-2-methylbut-2-enoic acid (4.95 g, 27.7 mmol) was dissolved in anhydrous THF (270 mmol) and cooled to 0 °C. $LiAlH_4$ (1.32 g, 35 mmol) was added in two portions and the reaction let warm to rt and stirred for 5 h. Water (1.3 mL) was added dropwise followed by 3M aq. NaOH (1.3 mL) and water (4 mL), the reaction mixture was filtered and washed with Et_2O (3 x 80 mL). The combined organic layers were washed with brine (20 mL), dried ($MgSO_4$) filtered and evaporated to give **18** (3.0 g, 18.2 mmol, 66%) which was used without further purification. ^1H NMR (401 MHz, $CDCl_3$) δ 4.11 (s, 2H), 2.31 (d, *J* = 1.5 Hz, 3H), 1.89 (q, *J* = 1.5 Hz, 3H) in accordance with previously reported data.[14]

19 (*E*)-1-((3-bromo-2-methylbut-2-en-1-yl)oxy)-3,5-dimethoxybenzene

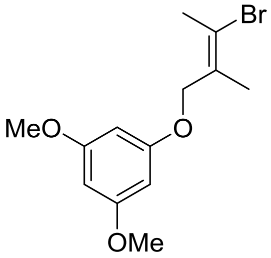

Vinyl bromide **18** (0.790 g, 4.78 mmol) was dissolved in anhydrous CH_2Cl_2 (28 mL), Et_3N (1.22 mL, 8.85 mmol) was added and the reaction cooled to 0 °C. MsCl (0.55 mL, 7.2 mmol) was added slowly and the reaction stirred for 10 minutes. Ice was added to the reaction followed by H_2O (10mL), CH_2Cl_2 (10 mL) and sat. aq. $NaHCO_3$ (10 mL), the organic layer separated, washed with H_2O (15 mL) and brine (15 mL), dried ($MgSO_4$), filtered and concentrated. To give (*E*)-3-bromo-2-methylbut-2-en-1-yl methanesulfonate (1.288 g) which was used without further purification. ^1H NMR (400 MHz, $CDCl_3$) δ 4.79 – 4.71 (m, 1H), 3.03 – 2.97 (m, 2H), 2.44 (dd, *J* = 2.9, 1.3 Hz, 1H), 1.97 (q, *J* = 1.5 Hz, 2H). The crude mesylate was placed in a dried rbf dissolved in anhydrous MeCN (24 mL), K_2CO_3 (0.992 g, 7.176 mmol) followed by 3,5-dimethoxyphenol (0.775 g, 5.02 mmol) were added, the reaction was heated to 50 °C for 4 h at which point ^1H NMR analysis indicated consumption of starting material. The reaction mixture was diluted with EtOAc (25 mL), filtered and concentrated, dissolved in minimal toluene and

purified by column chromatography (0.5% step gradient, 2-3% EtOAc in PS) to give **19** (0.767 g, 2.54 mmol, 53%) as a clear oil. R_f = 0.28 (2% EtOAc in PS); ^{1}H NMR (400 MHz, $CDCl_3$) δ 6.11 – 6.10 (m, 1H), 6.08 (d, *J* = 2.1 Hz, 2H), 4.47 (s, 2H), 3.77 (s, 6H), 2.41 (d, *J* = 1.2 Hz, 3H), 1.99 (dd, *J* = 2.9, 1.4 Hz, 3H). ^{13}C DEPT-Q NMR (101 MHz, $CDCl_3$) δ 161.7 (C), 160.7 (C), 130.5 (C), 123.0 (C), 93.7 (CH), 93.5 (CH), 67.7 (CH_2), 55.5 (CH_3), 25.4 (CH_3), 21.7 (CH_3). HRMS calcd. for $C_{13}H_{18}BrO_3$ $(M+H)^+$: 301.0434, found 301.0442.

20 (4*S*,5*R*)-3-((2*Z*,5*E*)-7-(3,5-dimethoxyphenoxy)-5,6-dimethyl-4-oxo-2-phenylhepta-2,5-dien-3-yl)-4,5-diphenyloxazolidin-2-one

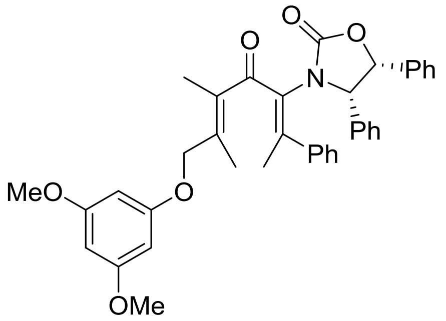

In a dried rbf equipped with a magnetic stir bar vinyl bromide **19** (188 mg, 0.626 mmol) was dissolved in anhydrous Et_2O (4 mL) and cooled to -78 °C. *t*-BuLi (1.5 M in pentane, 0.82 mL, 1.25 mmol) was added slowly and the reaction stirred for 0.5 h. In a dried rbf equipped with a magnetic stir bar aldehyde **15** (80 mg, 0.209 mmol) was dissolved in THF (0.1 mL), Et_2O (2 mL) was added and cooled to -78 °C, $AlMe_3$ (2 M in toluene, 0.1 mL, 0.2 mmol) was added followed by the vinyl lithium solution prepared above. The reaction was stirred for 5 min. quenched with aq. $NaHCO_3$ (4 mL) and warmed to room temperature. The organic layer was separated, the aq. layer extracted with Et_2O (10 mL x 2), the combined organic layers were washed with brine, dried ($MgSO_4$), filtered and concentrated. The residue was dissolved in CH_2Cl_2 (5 mL), $NaHCO_3$ (0.1051 g, 1.251 mmol) was added, the mixture cooled to 0 °C, Dess-Martin periodinane (174 mg, 0.411 mmol) was added and the reaction stirred for 2 h. At this time H_2O (5 mL), 10% aq. $Na_2S_2O_3$ (2 mL) and CH_2Cl_2 (10 mL) were added, the organic layer separated and the aq. layer extracted with CH_2Cl_2 (2 x 5 mL). The combined organic layers were washed with brine, dried ($MgSO_4$), filtered and concentrated, the resulting oil was dissolved in minimal toluene and purified by column chromatography (2.5% step gradient, 17.5-20% EtOAc in PS) giving (4*S*,5*R*)-3-((2*Z*,5*E*)-7-(3,5-dimethoxyphenoxy)-5,6-dimethyl-4-oxo-2-phenylhepta-2,5-dien-3-yl)-4,5-diphenyloxazolidin-2-one **20** (0.0559 g, 0.0925 mmol, 44%). Later eluting fractions could be contaminated with small amounts of **SI-3**, the purity of individual fractions was determined by ^{1}H NMR.

R_f = 0.25 (20% EtOAc in PS); ^{1}H NMR (401 MHz, $CDCl_3$) δ 7.53 – 7.38 (m, 3H), 7.15 (dd, *J* = 6.5, 2.9 Hz, 2H), 7.08 – 6.97 (m, 4H), 6.89 (t, *J* = 7.6 Hz, 2H), 6.83 (dd, *J* = 6.4, 2.8 Hz, 2H), 6.57 (d, *J* = 7.5 Hz, 2H), 6.08 – 6.04 (m, 3H), 5.54 (d, *J* = 8.7 Hz, 1H), 4.58 – 4.48 (m, 3H), 3.74 (s, 6H), 2.06 (s, 3H), 2.03 (s, 3H), 2.00 (s, 3H); ^{13}C DEPT-Q NMR (101 MHz, $CDCl_3$) δ 195.9 (C), 161.7 (C), 160.7 (C), 157.6 (C), 141.0 (C), 136.9 (C), 135.8 (C), 135.2 (C), 133.4 (C), 130.4 (C), 129.0 (CH), 128.9 (CH), 128.8 (CH), 128.2 (CH), 128.0 (CH), 127.8 (CH), 127.6 (CH), 127.5 (CH), 126.4 (CH), 93.7 (CH), 93.3 (CH), 80.7 (CH), 68.7 (CH_2), 65.6 (CH), 55.5 (CH_3), 21.4 (CH_3), 17.8 (CH_3), 15.6 (CH_3); HRMS calcd. for $C_{38}H_{38}NO_6$ $(M+H)^+$: 604.2694, found 604.2693.

SI-3 (*Z*)-5-((*E*)-4-(3,5-dimethoxyphenoxy)-3-methylbut-2-en-2-yl)-3-((*S*)-2-oxo-1,2-diphenylethyl)-4-(1-phenylethylidene)oxazolidin-2-one (Isolated from reactions for the synthesis of **20** performed in the absence of $AlMe_3$)

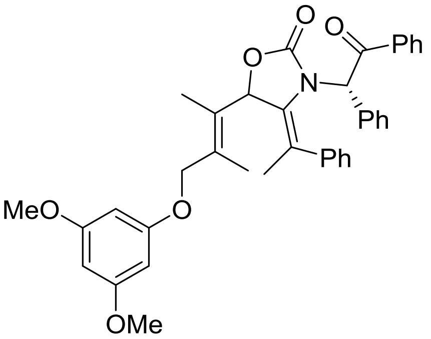

R_f = 0.25 (20% EtOAc in PS); ^{1}H NMR (401 MHz, $CDCl_3$) δ 7.46 – 7.37 (m, 3H), 7.28 (s, 2H), 7.24 – 7.03 (m, 9H), 6.94 – 6.89 (m, 2H), 6.11 (d, *J* = 1.3 Hz, 1H), 6.07 (s, 3H), 5.74 (s, 1H), 4.65 (d, *J* = 11.7 Hz, 1H), 4.50 (d, *J* = 11.7 Hz, 1H), 3.75 (s, 6H), 2.01 (d, *J* = 1.4 Hz, 3H), 1.87 (d, *J* = 1.1 Hz, 3H), 1.76 (d, *J* = 1.3 Hz, 3H). ^{13}C NMR (101 MHz, $CDCl_3$) δ 193.6 (C), 161.7 (C), 160.8 (C), 156.5 (C), 140.5 (C), 135.8 (C), 134.4 (C), 133.9 (C), 132.9 (C), 130.5 (C), 129.6 (CH), 129.4 (CH), 129.1 (CH), 128.8 (CH), 128.5 (CH), 128.3 (CH), 128.2 (CH), 127.9 (C), 127.7 (CH), 108.5 (C), 93.8 (CH), 93.4 (CH), 77.5 (CH) (N.B. overlapped with $CDCl_3$, observed in HSQC), 68.8 (CH_2), 63.8 (CH), 55.5 (CH_3), 22.0 (CH_3), 15.8 (CH_3), 12.0 (CH_3); HRMS calcd. for $C_{38}H_{38}NO_6$ $(M+H)^+$: 604.2694, found 604.2734;

23 (4*S*,5*R*)-3-(4,4-diethyl-2,3-dimethyl-5-oxo-3-phenylcyclopent-1-en-1-yl)-4,5-diphenyloxazolidin-2-one

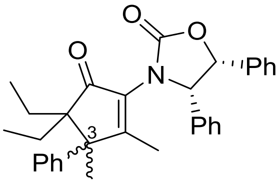

N.B. The following procedure was performed to specifically enable the preparation and isolation of **23**. Small scale reactions performed under the same conditions using isomerically pure **17** gave similar crude spectra, with slightly altered ratios of (3*S*)- and (3*R*)- **23**.

In a dried rbf a 2:1 (2*Z*,5*Z*):(2*Z*,5*E*) mixture of **17** (0.1378 g, 0.2877 mmol) was dissolved in anhydrous CH_2Cl_2 (2.9 mL) to this solution, $MeSO_3H$ (0.184 mL, 2.877 mmol) was added and the reaction was stirred at rt for 20 h. Sat. aq. $NaHCO_3$ (1 mL), H_2O (5 mL) and CH_2Cl_2 (5 mL) were added, the organic layer was separated and the aq. layer extracted with CH_2Cl_2 (2 x 5 mL). The combined organic layers were washed with brine, dried ($MgSO_4$), filtered and concentrated to give **23** as a 7:4 mixture of *S*:*R* isomers at C3 along with minor amounts of products possibly resulting from proton elimination without rearrangement. The residue was dissolved in minimal CH_2Cl_2 and PS and purified by column chromatography (step gradient, 30:2.5:67.5 to 30:5:65 CH_2Cl_2:EtOAc:PS) to give (3*S*)-**23** (33 mg, 0.0705 mmol, 25%) and (3*R*)-**23** (27 mg, 20%) in ~90% purity which was rechromatographed to yield pure (3*R*)-**23** (3.7 mg, 0.0077 mmol, 3%). Crystals of (3*S*)-**23** for X-ray diffraction were grown via solvent diffusion ($CHCl_3$, PS as antisolvent).

(3*S*)-**23**

R_f = 0.31 (30:5:65 CH_2Cl_2:EtOAc:PS); MP = 154-156 °C; ^{1}H NMR (400 MHz, $CDCl_3$) δ 7.29 – 7.23 (m, 4H), 7.22 – 7.18 (m, 1H), 7.16 – 6.97 (m, 9H), 6.82 (d, *J* = 6.9 Hz, 2H), 6.16 (d, *J* = 8.8 Hz, 1H), 6.01 (d, *J* = 8.8

Hz, 1H), 1.89 (s, 3H), 1.60 – 1.49 (m, 1H), 1.49 – 1.41 (m, 4H), 0.62 – 0.53 (m, 1H), 0.53 – 0.47 (m, 4H), 0.32 (t, *J* = 7.4 Hz, 3H); ^{13}C DEPT-Q NMR (101 MHz, $CDCl_3$) δ 206.8 (C), 169.4 (C), 155.6 (C), 142.4 (C), 135.2 (C), 134.2 (C), 132.4 (C), 128.5 (CH), 128.4 (CH), 128.1 (CH), 127.3 (CH), 126.9 (CH), 126.0 (CH), 80.1 (CH), 63.9 (CH), 57.9 (C), 55.6 (C), 26.4 (CH_2), 21.2 (CH_2), 19.5 (CH_3), 13.4 (CH_3), 8.5 (CH_3), 7.2 (CH_3); HRMS calcd. for $C_{32}H_{34}NO_3$ $(M+H)^+$: 480.2533, found 480.2531.

(3*R*)-**23**

R_f = 0.23 (30:5:65 CH_2Cl_2:EtOAc:PS); MP = 196-198 °C; ^{1}H NMR (400 MHz, $CDCl_3$) δ 7.19 – 7.03 (m, 10H), 7.00 (d, *J* = 4.7 Hz, 2H), 6.95 – 6.90 (m, 2H), 6.59 (s, 1H), 6.30 (d, *J* = 9.0 Hz, 1H), 6.07 (d, *J* = 9.0 Hz, 1H), 1.83 (s, 3H), 1.75 – 1.61 (m, 2H), 1.60 (s, 3H), 1.06 – 0.96 (m, 1H), 0.80 (t, *J* = 7.4 Hz, 3H), 0.48 – 0.40 (m, 1H), 0.39 – 0.35 (m, 3H); ^{13}C DEPT-Q NMR (101 MHz, $CDCl_3$) δ 207.1 (C), 171.8 (C), 155.3 (C), 141.6 (C), 135.3 (C), 134.8 (C), 131.7 (C), 128.8 (CH), 128.6 (CH), 128.2 (CH), 128.1 (CH), 128.0 (CH), 127.8 (CH), 126.5 (CH), 125.9 (CH), 79.7 (CH), 64.3 (CH), 57.2 (C), 55.8 (C), 24.3 (CH_2), 23.8 (CH_2), 21.1 (CH_3), 14.2 (CH_3), 8.7 (CH_3), 7.4 (CH_3); HRMS calcd. for $C_{32}H_{34}NO_3$ $(M+H)^+$: 480.2533, found 480.2524.

24 (4*S*,5*R*)-3-((1*S*,2*S*,3*S*)-3-ethyl-4-(*E/Z*)-ethylidene-2,3-dimethyl-5-oxo-2-phenylcyclopentyl)-4,5-diphenyloxazolidin-2-one

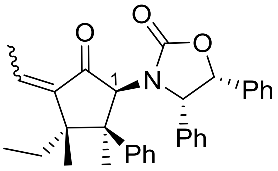

To a dried rbf equipped with a magnetic stirrer bar (2*Z*,5*E*)-**17** (71 mg, 0.149 mmol) was added and dissolved in anhydrous 1,4-dioxane (3 mL). $MeSO_3H$ (0.096 mL, 1.49 mmol) was added and the reaction stirred at rt for 18 h before being heated to 60 °C for 30 h at which point two major products were observed by ^{1}H NMR ((*Z*)- and (*E*)-**24**). Analysis of the reaction prior to this point indicated the presence starting material along with a third product which disappeared over time and is attributed to the C1-(*R*) isomer of (*Z*)-**24** which slowly isomerises to C1-(*S*) (*Z*)-**24**. $NaHCO_3$ (2 mL), H_2O (5 mL) and EtOAc (10 mL) were added, the organic layer was separated and the aq. layer extracted with EtOAc (2 x 5 mL). The combined organic layers were washed with brine, dried ($MgSO_4$), filtered and concentrated to give a 5:2 (*Z*:*E*) ratio of crude **24**. The residue was dissolved in minimal toluene and PS and purified by column chromatography (2.5% step gradient, 5-10% EtOAc in PS) to give (*Z*)-**24** (0.0219 g, 0.0457 mmol, 31%), (*E*)-**24** (9 mg, 0.0192 mmol, 13%) and 6 mg (8%) of a 3:2 mix of the *E*:*Z* alkene isomers of **24** (52% combined yield). Crystals of (*E*)-**24** for X-ray diffraction were grown via solvent diffusion ($CHCl_3$, PS as antisolvent).

(*Z*)-**24**

Peaks attributed the C1-(*R*) isomer initially observed: ^{1}H NMR (400 MHz, $CDCl_3$) δ 6.11 (q, *J* = 7.3 Hz, 1H), 5.80 (d, *J* = 8.4 Hz, 1H), 5.00 (d, *J* = 8.5 Hz, 1H).

R_f = 0.32 (10% EtOAc in PS); MP = 128-130 °C; ^{1}H NMR (400 MHz, $CDCl_3$) δ 7.19 – 7.06 (br s, 2H), 7.01 – 6.92 (m, 5H), 6.87 – 6.76 (m, 5H), 6.74 – 6.36 (br s, 3H), 5.98 (d, *J* = 7.2 Hz, 1H), 5.91 (q, *J* = 7.4 Hz, 1H), 5.31 (s, 1H), 4.60 (d, *J* = 7.2 Hz, 1H), 2.38 (d, *J* = 7.4 Hz, 3H), 1.83 – 1.70 (m, 1H), 1.66 – 1.55 (m, 1H), 1.50 (s, 3H), 0.84 (t, *J* = 7.3 Hz, 3H), 0.41 (s, 3H). DEPT-Q ^{13}C NMR (101 MHz, $CDCl_3$) δ 202.8 (C), 160.9 (C), 141.3 (C), 139.4 (CH), 139.2 (C), 135.5 (C), 134.0 (C), 127.8 (CH), 127.7 (CH), 126.8 (CH), 126.6

(CH), 126.2 (CH), 83.0 (CH), 72.0 (CH), 63.5 (CH), 50.4 (C), 49.7 (C), 29.6 (CH_2), 19.2 (CH_3), 19.1 (CH_3), 15.1 (CH_3), 9.7 (CH_3); HRMS calcd. for $C_{32}H_{34}NO_3$ $(M+H)^+$: 480.2533, found 480.2512

(*E*)-**24**

R_f = 0.24 (10% EtOAc in PS); ^{1}H NMR (400 MHz, $CDCl_3$) δ 7.44 – 7.29 (m, 1H), 7.16 (q, *J* = 7.7 Hz, 1H), 7.22 – 7.10 (m, 1H), 7.06 – 6.91 (m, 5H), 6.83 – 6.75 (m, 5H), 6.71 – 6.43 (br, 3H), 5.98 (d, *J* = 7.1 Hz, 1H), 5.36 (s, 1H), 4.58 (d, *J* = 7.1 Hz, 1H), 1.95 – 1.84 (m, 4H), 1.73 – 1.65 (m, 1H), 1.52 (s, 3H), 0.98 (t, *J* = 7.4 Hz, 3H), 0.67 (s, 3H). ^{13}C DEPT-Q NMR (101 MHz, $CDCl_3$) δ 201.2 (C), 161.0 (C), 141.2 (C), 140.6 (C), 136.6 (CH), 135.5 (C), 134.0 (C), 127.8 (CH), 127.7 (CH), 126.72 (CH), 126.68 (CH), 126.2 (CH), 83.2 (CH), 71.0 (CH), 63.5 (CH), 50.4 (C), 50.3 (C), 31.6 (CH_2), 19.8 (CH_3), 18.7 (CH_3), 15.4 (CH_3), 10.6 (CH_3); HRMS calcd. for $C_{32}H_{34}NO_3$ $(M+H)^+$: 480.2533, found 480.2504.

25 (4*S*,5*R*)-3-((3*S*,3a*S*,9b*R*)-1-hydroxy-7,9-dimethoxy-3,3a,9b-trimethyl-3-phenyl-3,3a,4,9b-tetrahydrocyclopenta[*c*]chromen-2-yl)-4,5-diphenyloxazolidin-2-one

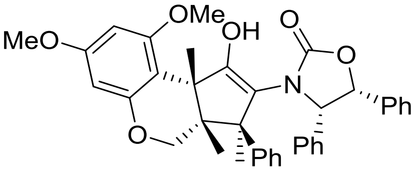

In a dried rbf with stirrer bar **20** (60 mg, 0.10 mmol) was dissolved in anhydrous 1,4-dioxane (1 mL). $MeSO_3H$ (0.065 mL, 0.994 mmol) was added resulting in formation of a white solid which dissolved upon continued stirring at which point the reaction was heated to 50 °C for 24 h. After this time the reaction was cooled to rt and quenched with sat. aq. $NaHCO_3$ (2 mL), diluted with ether (10 mL), the organic layer was separated and aq. layer extracted with Et_2O (2 x 5 mL). The combined organic layers were washed with brine, dried ($MgSO_4$), filtered and concentrated. The crude residue was purified by preparative reverse phase HPLC (60%-100% MeCN in H_2O gradient over 20 minutes, 10 mL min^{-1} , Luna 5µm C8(2) 100 Å 250x21.2 mm column, UV detection at 254 nm, t_R=15.0 min) to give a **25** (32 mg, 0.053 mmol, 53%) containing a small amount of the corresponding keto tautomer. N.B. This product degraded when silica gel flash chromatography was attempted. R_f = 0.14 (20% EtOAc in PS); ^{1}H NMR (401 MHz, $CDCl_3$) δ 8.33 (s, 1H), 7.63 (br s, 2H), 7.27 – 7.25 (m, 2H), 7.20 – 7.14 (m, 2H), 7.03 (d, *J* = 1.1 Hz, 3H), 6.96 (s, 2H), 6.92 (m, 5H), 6.13 (d, *J* = 2.5 Hz, 1H), 6.01 (d, *J* = 2.5 Hz, 1H), 5.80 (d, *J* = 8.7 Hz, 1H), 5.34 (d, *J* = 8.7 Hz, 1H), 3.94 (s, 3H), 3.77 (d, *J* = 10.4 Hz, 1H), 3.72 (s, 3H), 3.36 (d, *J* = 10.4 Hz, 1H), 1.32 (s, 3H), 1.30 (s, 3H), 0.69 (s, 3H). ^{13}C DEPT-Q NMR (101 MHz, $CDCl_3$) δ 159.7 (C), 158.4 (C), 157.9 (C), 157.1 (C), 156.6 (C), 145.7 (C), 135.4 (C), 135.1 (C), 129.3 (CH), 128.4 (CH), 127.9 (CH), 127.8 (CH), 127.8 (CH), 127.6 (CH), 126.5 (CH), 126.4 (CH), 108.6 (C), 107.0 (C), 95.0 (CH), 93.9 (CH), 79.8 (CH), 72.5 (C), 67.6 (CH), 56.2 (CH_3), 55.4 (CH_3), 54.3 (C), 48.3 (C), 46.1 (C), 21.7 (CH_3), 20.7 (CH_3), 15.7 (CH_3); HRMS calcd. for $C_{38}H_{38}NO_6$ $(M+H)^+$: 604.2694, found 604.2711.

1 WO Patent App., PCT/US2009/056555, 2010.

2 A. E. May, P. H. Willoughby and T. R. Hoye, *J. Org. Chem.*, **2008**, *73*, 3292.

3 F. Zhan and G. Liang, *Angew. Chemie Int. Ed.*, **2013**, *52*, 1266.

4 T. Hosoya, K. Sumi, H. Doi, M. Wakao and M. Suzuki, *Org. Biomol. Chem.*, **2006**, *4*, 410.

5 X. Nie and G. Wang, *J. Org. Chem.*, **2006**, *71*, 4734.

6 G. Cahiez, O. Gager and J. Buendia, *Angew. Chemie Int. Ed.*, **2010**, *49*, 1278.

7 J. R. Wolstenhulme, J. Rosenqvist, O. Lozano, J. Ilupeju, N. Wurz, K. M. Engle, G. W. Pidgeon, P. R. Moore, G. Sandford and V. Gouverneur, *Angew. Chemie Int. Ed.*, **2013**, *52*, 9796.

8 D. J. Kerr, M. Miletic, N. Manchala, J. M. White and B. L. Flynn, *Org. Lett.*, **2013**, *15*, 4118.

9 W. Adam and P. Klug, *J. Org. Chem.*, **1993**, *58*, 3416.

10 J. J. Eisch and J. E. Galle, *J. Organomet. Chem.*, **1988**, *341*, 293.

11 Y.-X. Li, K.-G. Ji, H.-X. Wang, S. Ali and Y.-M. Liang, *J. Org. Chem.*, **2011**, *76*, 744.

12 N. E. Campbell and G. M. Sammis, *Angew. Chemie Int. Ed.*, **2014**, *53*, 6228.

13 R. E. Buckles and G. V. Mock, *J. Org. Chem.*, **1950**, *15*, 680.

14 D. P. Curran and S.-C. Kuo, *Tetrahedron*, **1987**, *43*, 5653.

^{1}H, ^{13}C and 2D NMR spectra

(4*S*,5*R*)-4,5-diphenyl-3-(phenylethynyl)oxazolidin-2-one 4a

^{1}H NMR ($CDCl_3$, 400 MHz)

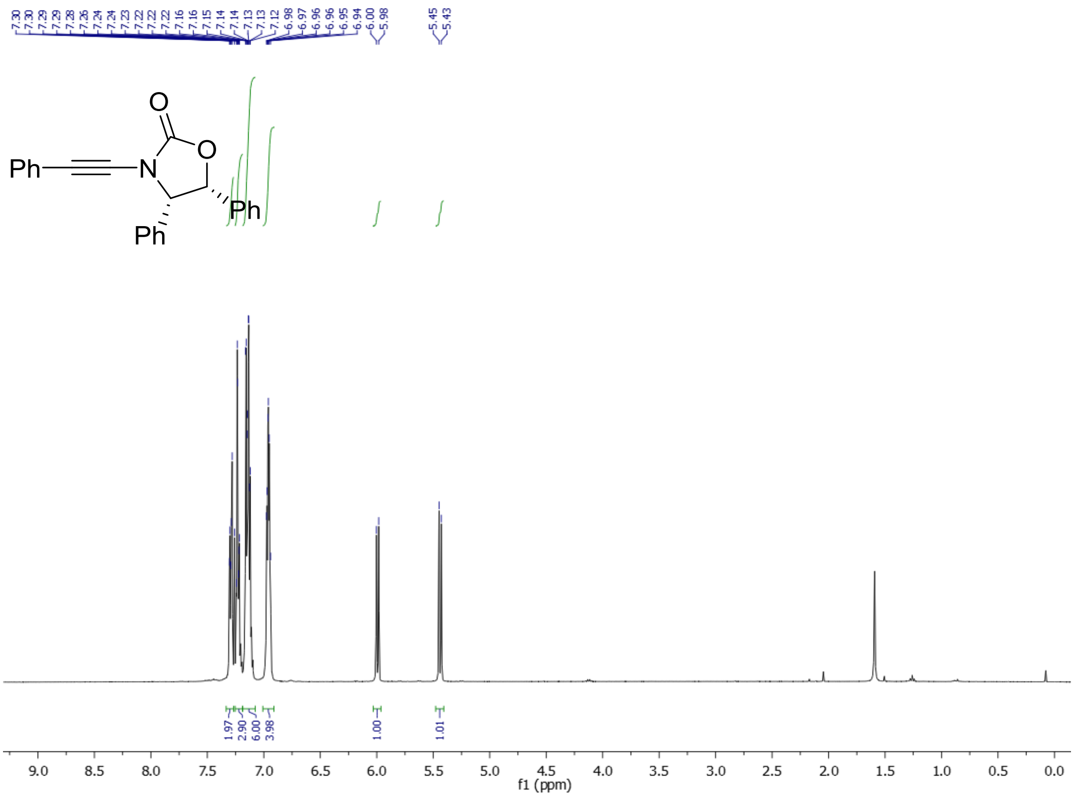

^{13}C NMR ($CDCl_3$, 101 MHz)

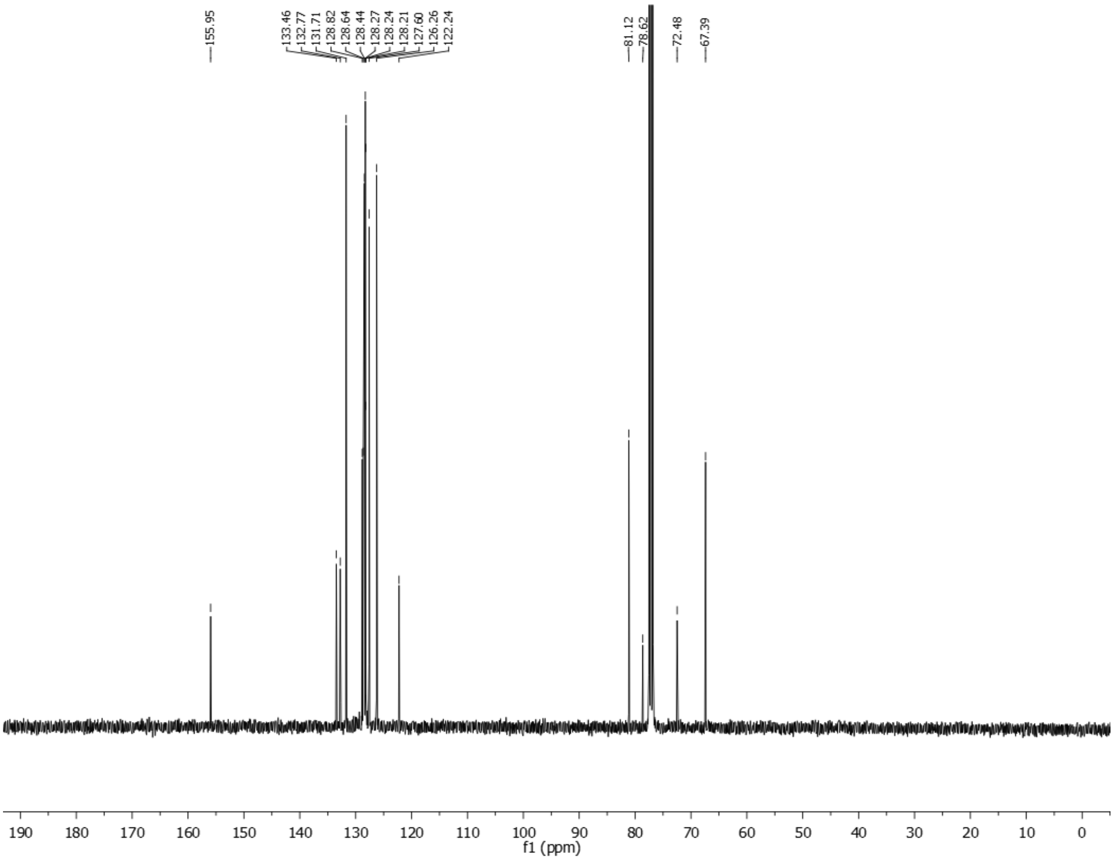

(4*S*,5*R*)-3-(hept-1-yn-1-yl)-4,5-diphenyloxazolidin-2-one 4b

^{1}H NMR ($CDCl_3$, 400 MHz)

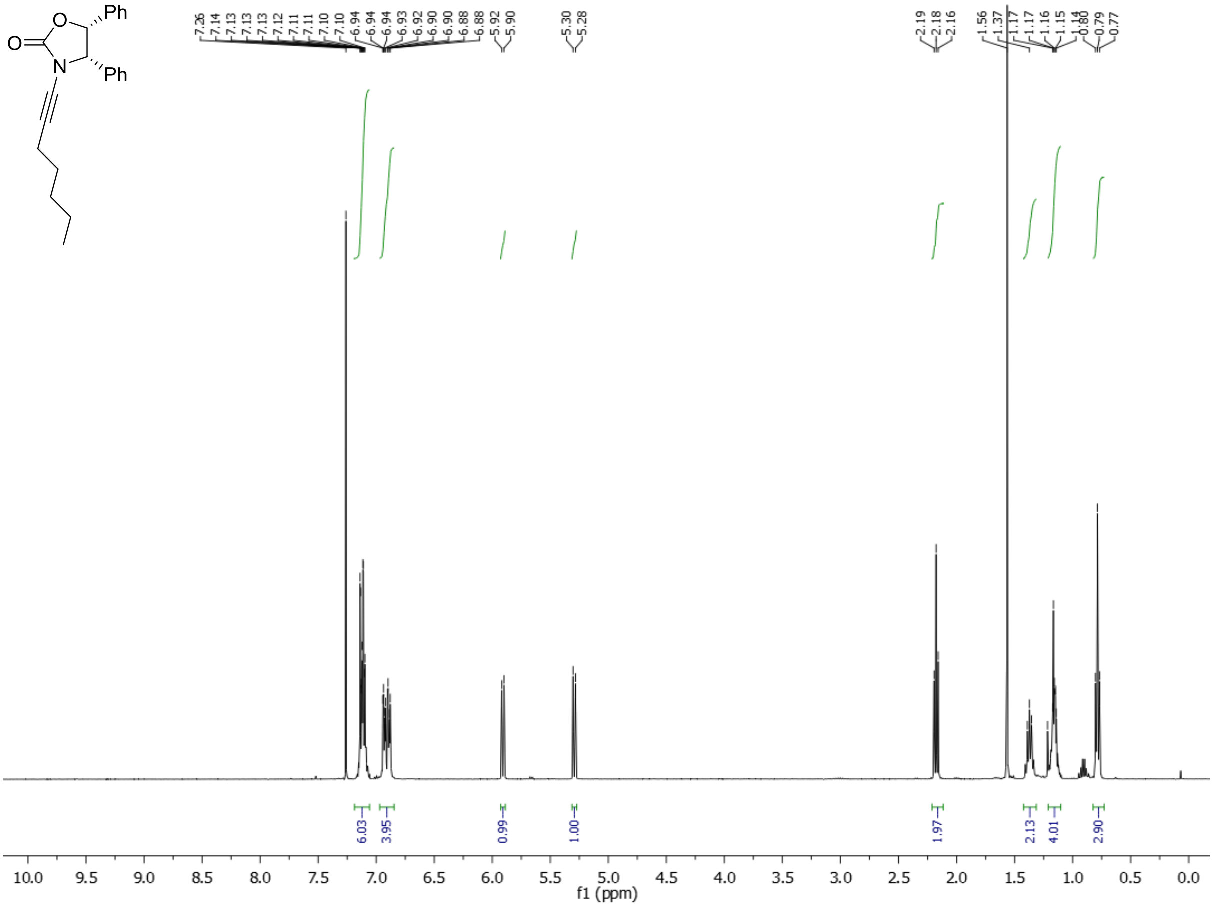

^{13}C NMR ($CDCl_3$, 101 MHz)

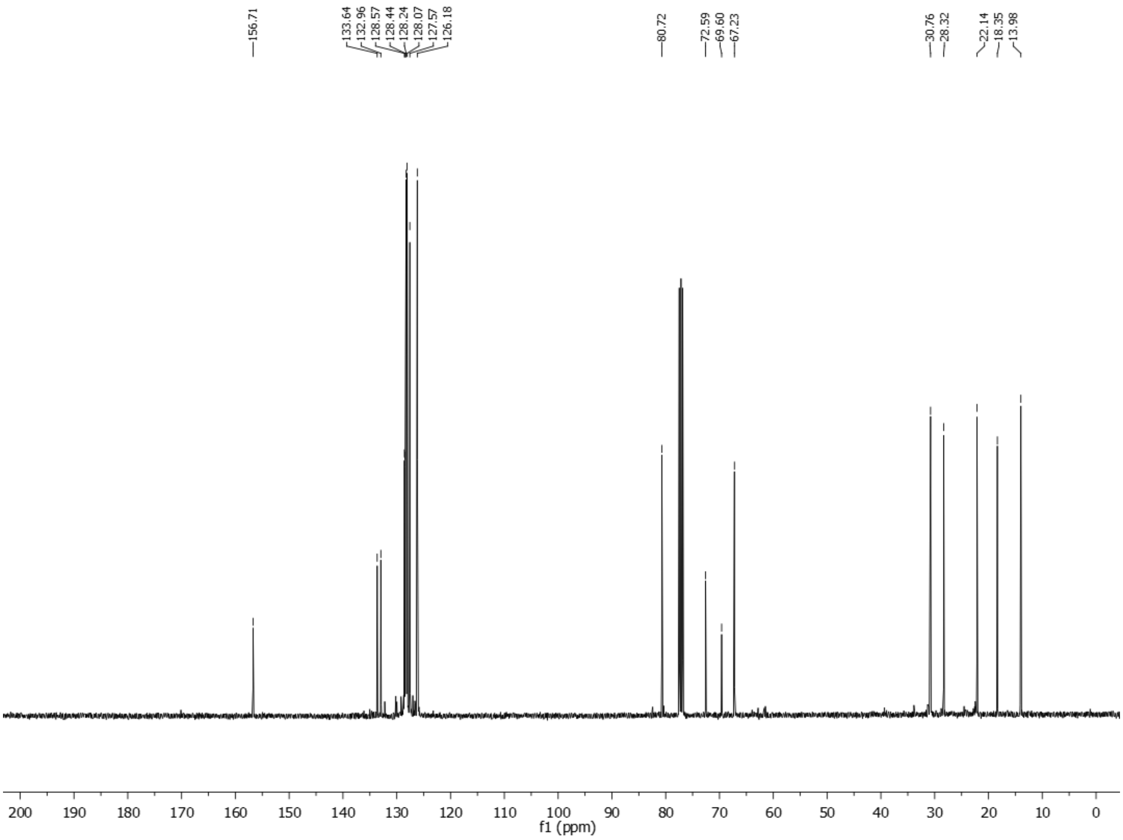

(4*S*,5*R*)-3-((*E*)-1-iodo-2-phenylprop-1-en-1-yl)-4,5-diphenyloxazolidin-2-one 10a

^{1}H NMR ($CDCl_3$, 400 MHz)

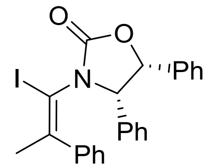

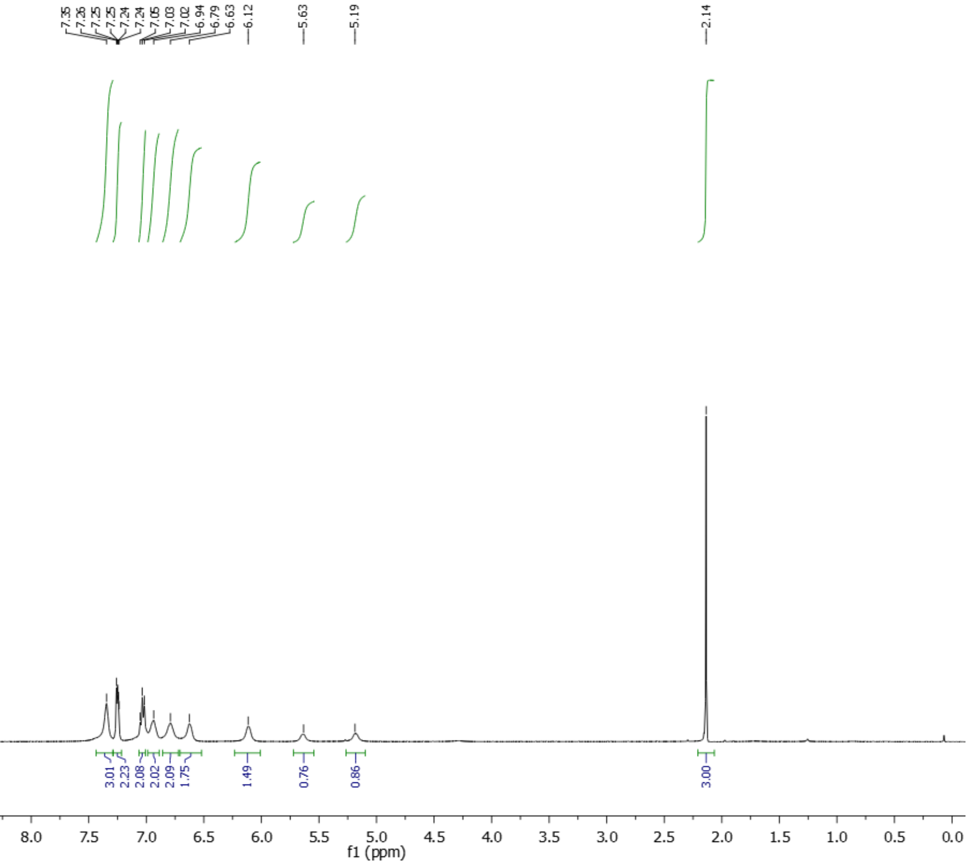

^{13}C NMR ($CDCl_3$, 101 MHz, DEPT-Q)

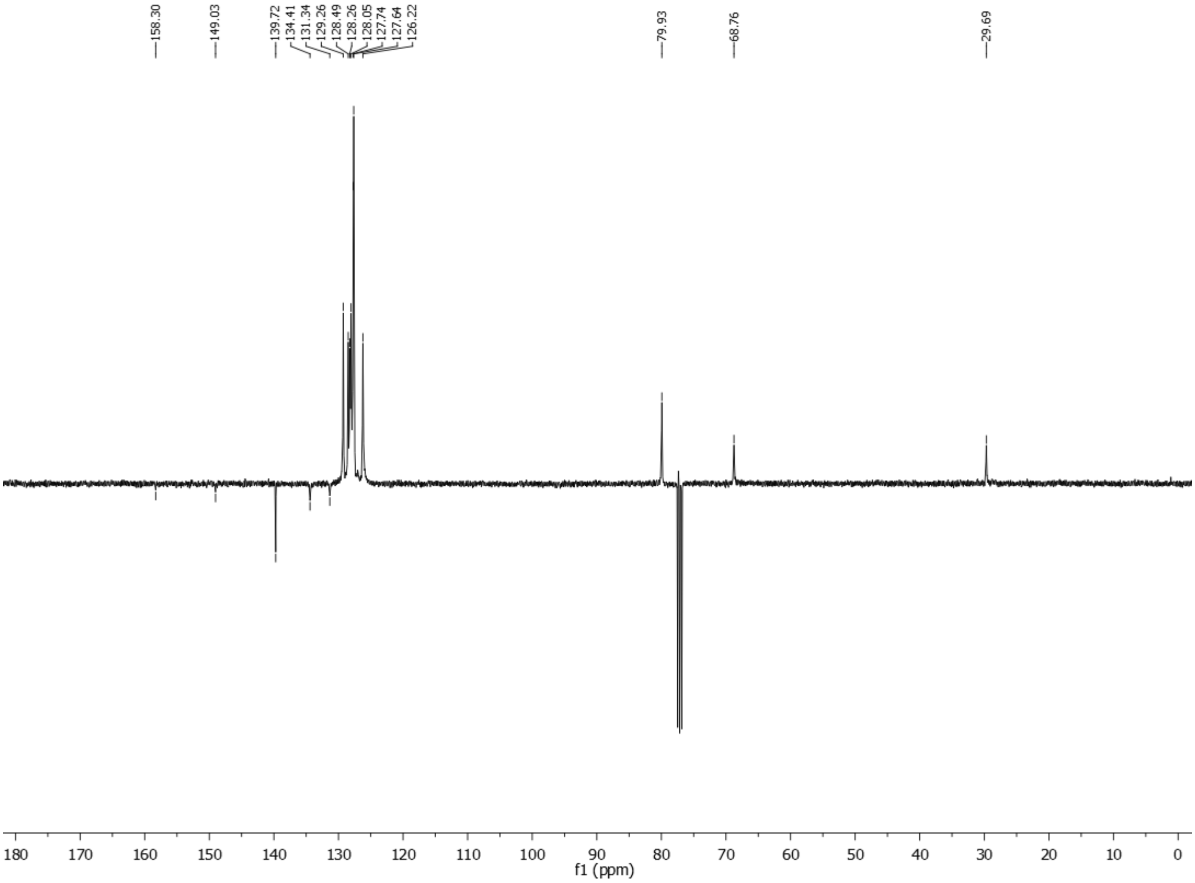

(4*S*,5*R*)-3-((*E*)-1-iodo-2-phenylbut-1-en-1-yl)-4,5-diphenyloxazolidin-2-one 10b

^{1}H NMR ($CDCl_3$, 400 MHz)

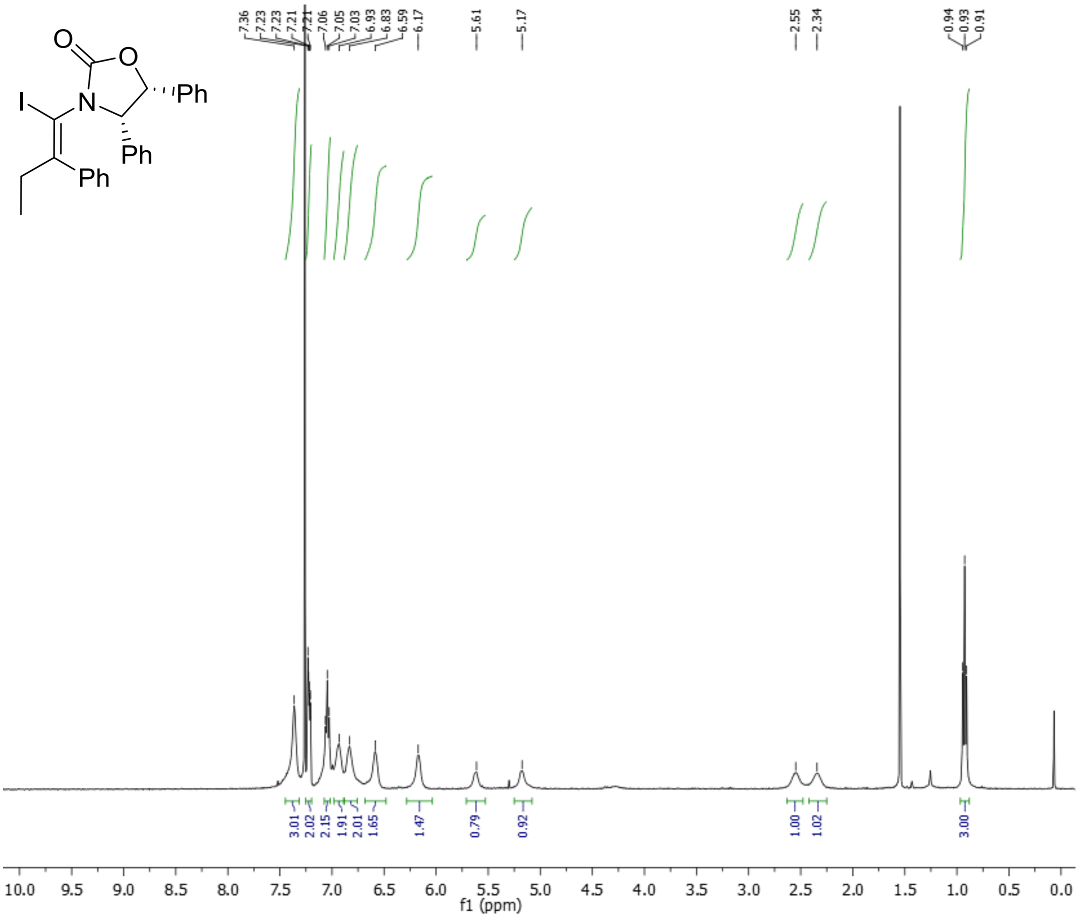

^{13}C NMR ($CDCl_3$, 101 MHz, DEPT-Q)

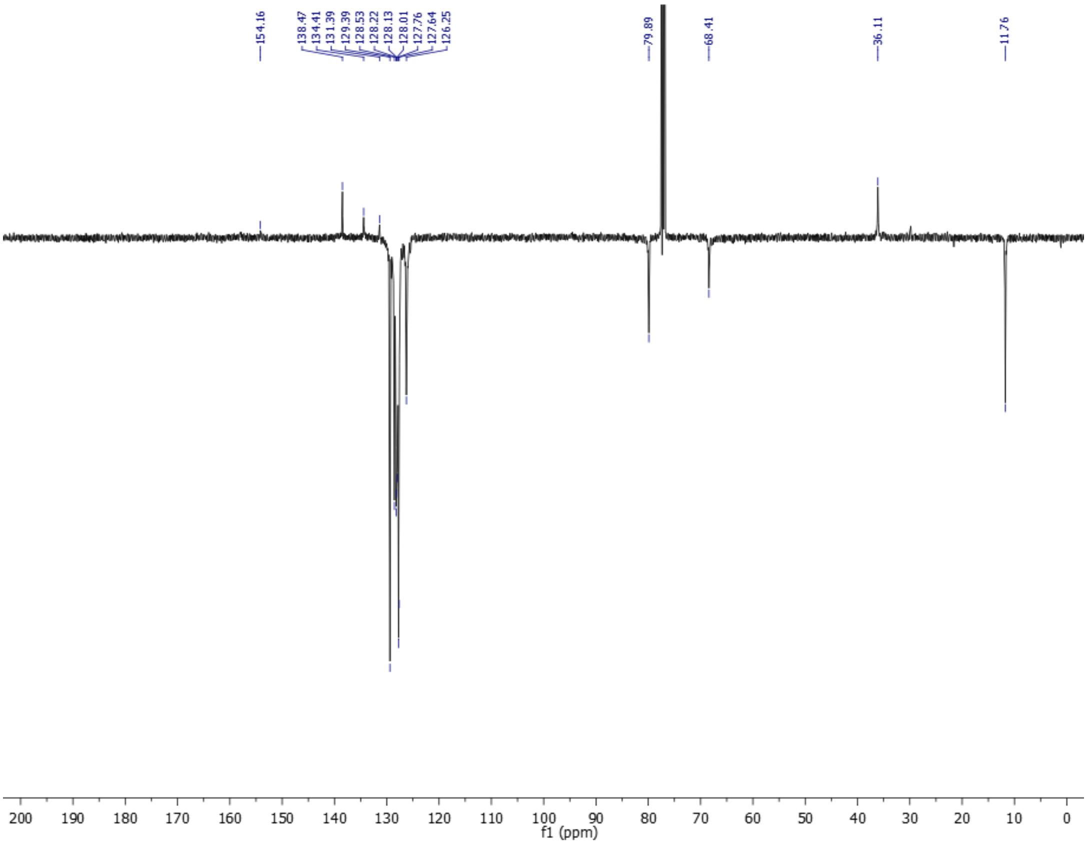

(4*S*,5*R*)-3-((*E*)-1-iodo-2-phenylbut-1-en-1-yl)-4,5-diphenyloxazolidin-2-one 10b

HSQC ($CDCl_3$)

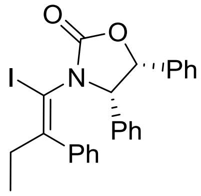

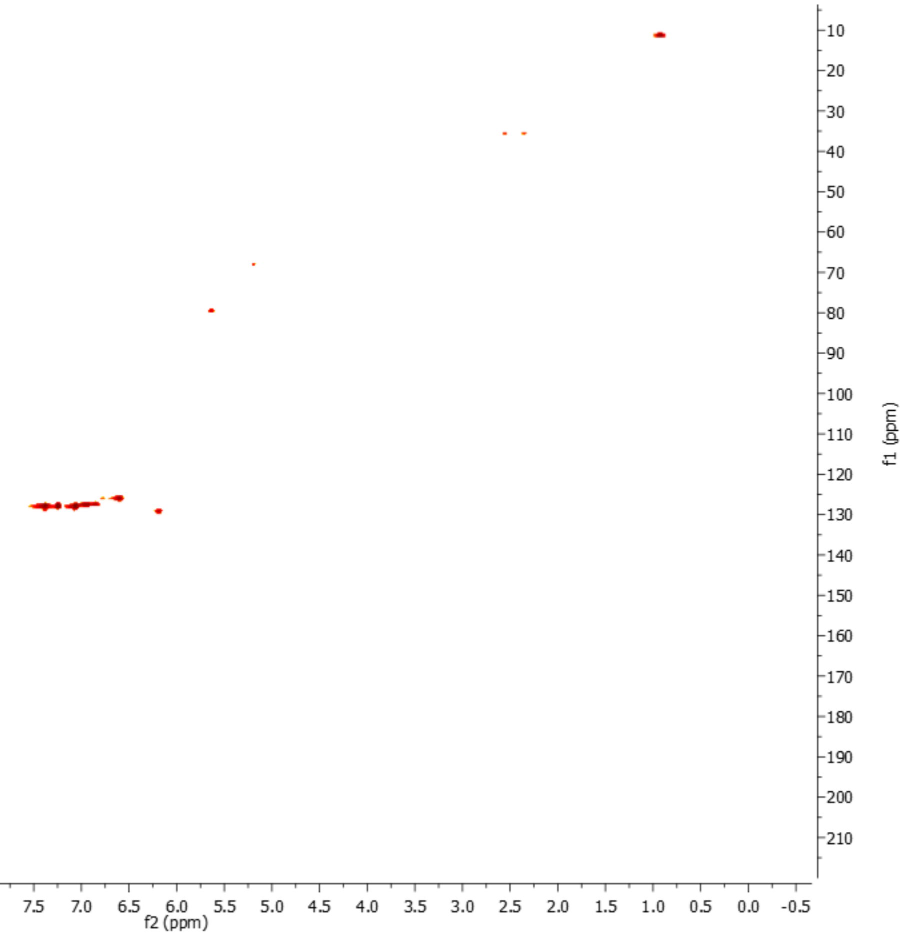

HMBC ($CDCl_3$)

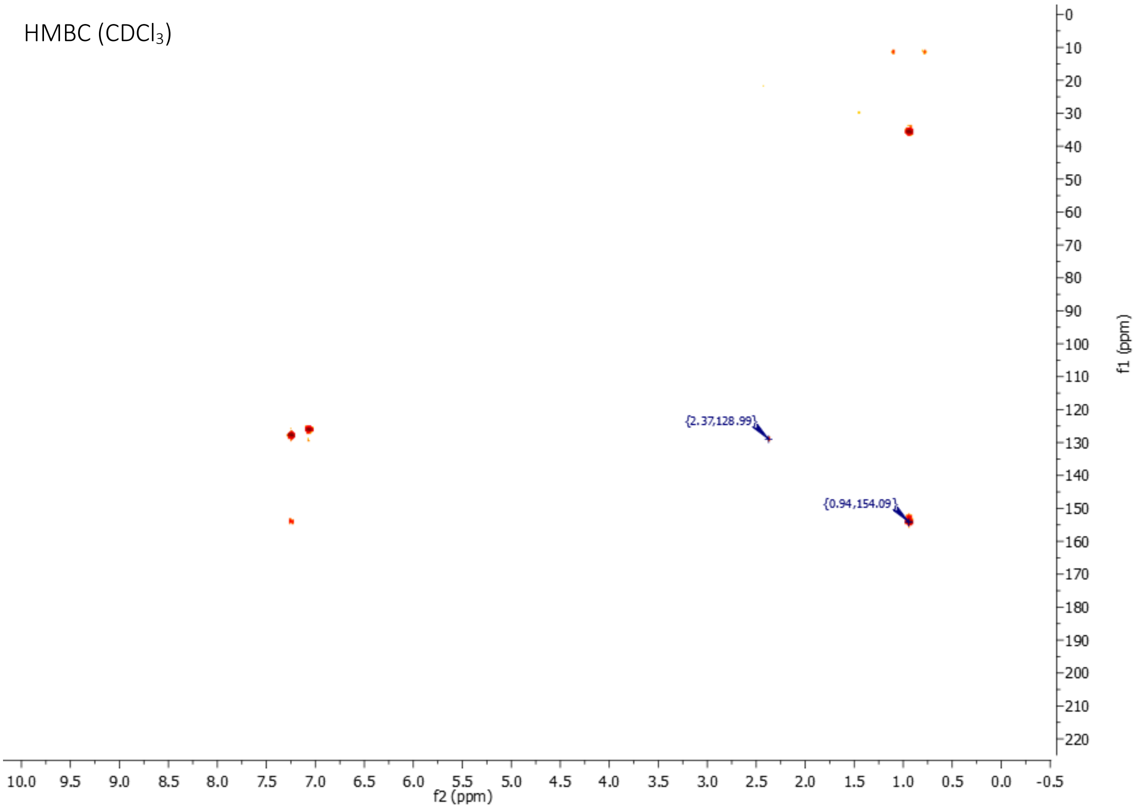

(4*S*,5*R*)-3-((*Z*)-1-(cyclohex-1-en-1-yl)-1-oxo-3-phenylbut-2-en-2-yl)-4,5-diphenyloxazolidin-2-one 5a

^{1}H NMR ($CDCl_3$, 400 MHz)

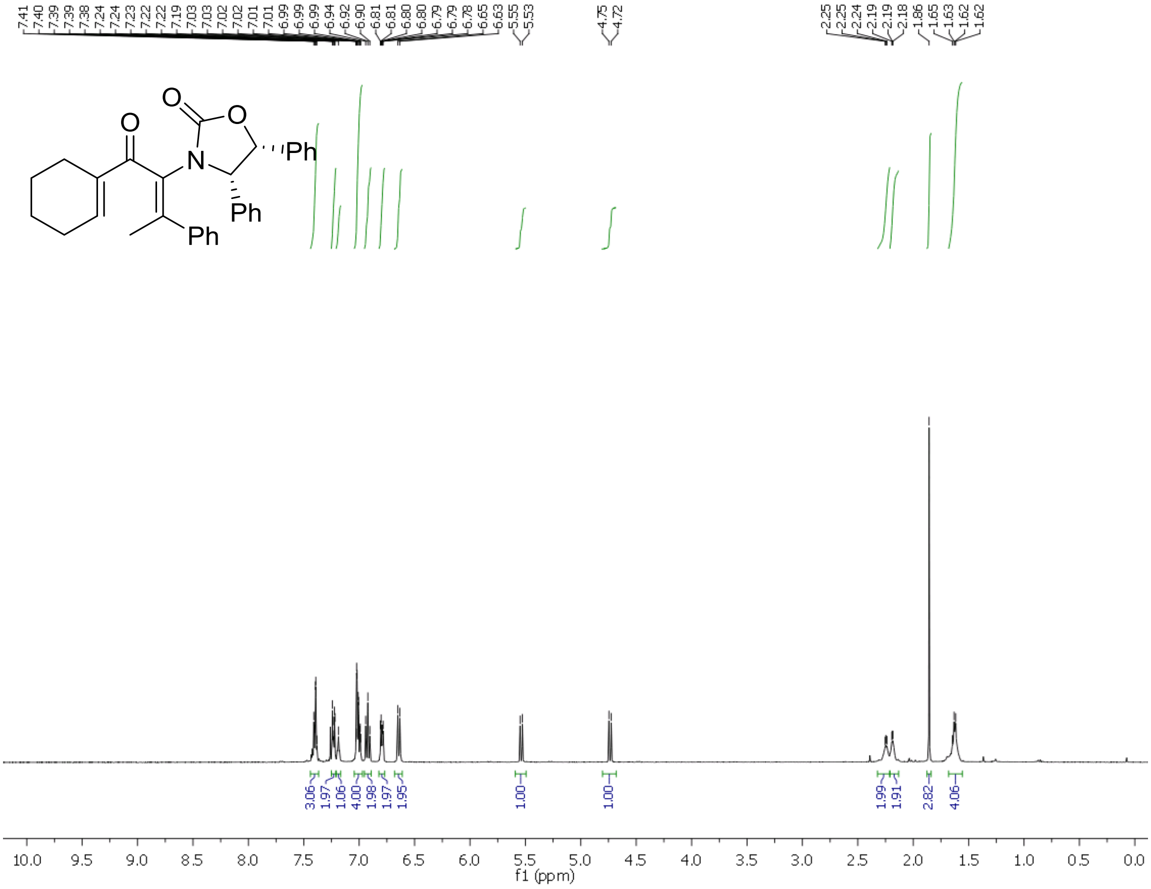

^{13}C NMR ($CDCl_3$, 101 MHz, DEPT-Q)

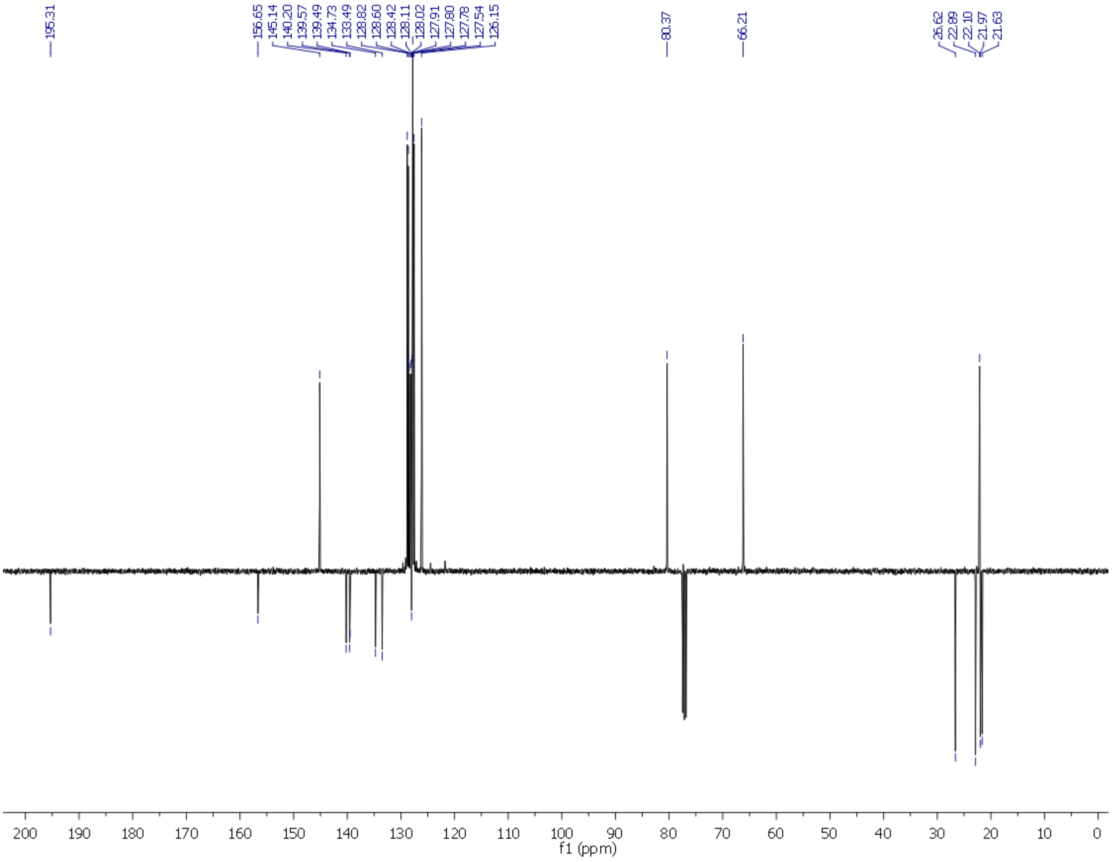

(4*S*,5*R*)-3-((*Z*)-1-(cyclohex-1-en-1-yl)-1-oxo-3-phenylbut-2-en-2-yl)-4,5-diphenyloxazolidin-2-one 5a

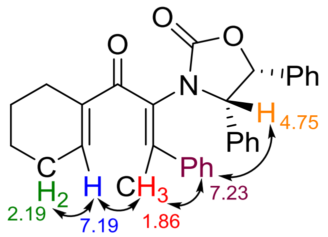

NOESY ($CDCl_3$, 400 MHz)

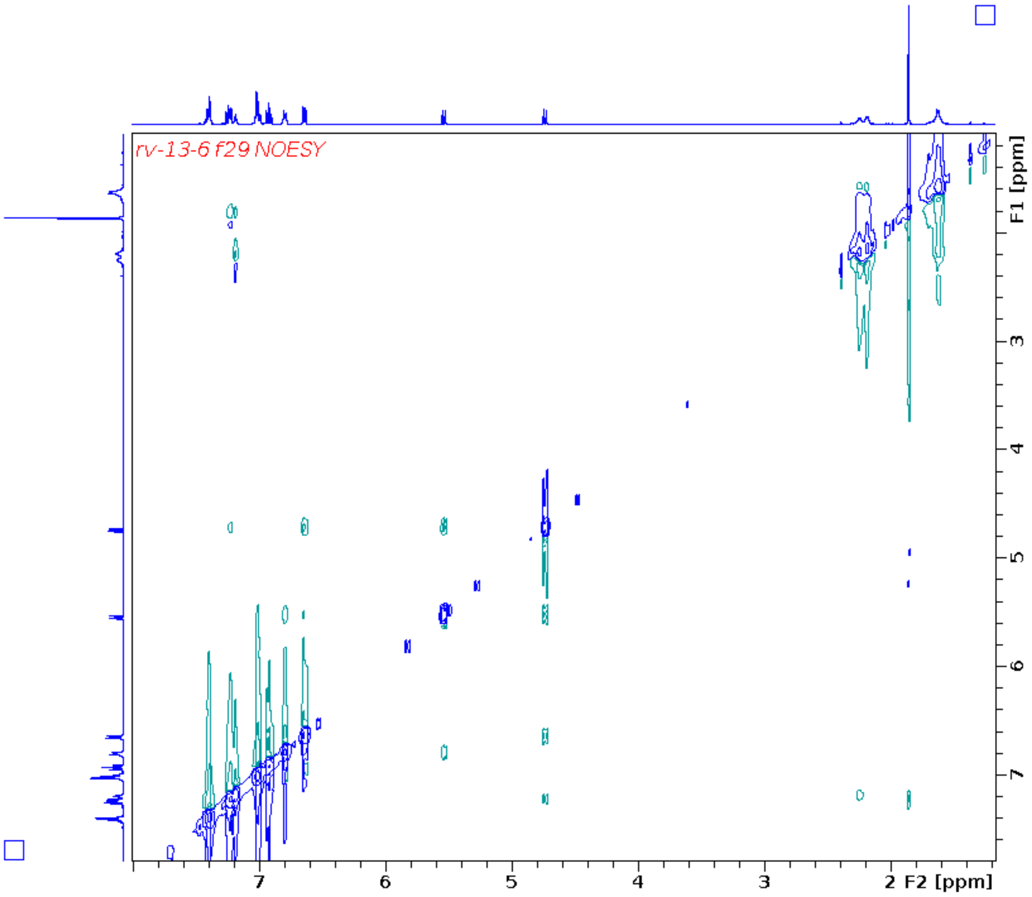

(4*S*,5*R*)-3-((*E*)-1-(cyclohex-1-en-1-yl)-1-oxo-3-phenylbut-2-en-2-yl)-4,5-diphenyloxazolidin-2-one 5a’

^{1}H NMR ($CDCl_3$, 400 MHz)

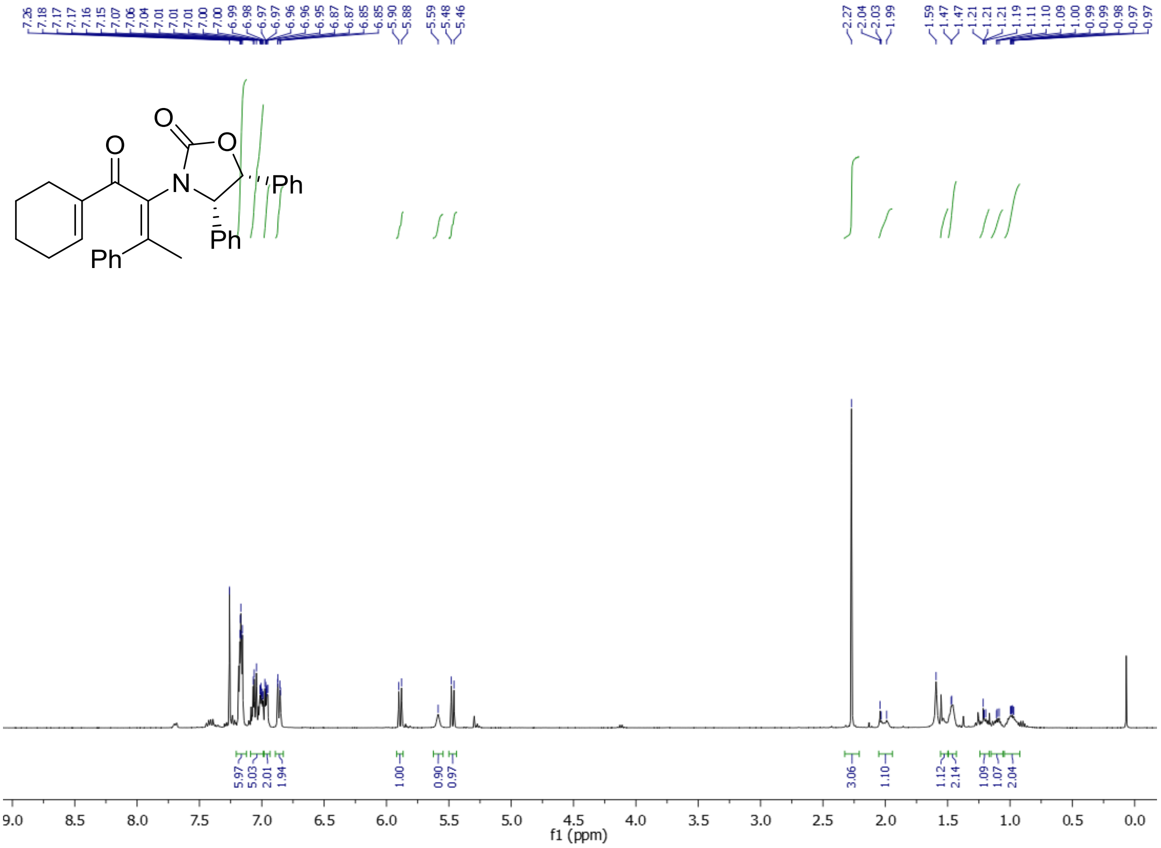

^{13}C NMR ($CDCl_3$, 101 MHz, DEPT-Q)

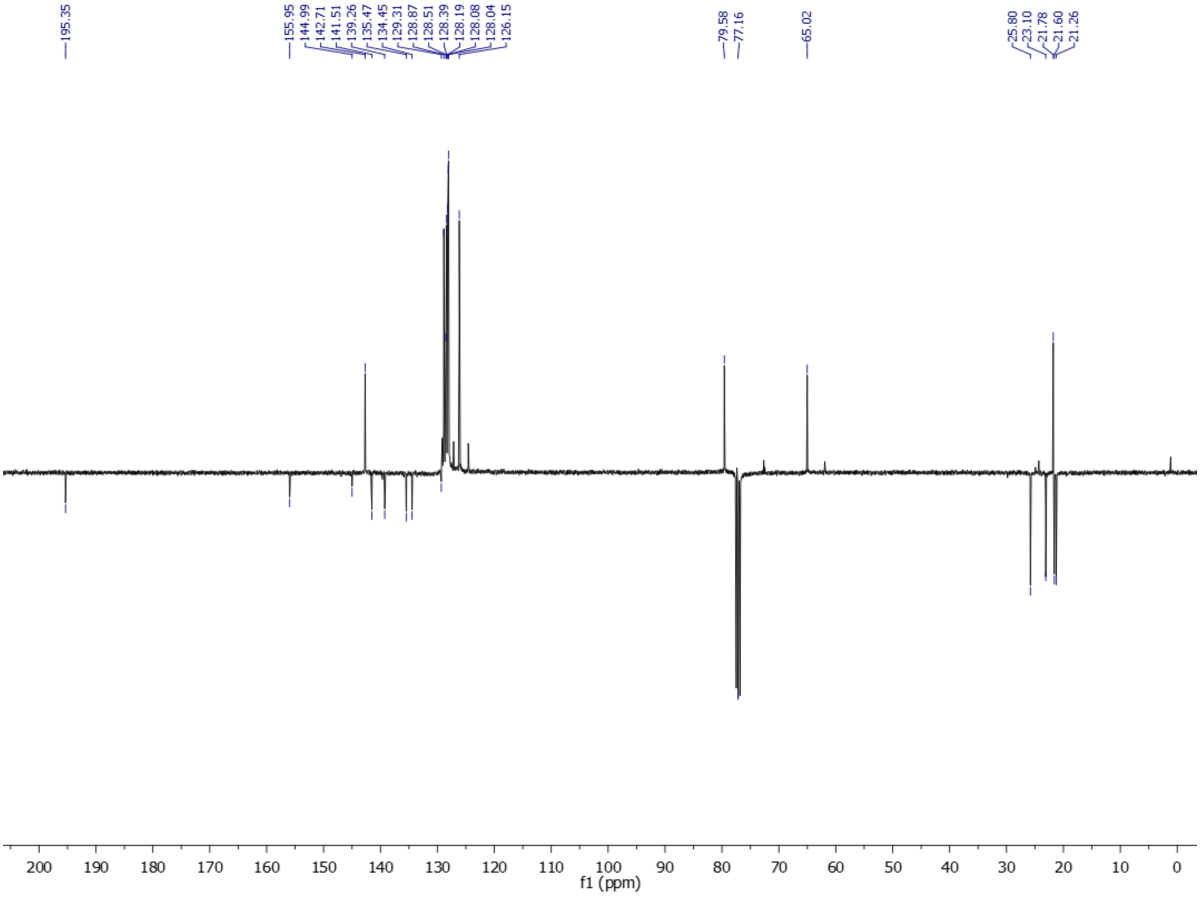

(4*S*,5*R*)-3-((*E*)-1-(cyclohex-1-en-1-yl)-1-oxo-3-phenylbut-2-en-2-yl)-4,5-diphenyloxazolidin-2-one 5a’

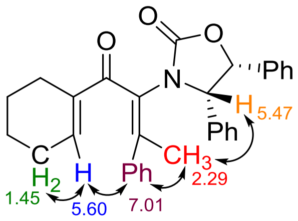

NOESY ($CDCl_3$, 400 MHz)

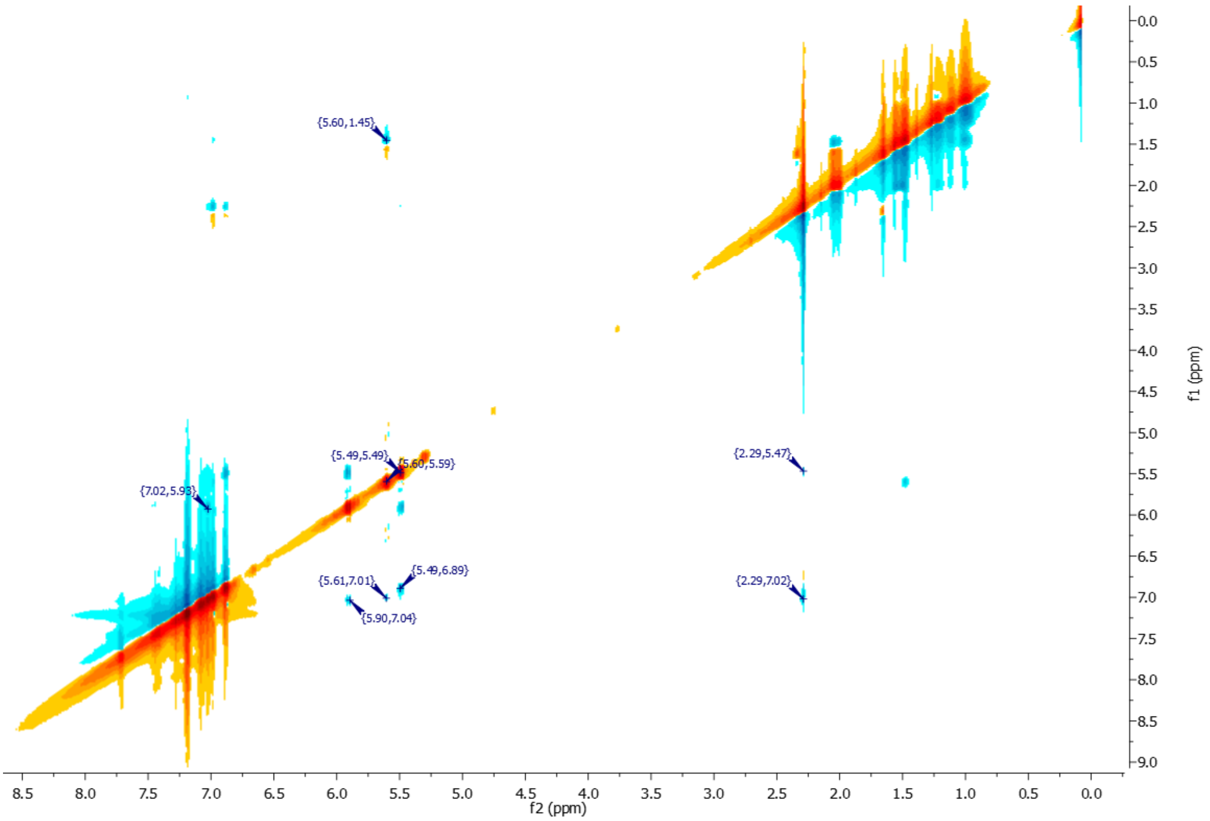

(4*S*,5*R*)-3-((*Z*)-1-(cyclohex-1-en-1-yl)-1-oxo-3-phenylpent-2-en-2-yl)-4,5-diphenyloxazolidin-2-one 5b

^{1}H NMR ($CDCl_3$, 400 MHz)

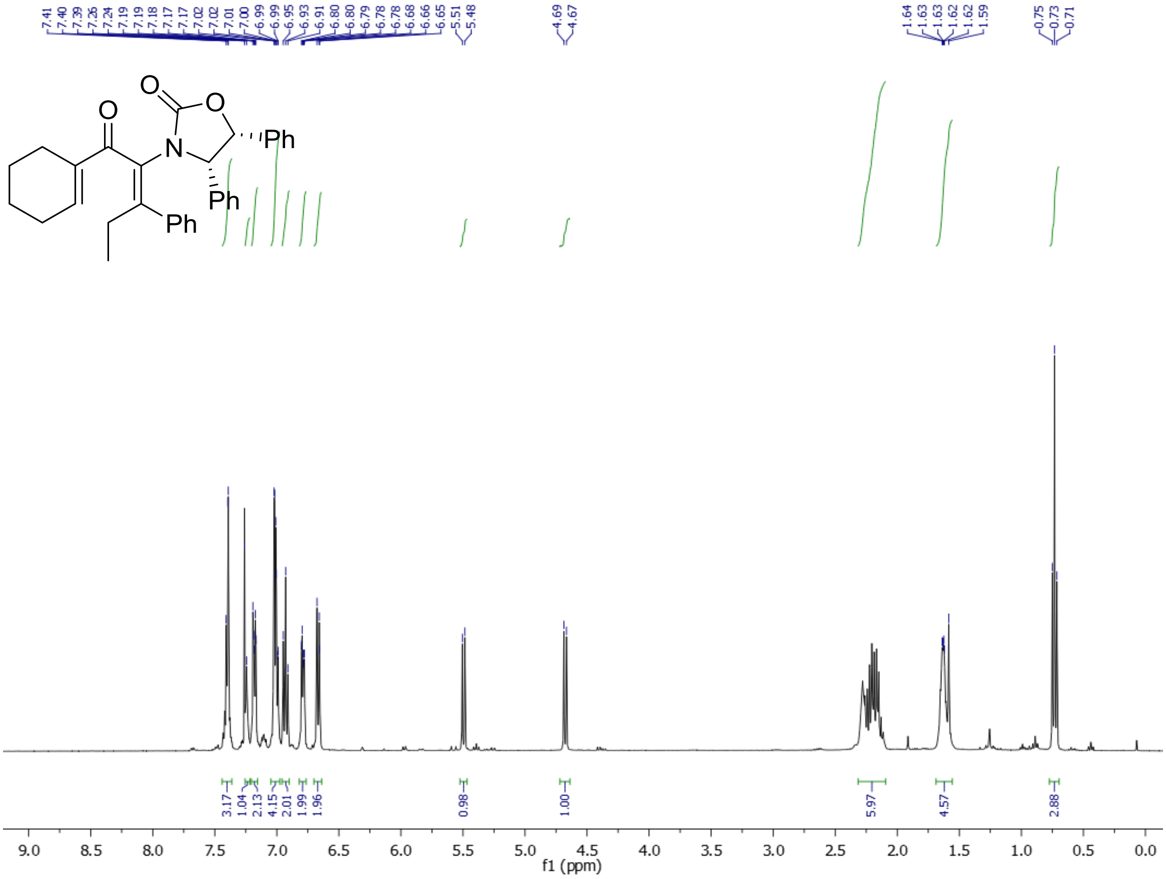

^{13}C NMR ($CDCl_3$, 101 MHz, DEPT-Q)

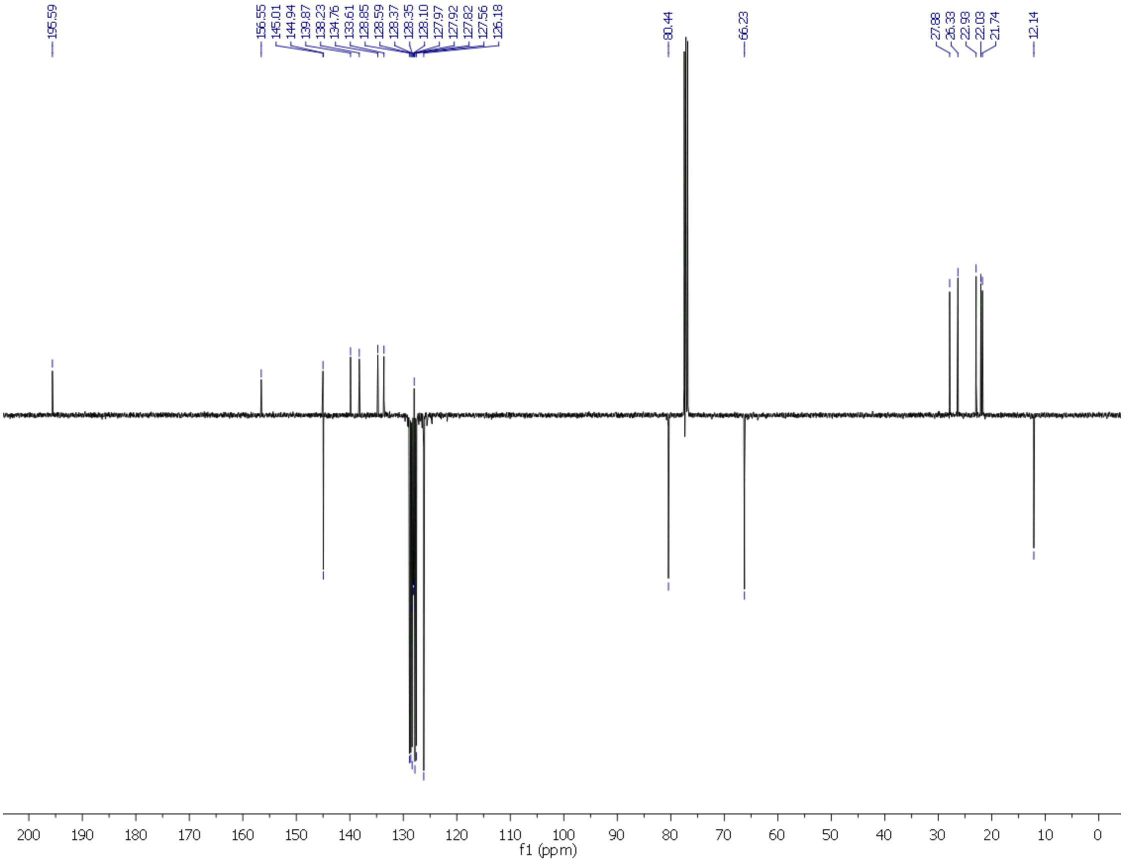

(4*S*,5*R*)-3-((*Z*)-1-(cyclohex-1-en-1-yl)-1-oxo-3-phenylpent-2-en-2-yl)-4,5-diphenyloxazolidin-2-one 5b

NOESY ($CDCl_3$, 400 MHz)

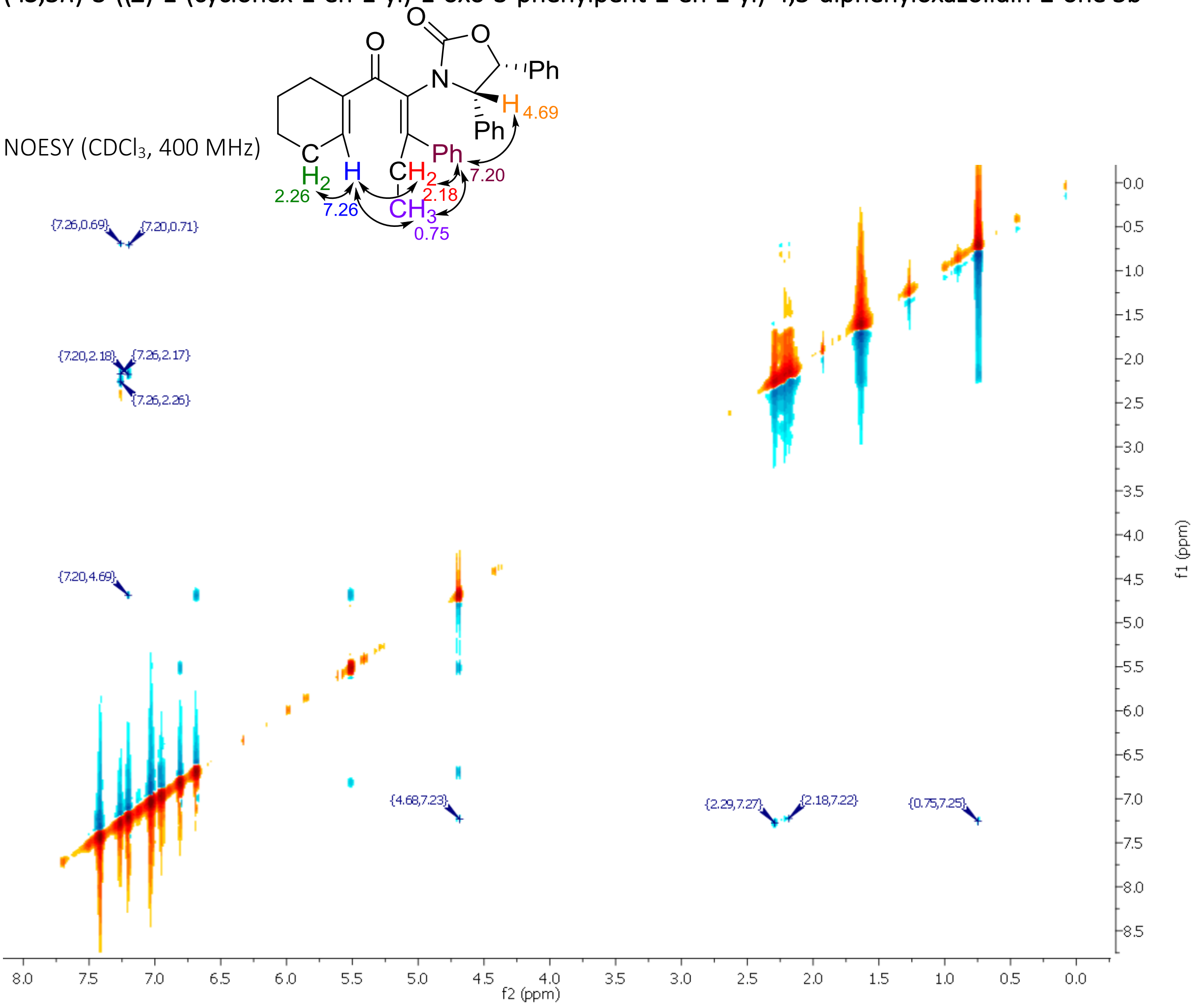

(4*S*,5*R*)-3-((*E*)-1-(cyclohex-1-en-1-yl)-1-oxo-3-phenylpent-2-en-2-yl)-4,5-thiopyloxazolidin-2-one 5b’

^{1}H NMR ($CDCl_3$, 400 MHz)

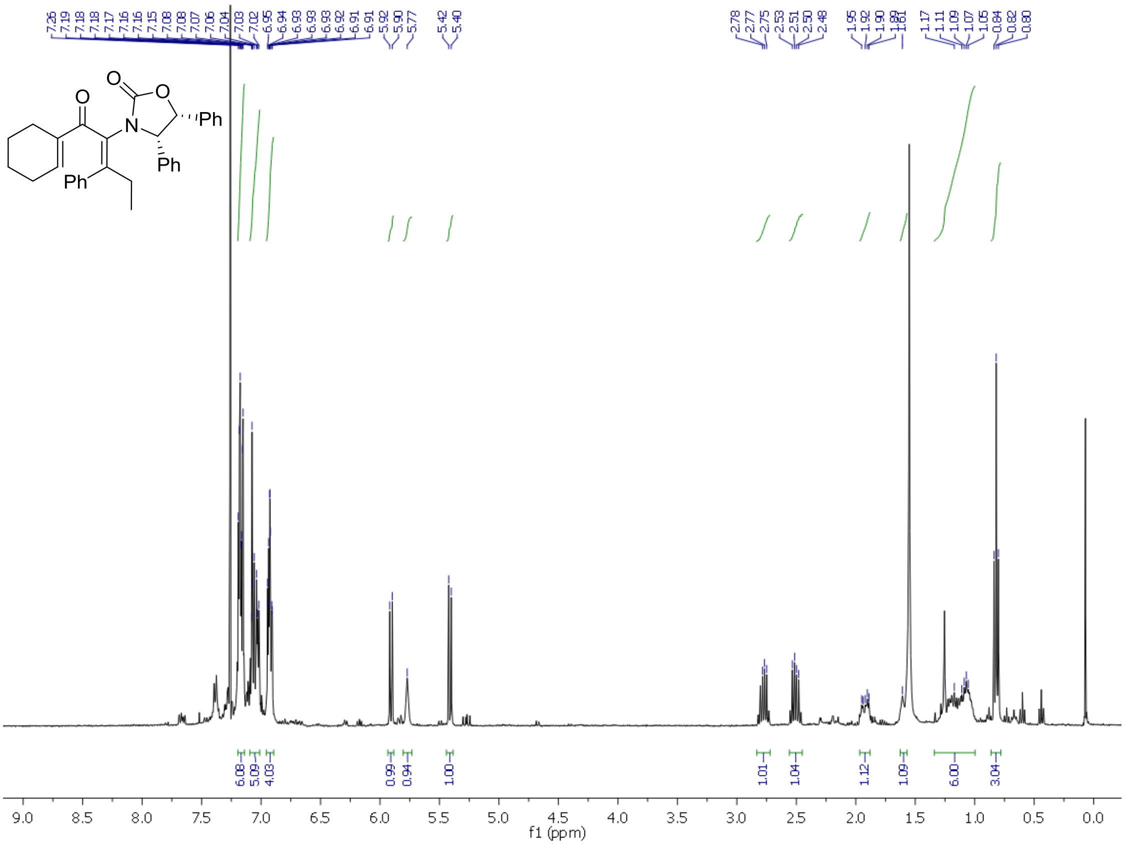

^{13}C NMR ($CDCl_3$, 101 MHz, DEPT-Q)

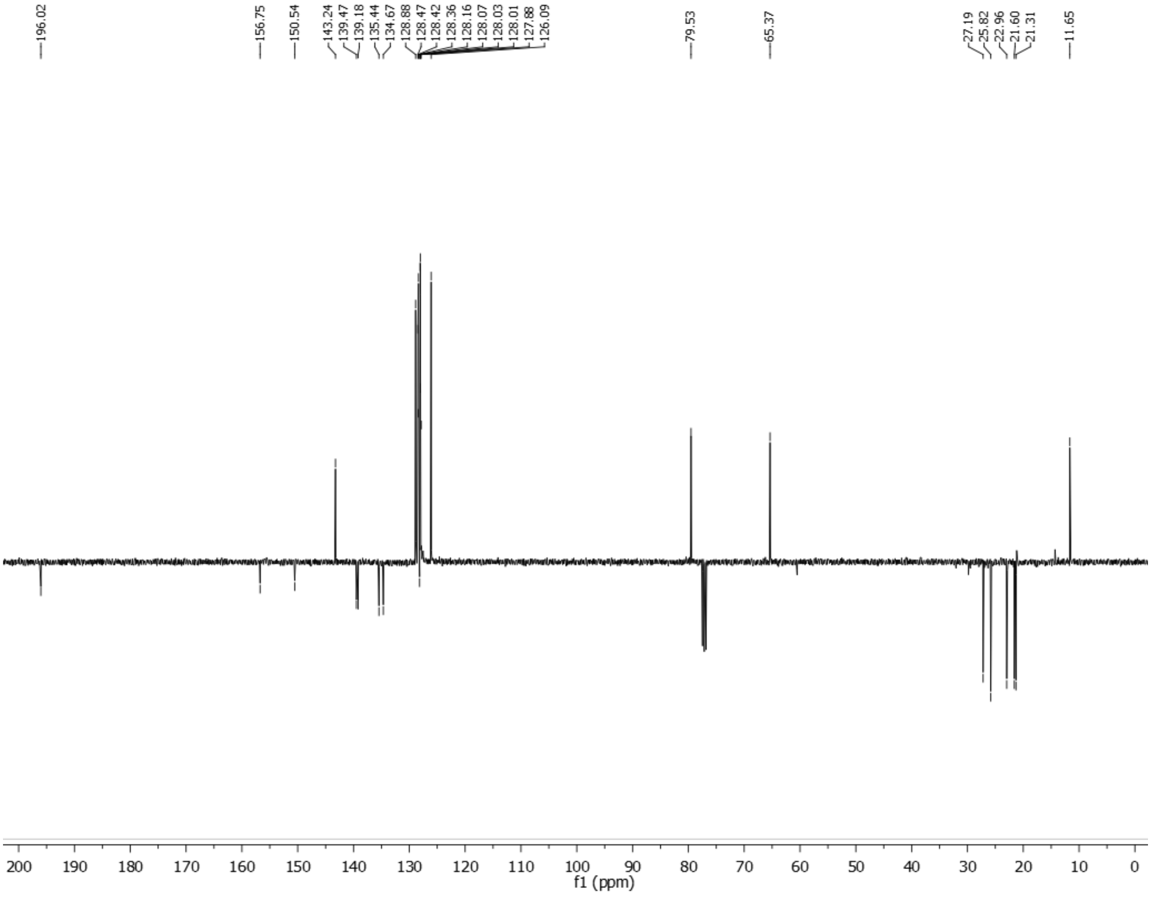

(4*S*,5*R*)-3-((*E*)-1-(cyclohex-1-en-1-yl)-1-oxo-3-phenylpent-2-en-2-yl)-4,5-thiopyloxazolidin-2-one 5b’

NOESY ($CDCl_3$, 400 MHz)

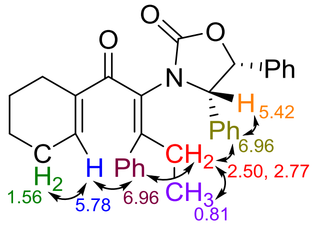

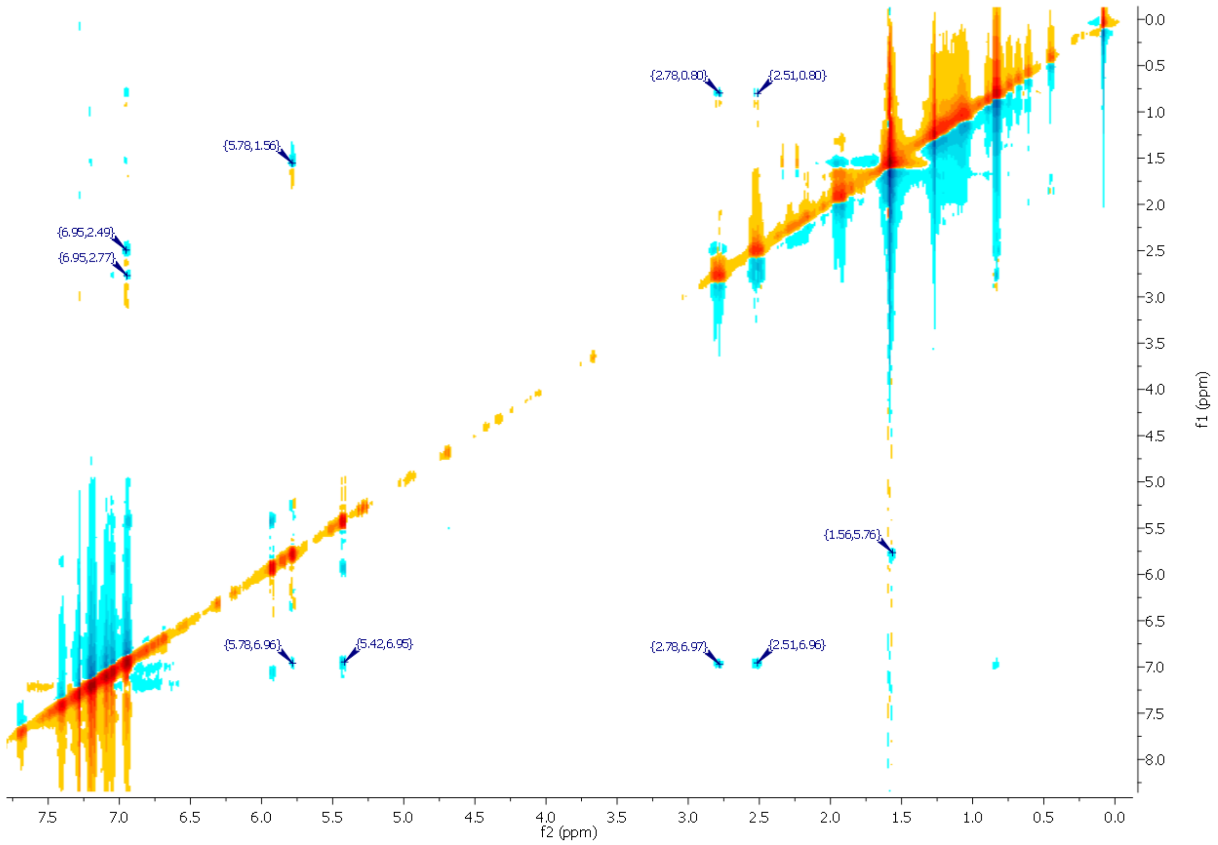

(4*S*,5*R*)-3-((*Z*)-1-(3,4-dihydronaphthalen-2-yl)-1-oxo-3-phenylbut-2-en-2-yl)-4,5-diphenyloxazolidin-2-one 5c

^{1}H NMR ($CDCl_3$, 400 MHz)

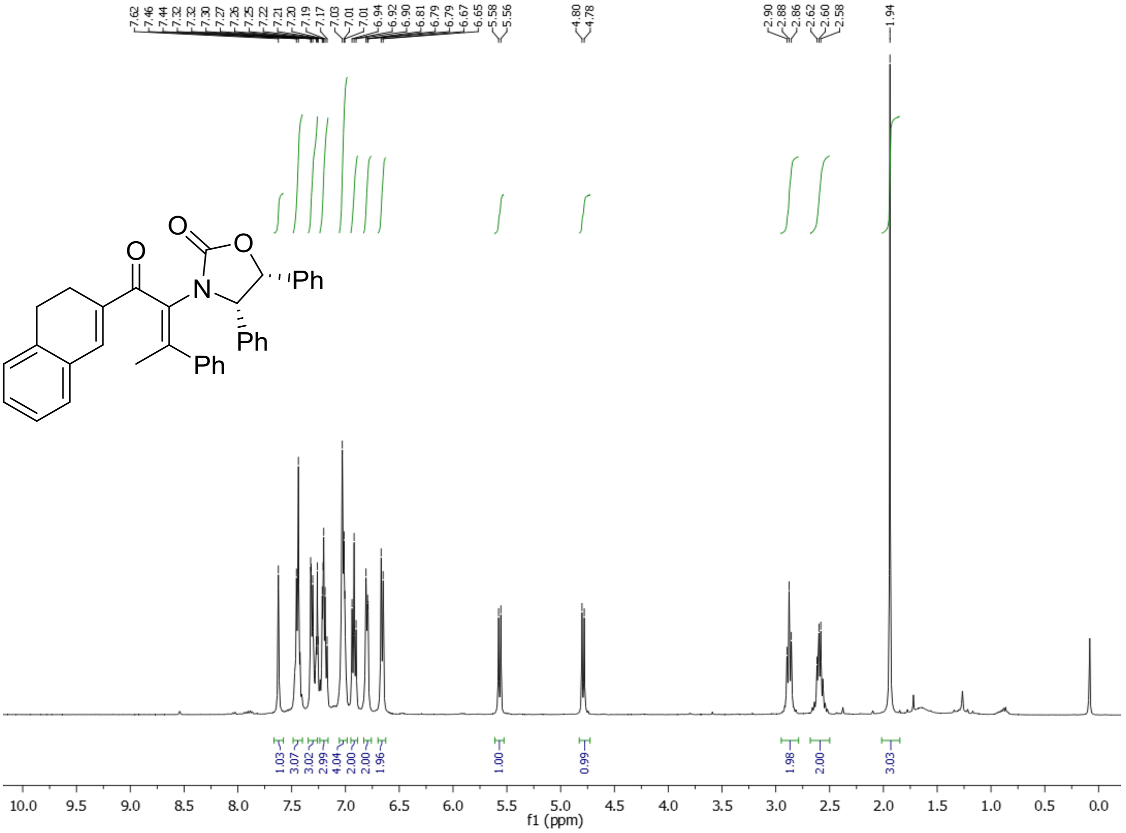

^{13}C NMR ($CDCl_3$, 101 MHz, DEPT-Q)

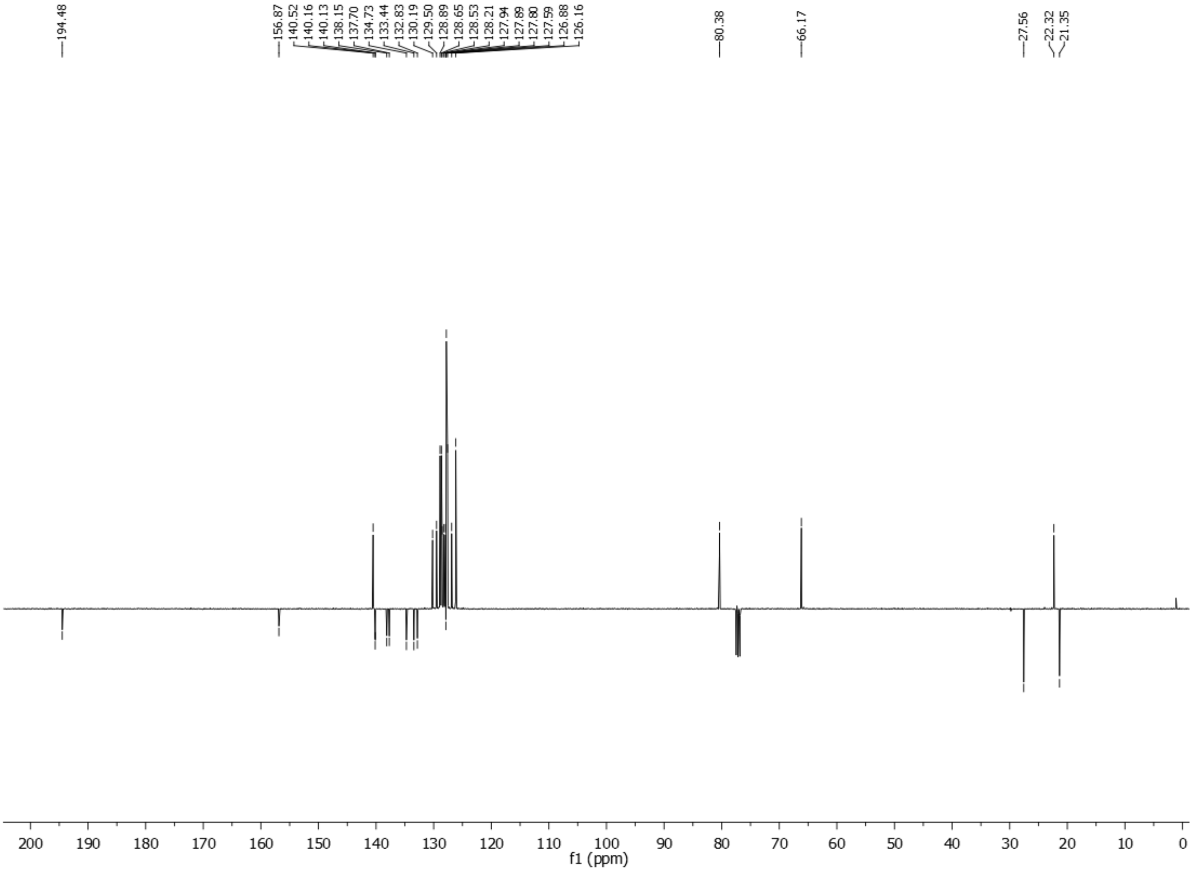

(4*S*,5*R*)-3-((*Z*)-1-(3,4-dihydronaphthalen-2-yl)-1-oxo-3-phenylbut-2-en-2-yl)-4,5-diphenyloxazolidin-2-one 5c

NOESY ($CDCl_3$, 400 MHz)

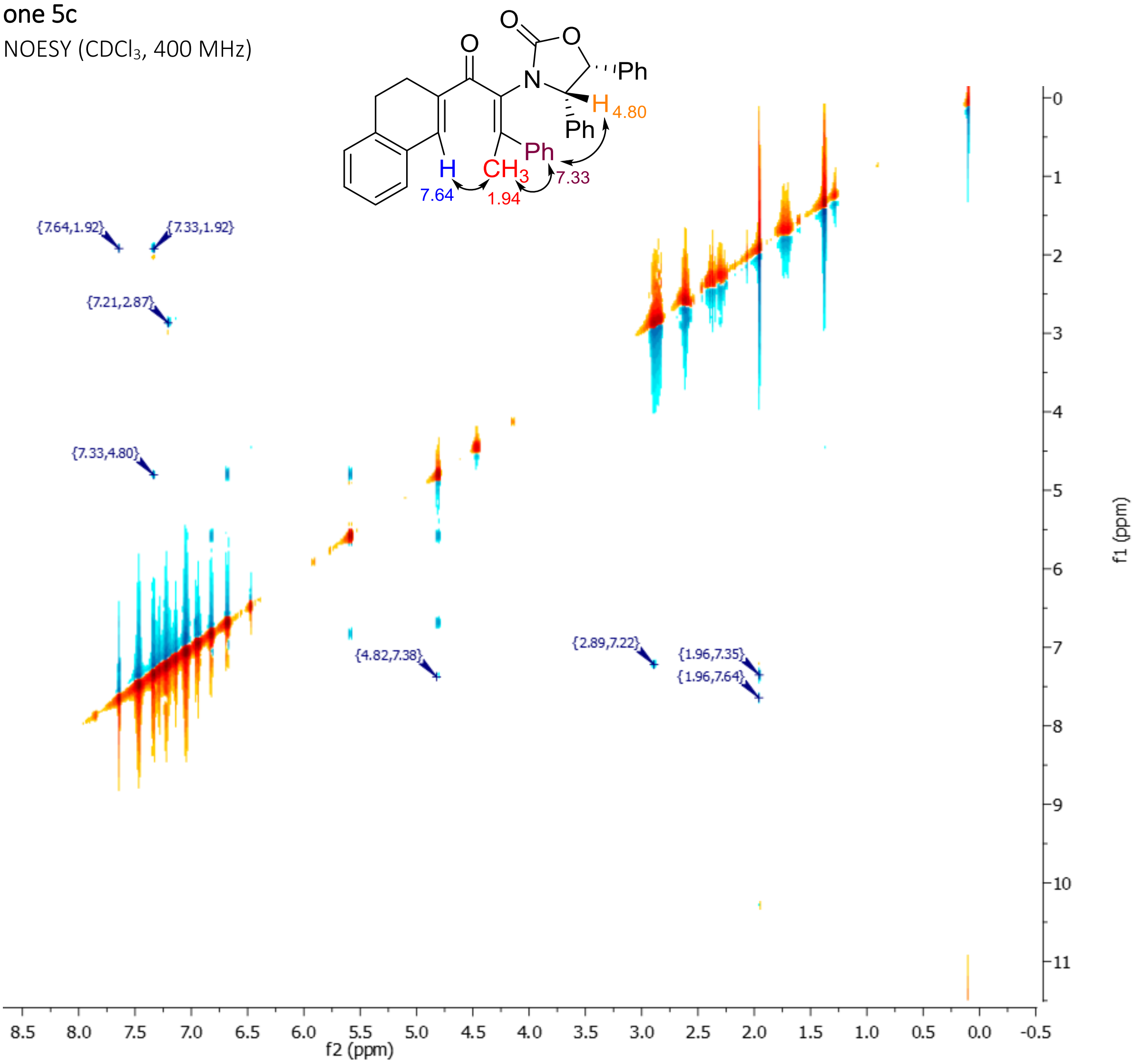

(4*S*,5*R*)-3-((*Z*)-1-(3,4-dihydronaphthalen-2-yl)-4-methyl-1-oxo-3-phenylpent-2-en-2-yl)-4,5-diphenyloxazolidin-2-one 5d

^{1}H NMR ($CDCl_3$, 400 MHz)

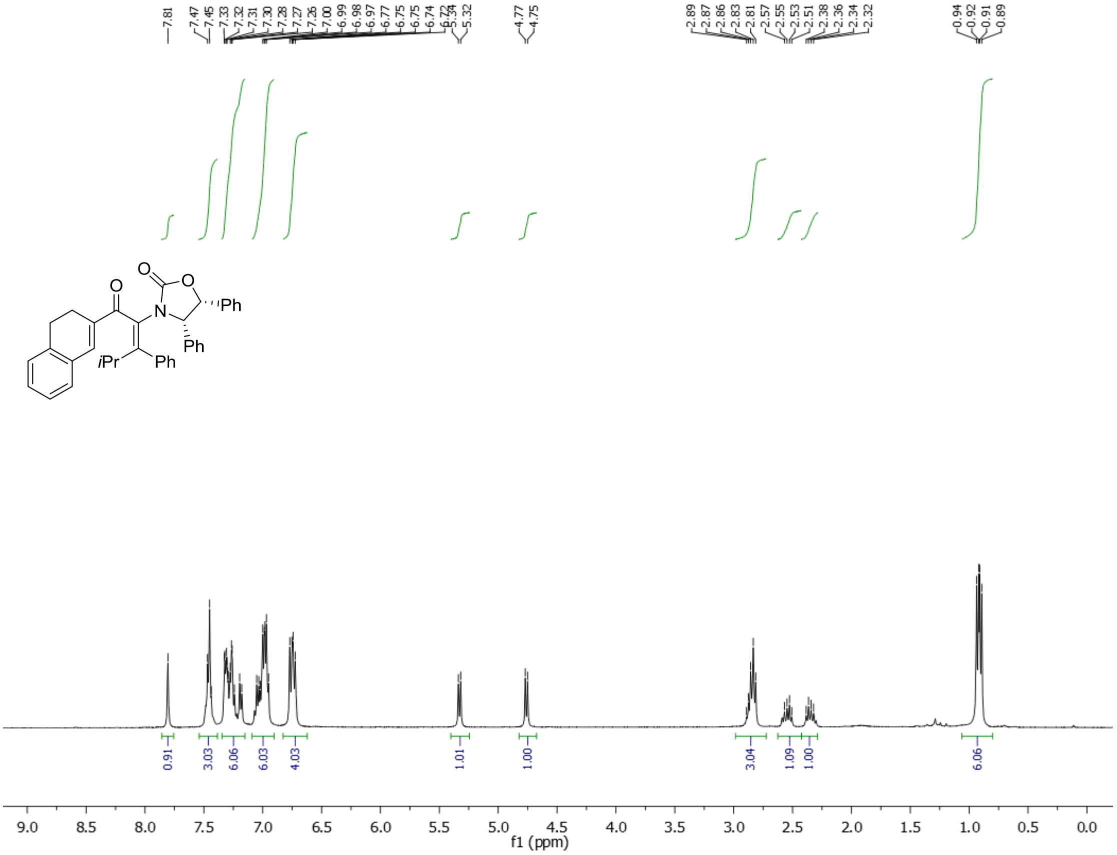

^{13}C NMR ($CDCl_3$, 101 MHz, DEPT-Q)

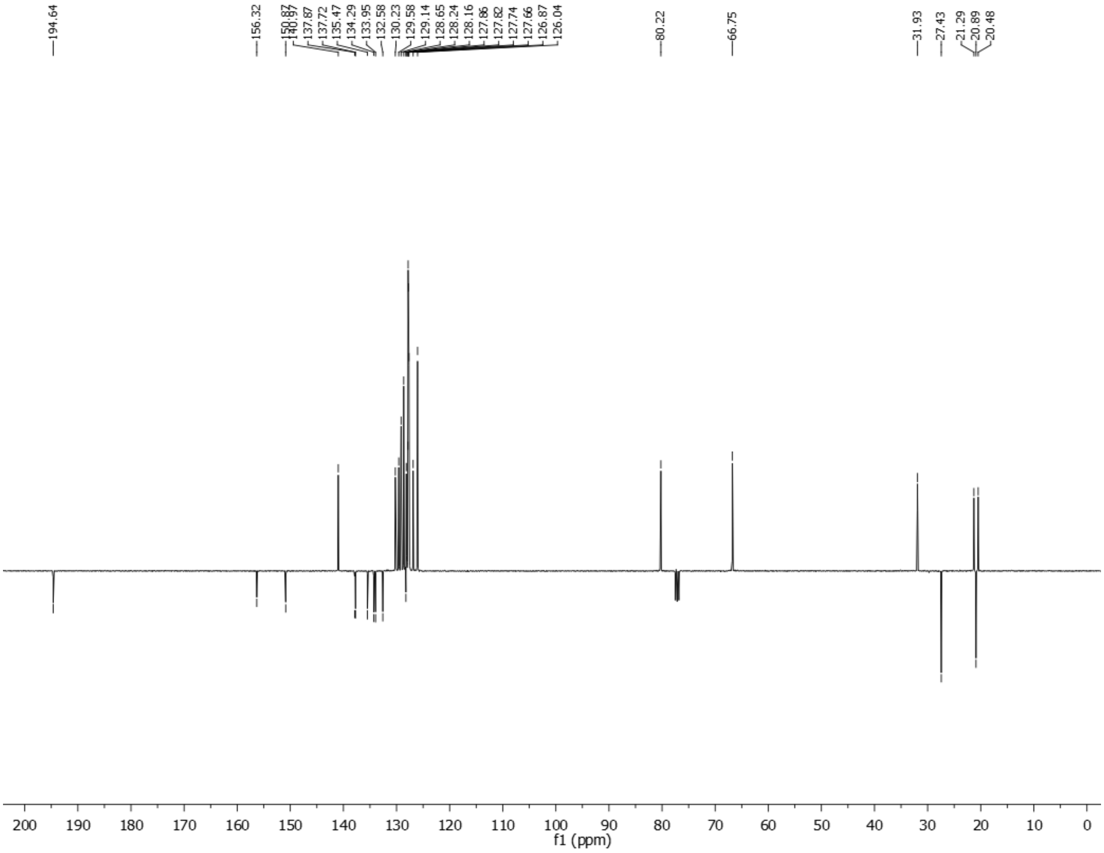

(4*S*,5*R*)-3-((*Z*)-1-(3,4-dihydronaphthalen-2-yl)-3-methyl-1-oxooct-2-en-2-yl)-4,5-diphenyloxazolidin-2-one 5e

^{1}H NMR (CDCl$_3$, 400 MHz)

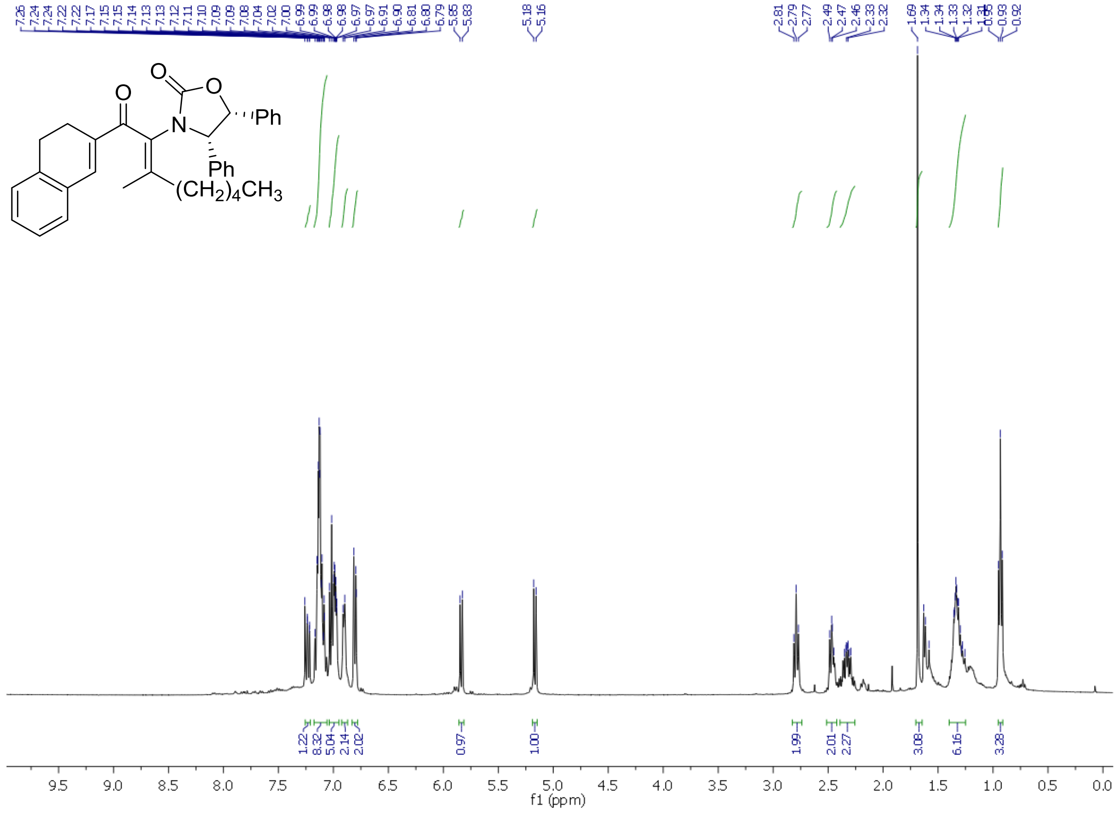

^{13}C NMR (CDCl$_3$, 101 MHz, DEPT-Q)

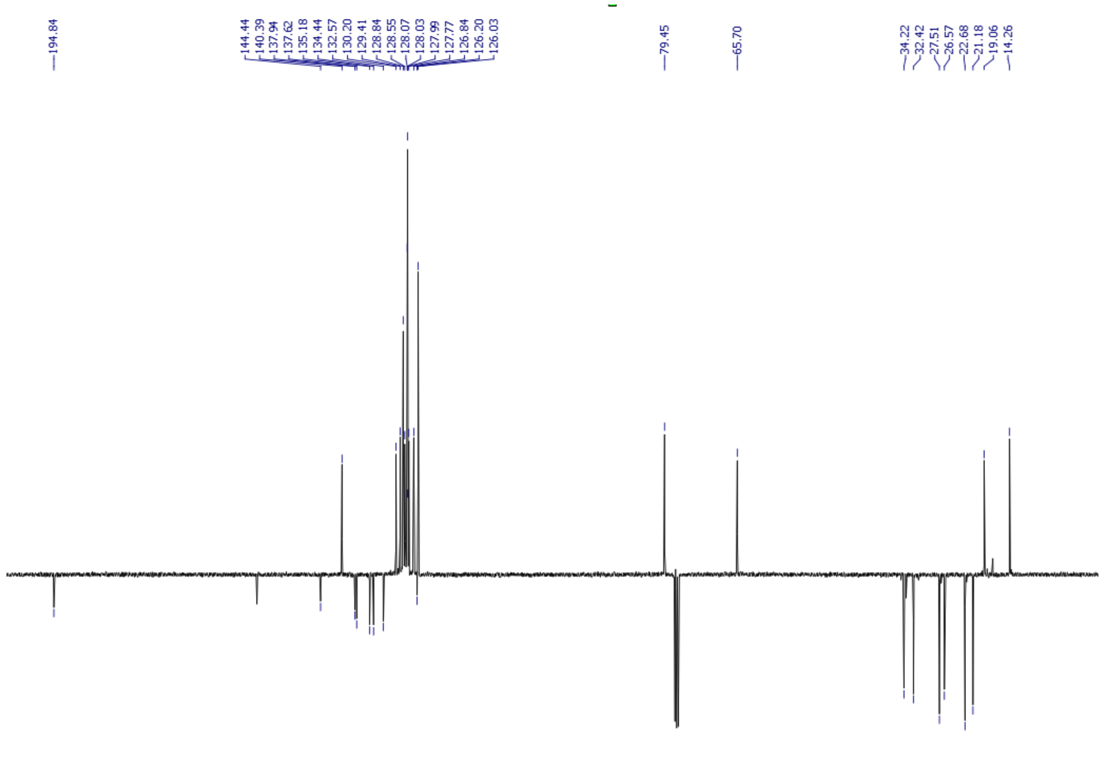

(4*S*,5*R*)-3-((*Z*)-3-(3,4-dihydronaphthalen-2-yl)-1-(3,5-dimethoxyphenyl)-3-oxo-1-phenylprop-1-en-2-yl)-4,5-diphenyloxazolidin-2-one 5f

^{1}H NMR ($CDCl_3$, 400 MHz)

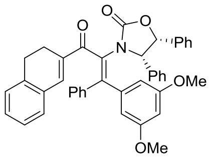

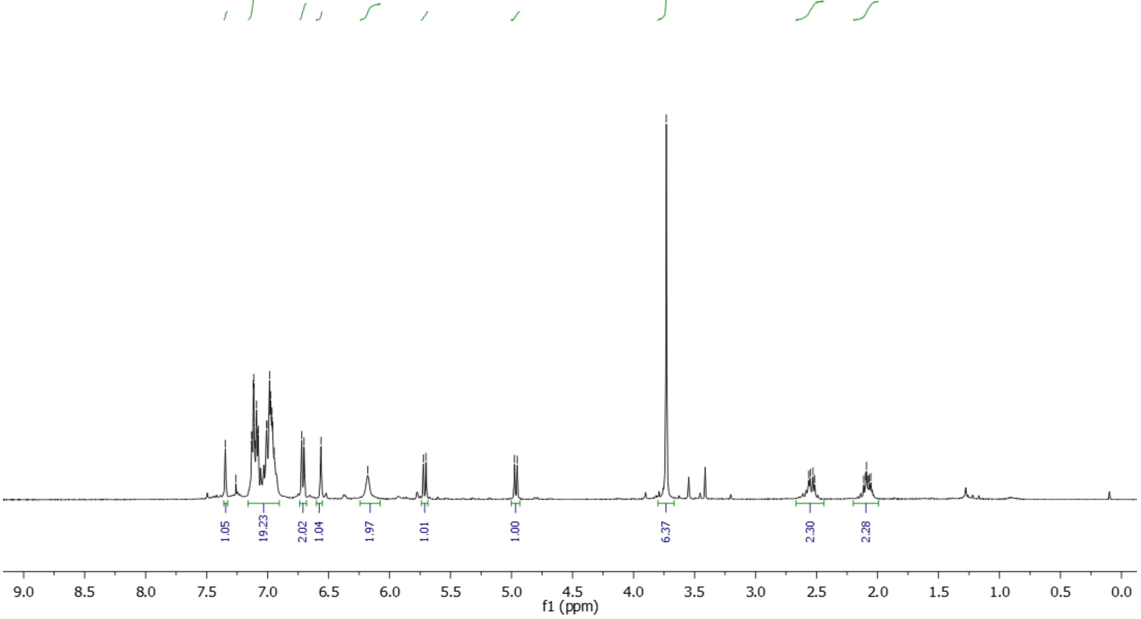

^{13}C NMR ($CDCl_3$, 101 MHz, DEPT-Q)

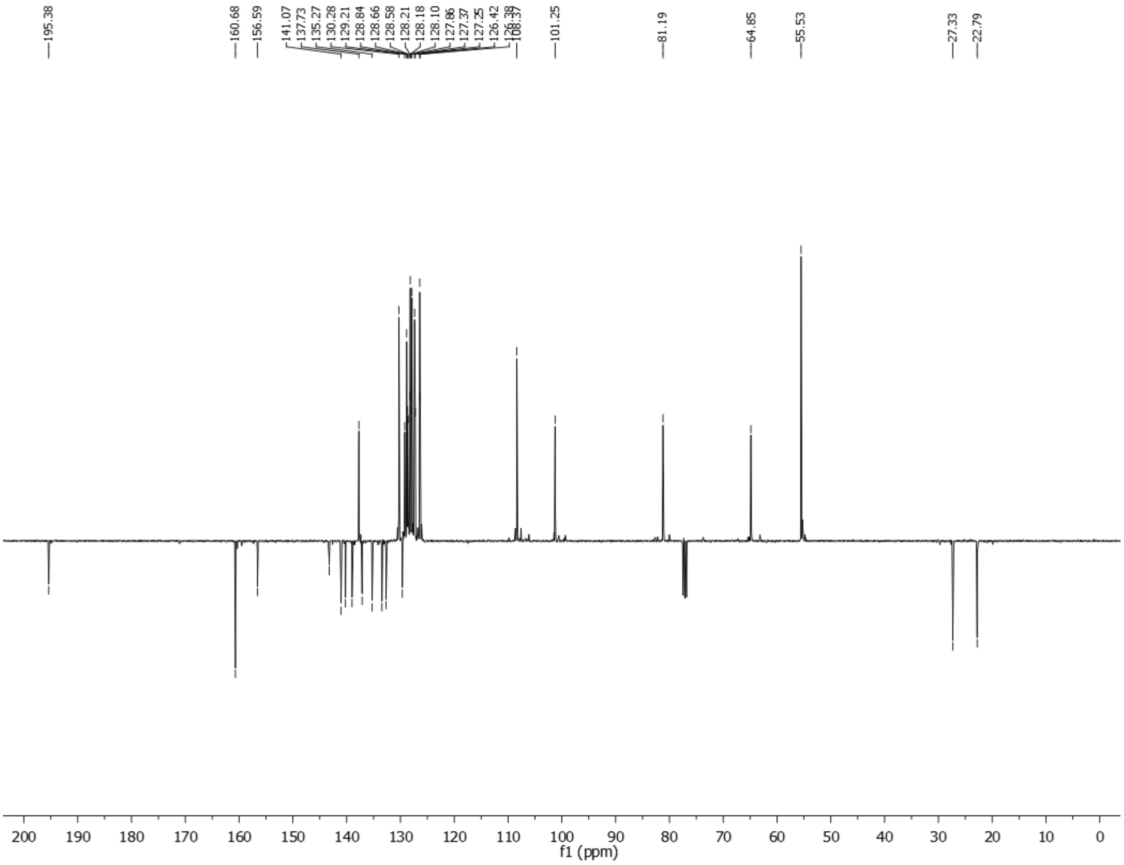

(4*S*,5*R*)-3-((*Z*)-1-(3,5-dimethoxyphenyl)-1-oxo-3-phenylbut-2-en-2-yl)-4,5-diphenyloxazolidin-2-one 5g

^{1}H NMR ($CDCl_3$, 400 MHz)

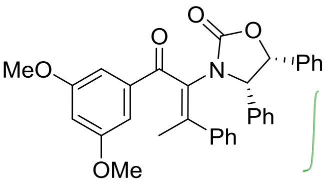

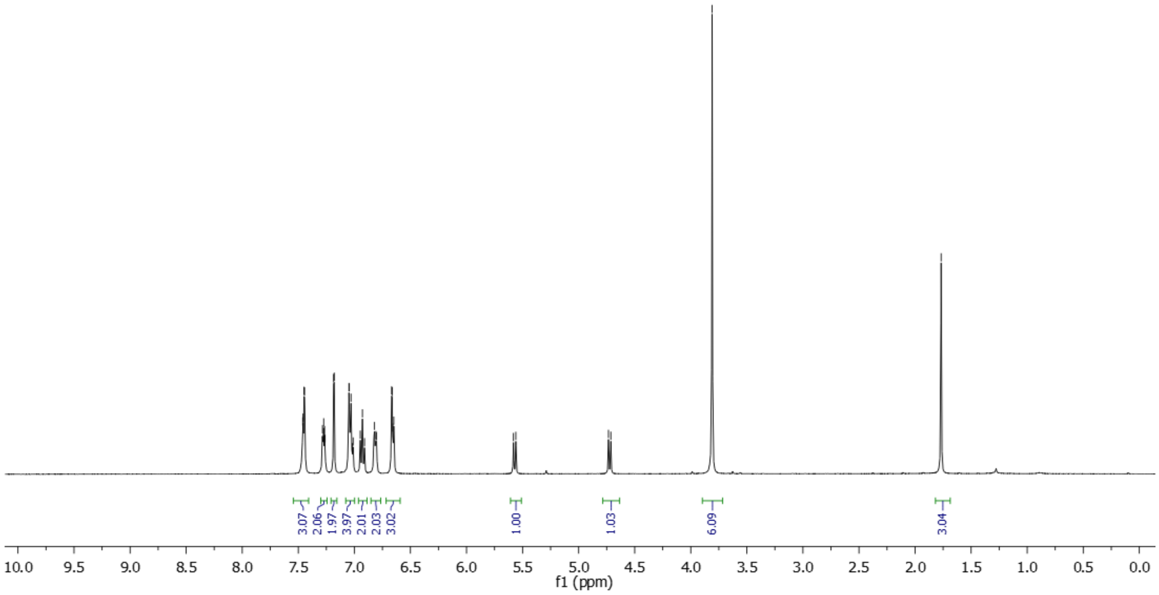

^{13}C NMR ($CDCl_3$, 101 MHz, DEPT-Q)

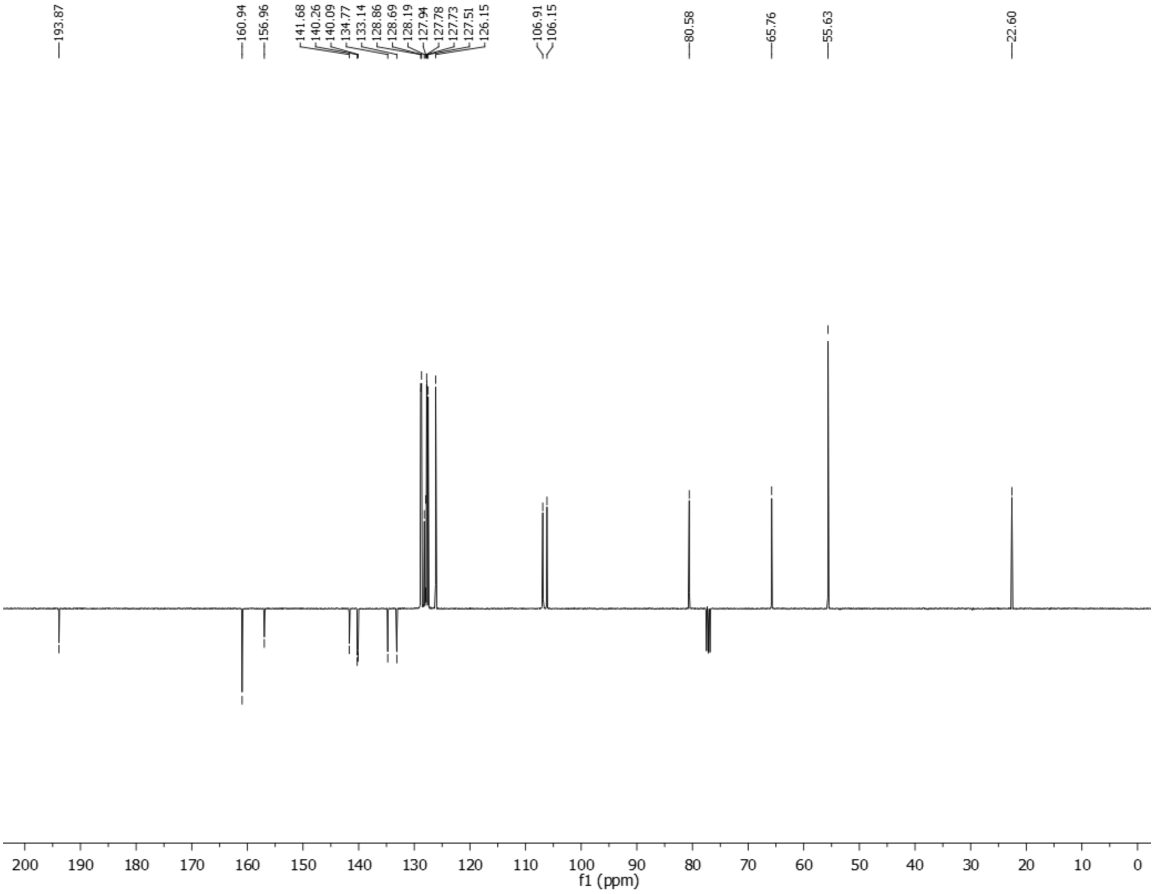

(4*S*,5*R*)-3-((*Z*)-1-(3,5-dimethoxyphenyl)-4-methyl-1-oxo-3-phenylpent-2-en-2-yl)-4,5-diphenyloxazolidin-2-one 5h

^{1}H NMR ($CDCl_3$, 400 MHz)

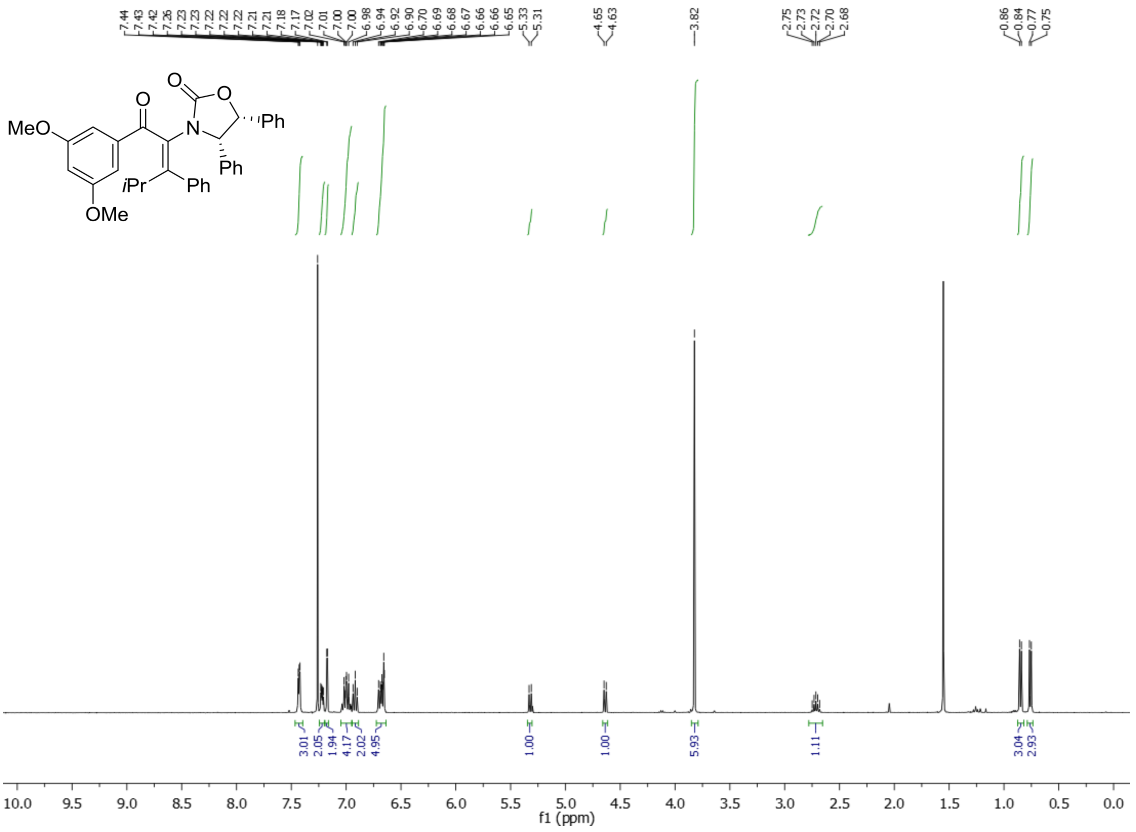

^{13}C NMR ($CDCl_3$, 101 MHz, DEPT-Q)

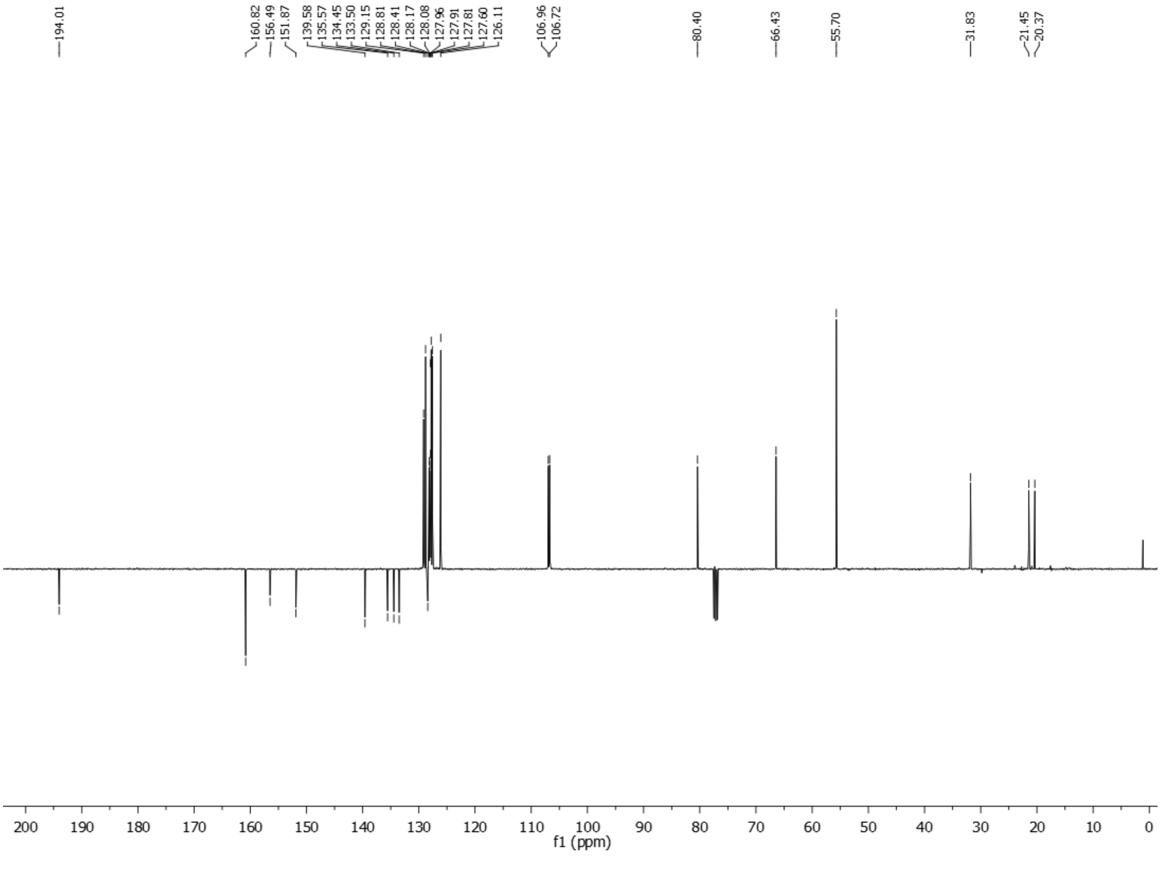

(4*S*,5*R*)-3-((*Z*)-1-oxo-3-phenyl-1-(thiophen-2-yl)but-2-en-2-yl)-4,5-diphenyloxazolidin-2-one 5i

^{1}H NMR ($CDCl_3$, 400 MHz)

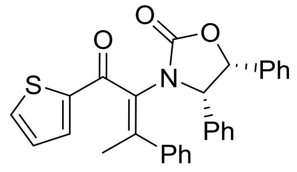

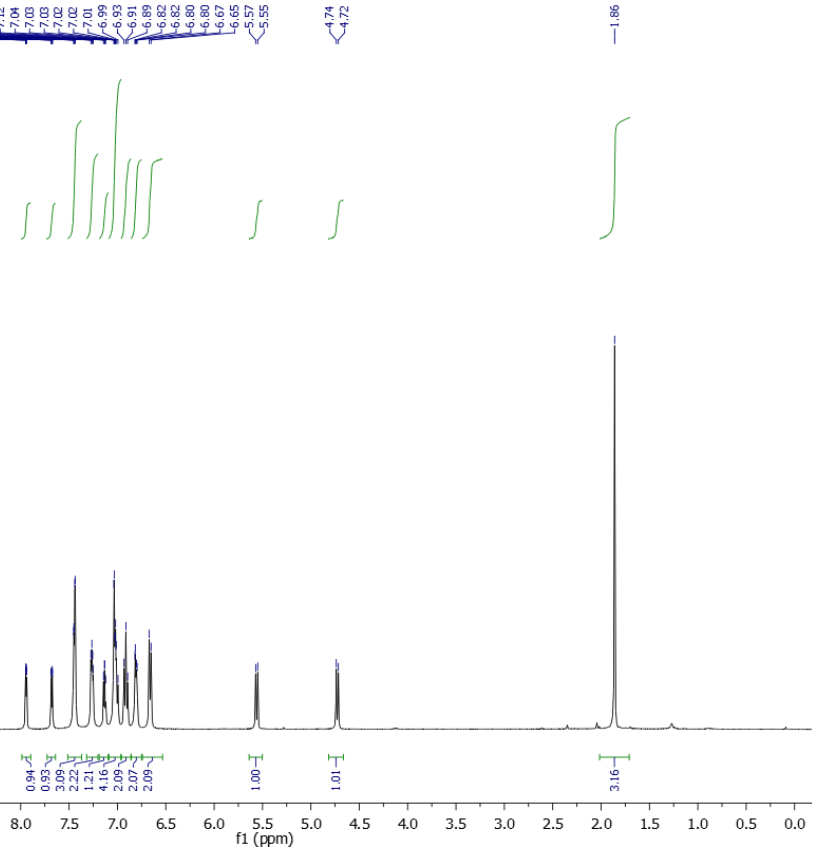

^{13}C NMR ($CDCl_3$, 400 MHz, DEPT-Q)

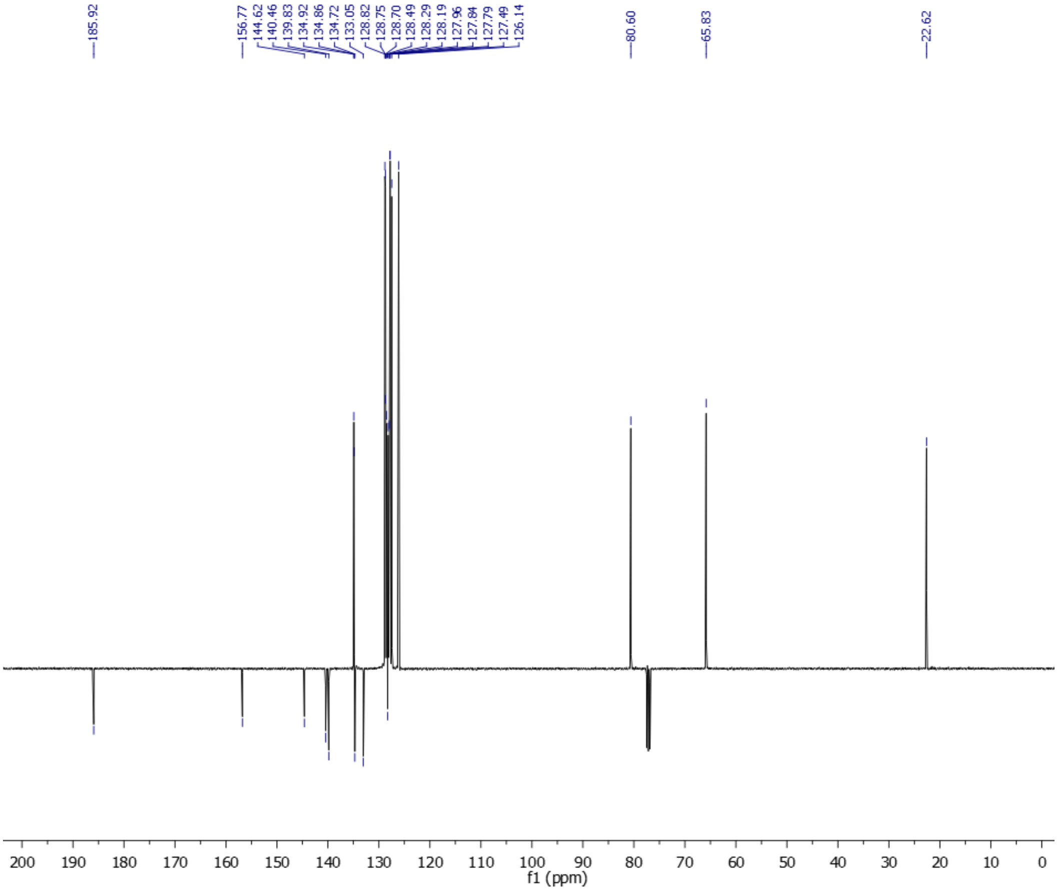

(4*S*,5*R*)-3-((*Z*)-1-(4-methoxyphenyl)-1-oxo-3-phenylbut-2-en-2-yl)-4,5-diphenyloxazolidin-2-one 5j

^{1}H NMR ($CDCl_3$, 400 MHz)

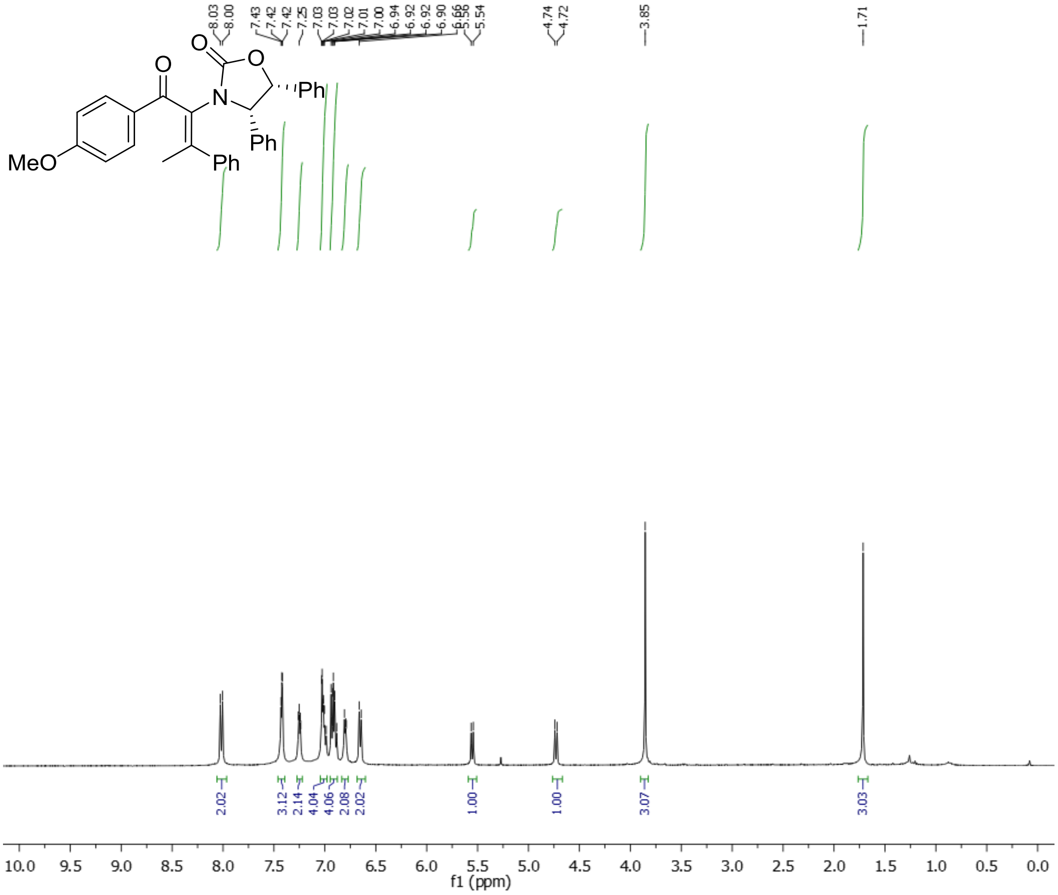

^{13}C NMR ($CDCl_3$, 101 MHz, DEPT-Q)

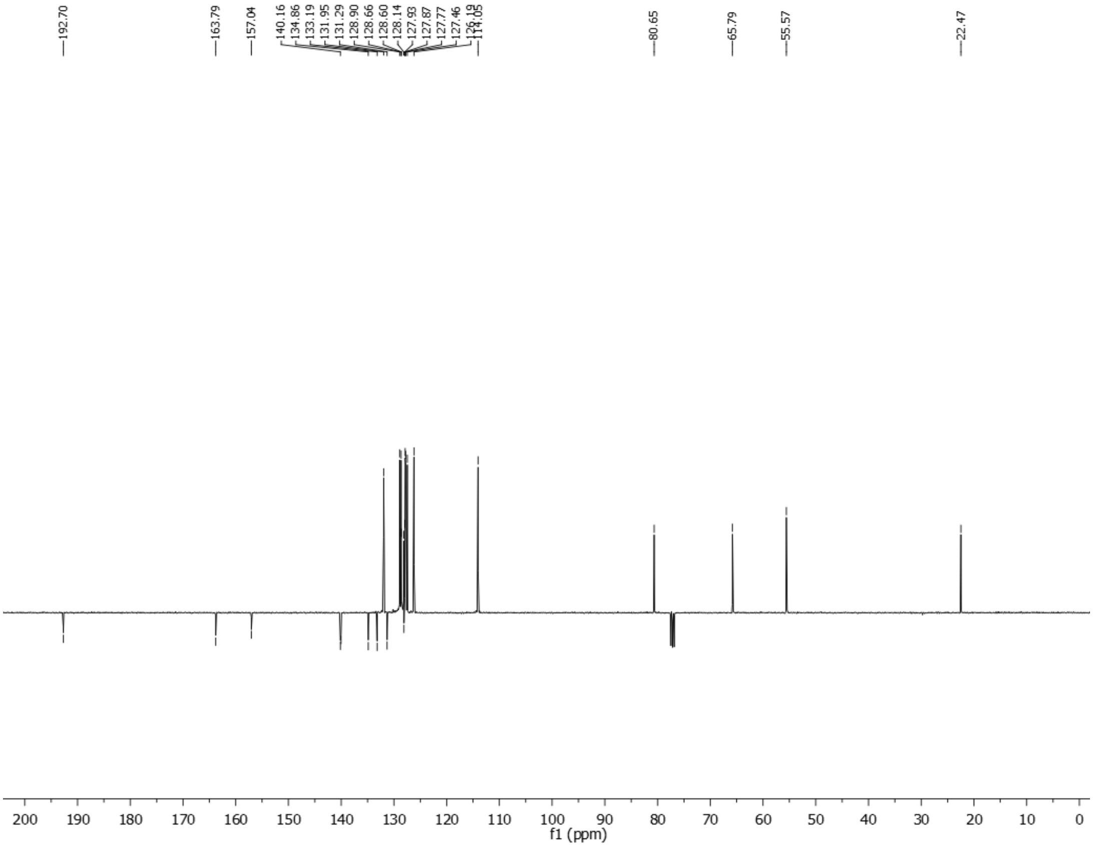

(4*S*,5*R*)-3-((1*S*)-1-methyl-3-oxo-1-phenyl-2,3,4,5,6,7-hexahydro-1*H*-inden-2-yl)-4,5-diphenyloxazolidin-2-one 7a

^{1}H NMR ($CDCl_3$, 400 MHz)

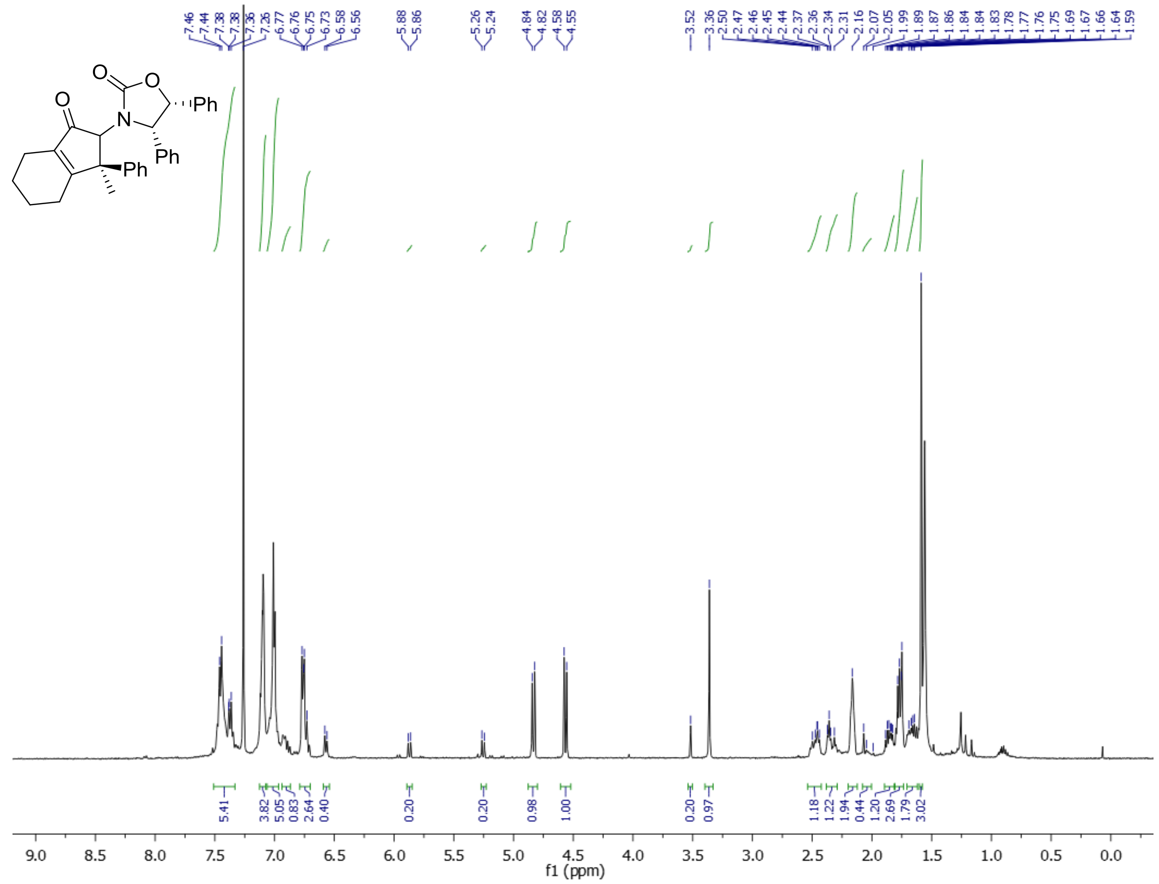

^{13}C NMR ($CDCl_3$, 101 MHz, DEPT-Q)

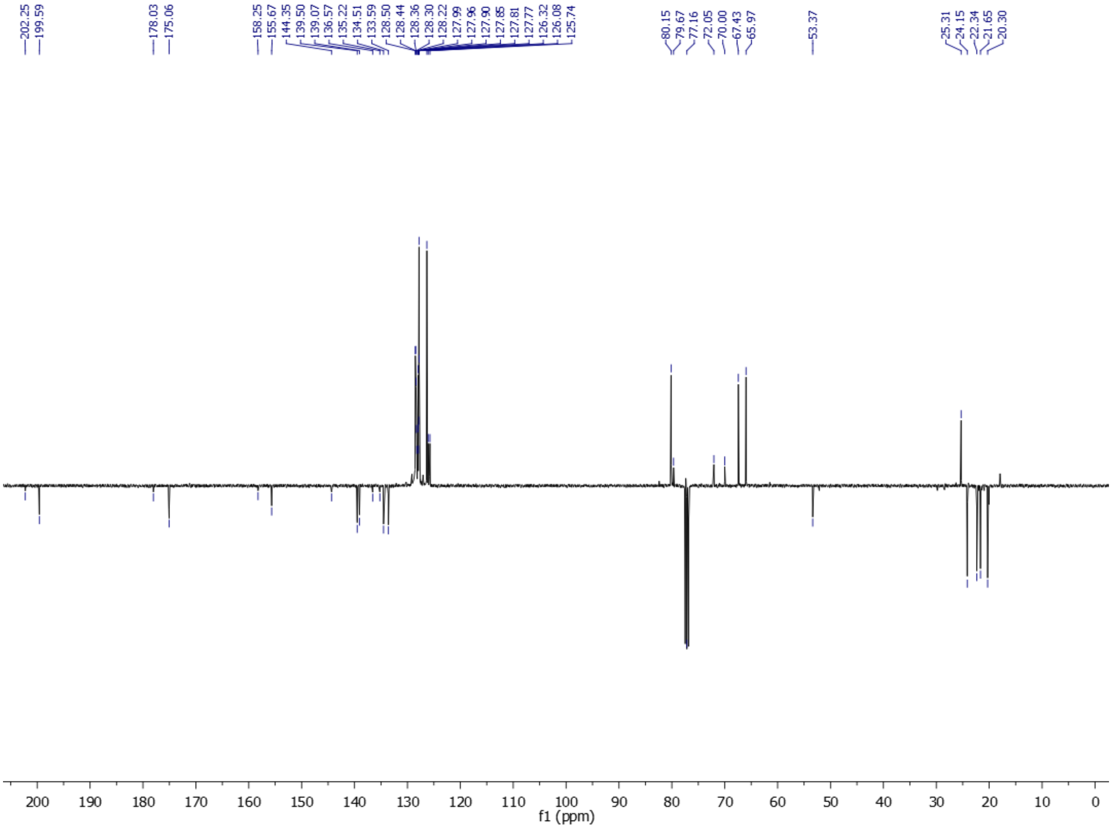

NOESY (CDCl$_3$, 400 MHz)

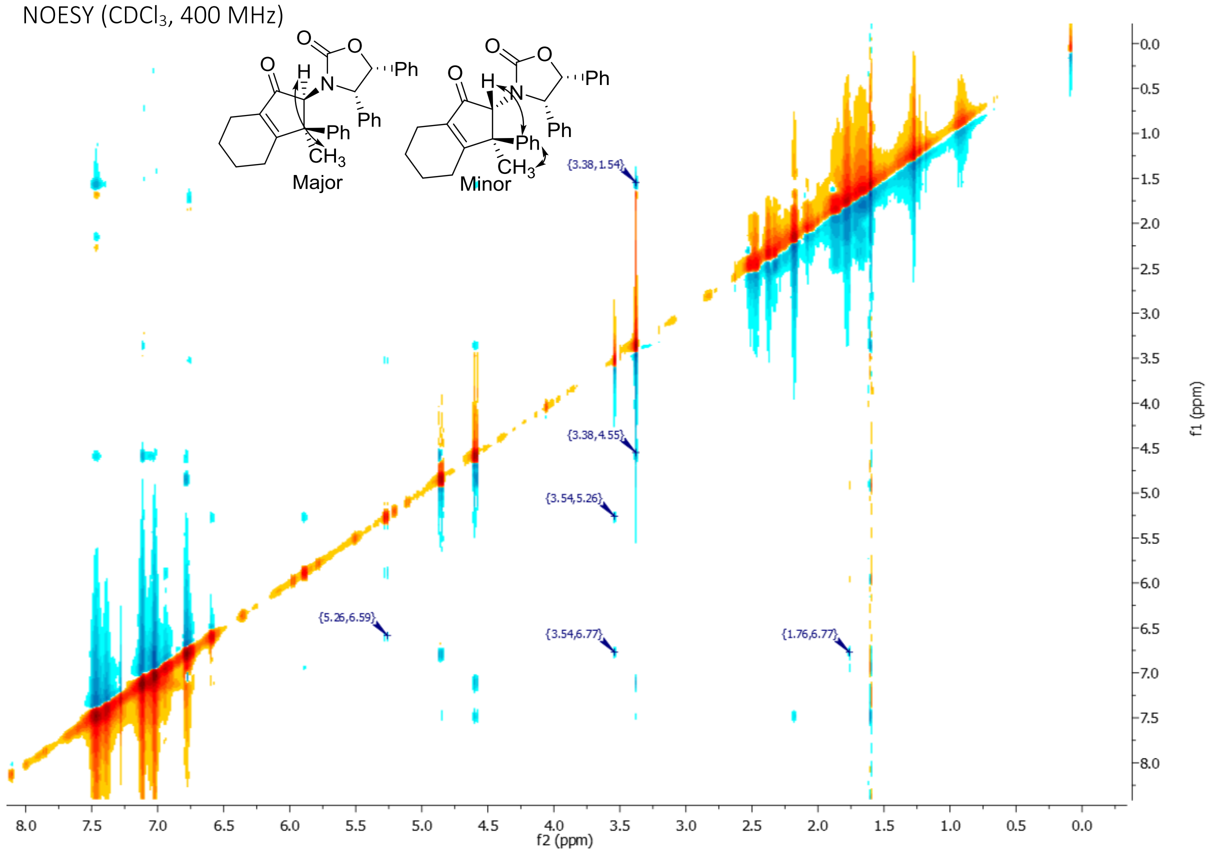

(4*S*,5*R*)-3-((1*R*)-1-methyl-3-oxo-1-phenyl-2,3,4,5,6,7-hexahydro-1*H*-inden-2-yl)-4,5-diphenyloxazolidin-2-one 7a'

^{1}H NMR ($CDCl_3$, 400 MHz)

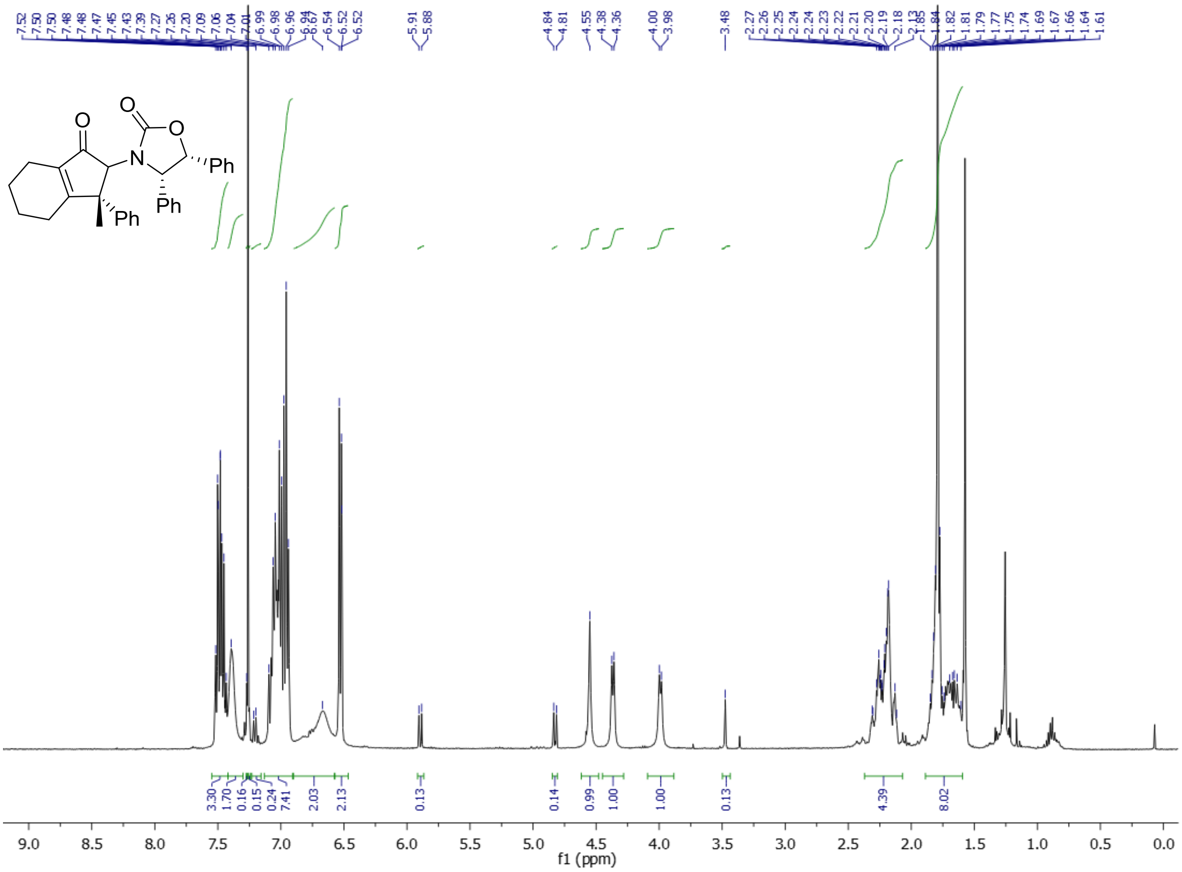

^{13}C NMR ($CDCl_3$, 101 MHz, DEPT-Q)

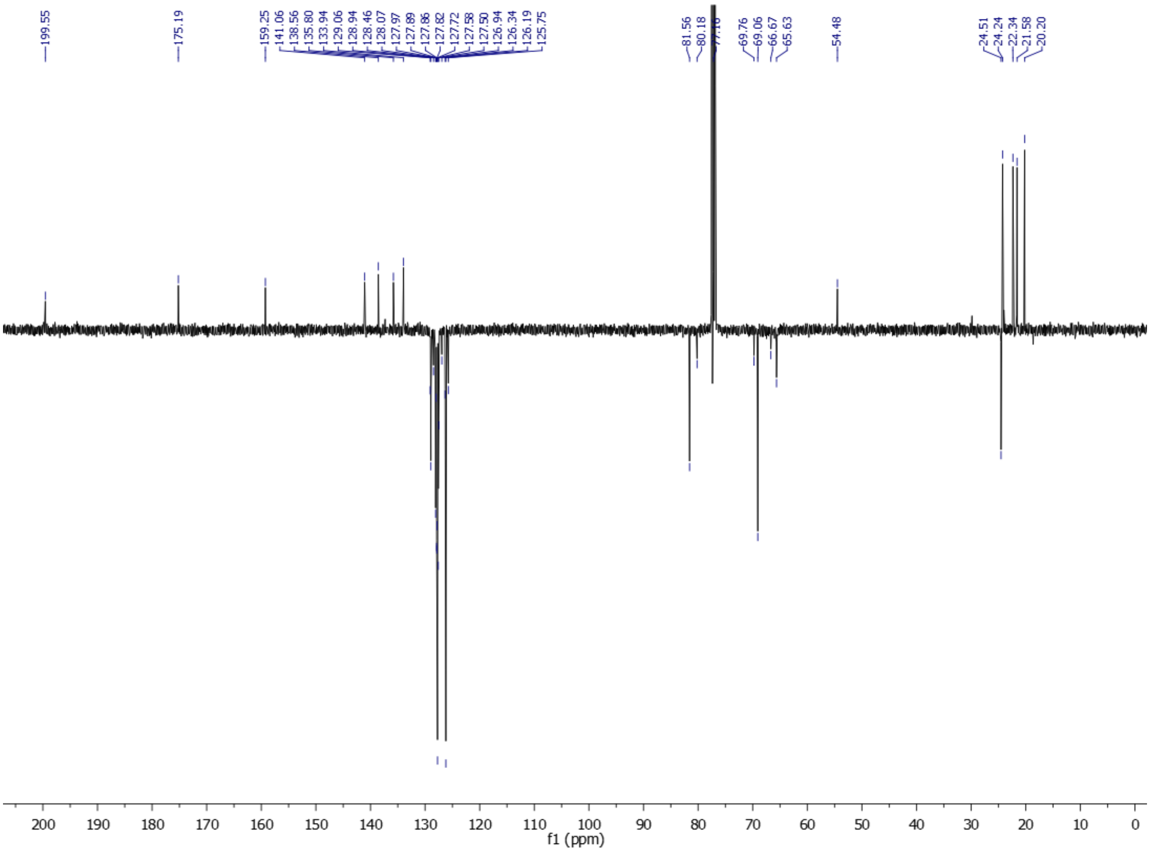

(4*S*,5*R*)-3-((1*R*)-1-methyl-3-oxo-1-phenyl-2,3,4,5,6,7-hexahydro-1*H*-inden-2-yl)-4,5-diphenyloxazolidin-2-one 7a’

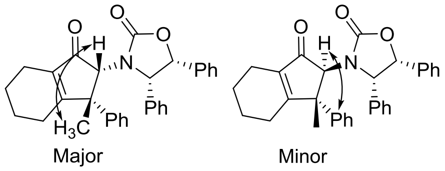

NOESY ($CDCl_3$, 400 MHz)

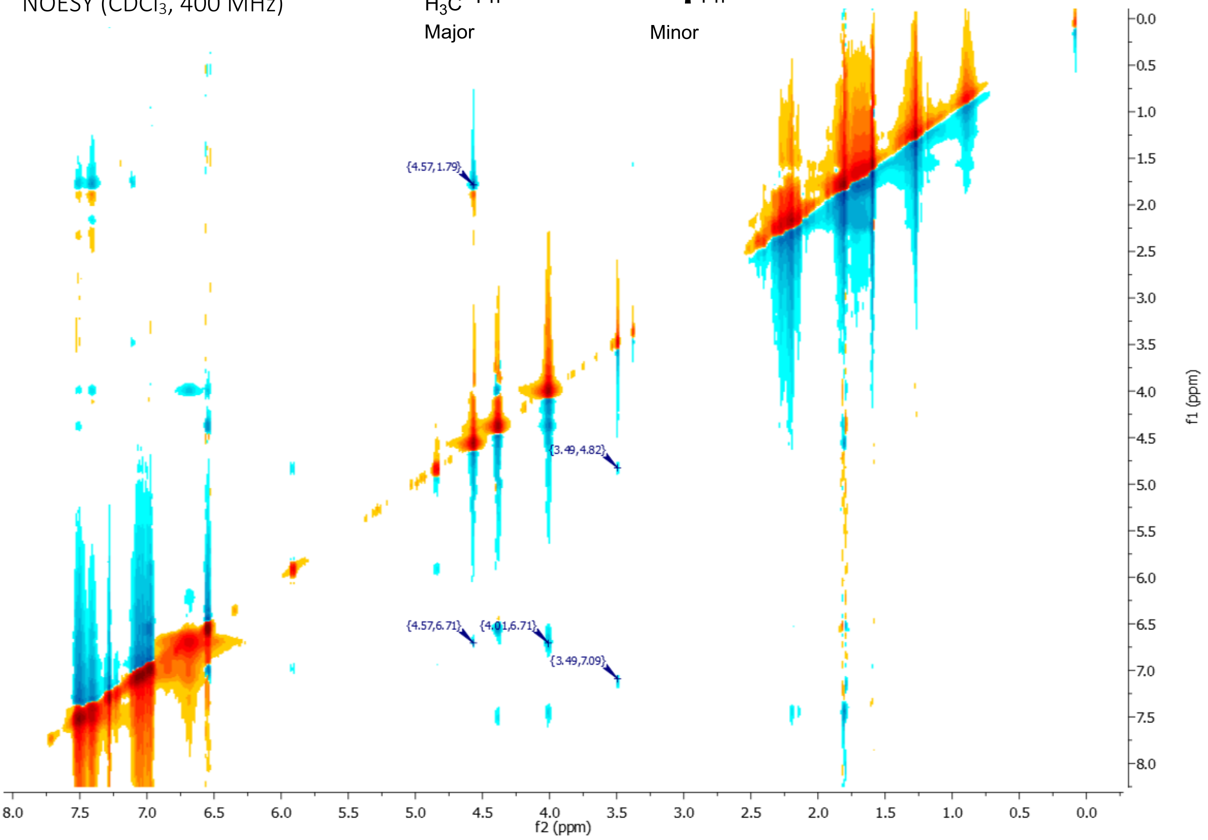

(4*S*,5*R*)-3-((1*S*)-1-ethyl-3-oxo-1-phenyl-2,3,4,5,6,7-hexahydro-1*H*-inden-2-yl)-4,5-diphenyloxazolidin-2-one 7b

^{1}H NMR ($CDCl_3$, 400 MHz)

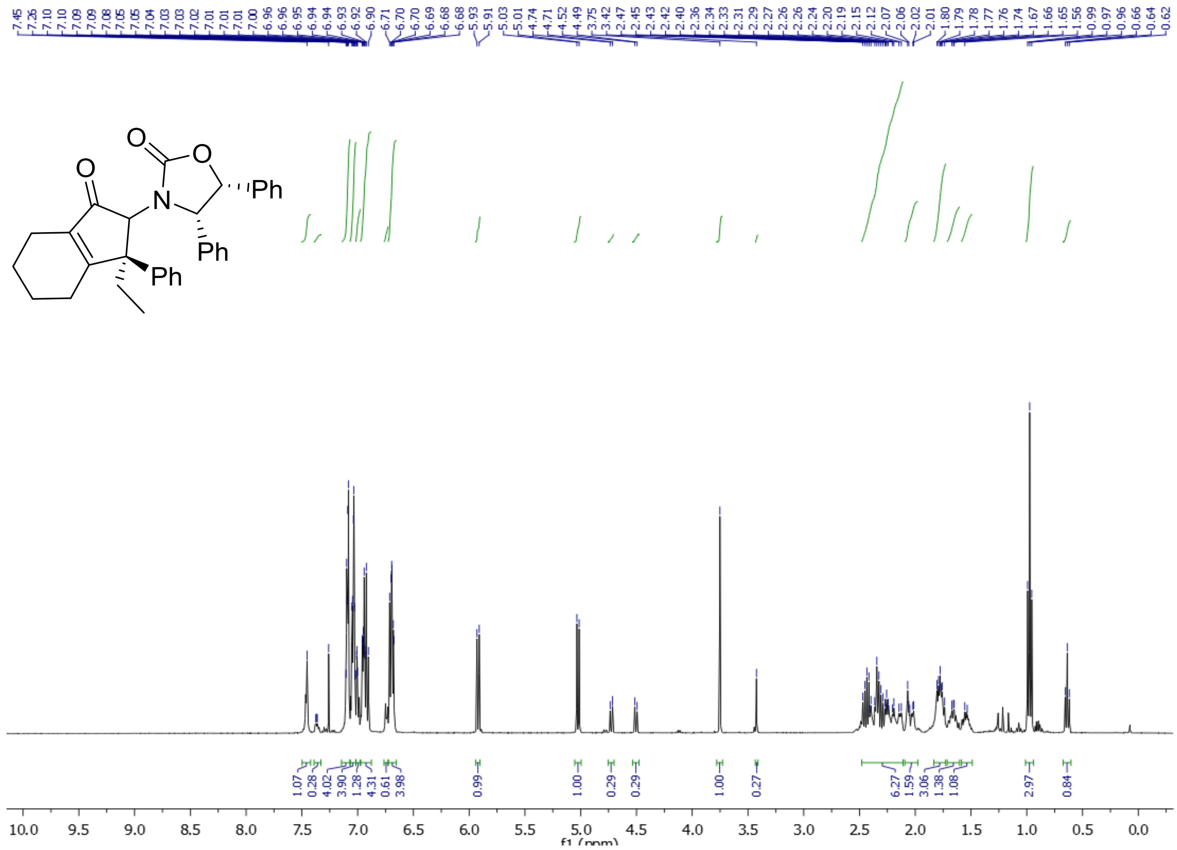

^{13}C NMR ($CDCl_3$, 101 MHz, DEPT-Q)

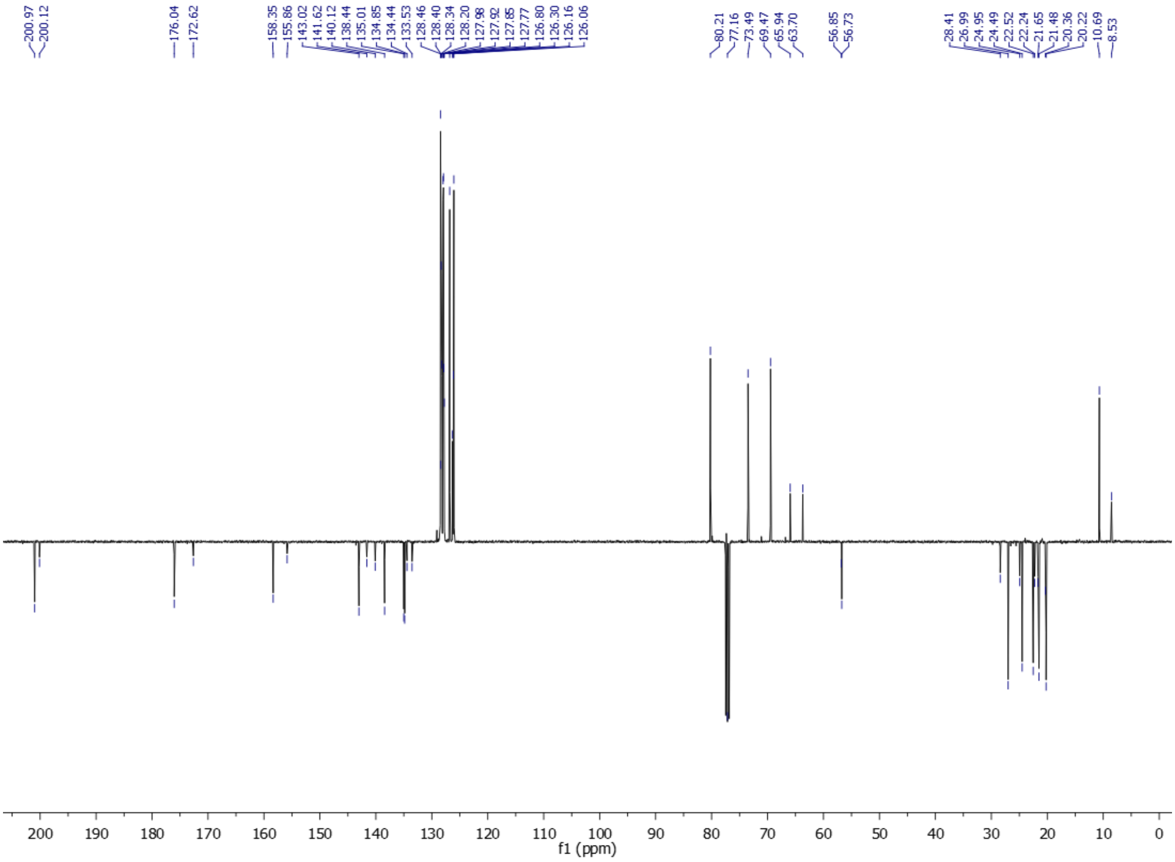

(4*S*,5*R*)-3-((1*S*)-1-ethyl-3-oxo-1-phenyl-2,3,4,5,6,7-hexahydro-1*H*-inden-2-yl)-4,5-diphenyloxazolidin-2-one **7b**

NOESY ($CDCl_3$, 400 MHz)

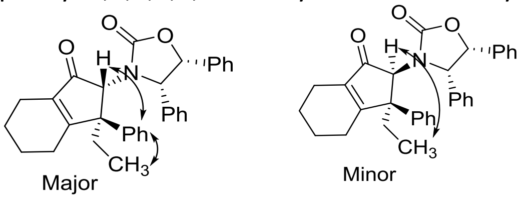

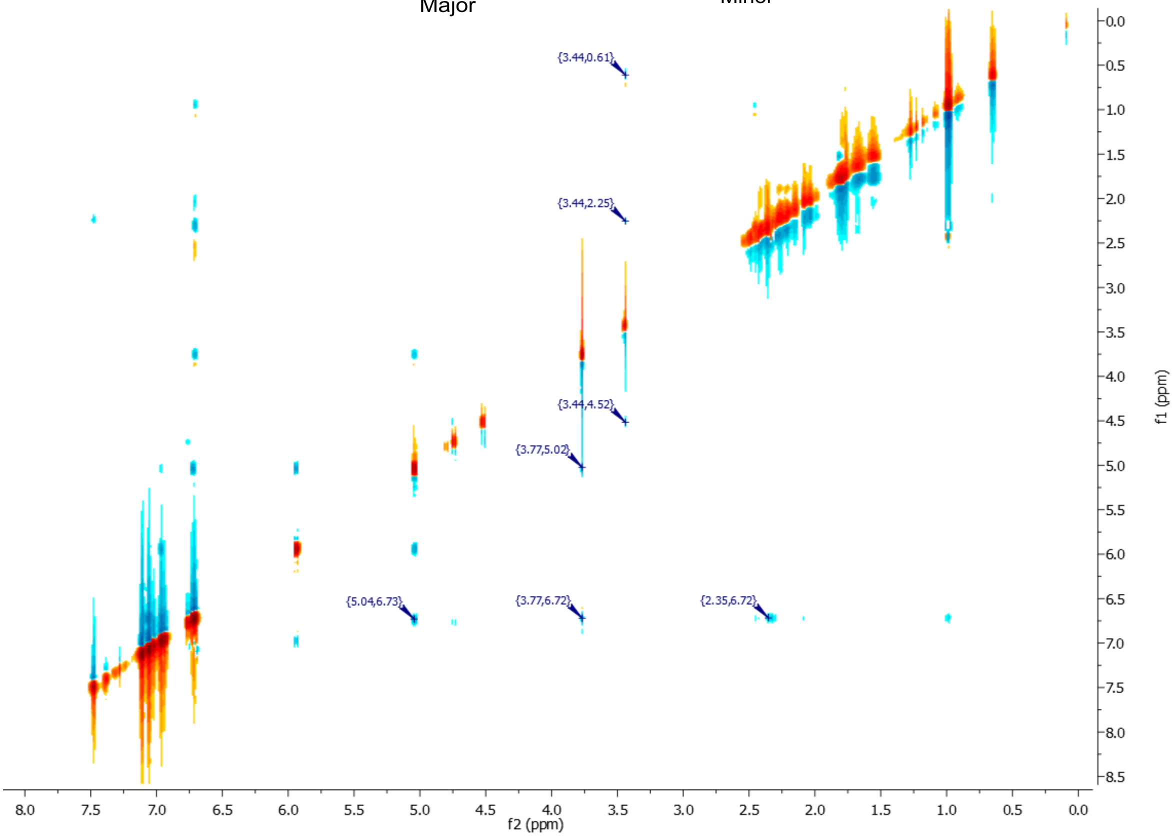

(4*S*,5*R*)-3-((1*R*,2*S*)-1-ethyl-3-oxo-1-phenyl-2,3,4,5,6,7-hexahydro-1*H*-inden-2-yl)-4,5-diphenyloxazolidin-2-one 7b’

^{1}H NMR ($CDCl_3$, 400 MHz)

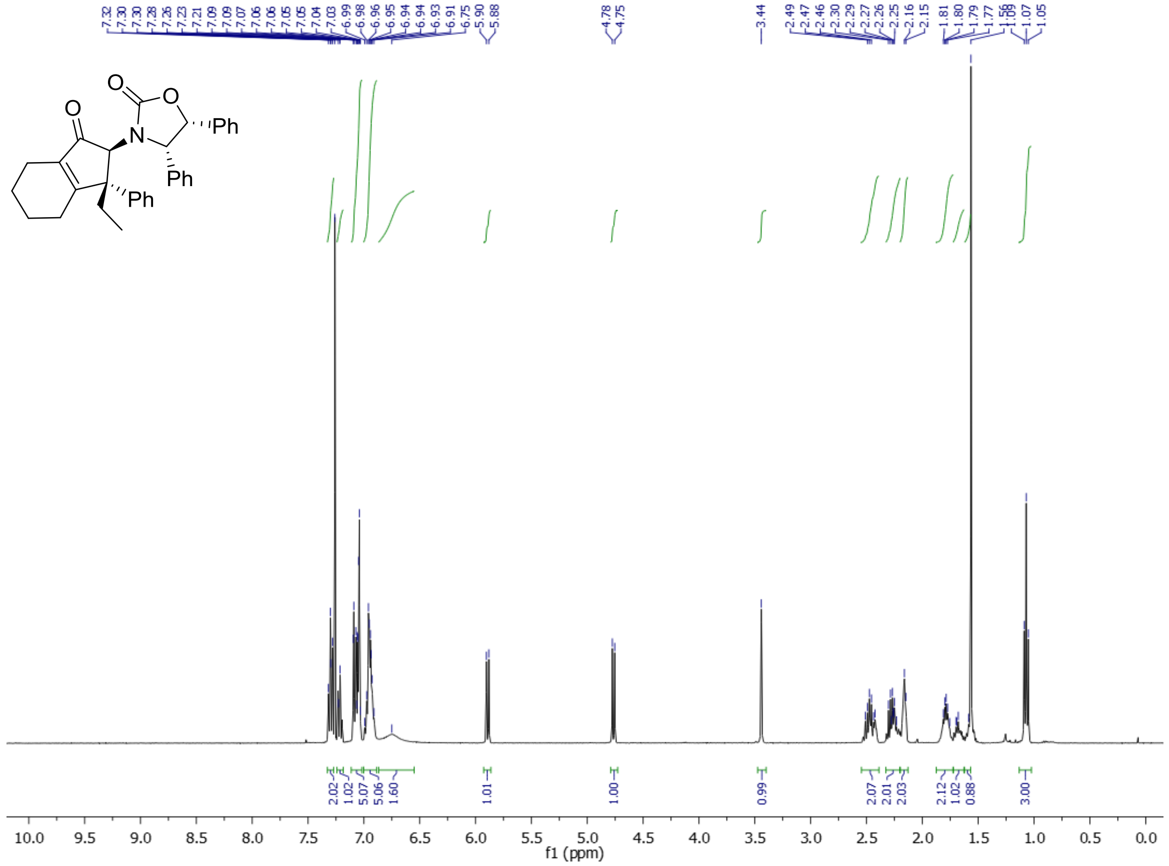

^{13}C NMR ($CDCl_3$, 101 MHz, DEPT-Q)

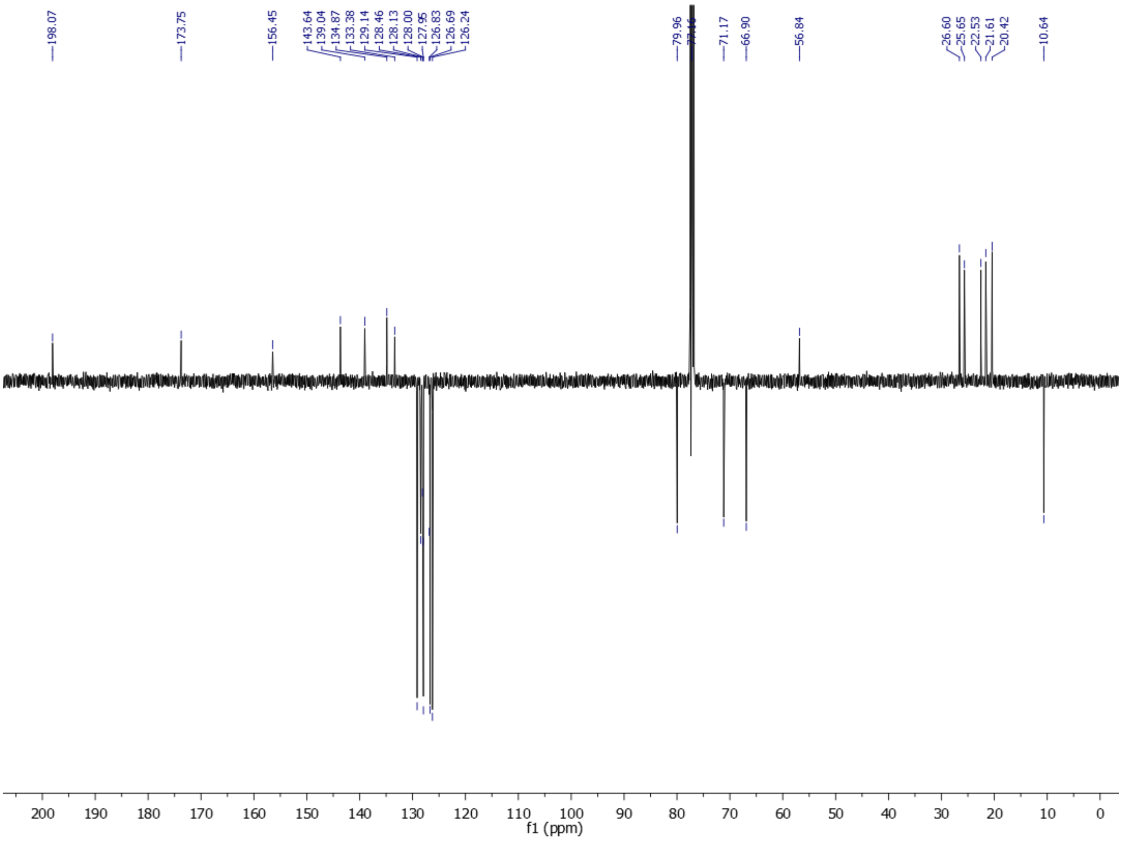

(4*S*,5*R*)-3-((1*R*,2*S*)-1-ethyl-3-oxo-1-phenyl-2,3,4,5,6,7-hexahydro-1*H*-inden-2-yl)-4,5-diphenyloxazolidin-2-one 7b’

NOESY ($CDCl_3$, 400 MHz)

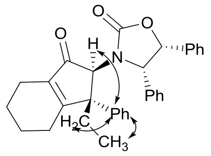

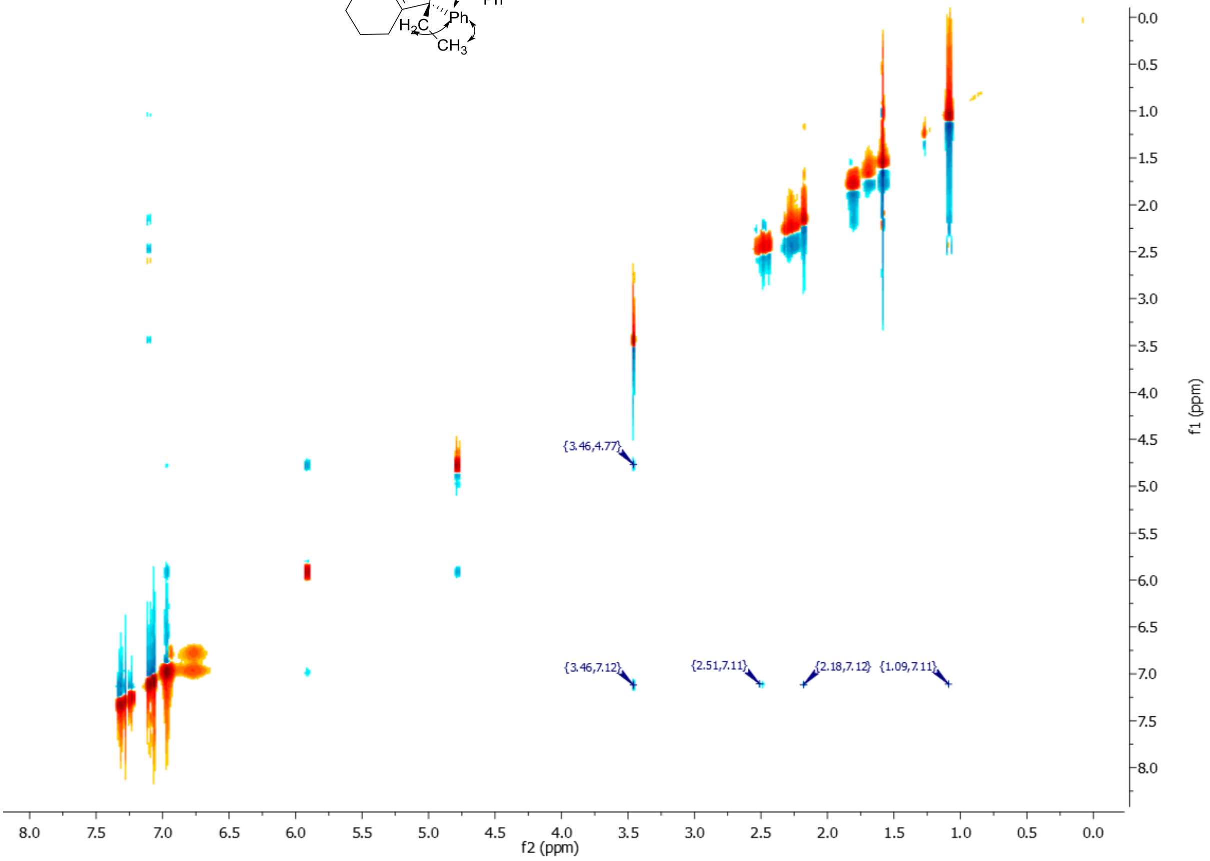

(4*S*,5*R*)-3-((1*S*,2*S*)-1-methyl-3-oxo-1-phenyl-2,3,4,5-tetrahydro-1*H*-cyclopenta[*a*]naphthalen-2-yl)-4,5-diphenyloxazolidin-2-one 7c

^{1}H NMR ($CDCl_3$, 400 MHz)

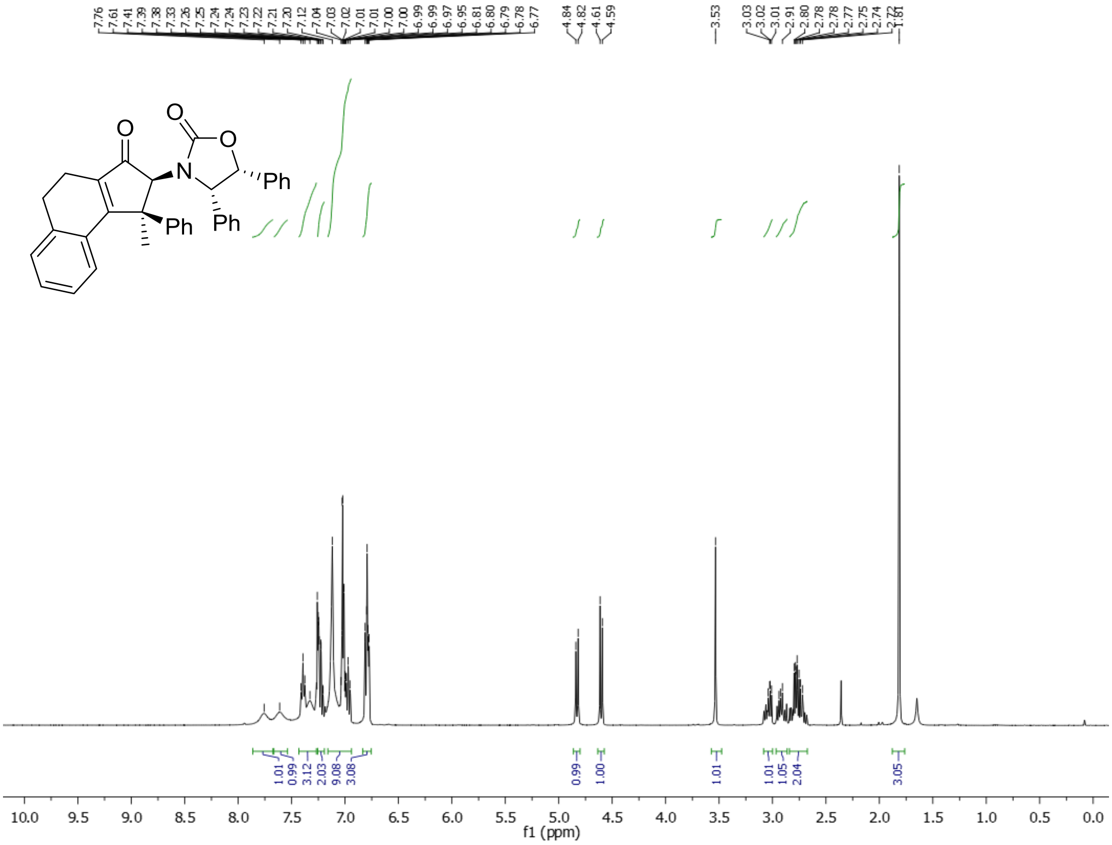

^{13}C NMR ($CDCl_3$, 101 MHz, DEPT-Q)

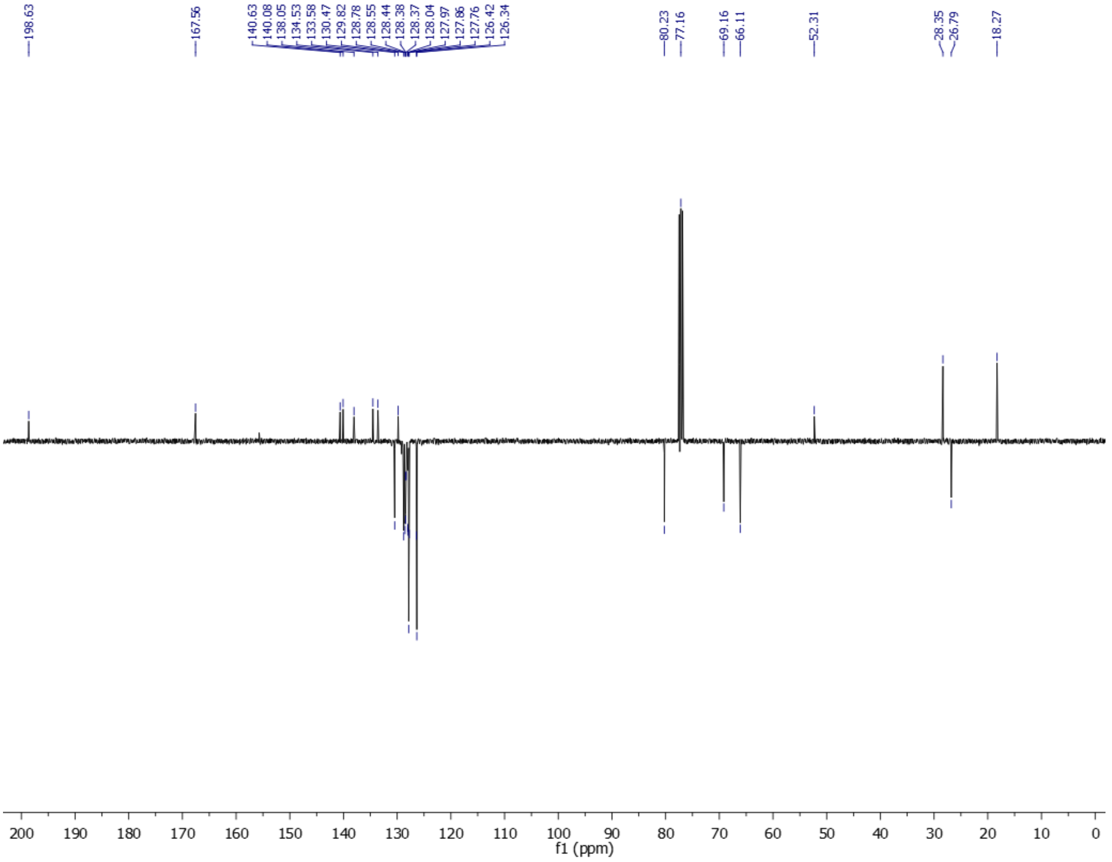

(4*S*,5*R*)-3-((1*S*,2*S*)-1-methyl-3-oxo-1-phenyl-2,3,4,5-tetrahydro-1*H*-cyclopenta[*a*]naphthalen-2-yl)-4,5-diphenyloxazolidin-2-one 7c

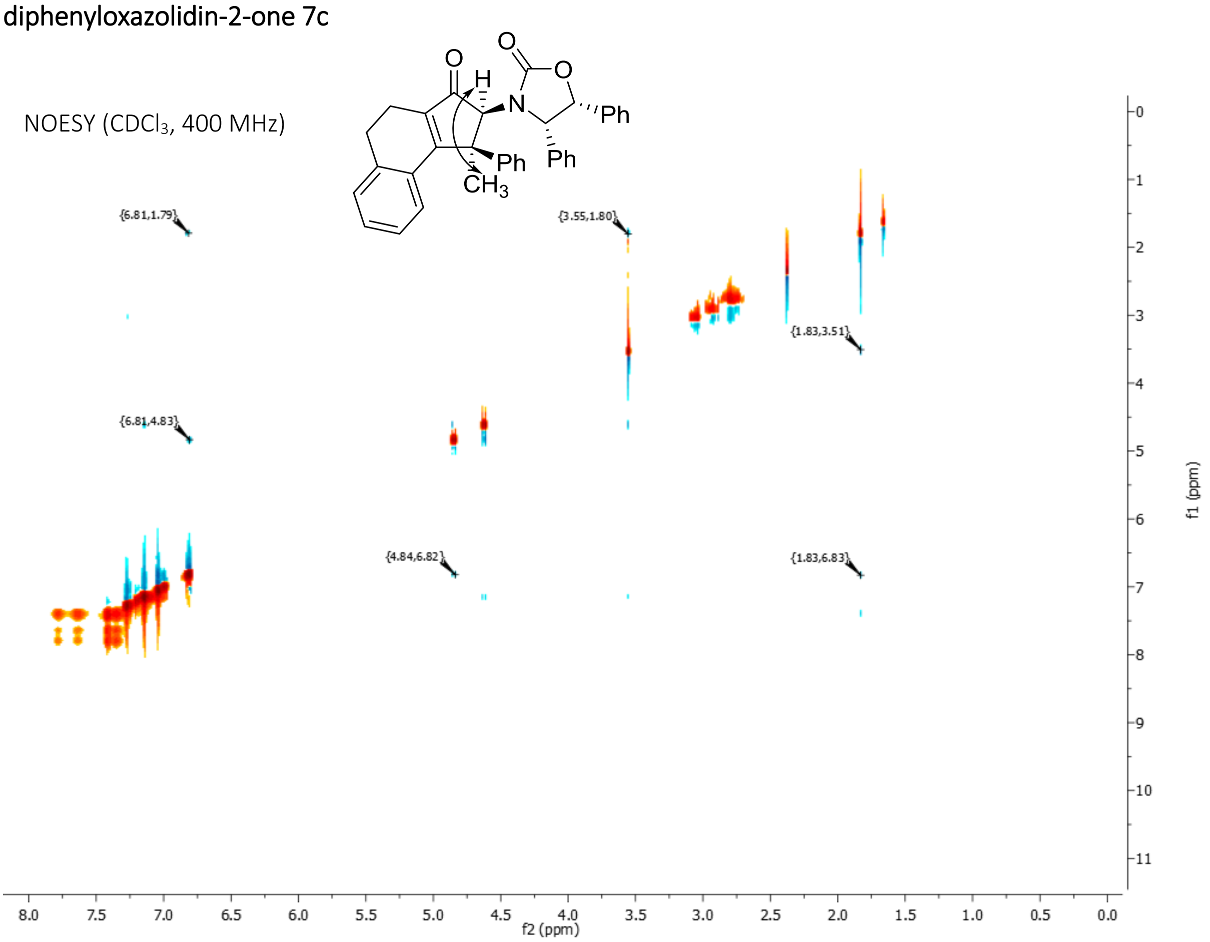

(4*S*,5*R*)-3-((1*S*)-1-isopropyl-3-oxo-1-phenyl-2,3,4,5-tetrahydro-1*H*-cyclopenta[*a*]naphthalen-2-yl)-4,5-diphenyloxazolidin-2-one (1*S*)-7d

^{1}H NMR ($CDCl_3$, 400 MHz)

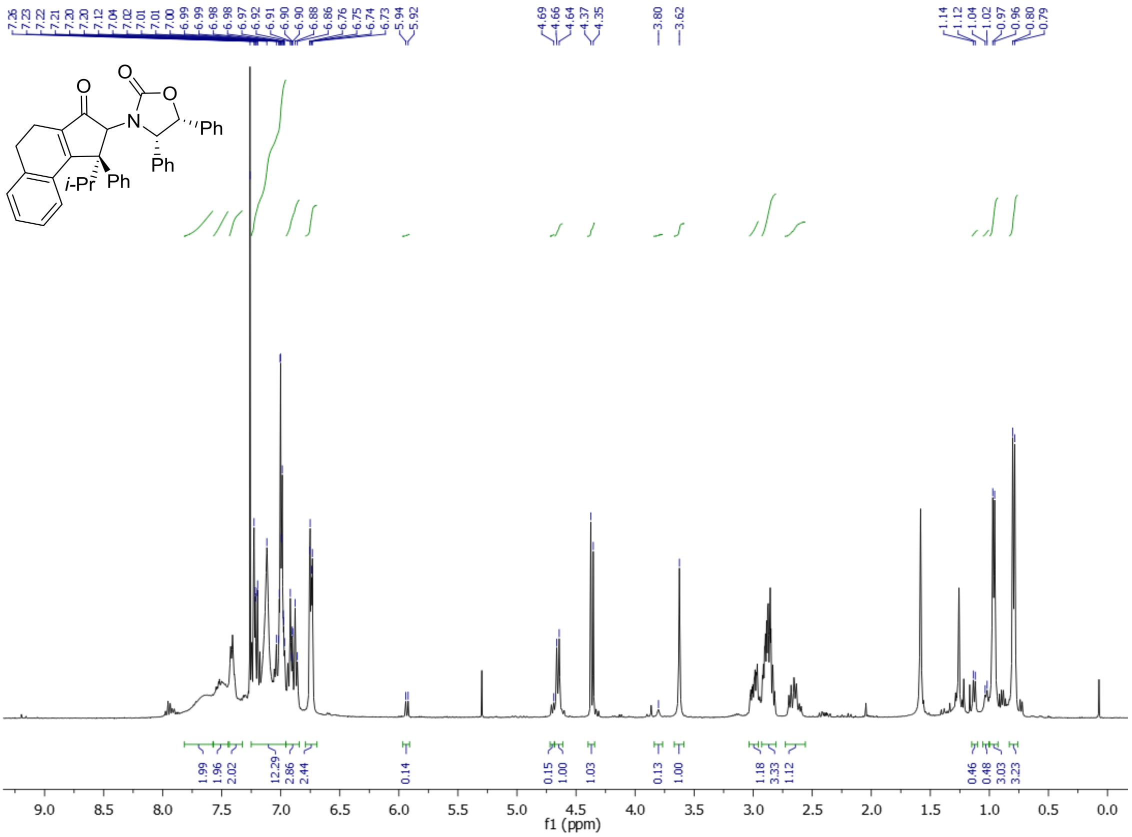

^{13}C NMR ($CDCl_3$, 101 MHz, DEPT-Q)

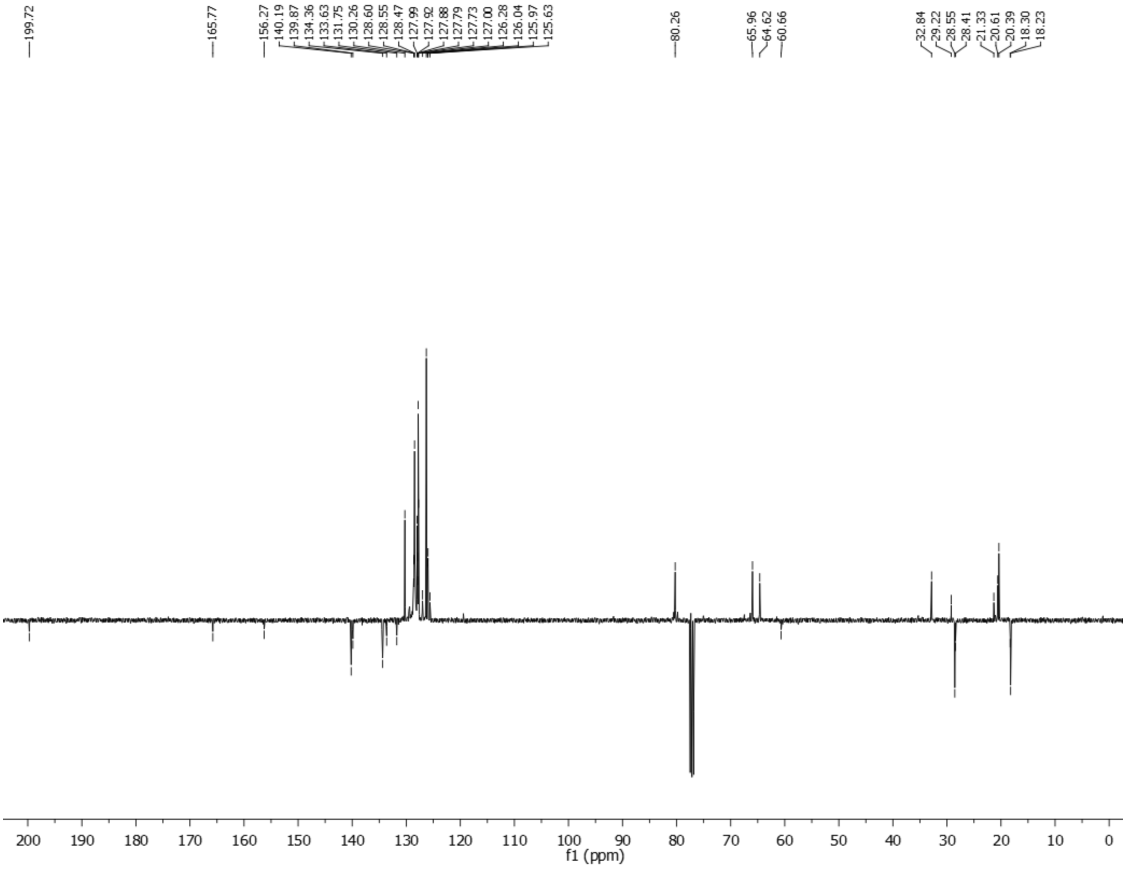

(4*S*,5*R*)-3-((1*S*)-1-isopropyl-3-oxo-1-phenyl-2,3,4,5-tetrahydro-1*H*-cyclopenta[*a*]naphthalen-2-yl)-4,5-diphenyloxazolidin-2-one (1*S*)-7d

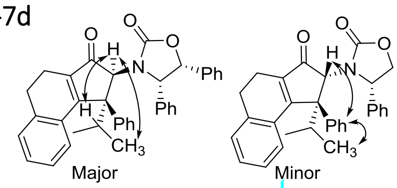

NOESY ($CDCl_3$, 400 MHz)

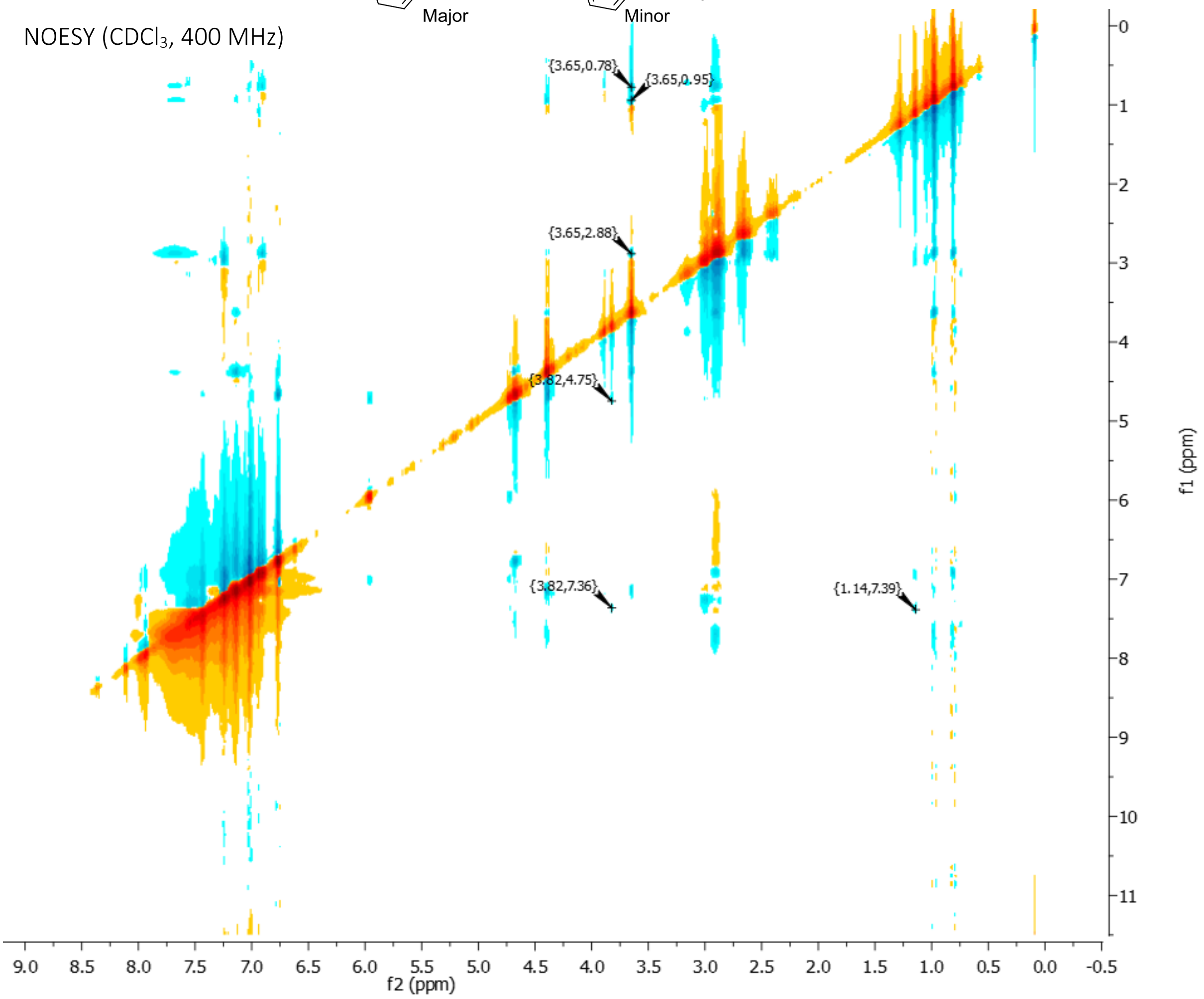

(4*S*,5*R*)-3-((1*R*)-1-isopropyl-3-oxo-1-phenyl-2,3,4,5-tetrahydro-1*H*-cyclopenta[*a*]naphthalen-2-yl)-4,5-diphenyloxazolidin-2-one (1*R*)-7d with minor amount of (1*S*)-7d

^{1}H NMR ($CDCl_3$, 400 MHz)

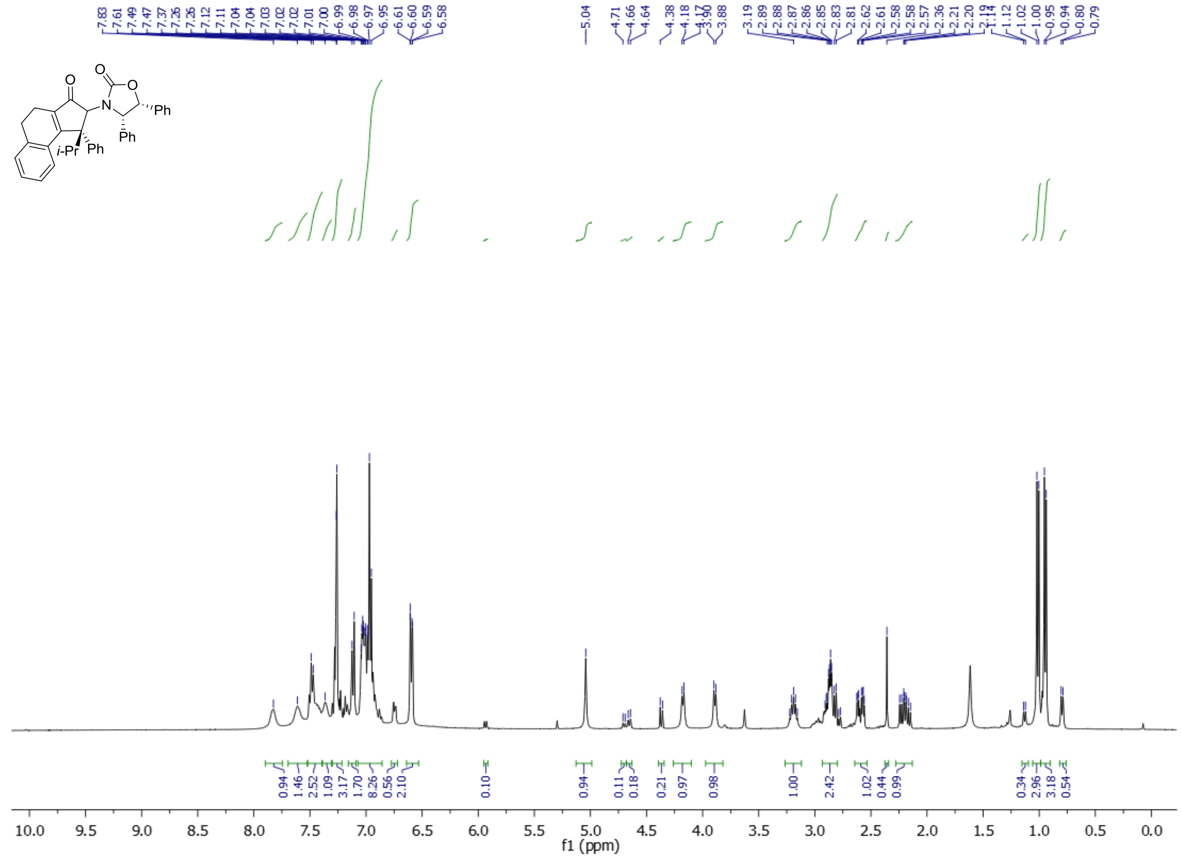

^{13}C NMR ($CDCl_3$, 101 MHz, DEPT-Q)

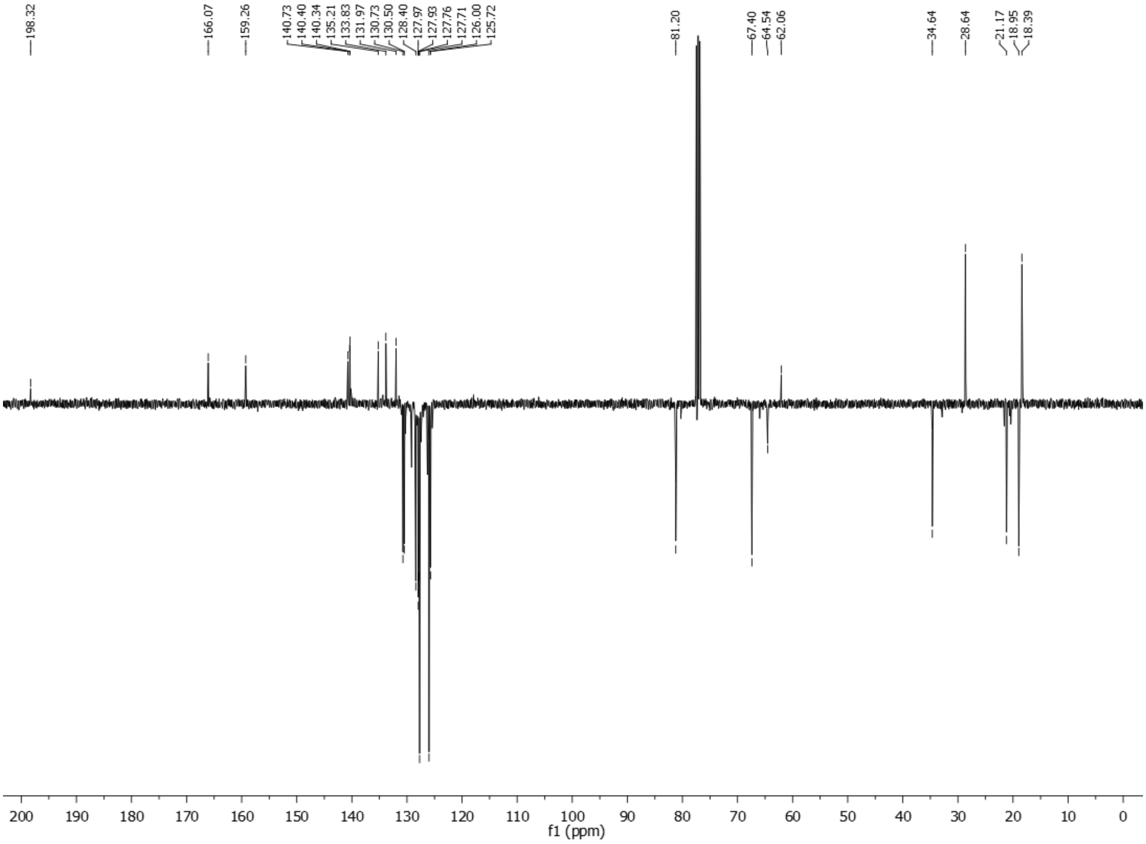

(4*S*,5*R*)-3-((1*R*)-1-isopropyl-3-oxo-1-phenyl-2,3,4,5-tetrahydro-1*H*-cyclopenta[*a*]naphthalen-2-yl)-4,5-diphenyloxazolidin-2-one (1*R*)-7d with minor amount of (1*S*)-7d

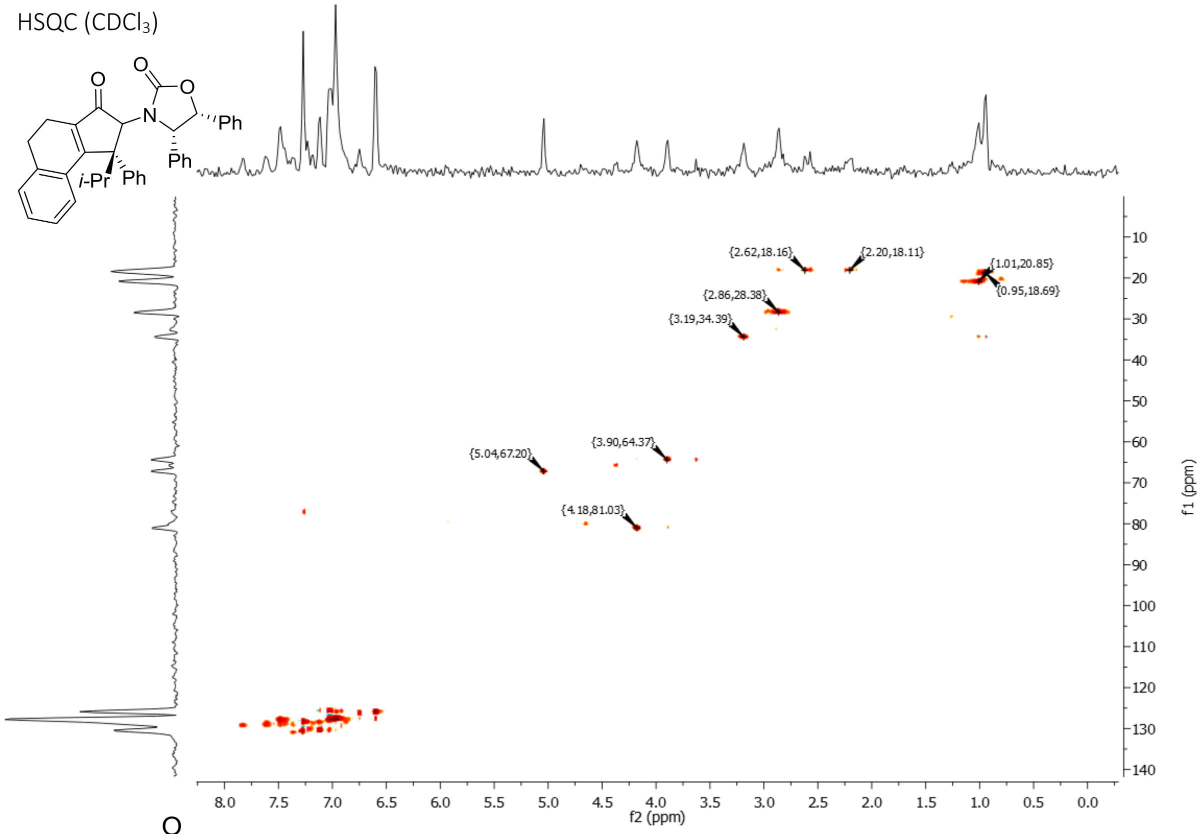

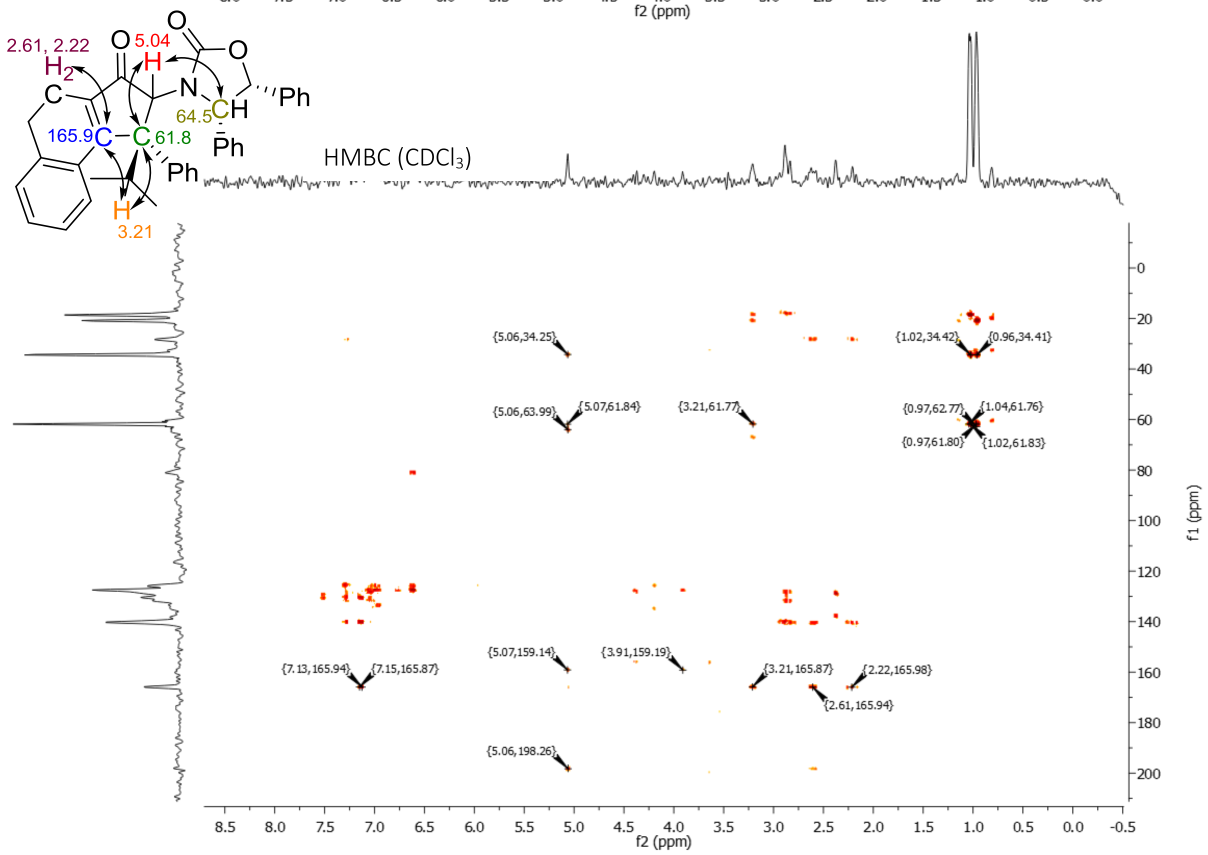

(4*S*,5*R*)-3-((1*S*,2*S*)-1-methyl-3-oxo-1-pentyl-2,3,4,5-tetrahydro-1*H*-cyclopenta[*a*]naphthalen-2-yl)-4,5-diphenyloxazolidin-2-one 7e

^{1}H NMR ($CDCl_3$, 400 MHz)

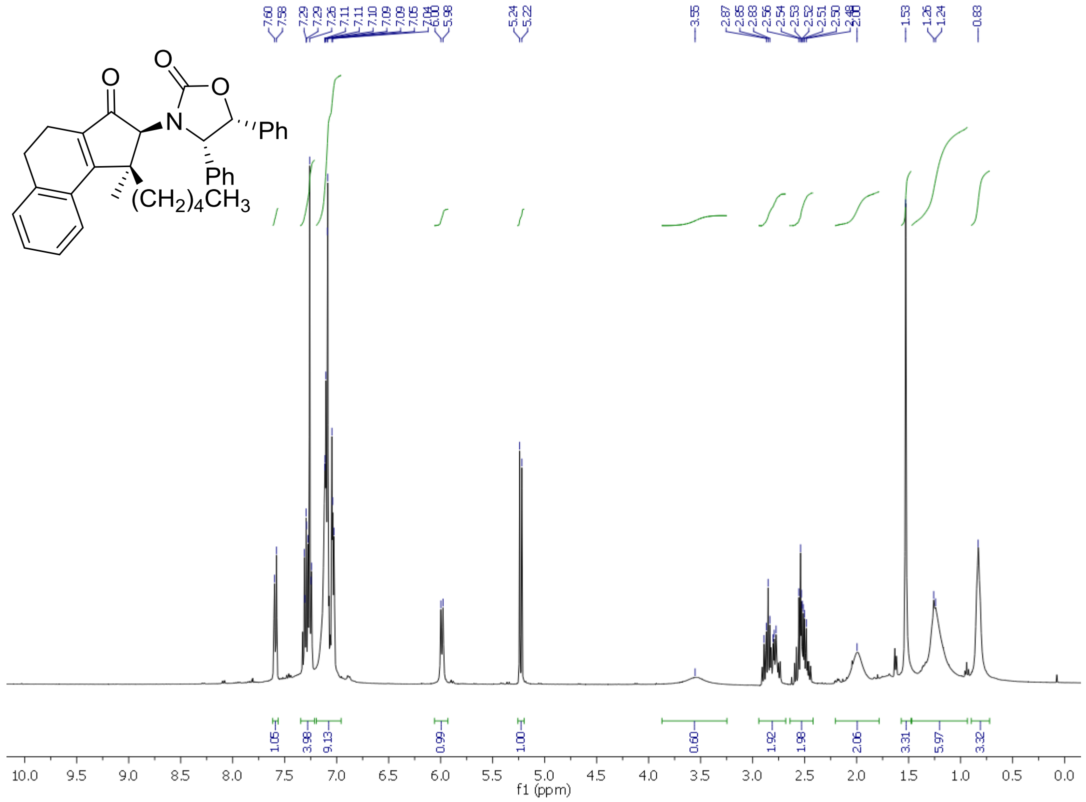

^{13}C NMR ($CDCl_3$, 101 MHz, DEPT-Q)

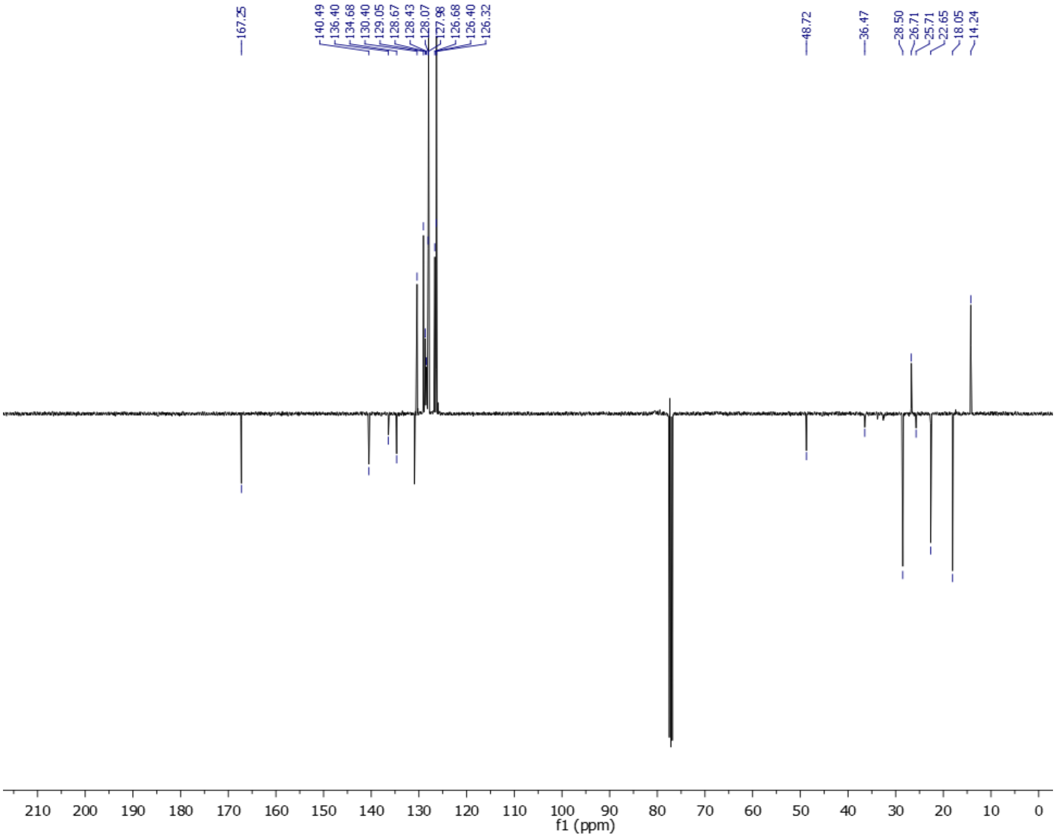

(4*S*,5*R*)-3-((1*S*,2*S*)-1-methyl-3-oxo-1-pentyl-2,3,4,5-tetrahydro-1*H*-cyclopenta[*a*]naphthalen-2-yl)-4,5-diphenyloxazolidin-2-one 7e

NOESY ($CDCl_3$, 400 MHz)

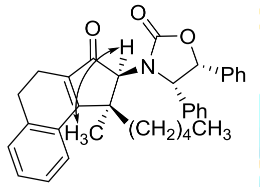

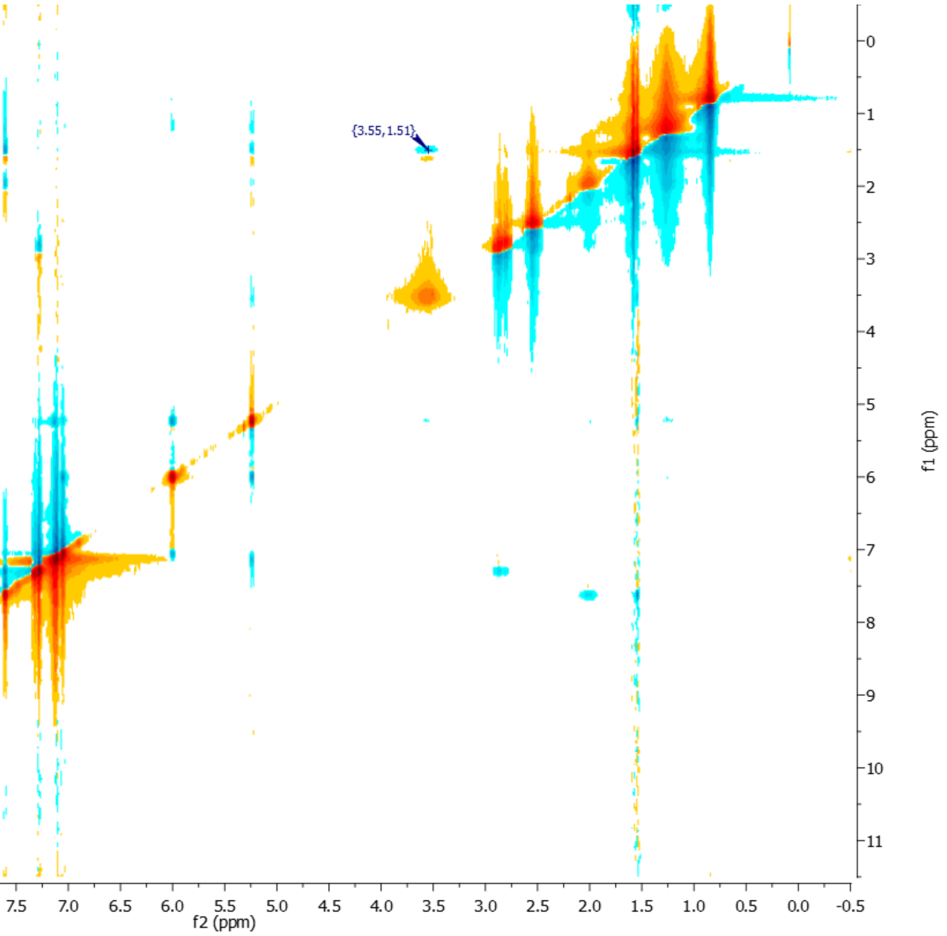

4*S*,5*R*)-3-((1*S*)-5,7-dimethoxy-1-methyl-3-oxo-1-phenyl-2,3-dihydro-1*H*-inden-2-yl)-4,5-diphenyloxazolidin-2-one 7g

^{1}H NMR ($CDCl_3$, 400 MHz)

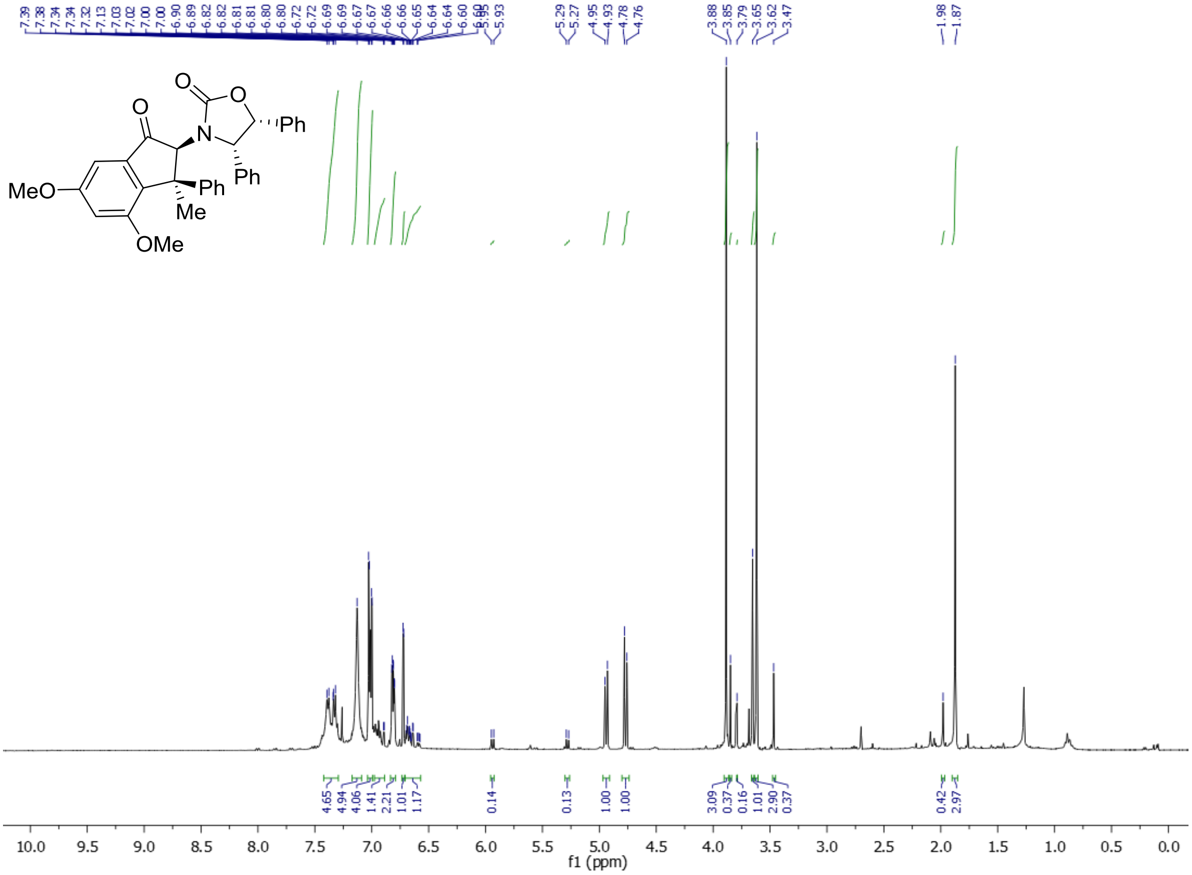

^{13}C NMR ($CDCl_3$, 101 MHz, DEPT-Q)

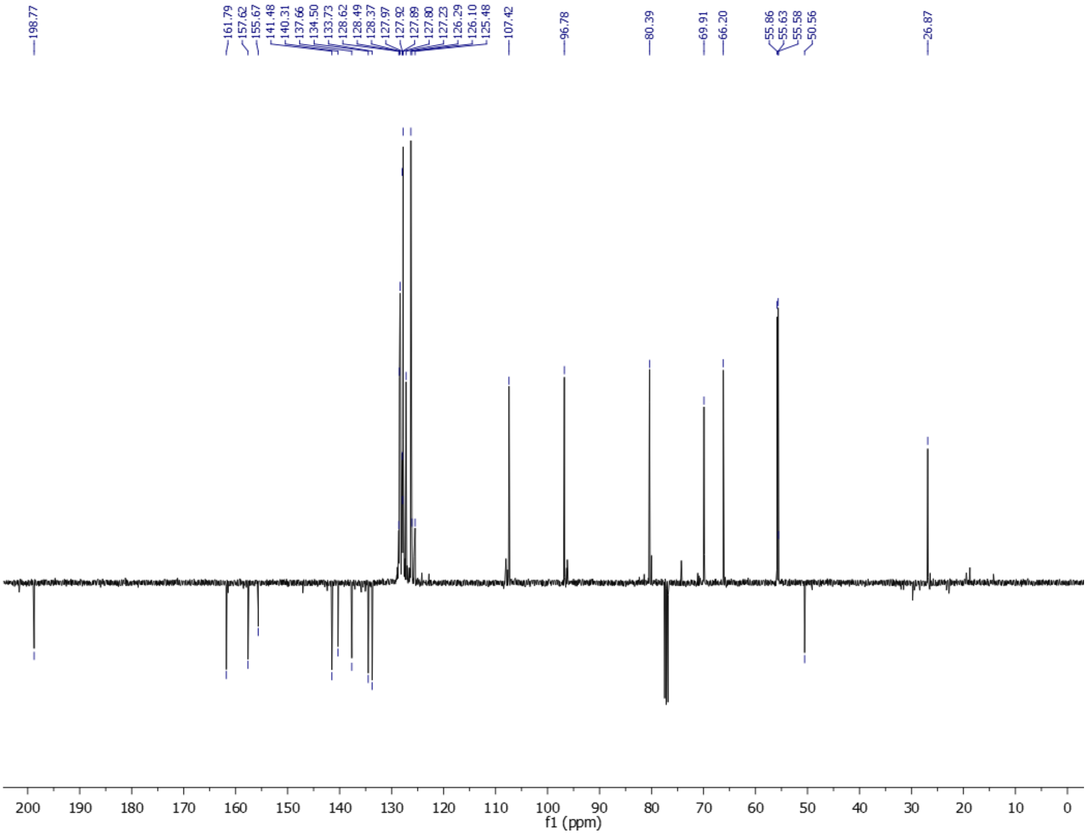

(4*S*,5*R*)-3-((1*S*)-5,7-dimethoxy-1-methyl-3-oxo-1-phenyl-2,3-dihydro-1*H*-inden-2-yl)-4,5-diphenyloxazolidin-2-one 7g

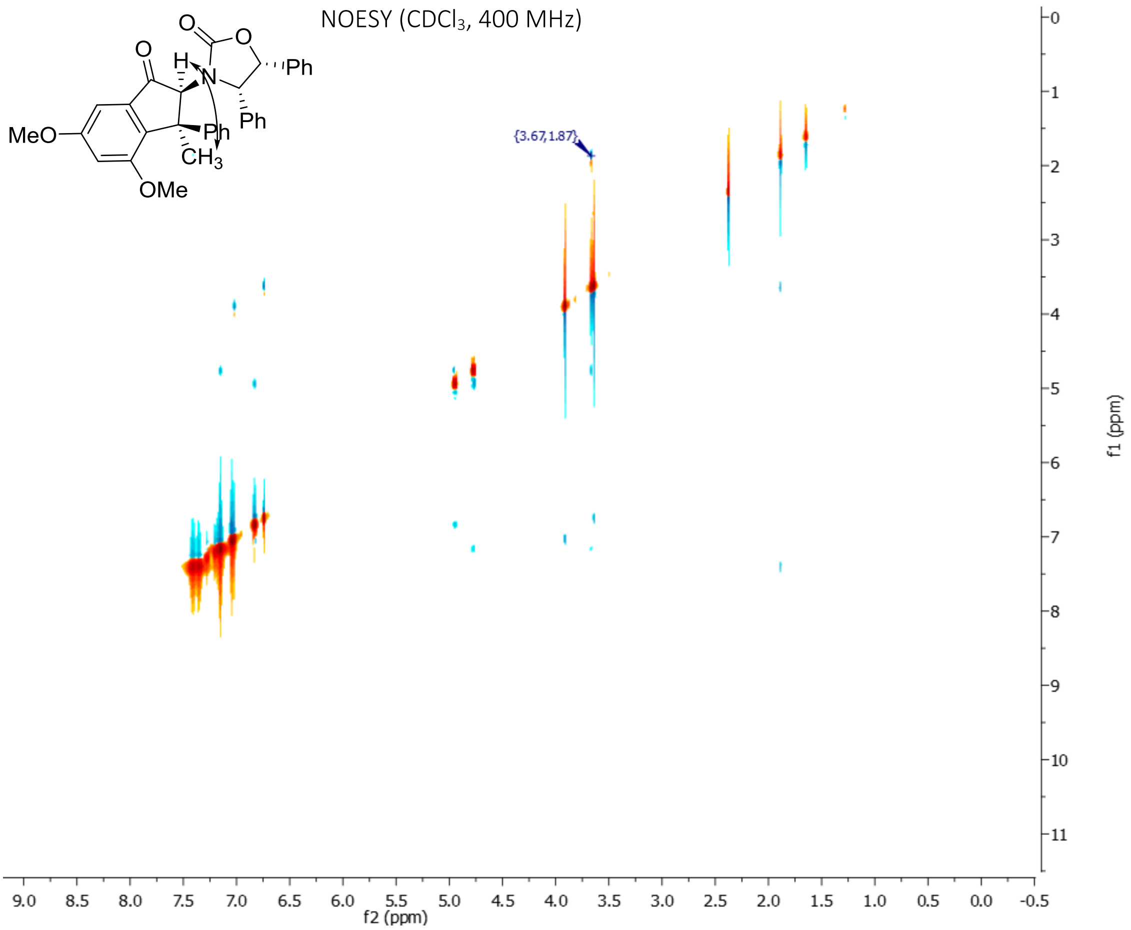

(4*S*,5*R*)-3-((1*S*,2*S*)-1-isopropyl-5,7-dimethoxy-3-oxo-1-phenyl-2,3-dihydro-1*H*-inden-2-yl)-4,5-diphenyloxazolidin-2-one 7h

^{1}H NMR ($CDCl_3$, 400 MHz)

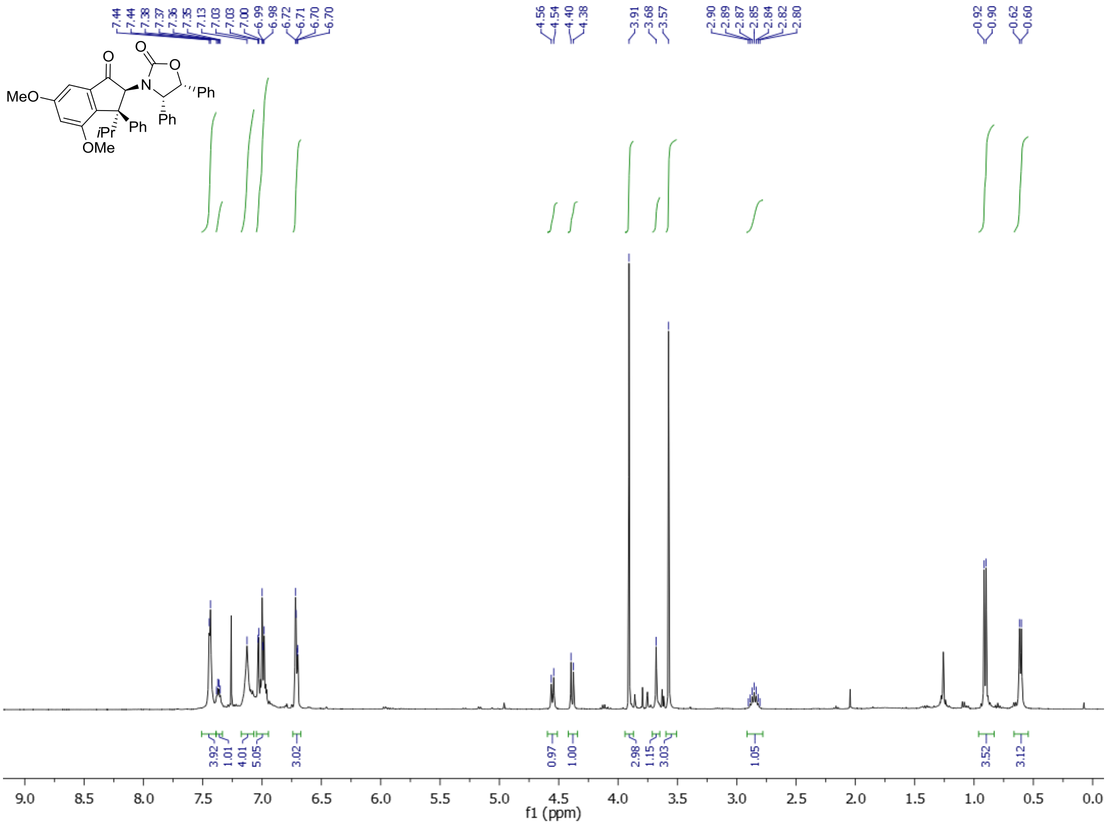

^{13}C NMR ($CDCl_3$, 101 MHz, DEPT-Q)

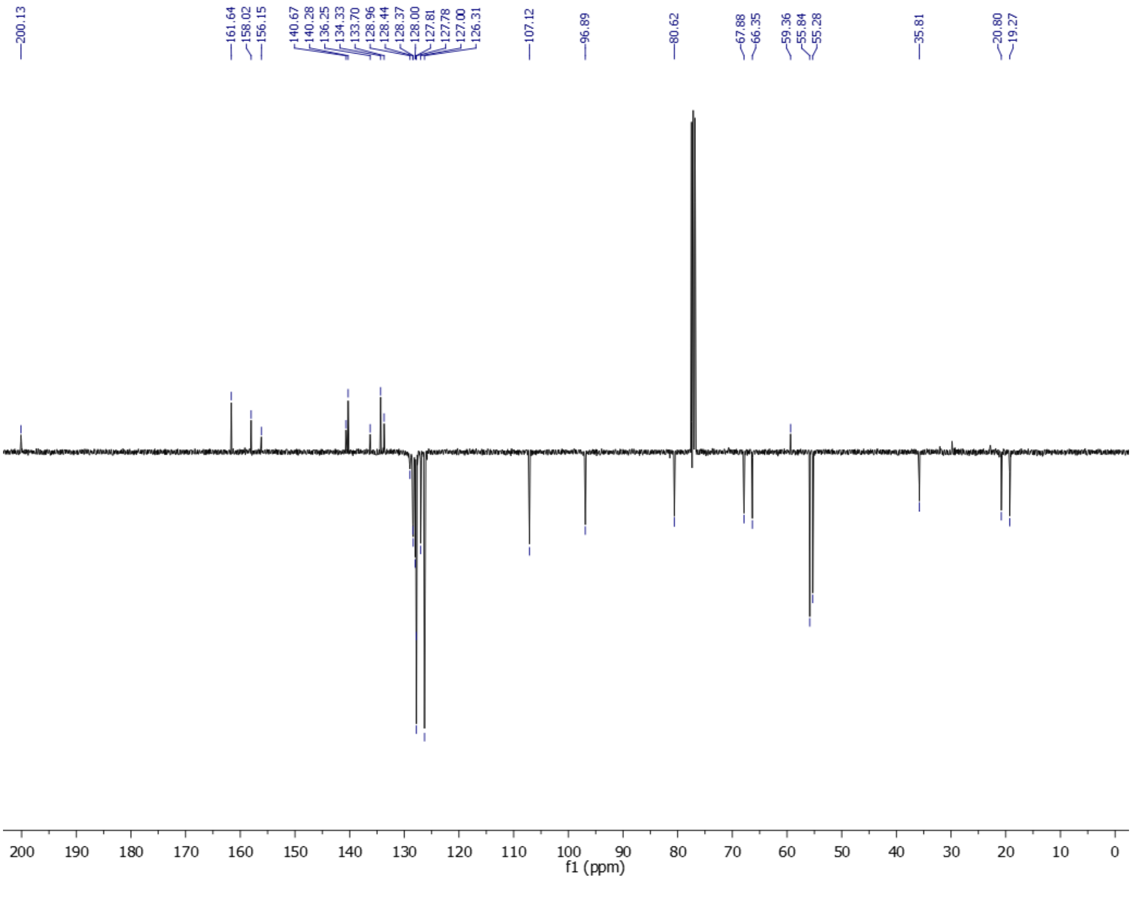

(4*S*,5*R*)-3-((1*S*,2*S*)-1-isopropyl-5,7-dimethoxy-3-oxo-1-phenyl-2,3-dihydro-1*H*-inden-2-yl)-4,5-diphenyloxazolidin-2-one 7h

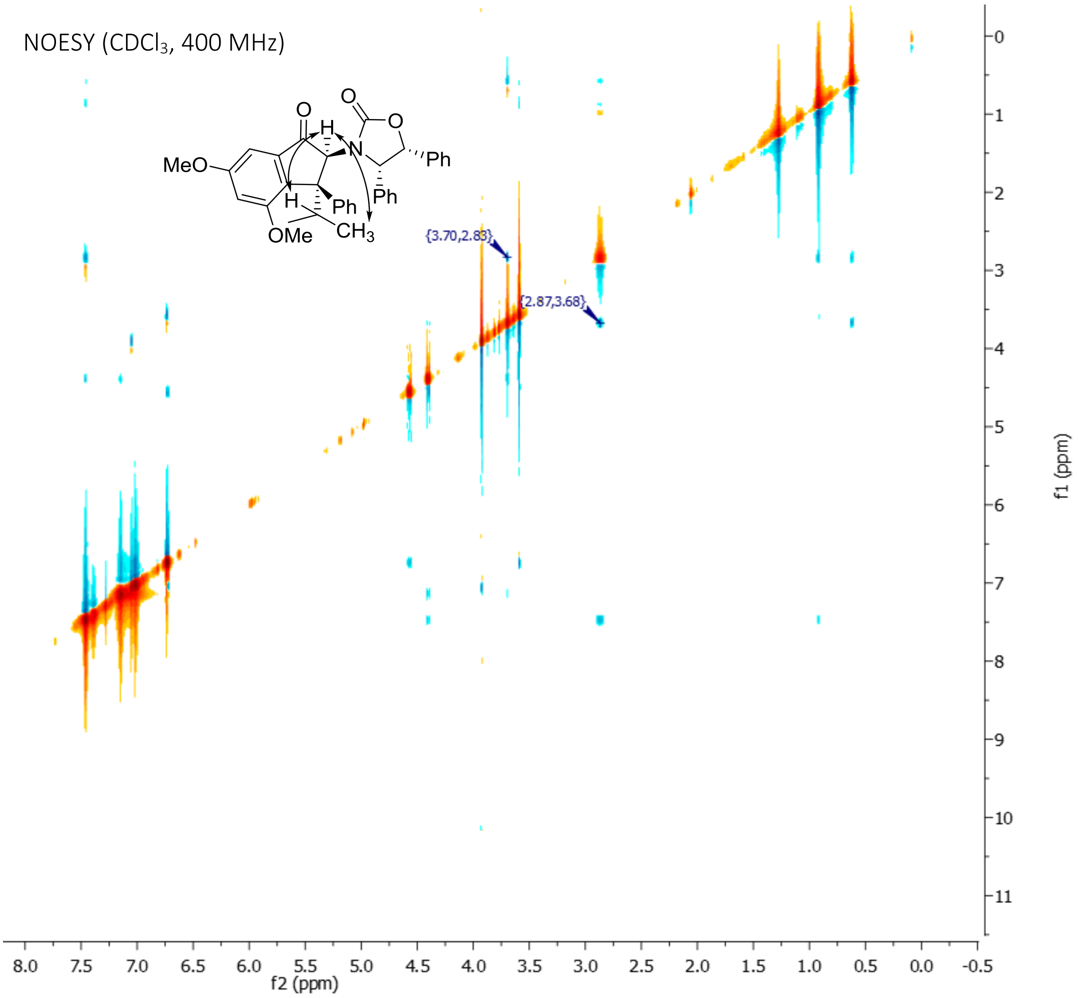

(4*S*,5*R*)-3-((4*S*)-4-methyl-6-oxo-4-phenyl-5,6-dihydro-4*H*-cyclopenta[*b*]thiophen-5-yl)-4,5-diphenyloxazolidin-2-one 7i

^{1}H NMR ($CDCl_3$, 400 MHz)

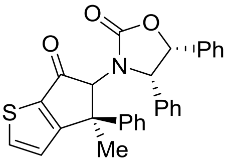

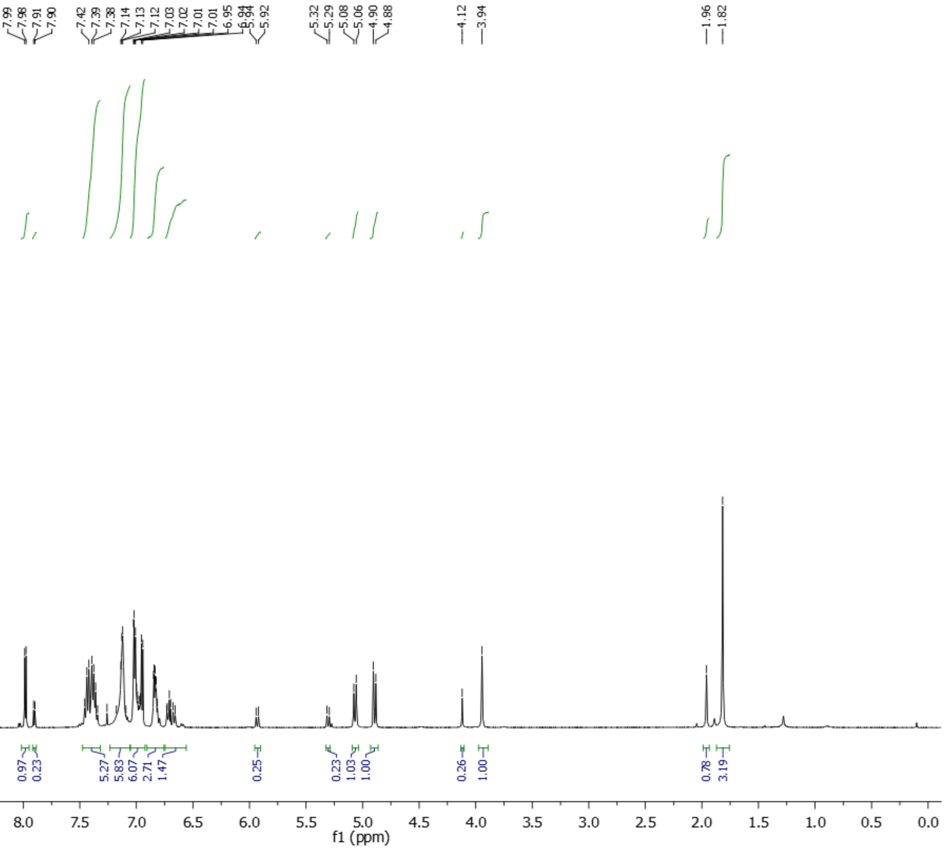

^{13}C NMR ($CDCl_3$, 101 MHz, DEPT-Q)

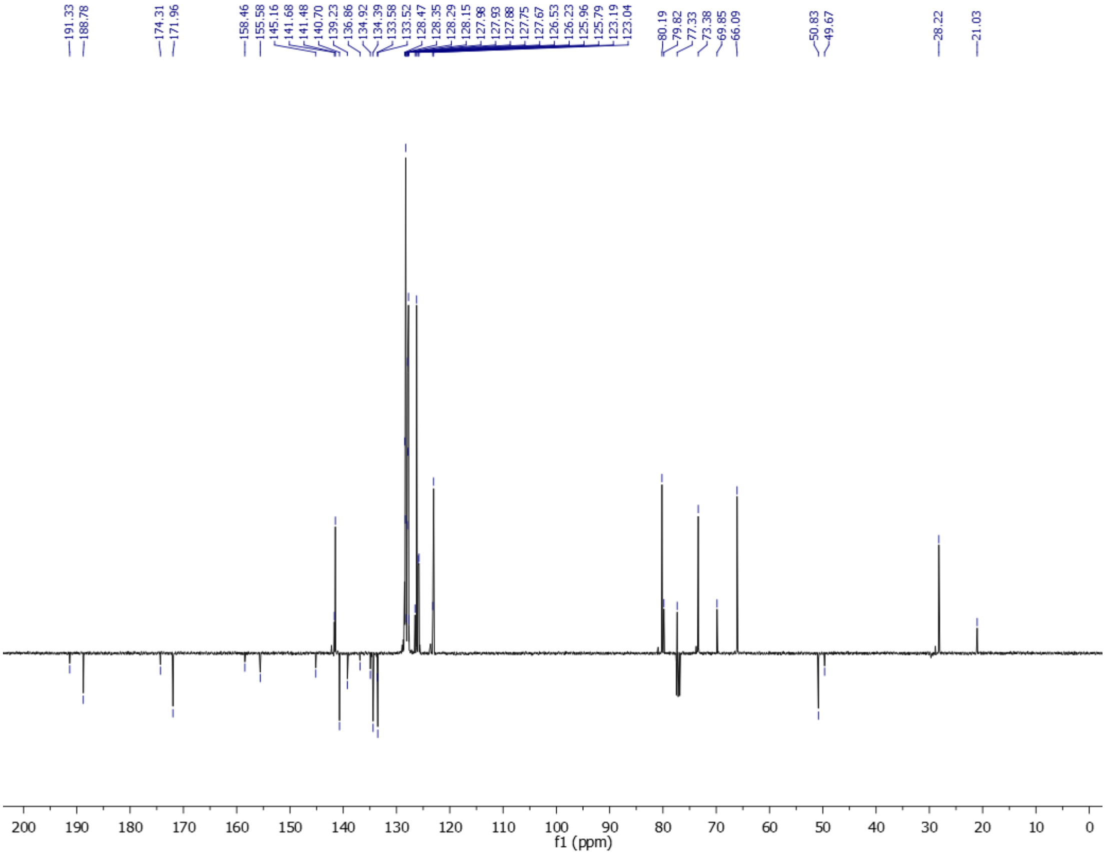

(4*S*,5*R*)-3-(1-(4-methoxyphenyl)-1-oxo-3-phenylbut-3-en-2-yl)-4,5-diphenyloxazolidin-2-one 11

^{1}H NMR ($CDCl_3$, 400 MHz) (crude reaction mixture)

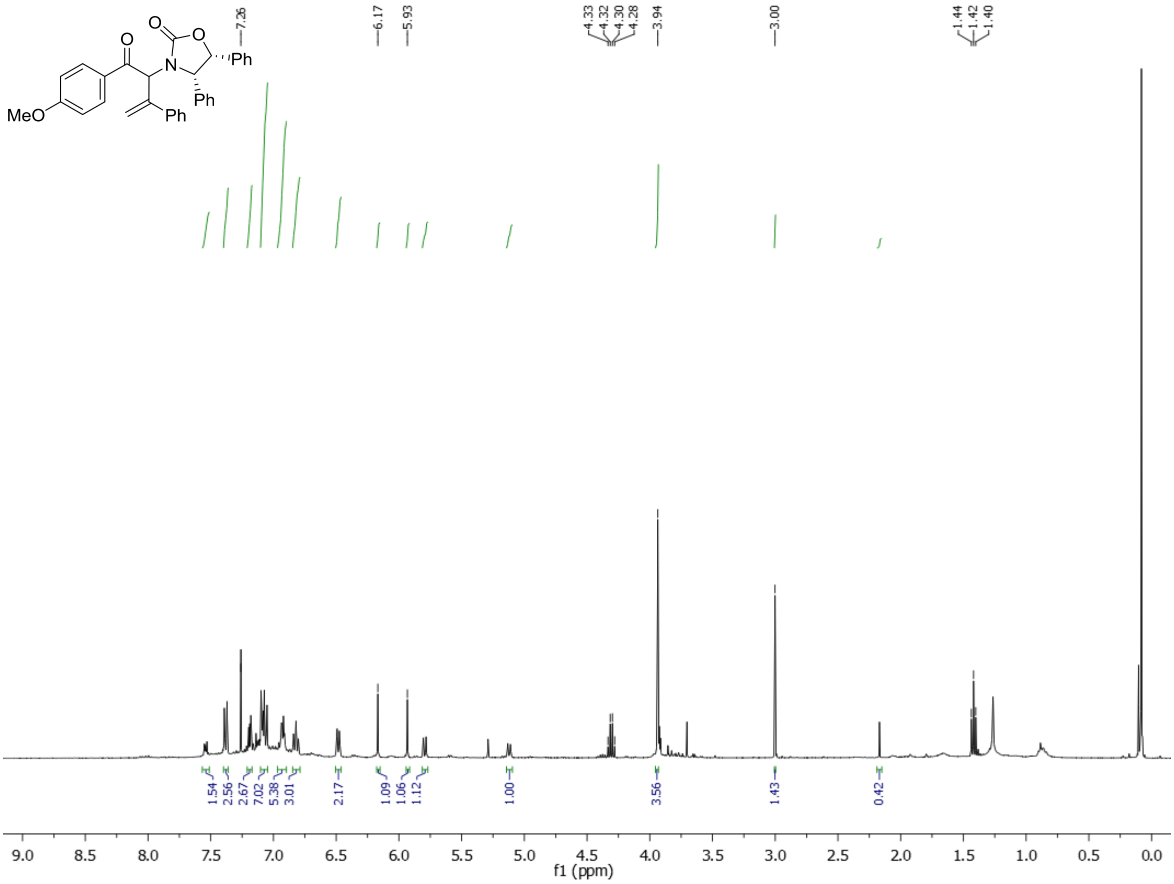

^{13}C NMR ($CDCl_3$, 101 MHz, DEPT-Q)

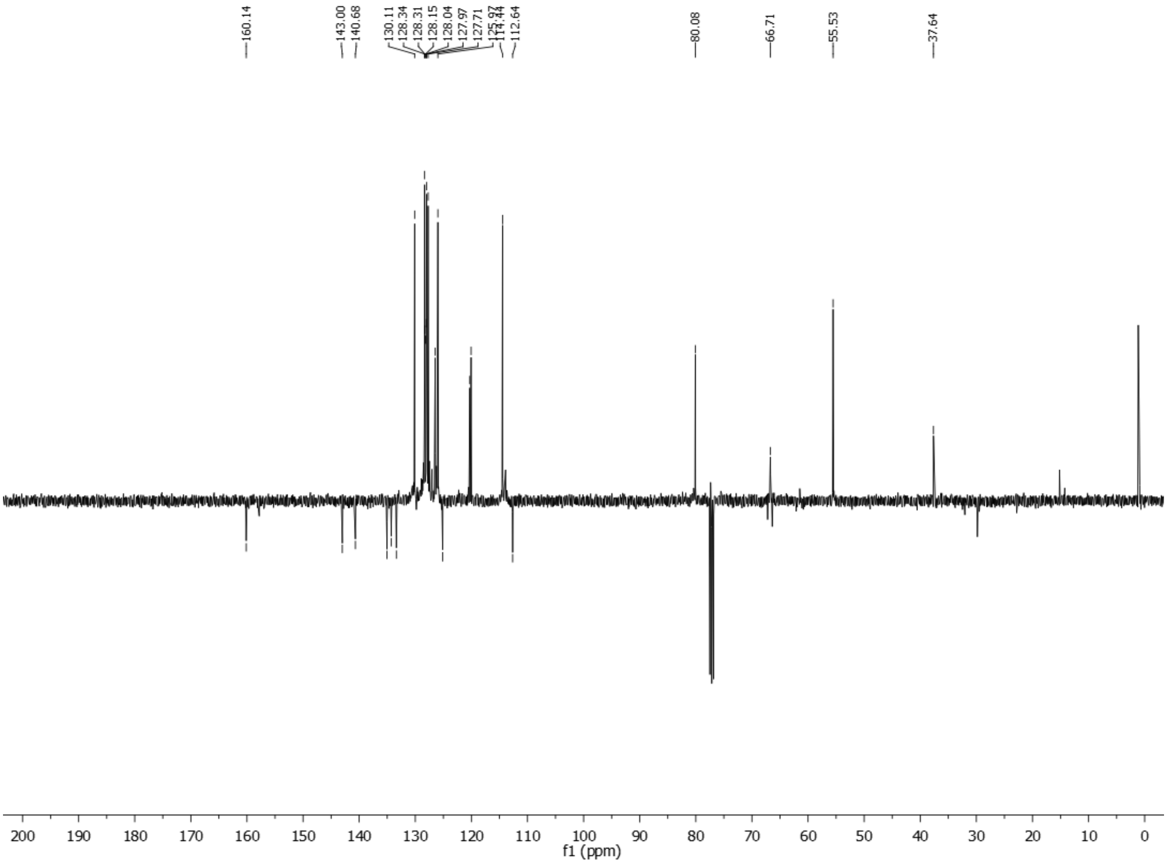

(4*S*,5*R*)-3-(1-(4-methoxyphenyl)-1-oxo-3-phenylbut-3-en-2-yl)-4,5-diphenyloxazolidin-2-one 11

HSQC ($CDCl_3$)

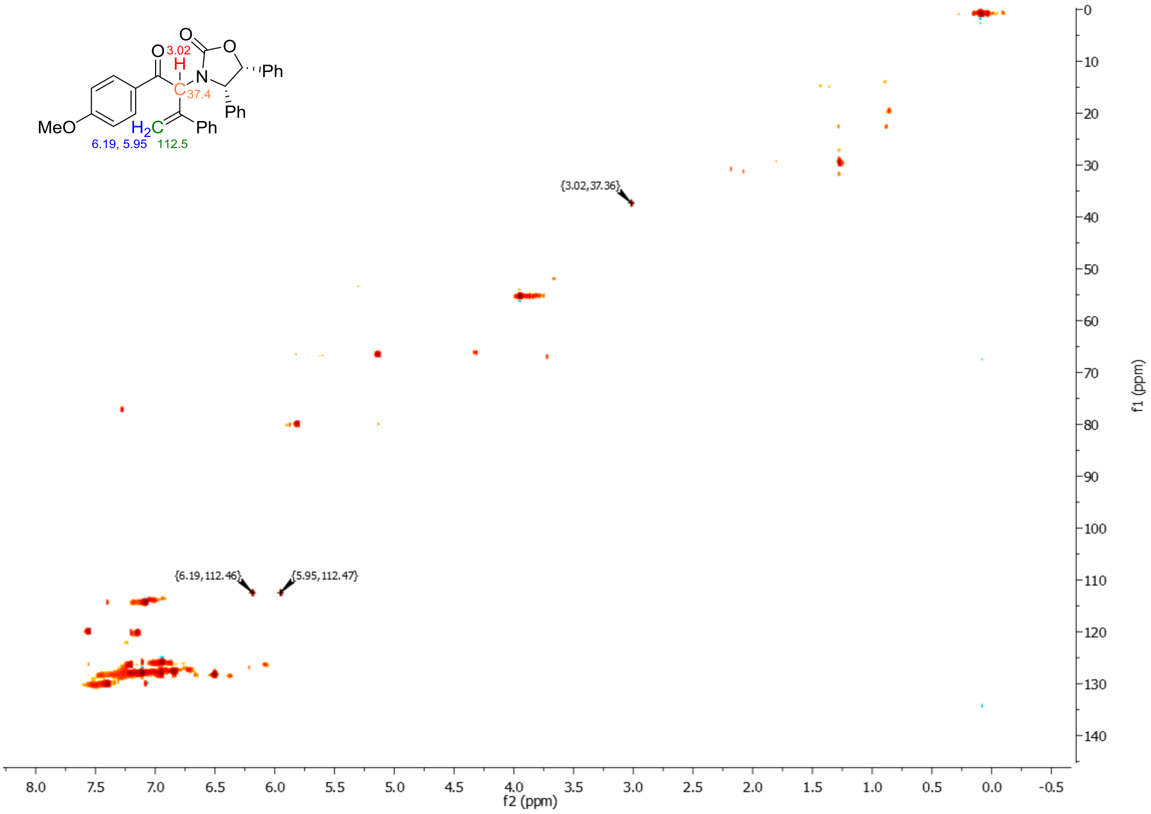

(*S*)-1-methyl-1-phenyl-1,2,4,5-tetrahydro-3*H*-cyclopenta[*a*]naphthalen-3-one 12

^{1}H NMR ($CDCl_3$, 400 MHz)

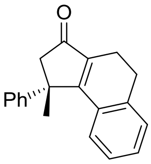

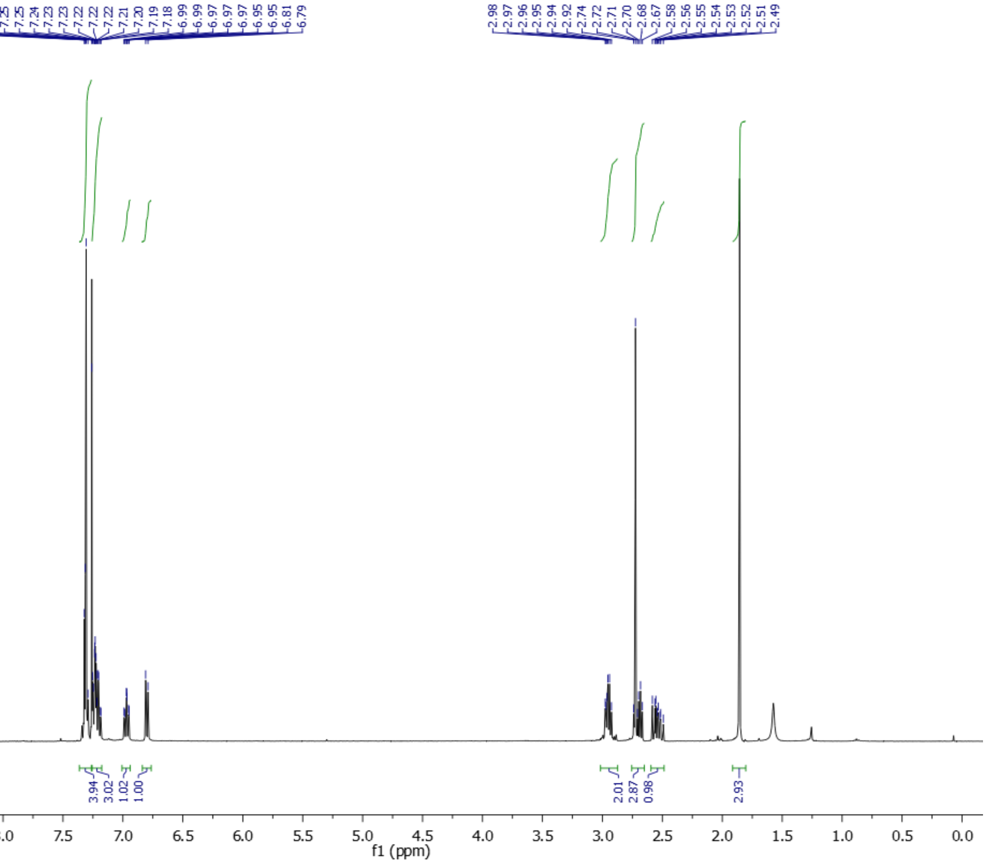

^{13}C NMR ($CDCl_3$, 101 MHz, DEPT-Q)

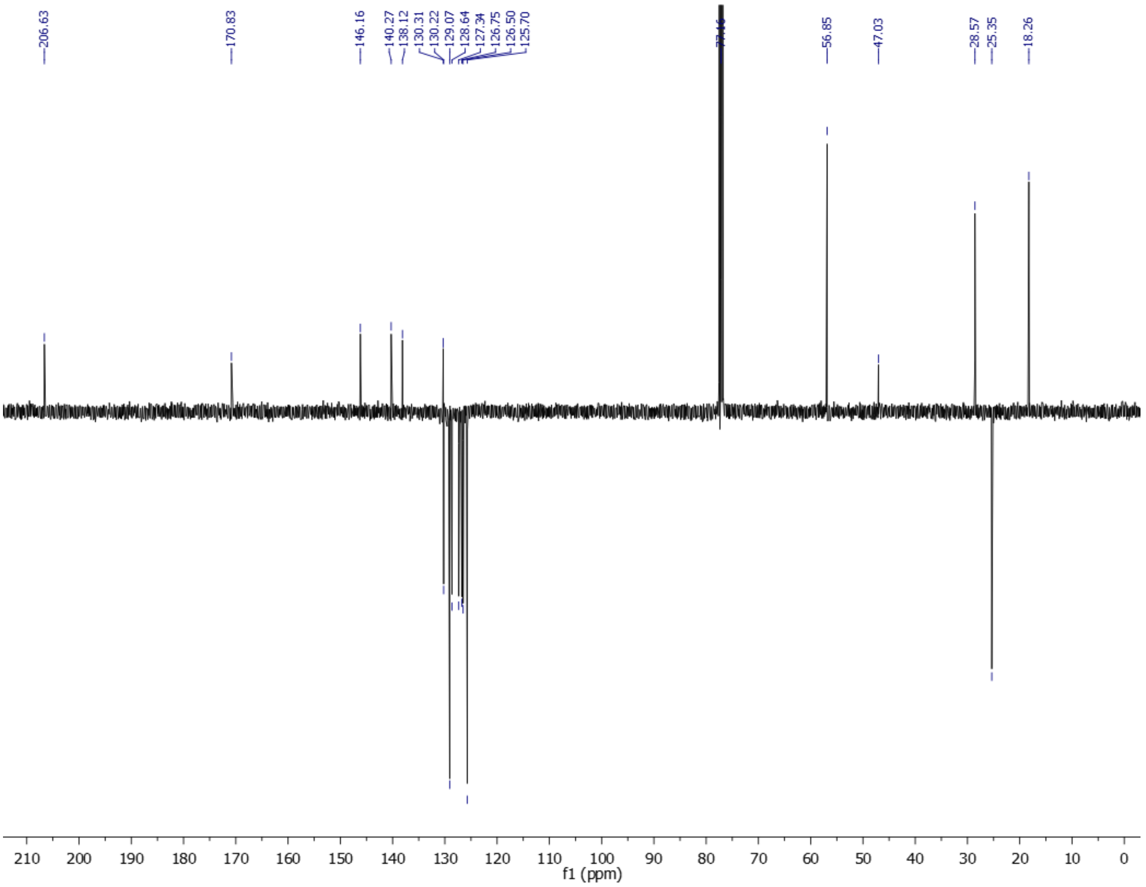

(*S*)-4-methyl-4-phenyl-4,5-dihydro-6*H*-cyclopenta[*b*]thiophen-6-one 13

^{1}H NMR (CDCl$_3$, 400 MHz)

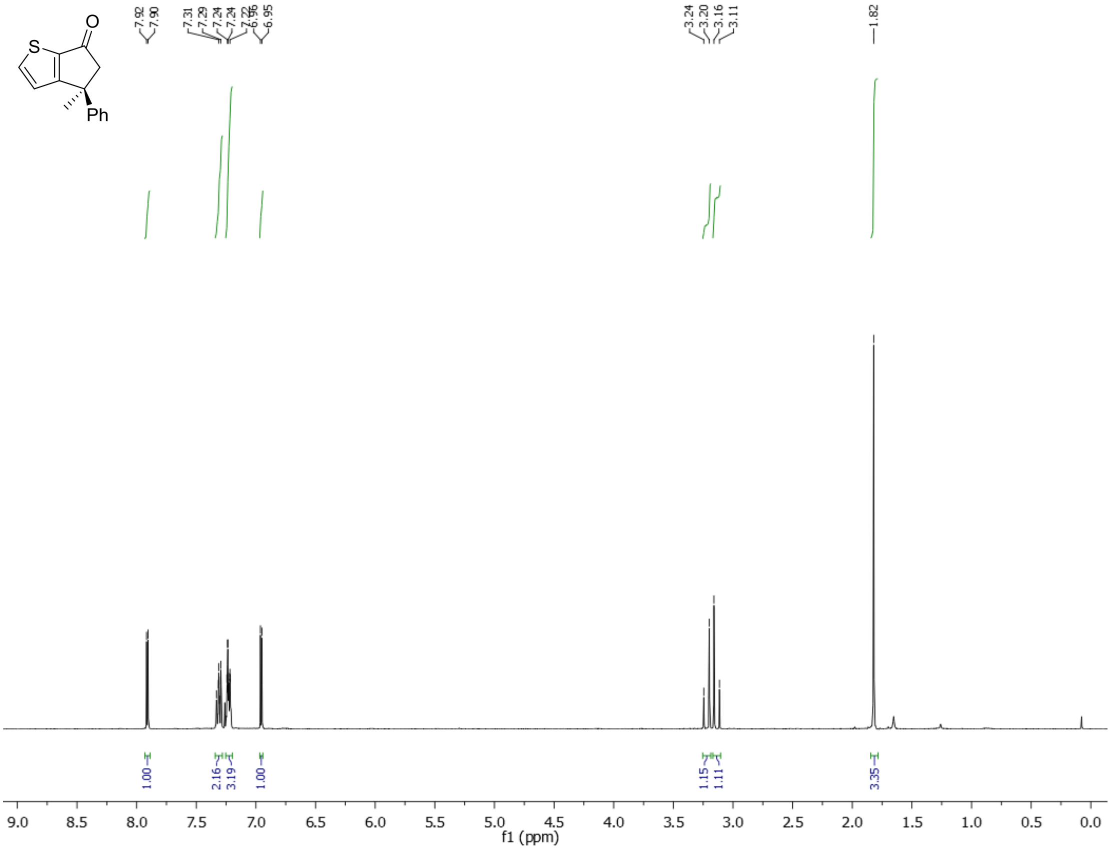

^{13}C NMR (CDCl$_3$, 101 MHz, DEPT-Q)

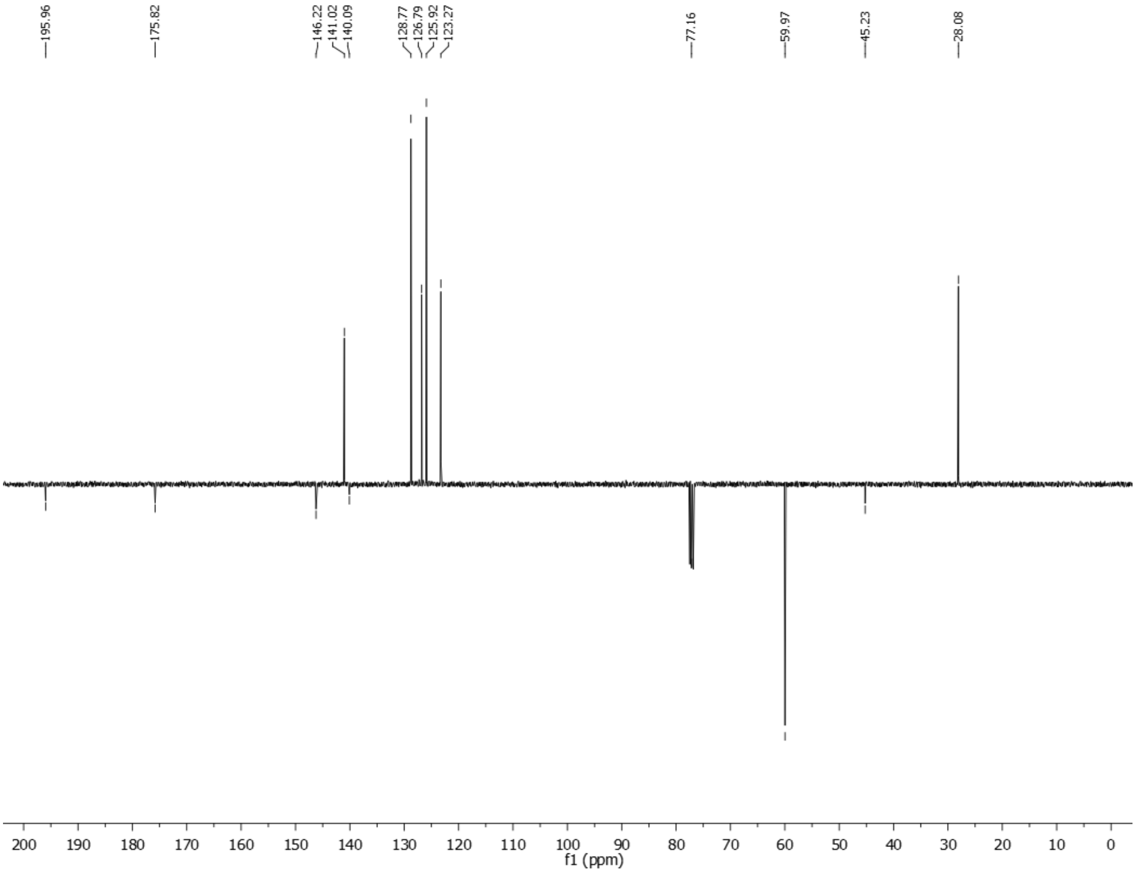

(4*S*,5*R*)-3-((1*R*,2*R*,3a*S*,9b*S*)-1-methyl-3a-(1-methyl-1*H*-indol-3-yl)-3-oxo-1-phenyl-2,3,3a,4,5,9b-hexahydro-1*H*-cyclopenta[*a*]naphthalen-2-yl)-4,5-diphenyloxazolidin-2-one 14

^{1}H NMR ($CDCl_3$, 400 MHz)

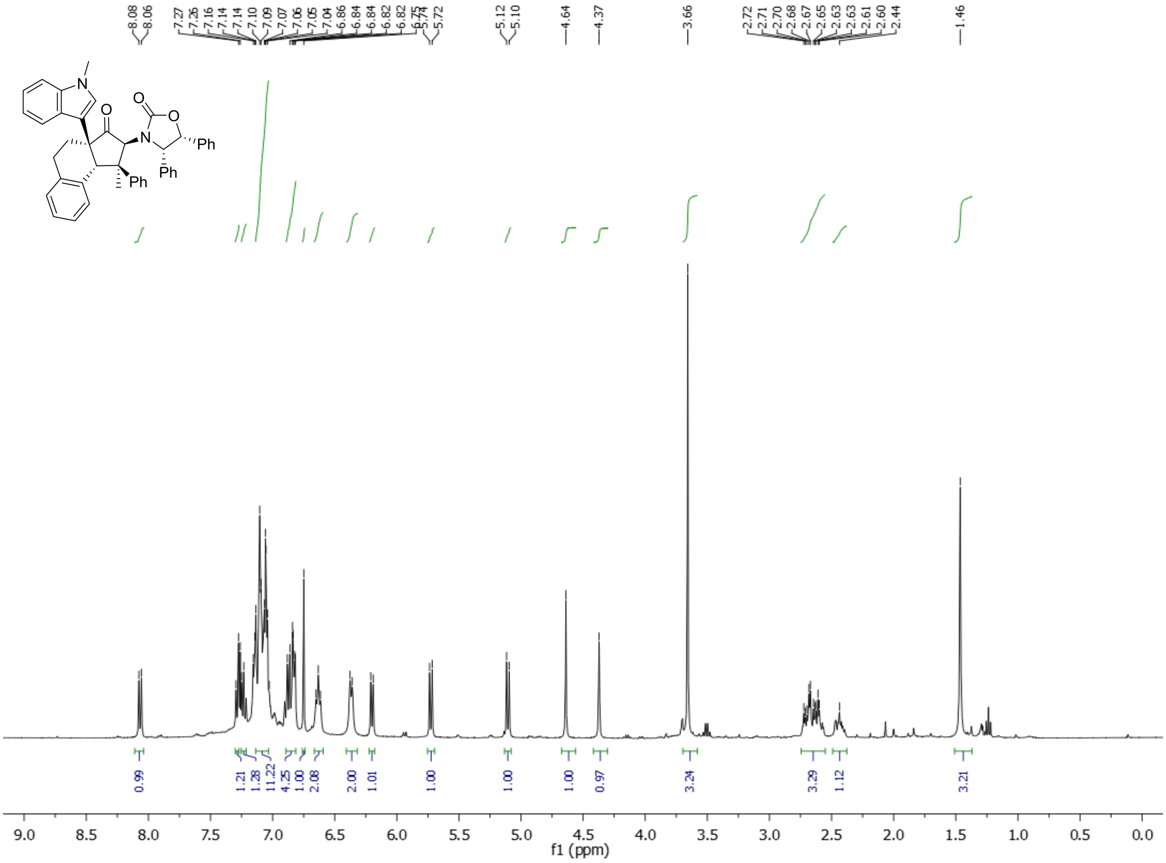

^{13}C NMR ($CDCl_3$, 101 MHz, DEPT-Q)

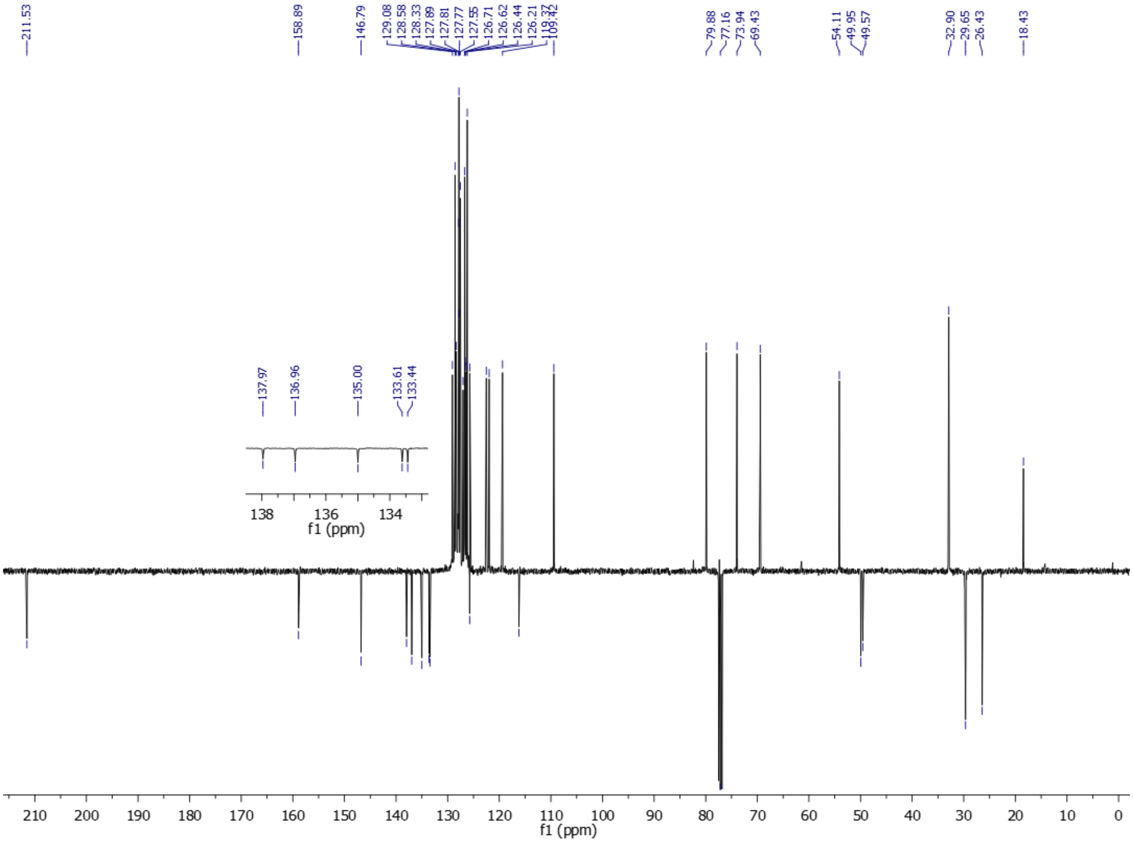

(4*S*,5*R*)-3-((1*R*,2*R*,3a*S*,9b*S*)-1-methyl-3a-(1-methyl-1*H*-indol-3-yl)-3-oxo-1-phenyl-2,3,3a,4,5,9b-hexahydro-1*H*-cyclopenta[*a*]naphthalen-2-yl)-4,5-diphenyloxazolidin-2-one 14

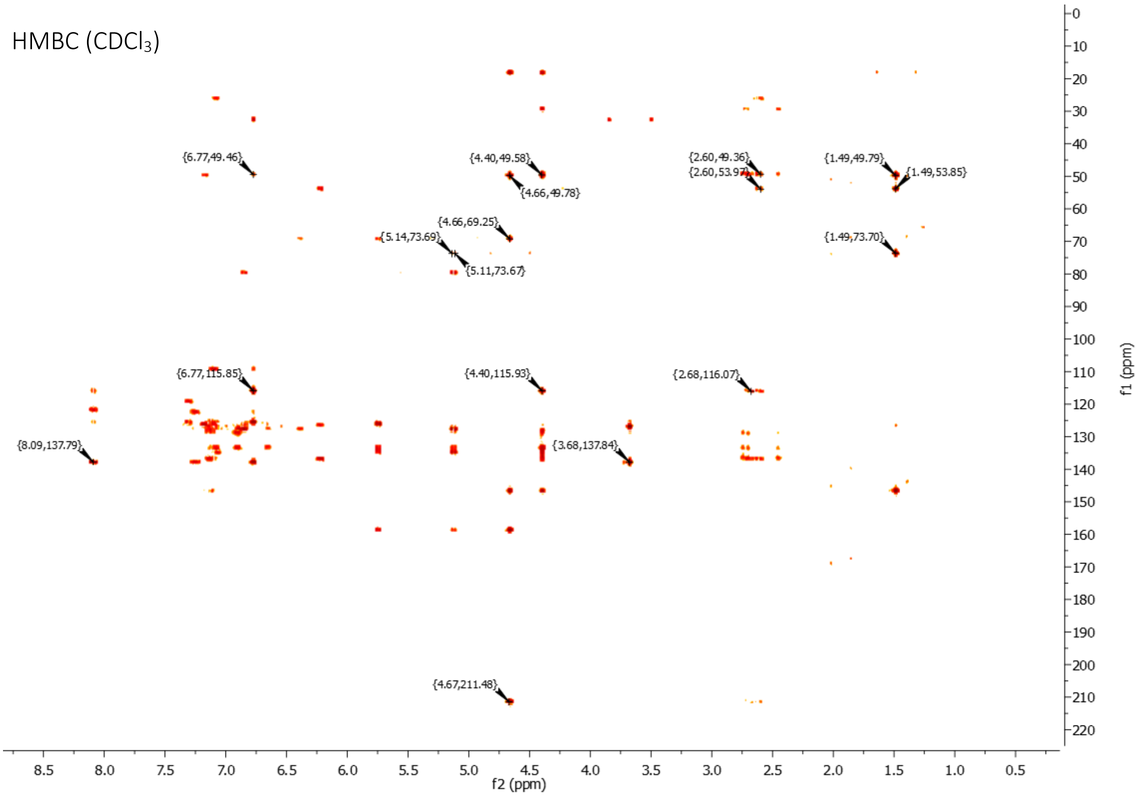

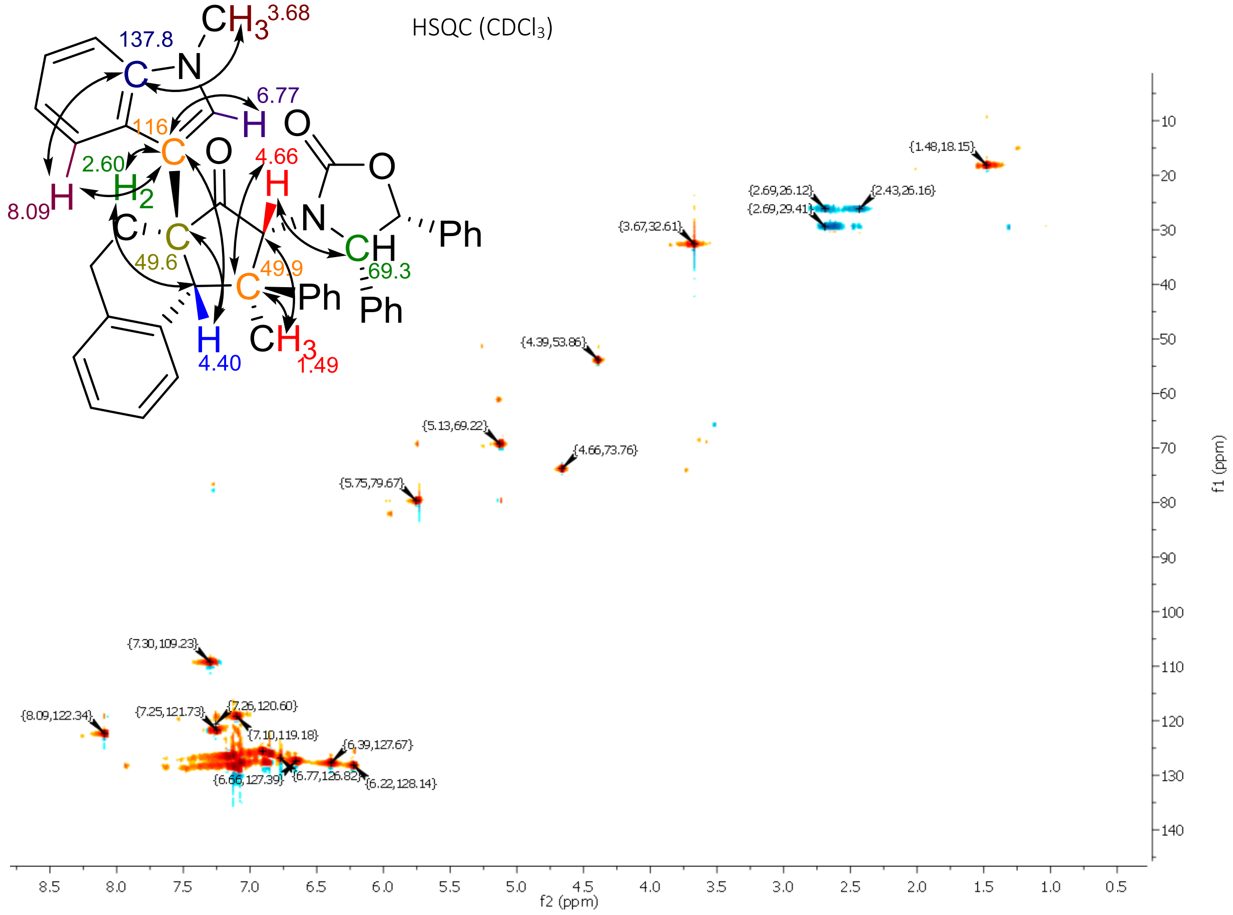

(4*S*,5*R*)-3-((1*R*,2*R*,3a*S*,9b*S*)-1-methyl-3a-(1-methyl-1*H*-indol-3-yl)-3-oxo-1-phenyl-2,3,3a,4,5,9b-hexahydro-1*H*-cyclopenta[*a*]naphthalen-2-yl)-4,5-diphenyloxazolidin-2-one 14

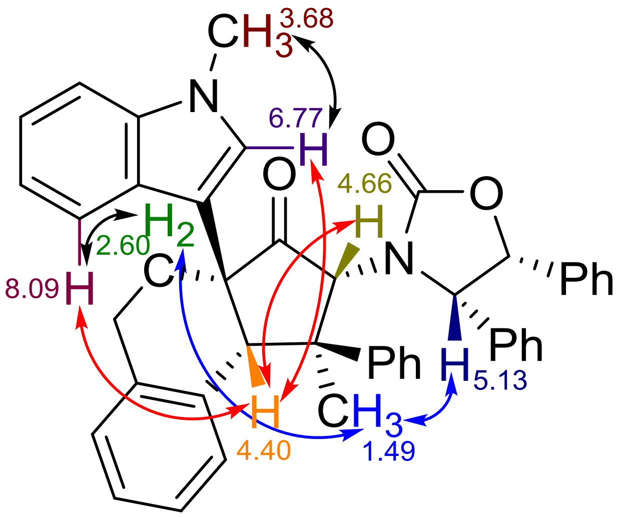

NOESY (CDCl$_3$, 400 MHz)

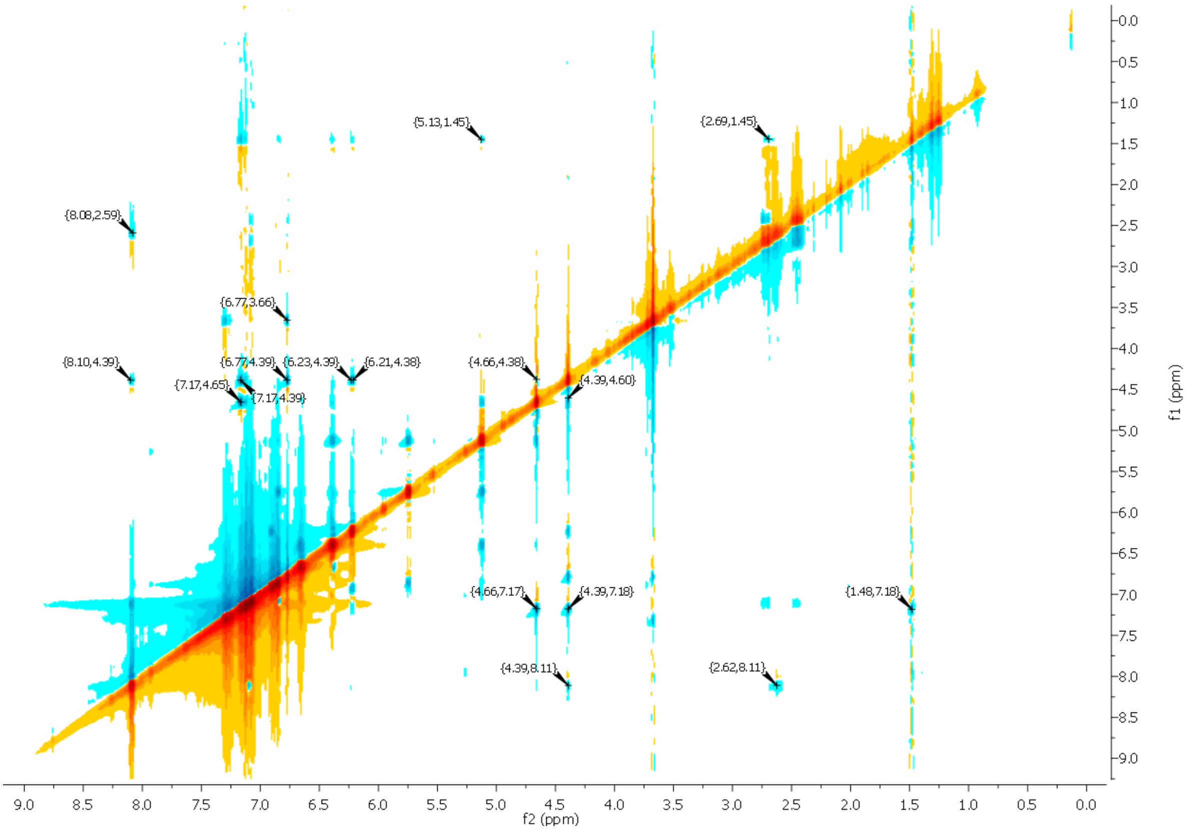

(*Z*)-2-((4*S*,5*R*)-2-oxo-4,5-diphenyloxazolidin-3-yl)-3-phenylbut-2-enal 15

^{1}H NMR ($CDCl_3$, 400 MHz)

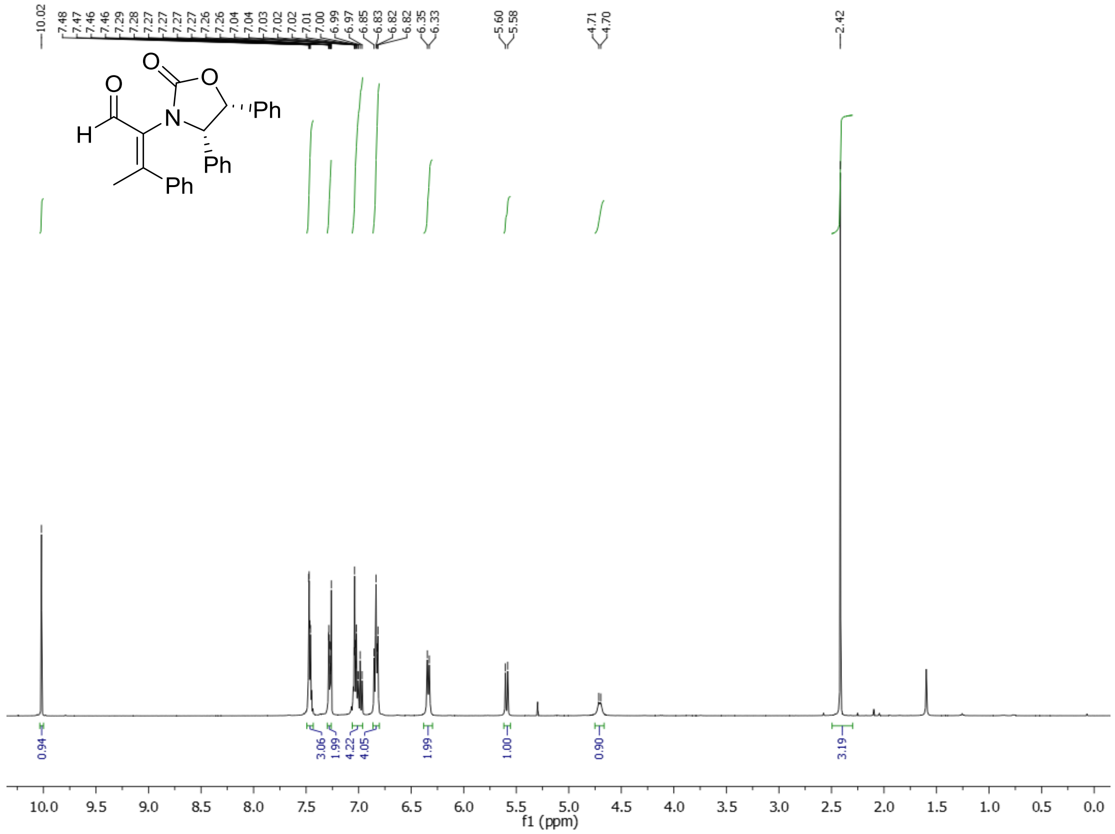

^{13}C NMR ($CDCl_3$, 101 MHz, DEPT-Q)

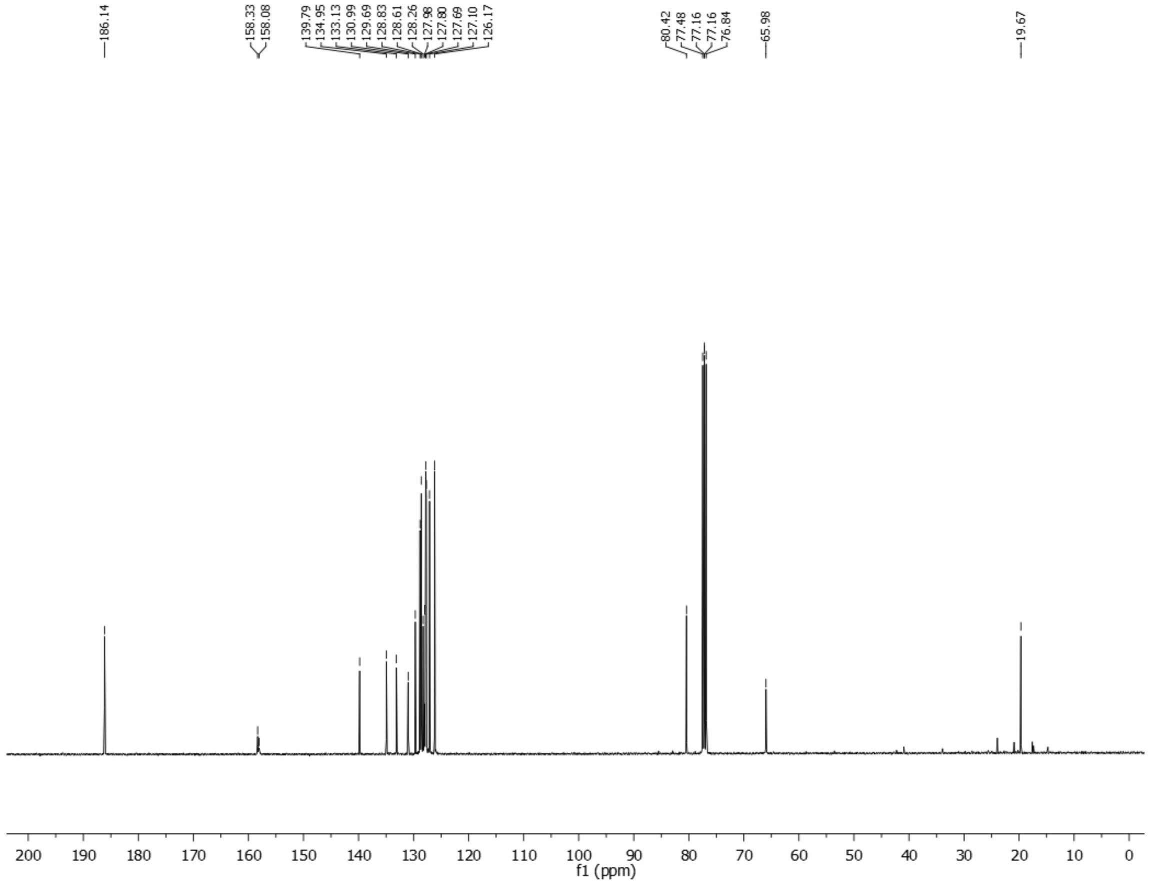

(4*S*,5*R*)-3-((2*Z*,5*E*)-5-ethyl-6-methyl-4-oxo-2-phenylocta-2,5-dien-3-yl)-4,5-diphenyloxazolidin-2-one (2*Z*,5*E*)-17

^{1}H NMR ($CDCl_3$, 400 MHz)

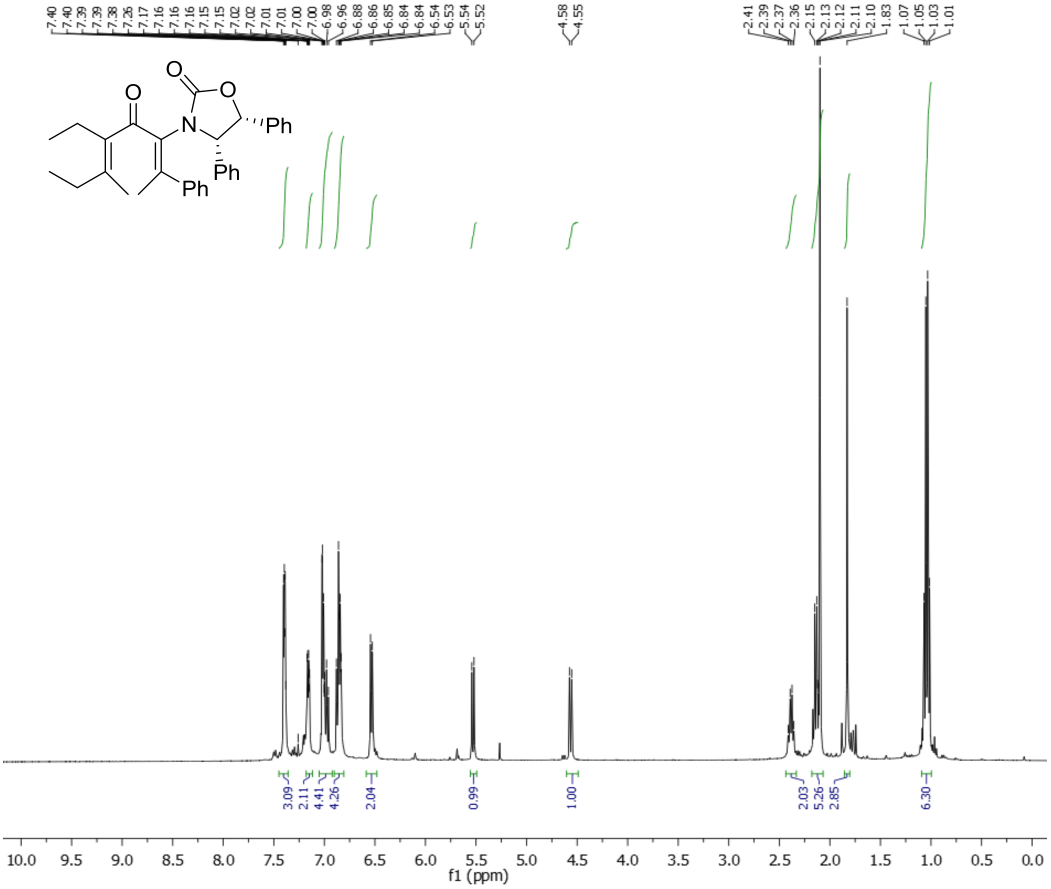

^{13}C NMR ($CDCl_3$, 101 MHz, DEPT-Q)

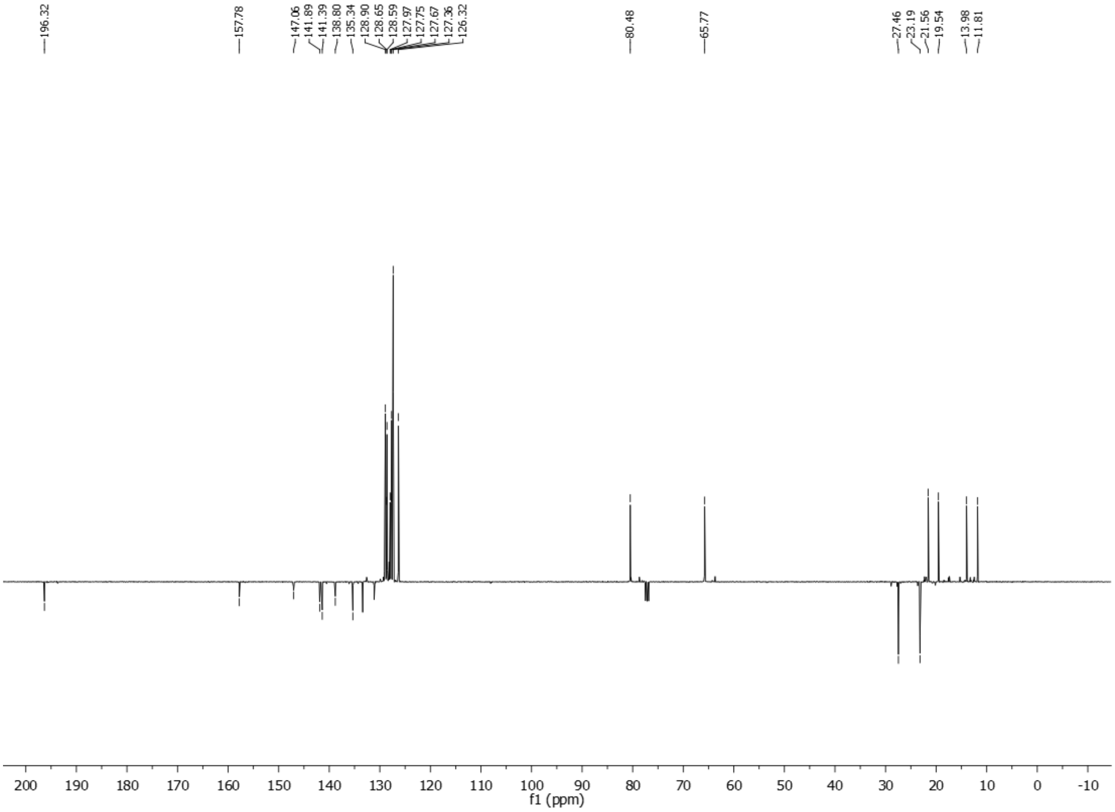

(4*S*,5*R*)-3-((2*Z*,5*E*)-5-ethyl-6-methyl-4-oxo-2-phenylocta-2,5-dien-3-yl)-4,5-diphenyloxazolidin-2-one (2*Z*,5*E*)-17

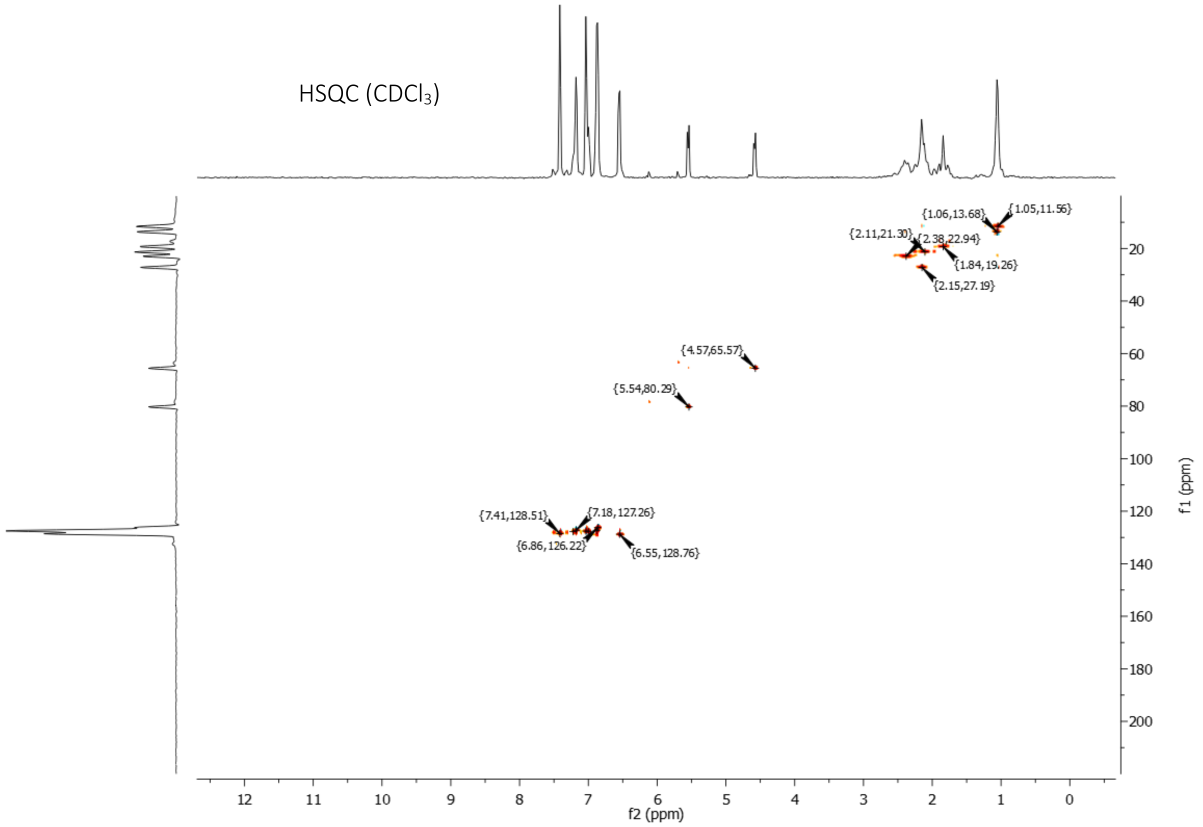

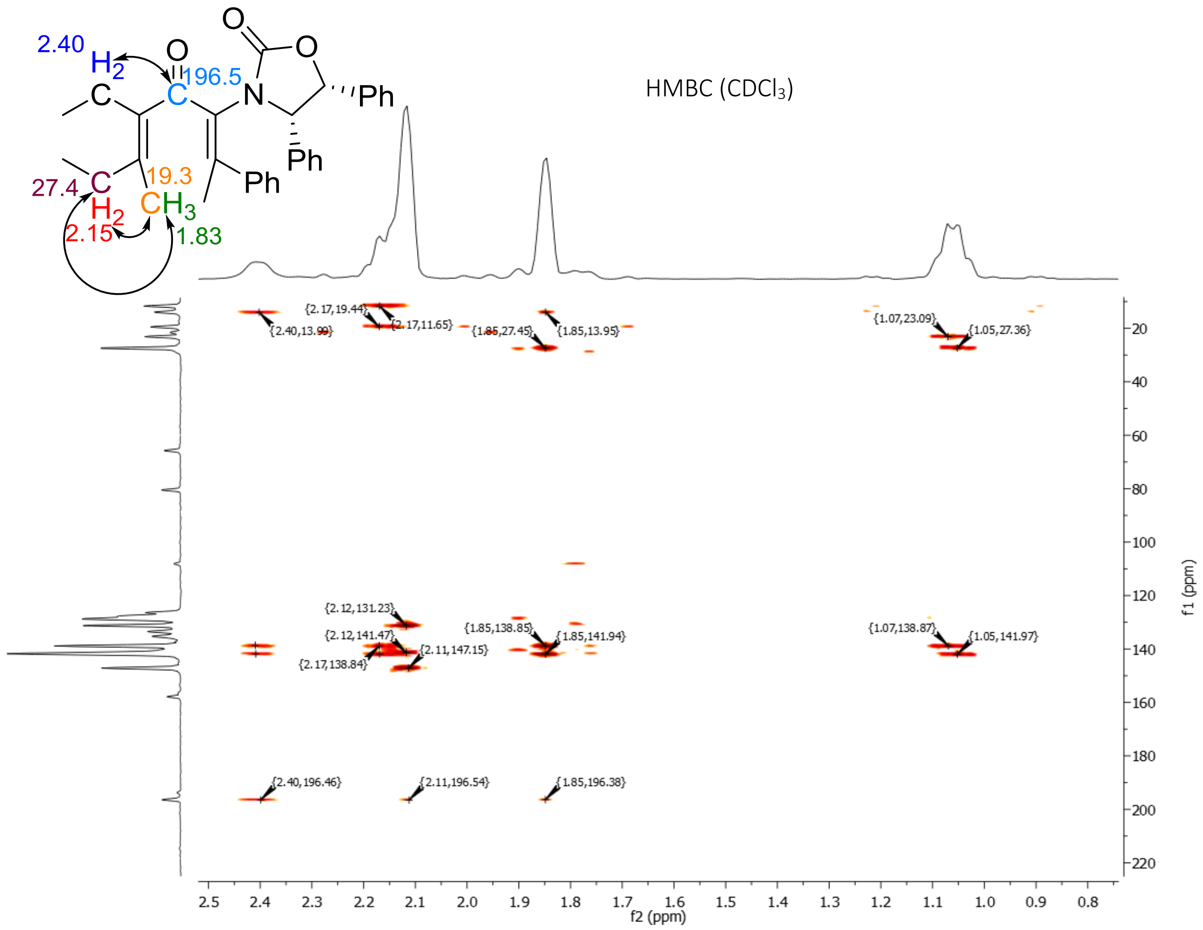

(4*S*,5*R*)-3-((2*Z*,5*E*)-5-ethyl-6-methyl-4-oxo-2-phenylocta-2,5-dien-3-yl)-4,5-diphenyloxazolidin-2-one (2*Z*,5*E*)-17

NOESY ($CDCl_3$, 400 MHz)

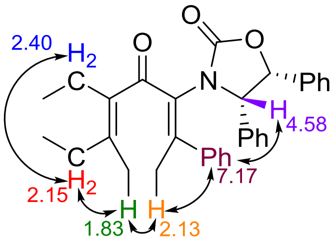

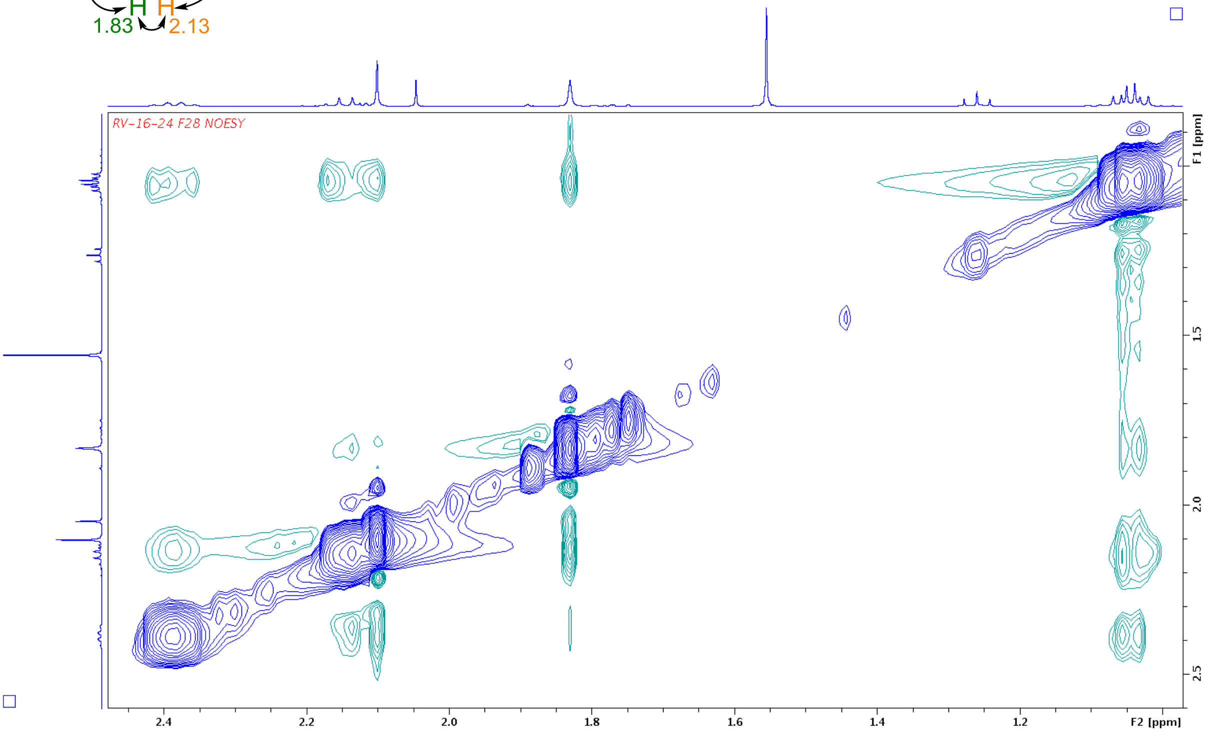

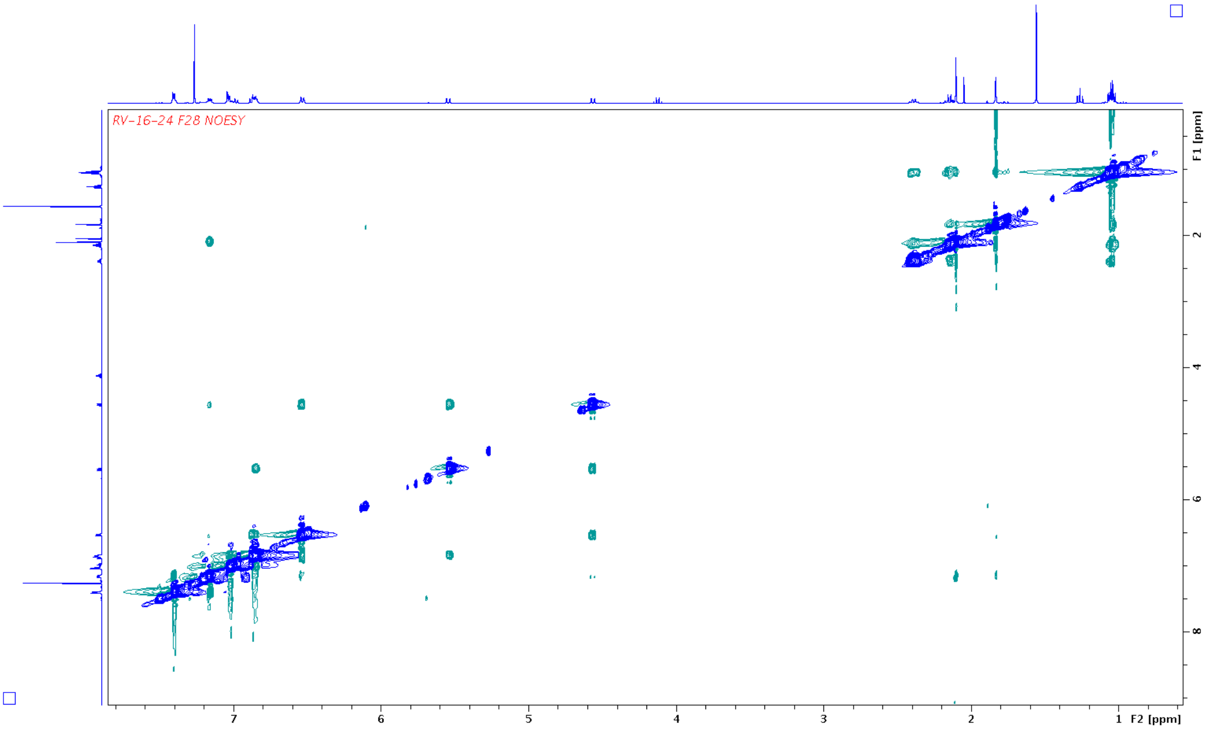

(4*S*,5*R*)-3-((2*Z*,5*Z*)-5-ethyl-6-methyl-4-oxo-2-phenylocta-2,5-dien-3-yl)-4,5-diphenyloxazolidin-2-one (2Z,5Z)-17

^{1}H NMR ($CDCl_3$, 400 MHz)

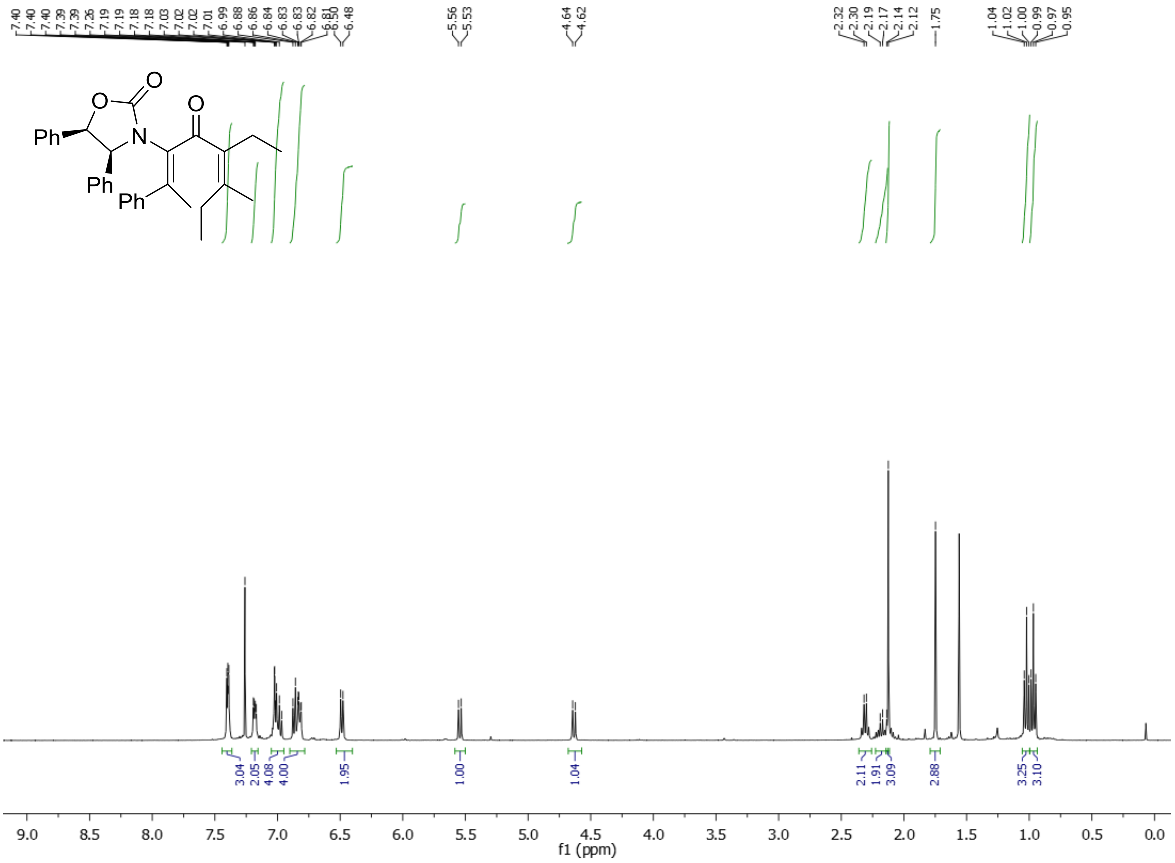

^{13}C NMR ($CDCl_3$, 101 MHz, DEPT-Q)

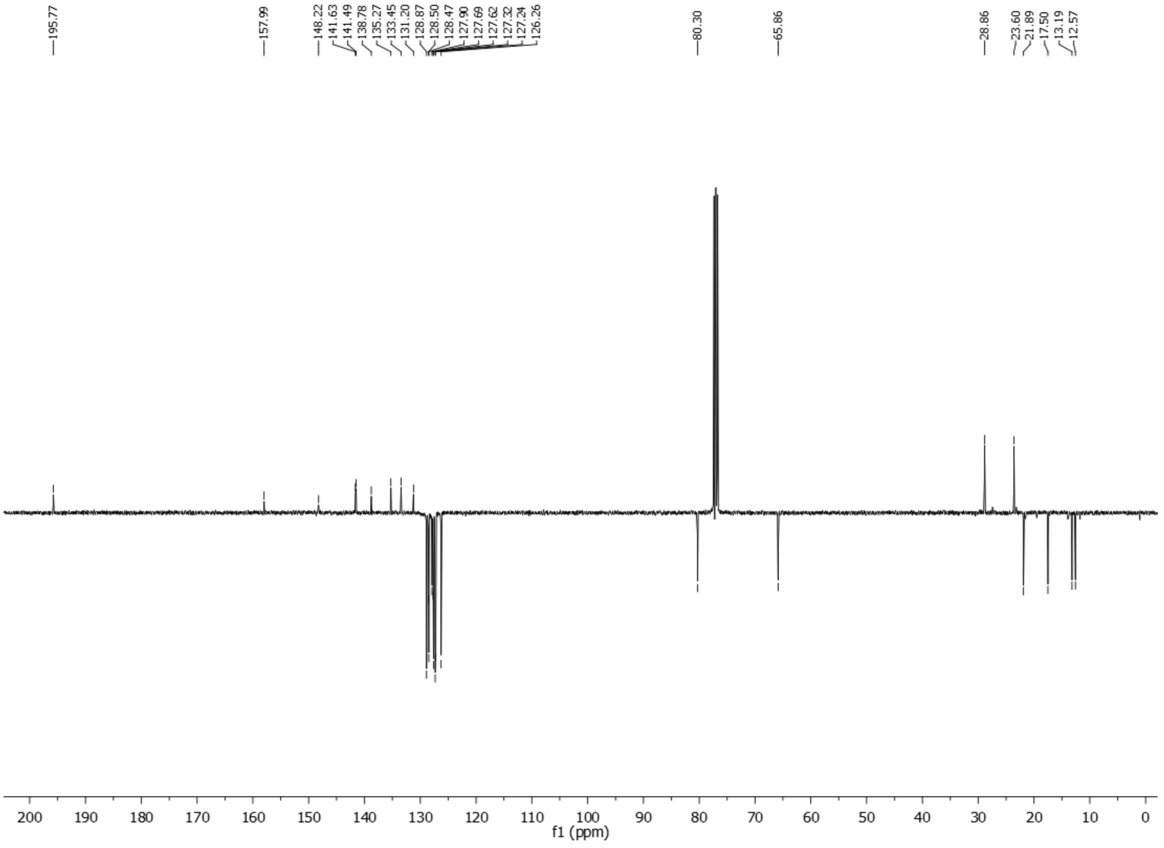

(4*S*,5*R*)-3-((2*Z*,5*Z*)-5-ethyl-6-methyl-4-oxo-2-phenylocta-2,5-dien-3-yl)-4,5-diphenyloxazolidin-2-one (2Z,5Z)-17

HMBC ($CDCl_3$)

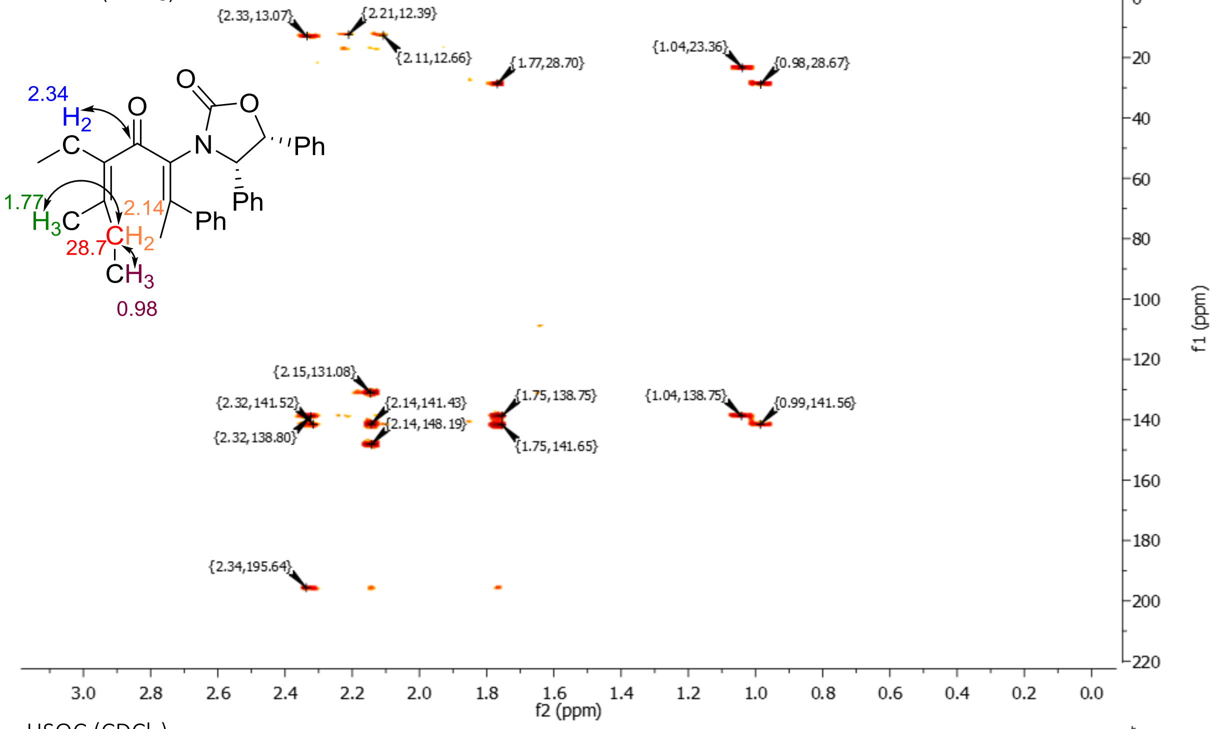

HSQC ($CDCl_3$)

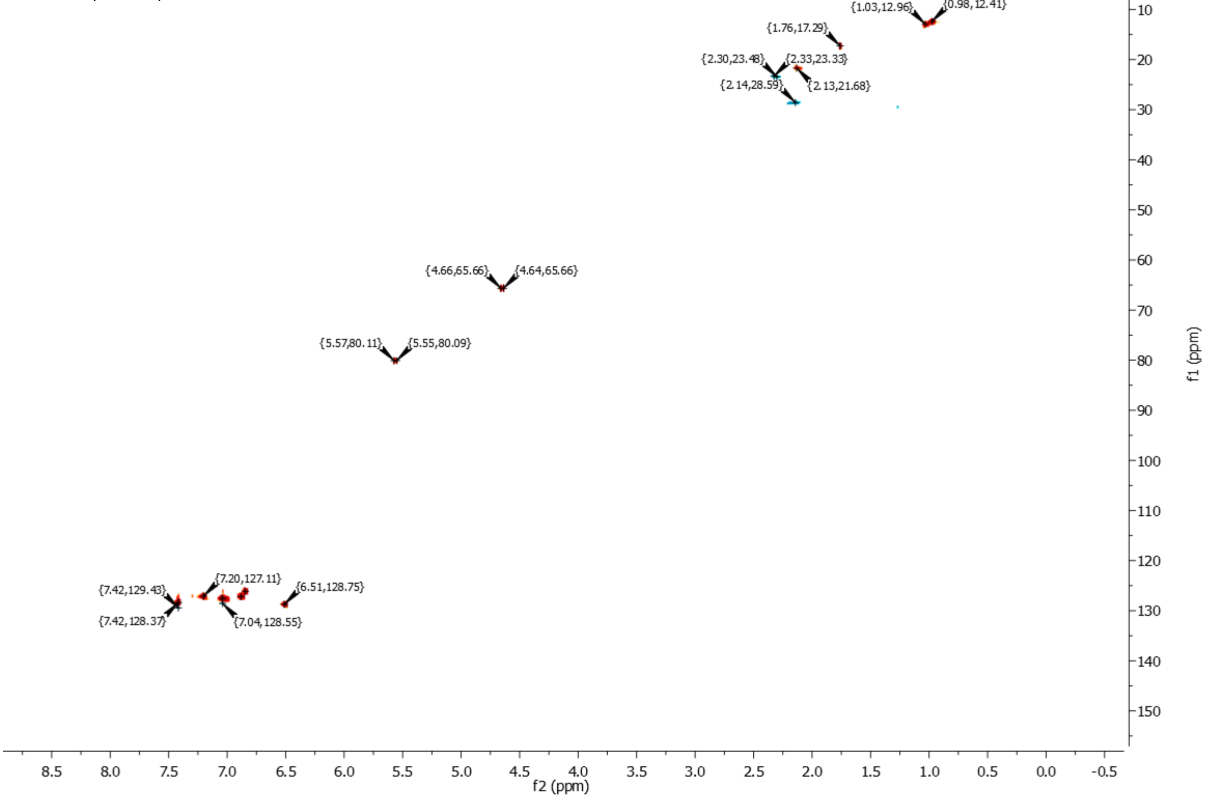

(4*S*,5*R*)-3-((2*Z*,5*Z*)-5-ethyl-6-methyl-4-oxo-2-phenylocta-2,5-dien-3-yl)-4,5-diphenyloxazolidin-2-one (2Z,5Z)-17

NOESY ($CDCl_3$, 400 MHz)

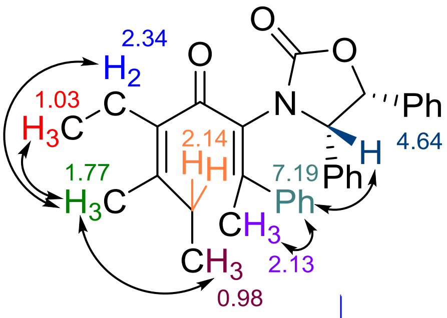

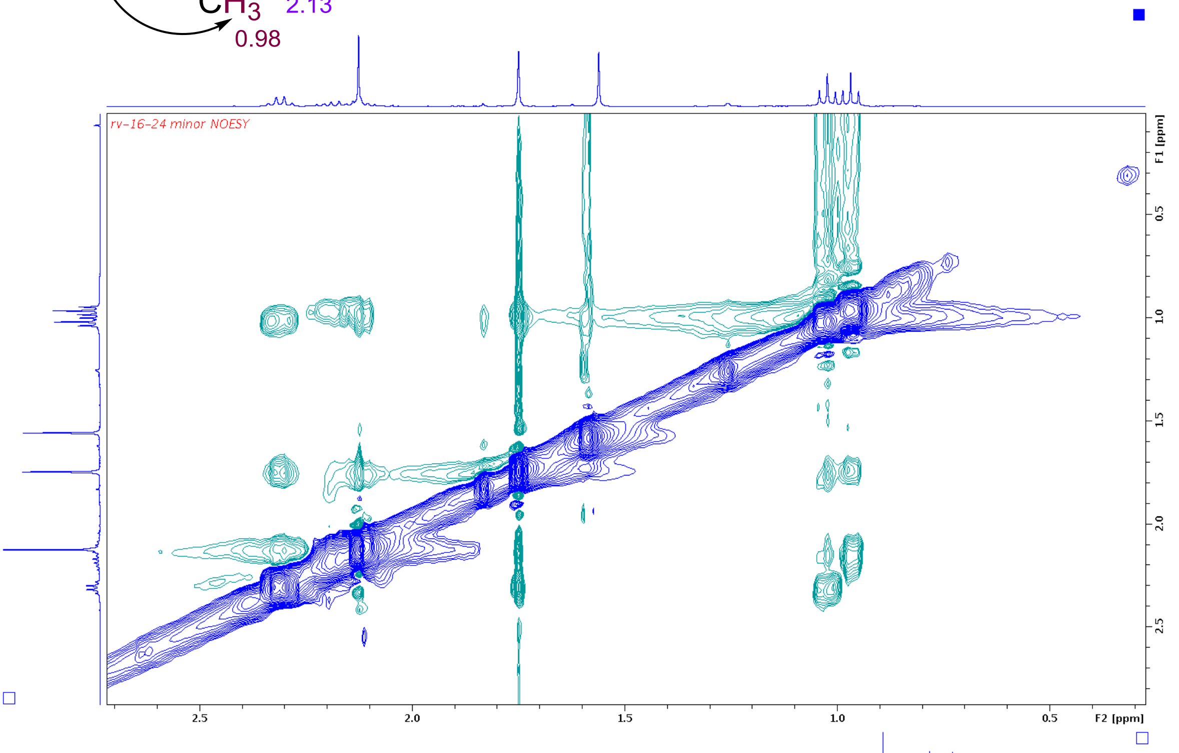

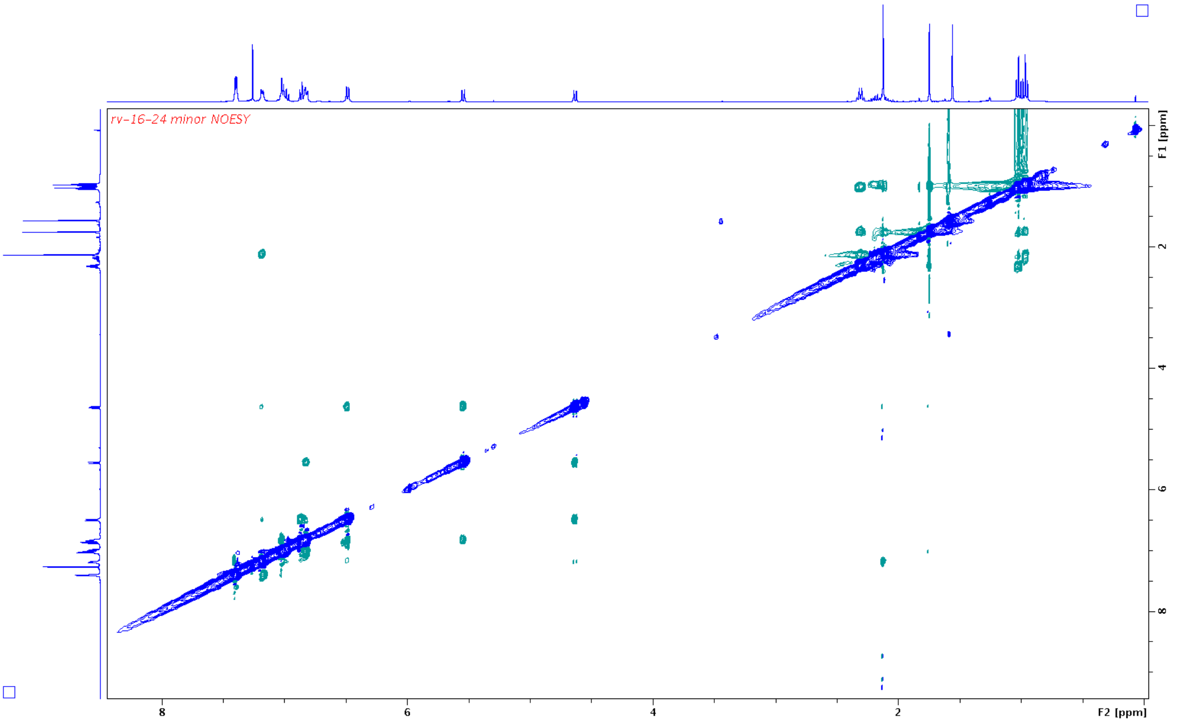

(*E*)-1-((3-bromo-2-methylbut-2-en-1-yl)oxy)-3,5-dimethoxybenzene 19

^{1}H NMR ($CDCl_3$, 400 MHz)

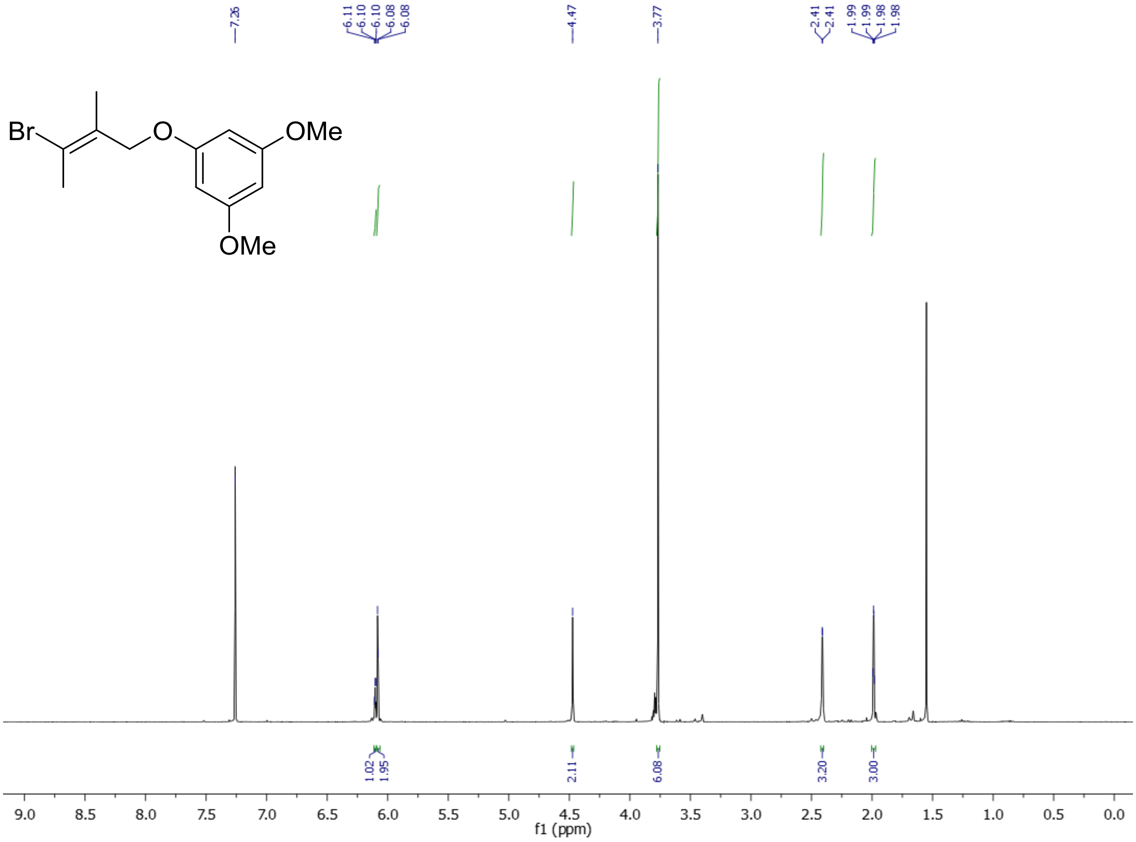

^{13}C NMR ($CDCl_3$, 101 MHz, DEPT-Q)

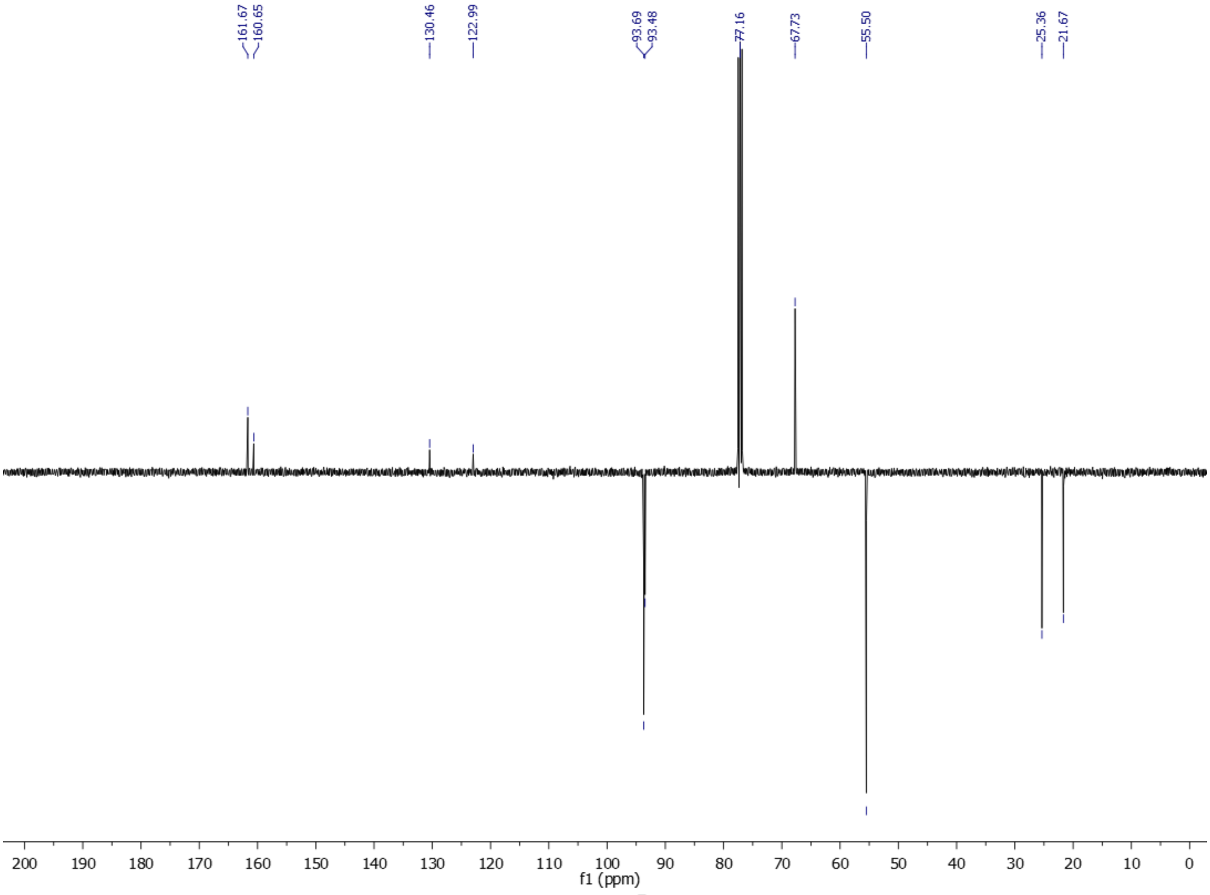

(4*S*,5*R*)-3-((2*Z*,5*E*)-7-(3,5-dimethoxyphenoxy)-5,6-dimethyl-4-oxo-2-phenylhepta-2,5-dien-3-yl)-4,5-diphenyloxazolidin-2-one 20

^{1}H NMR ($CDCl_3$, 400 MHz)

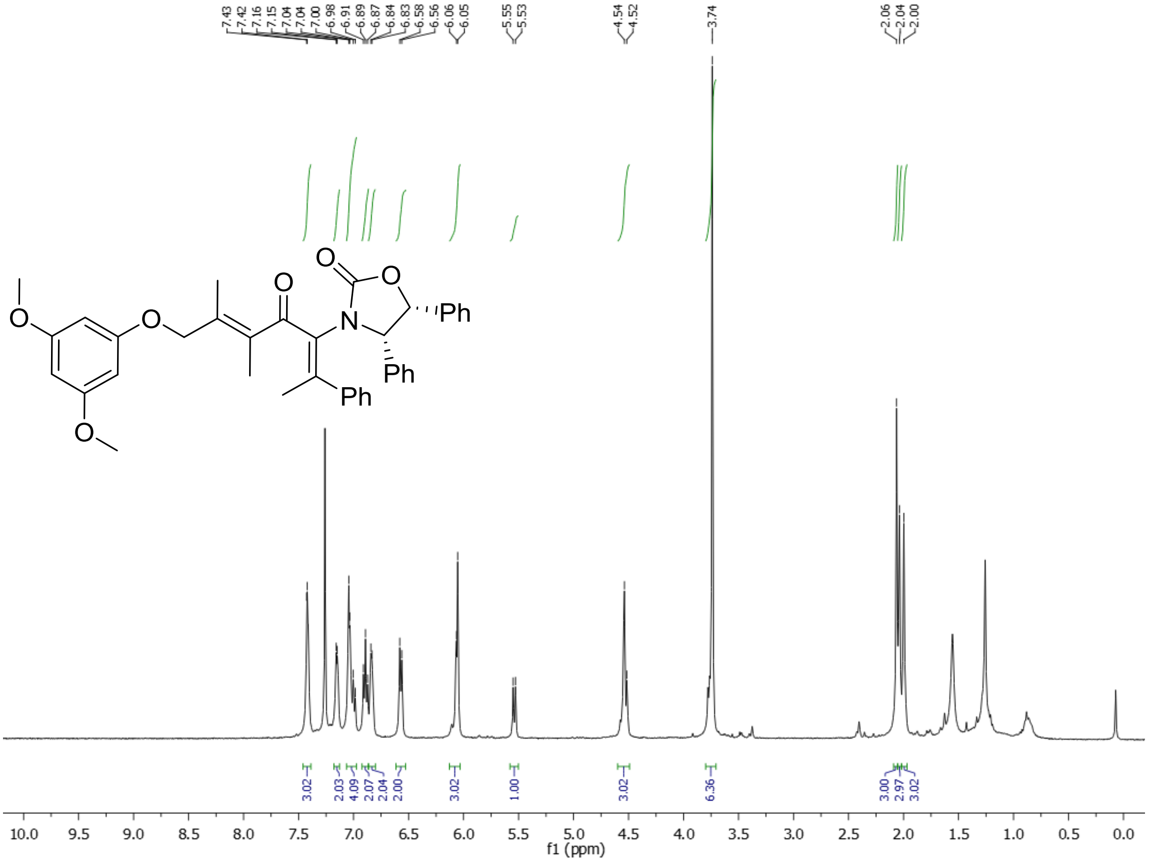

^{13}C NMR ($CDCl_3$, 101 MHz, DEPT-Q)

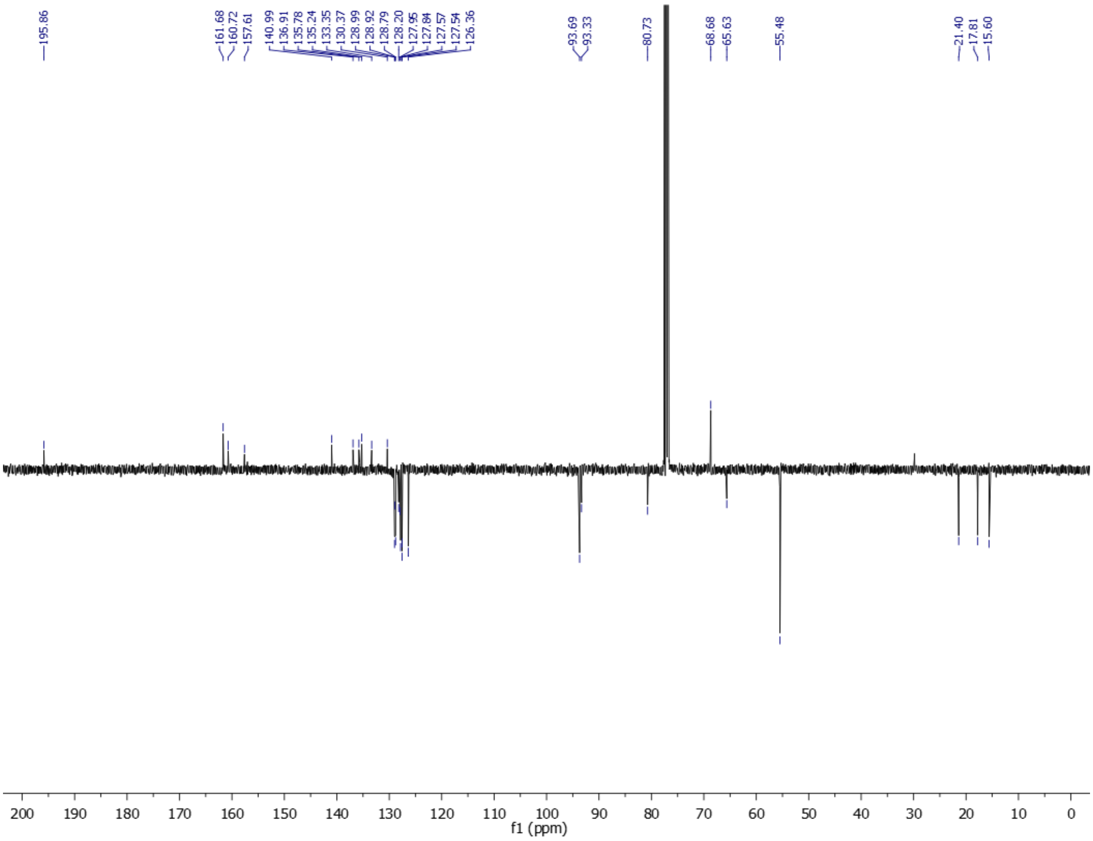

(*Z*)-5-((*E*)-4-(3,5-dimethoxyphenoxy)-3-methylbut-2-en-2-yl)-3-((*S*)-2-oxo-1,2-diphenylethyl)-4-(1-phenylethylidene)oxazolidin-2-one (SI-3)

^{1}H NMR ($CDCl_3$, 400 MHz)

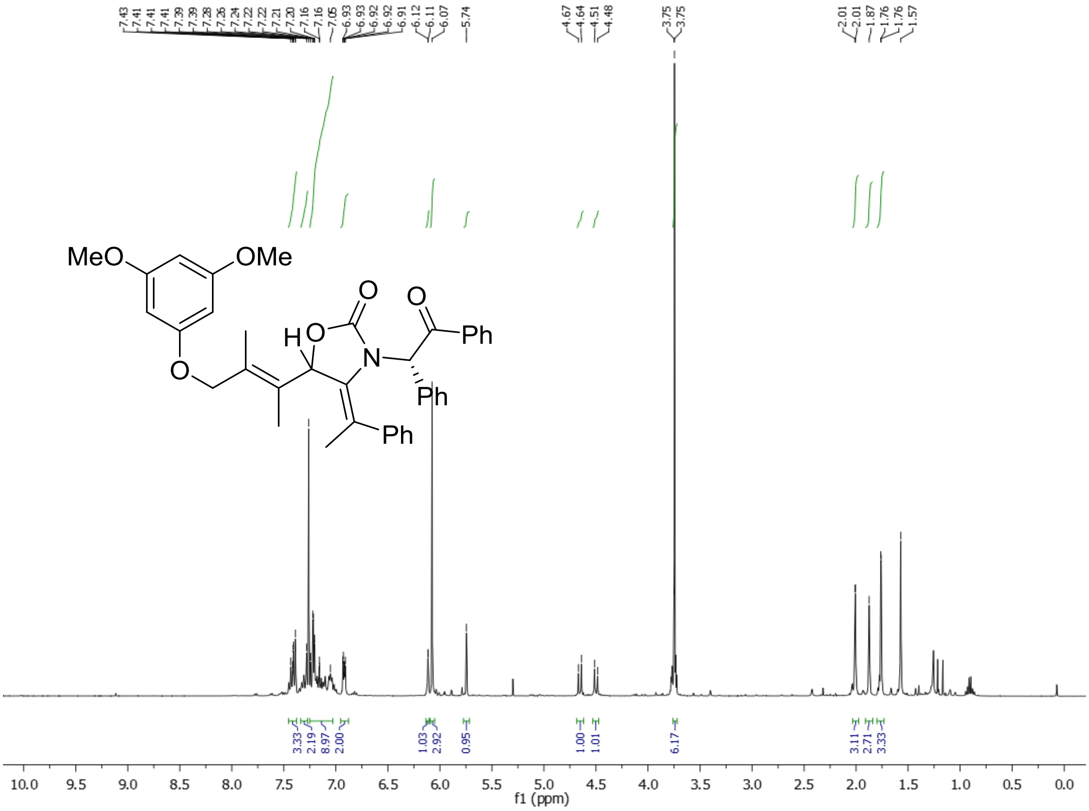

^{13}C NMR ($CDCl_3$, 101 MHz, DEPT-Q)

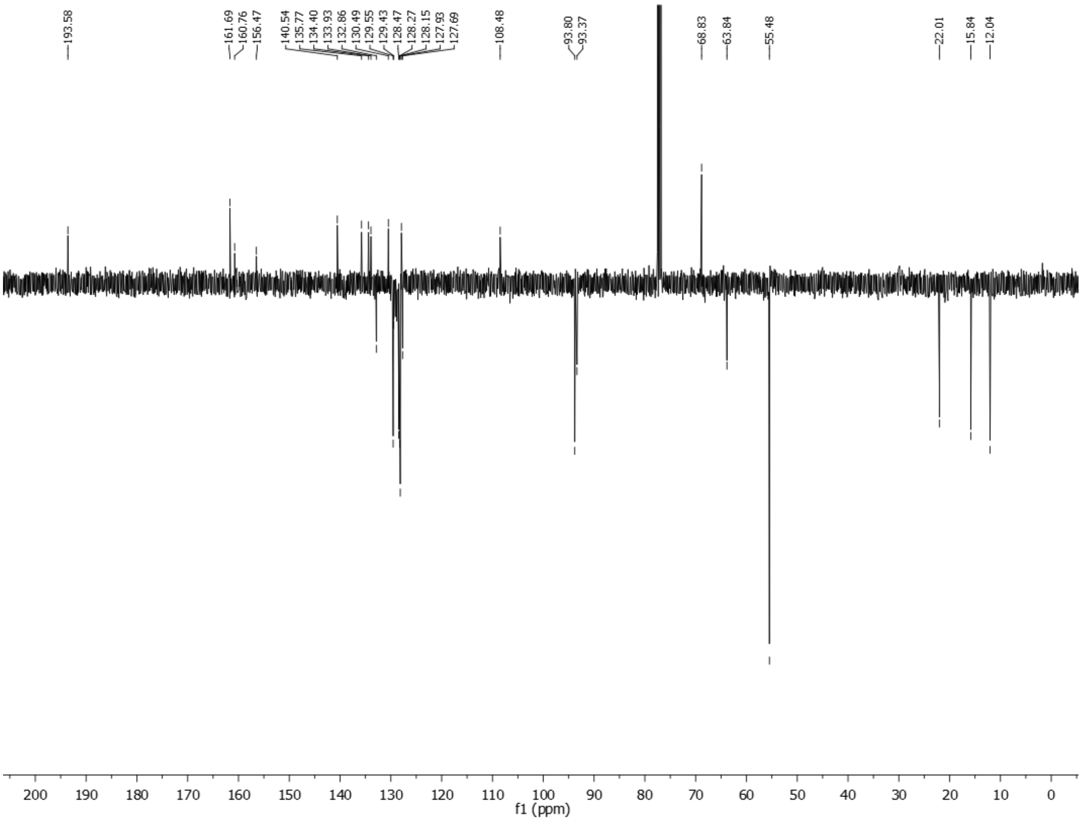

(*Z*)-5-((*E*)-4-(3,5-dimethoxyphenoxy)-3-methylbut-2-en-2-yl)-3-((*S*)-2-oxo-1,2-diphenylethyl)-4-(1-phenylethylidene)oxazolidin-2-one (SI-3)

HSQC ($CDCl_3$)

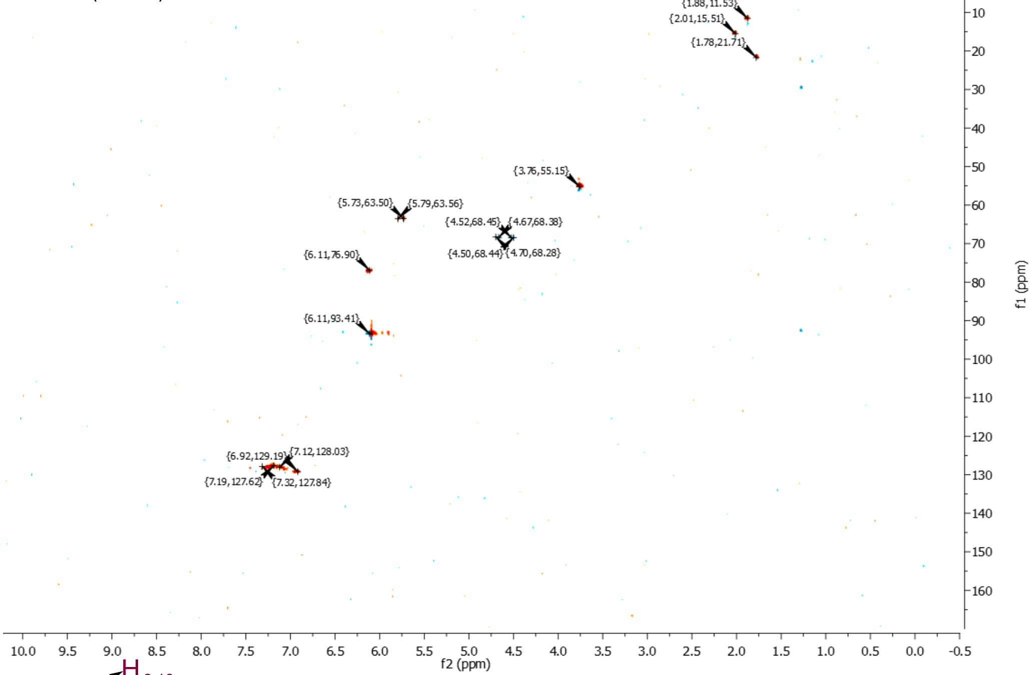

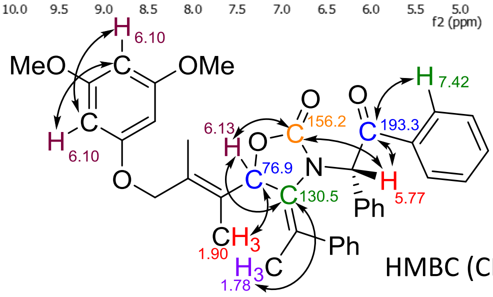

HMBC ($CDCl_3$)

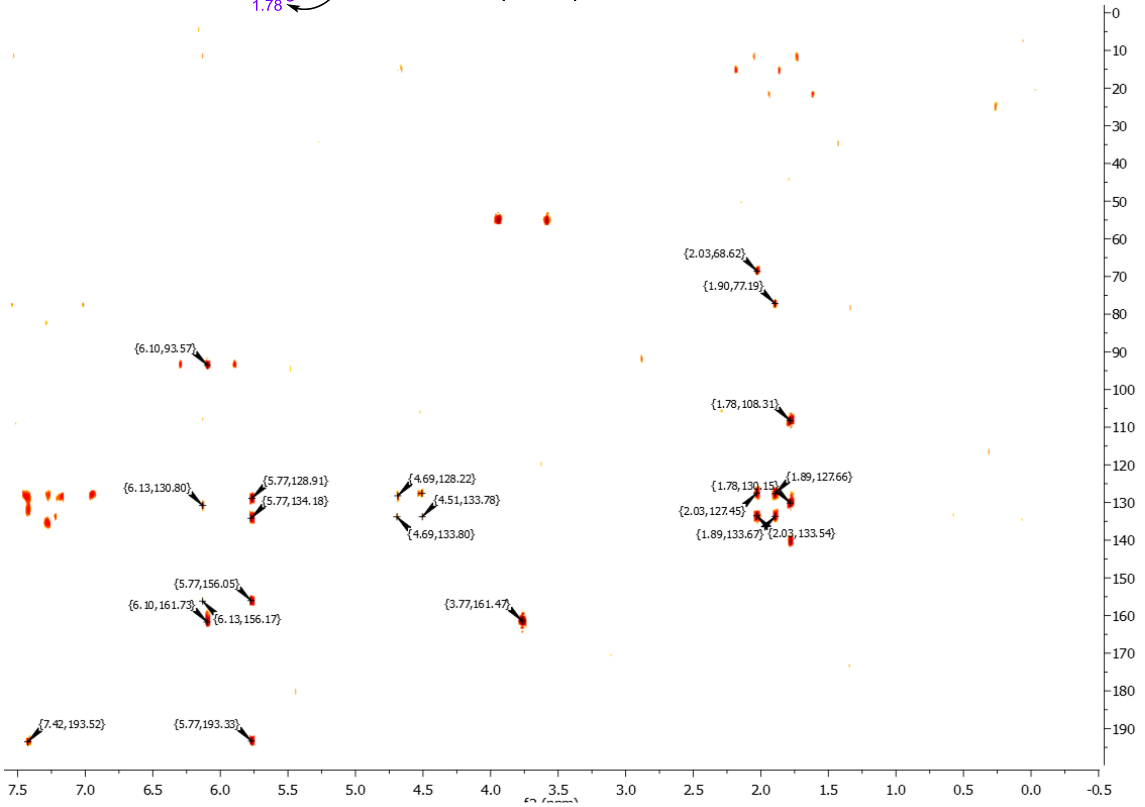

(*Z*)-5-((*E*)-4-(3,5-dimethoxyphenoxy)-3-methylbut-2-en-2-yl)-3-((*S*)-2-oxo-1,2-diphenylethyl)-4-(1-phenylethylidene)oxazolidin-2-one (SI-3)

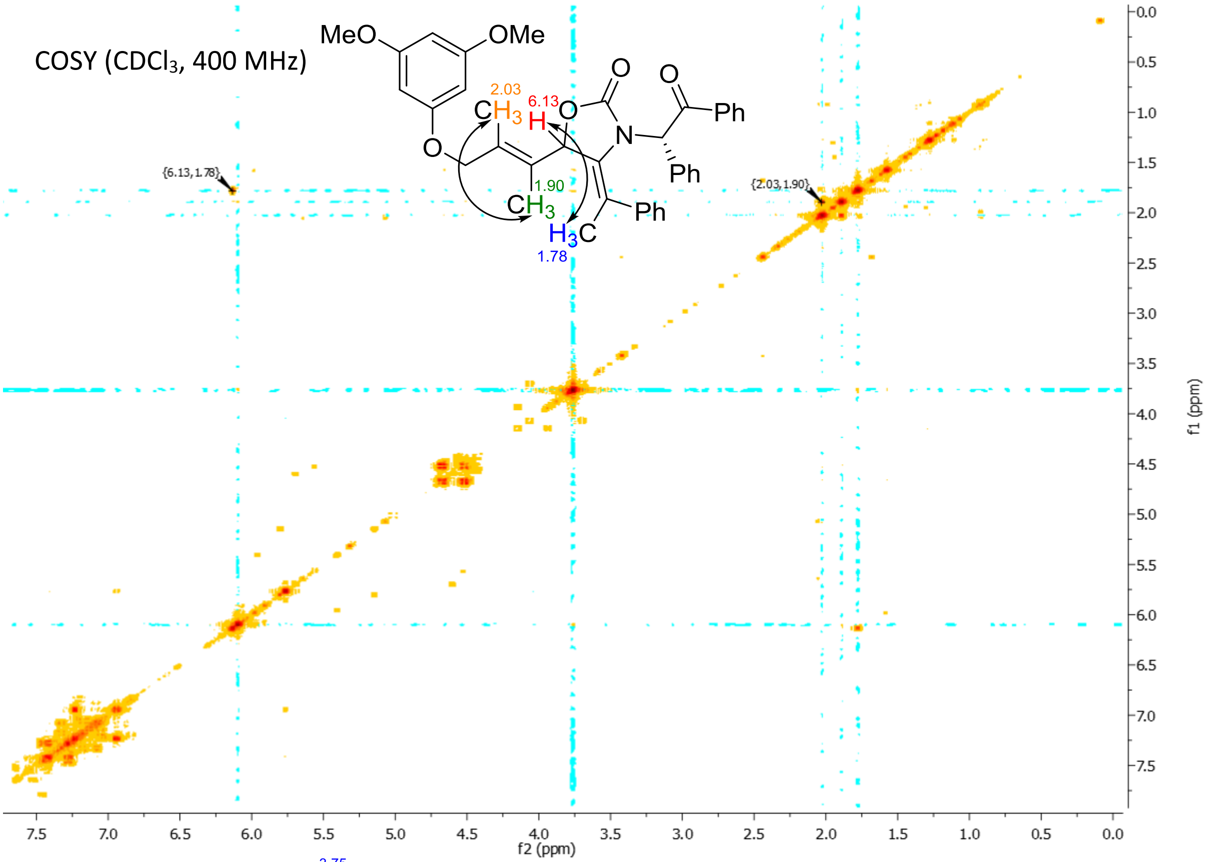

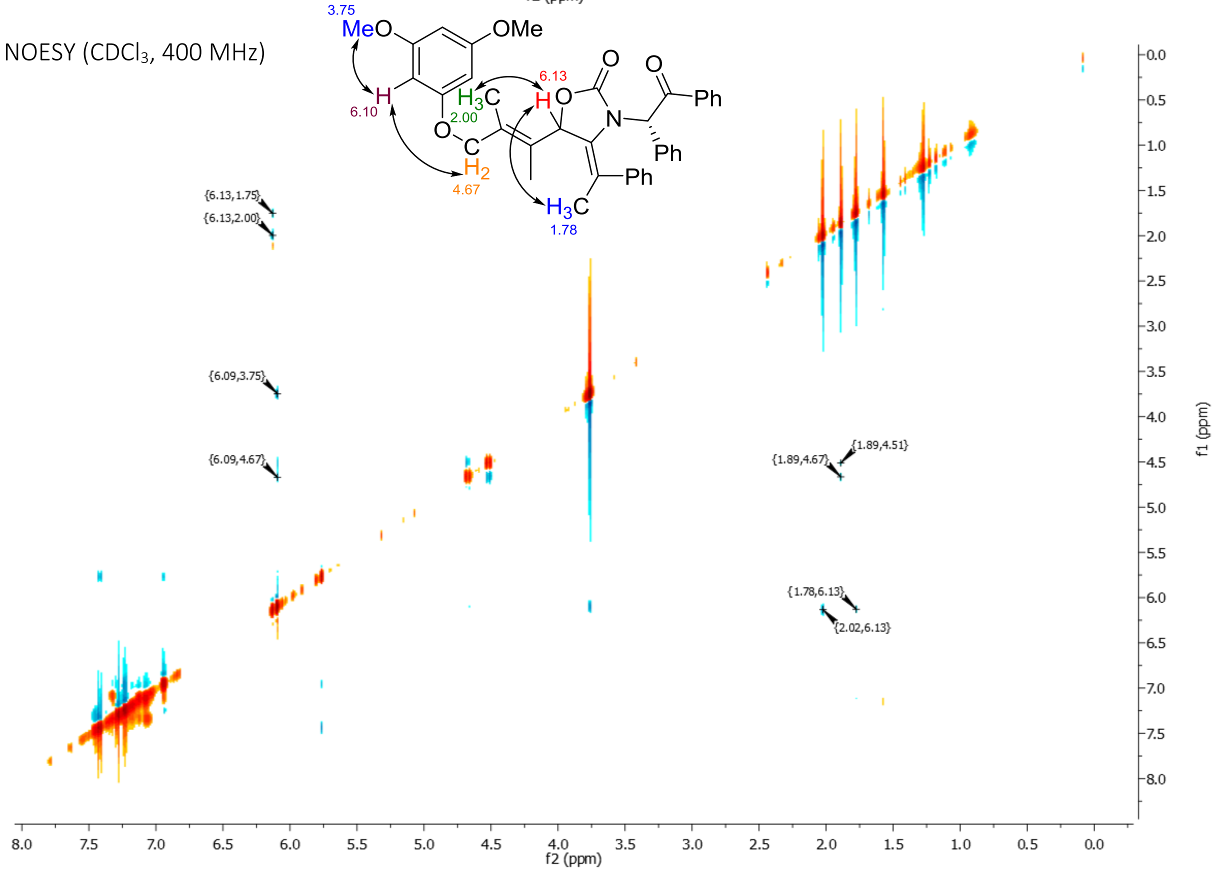

(4*S*,5*R*)-3-((*R*)-4,4-diethyl-2,3-dimethyl-5-oxo-3-phenylcyclopent-1-en-1-yl)-4,5-diphenyloxazolidin-2-one (3*R*)-23

^{1}H NMR ($CDCl_3$, 400 MHz)

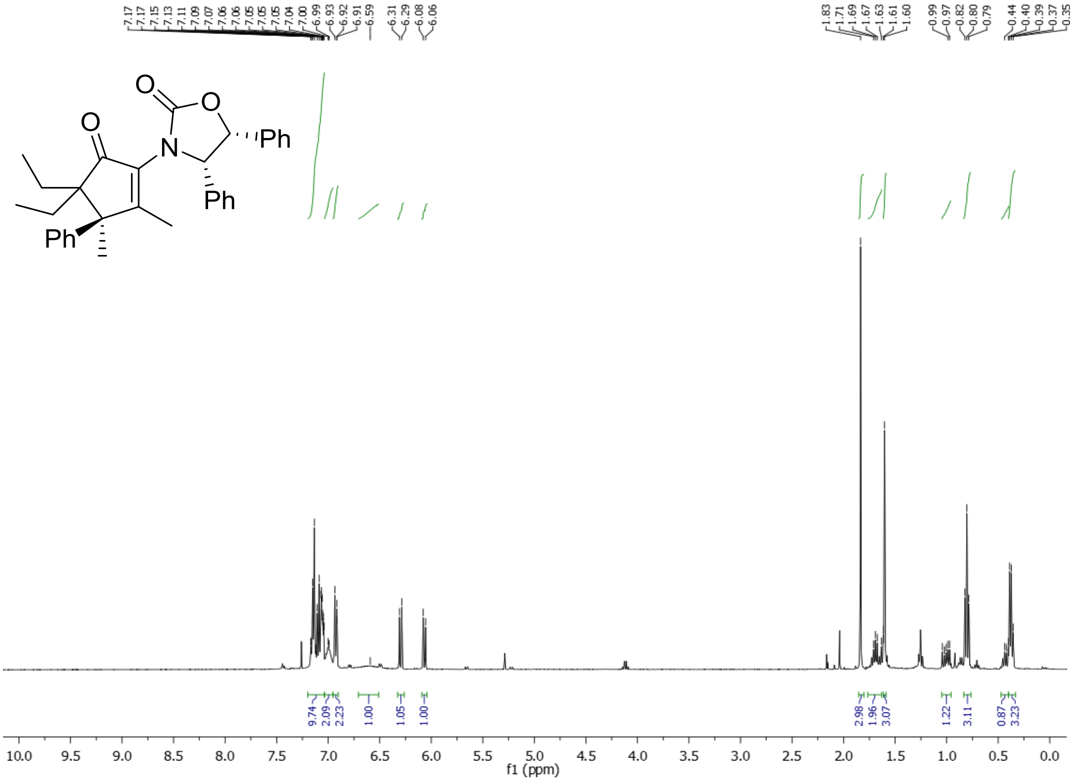

^{13}C NMR ($CDCl_3$, 101 MHz, DEPT-Q)

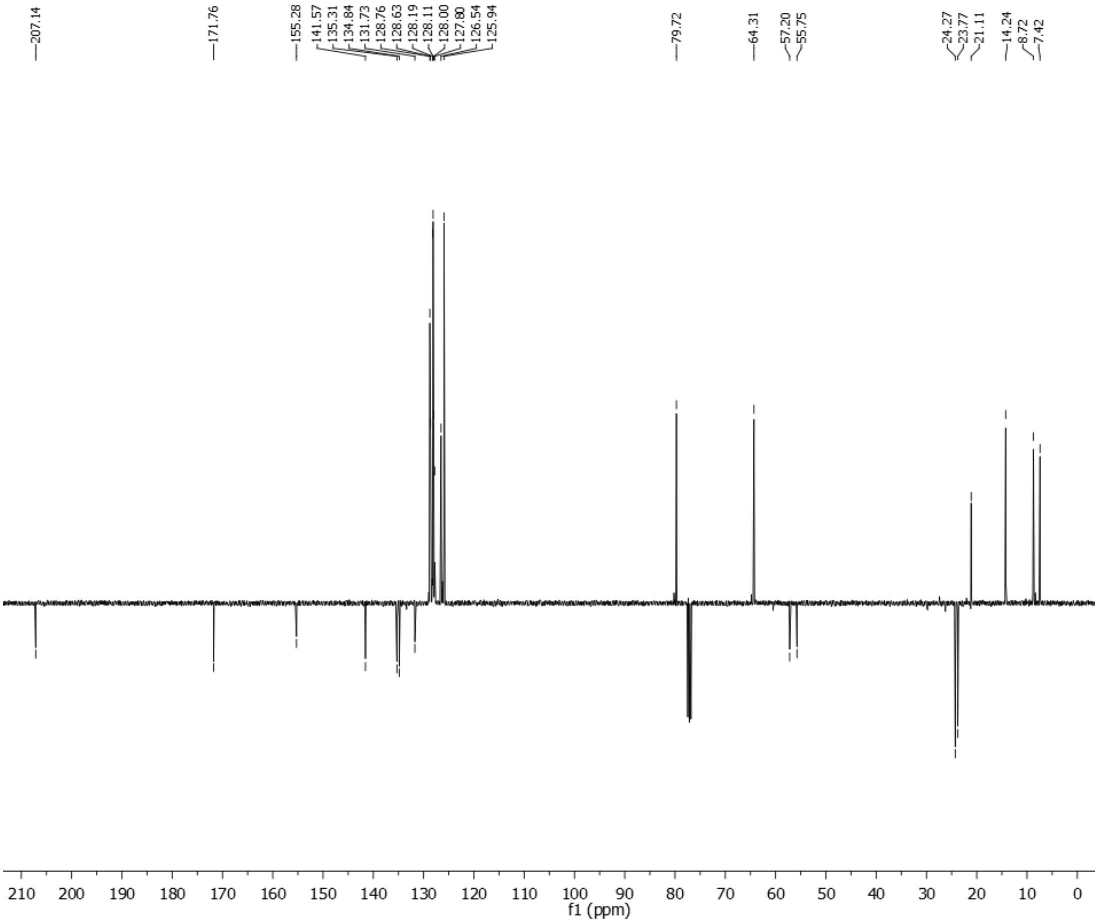

(4*S*,5*R*)-3-((*R*)-4,4-diethyl-2,3-dimethyl-5-oxo-3-phenylcyclopent-1-en-1-yl)-4,5-diphenyloxazolidin-2-one (3*R*)-23

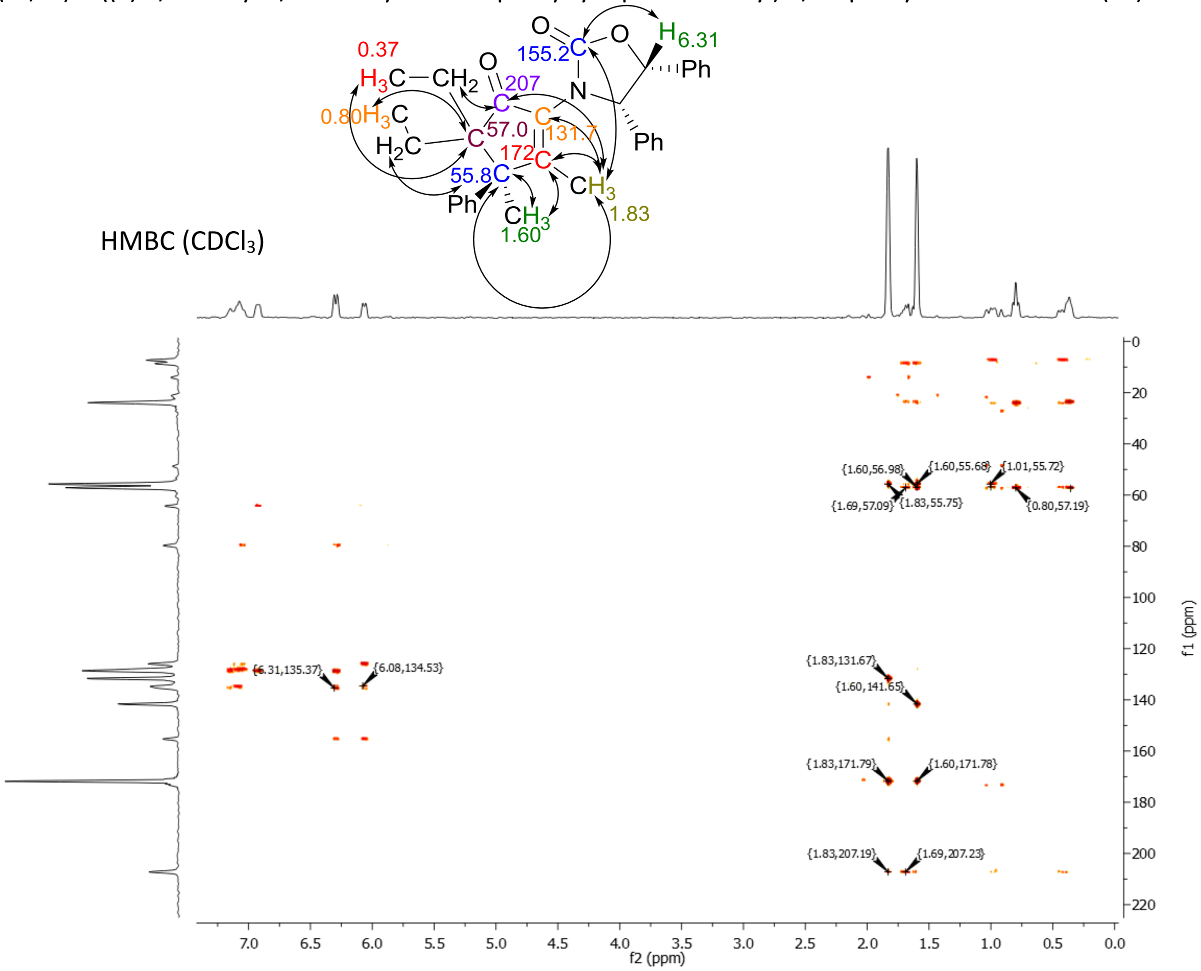

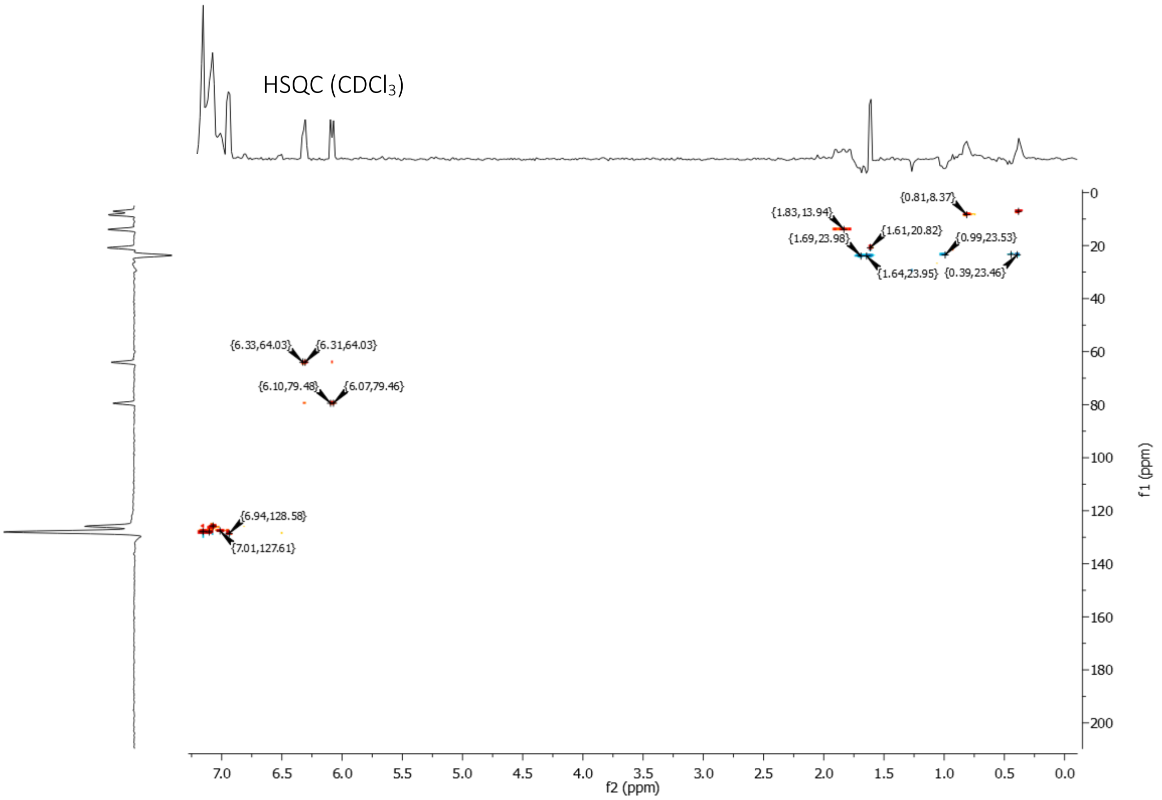

(4*S*,5*R*)-3-((*S*)-4,4-diethyl-2,3-dimethyl-5-oxo-3-phenylcyclopent-1-en-1-yl)-4,5-diphenyloxazolidin-2-one (3*S*)-23

^{1}H NMR (CDCl$_3$, 400 MHz)

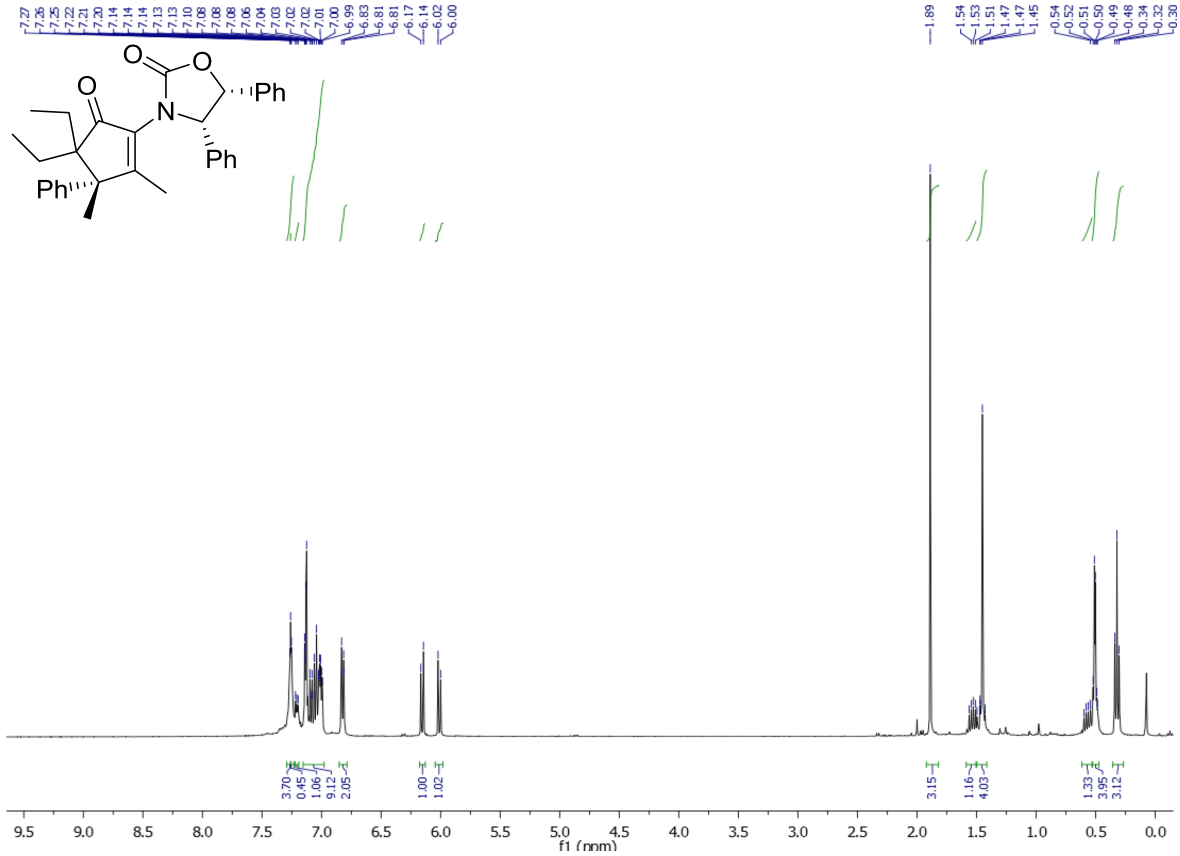

^{13}C NMR (CDCl$_3$, 101 MHz, DEPT-Q)

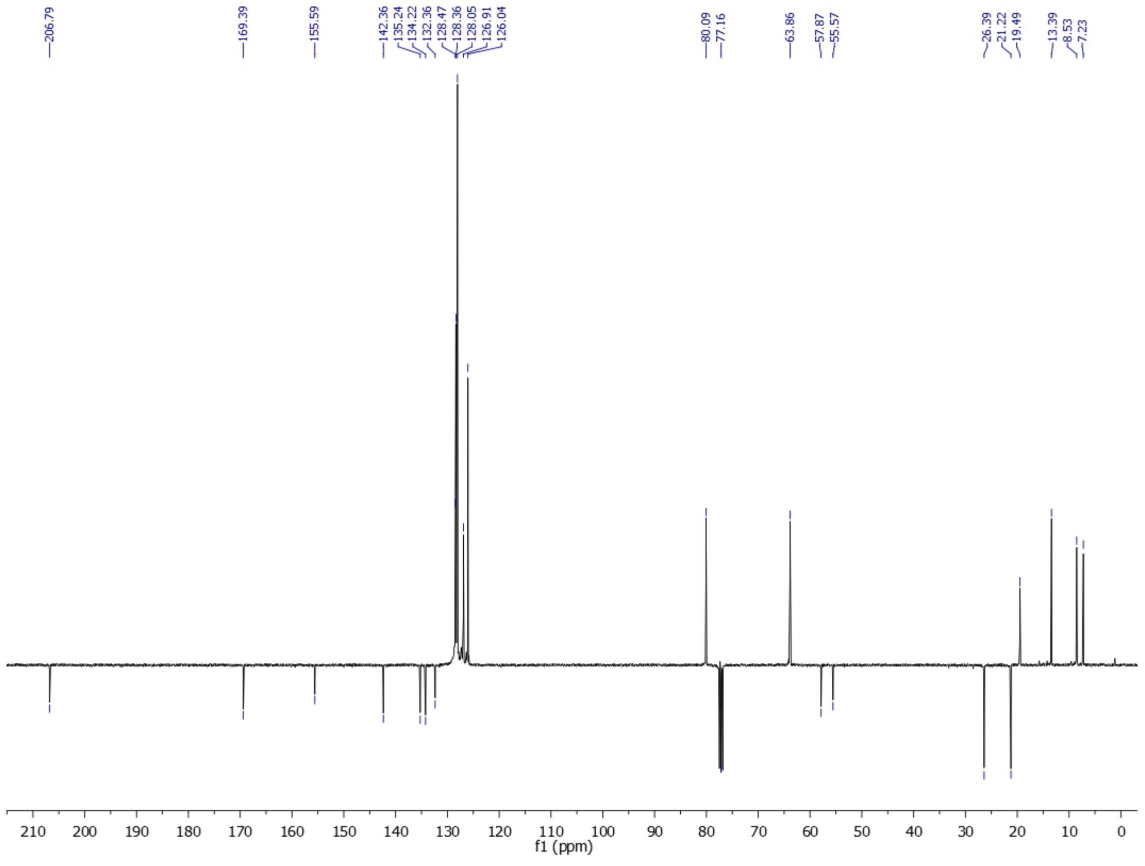

(4*S*,5*R*)-3-((1*S*,2*S*,3*S*,*Z*)-3-ethyl-4-ethylidene-2,3-dimethyl-5-oxo-2-phenylcyclopentyl)-4,5-diphenyloxazolidin-2-one (*Z*)-24

^{1}H NMR (CDCl$_3$, 400 MHz)

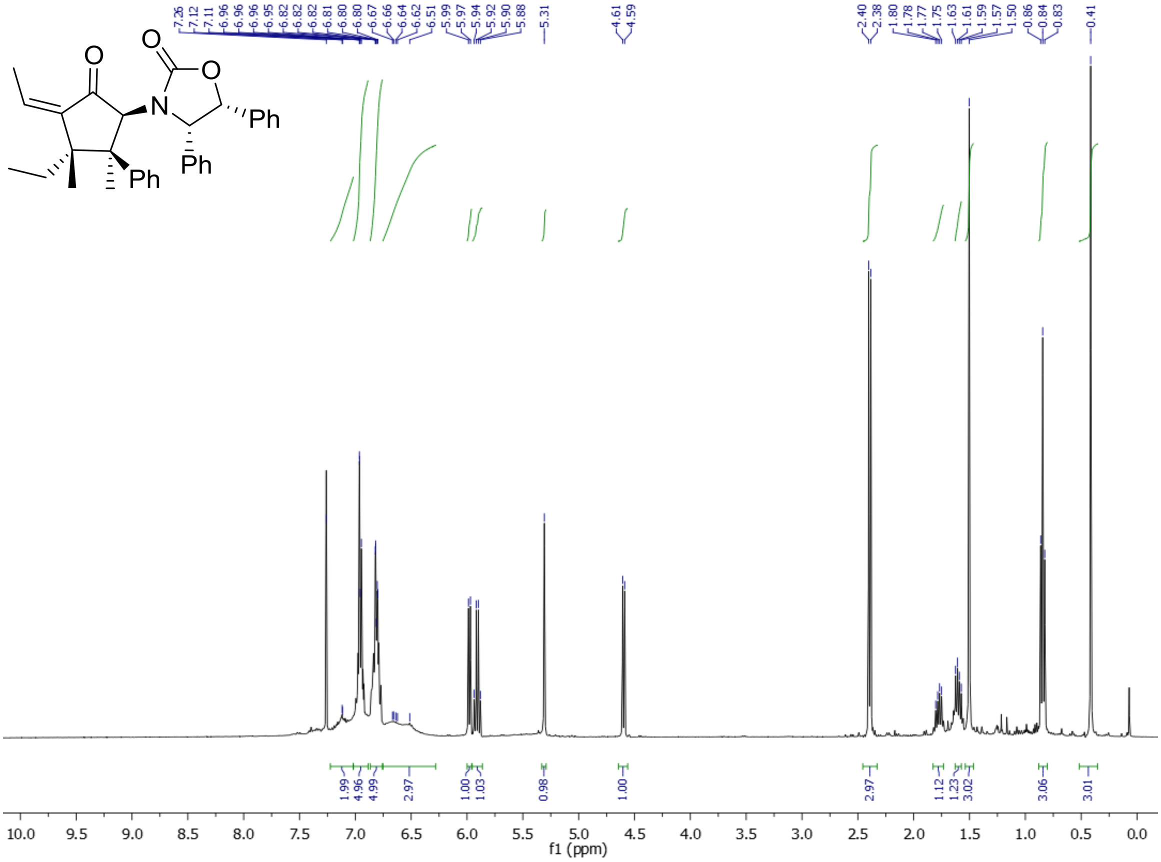

^{13}C NMR (CDCl$_3$, 101 MHz, DEPT-Q)

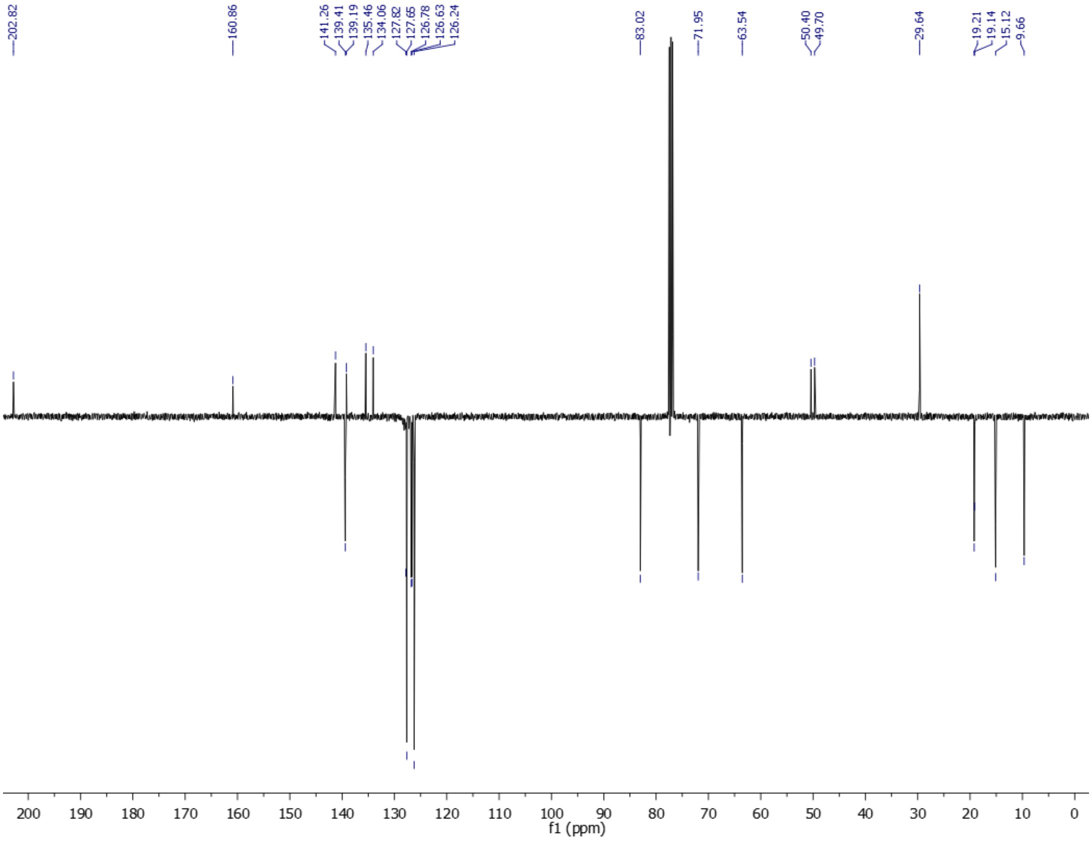

(4*S*,5*R*)-3-((1*S*,2*S*,3*S*,*Z*)-3-ethyl-4-ethylidene-2,3-dimethyl-5-oxo-2-phenylcyclopentyl)-4,5-diphenyloxazolidin-2-one (*Z*)-24

HMBC ($CDCl_3$)

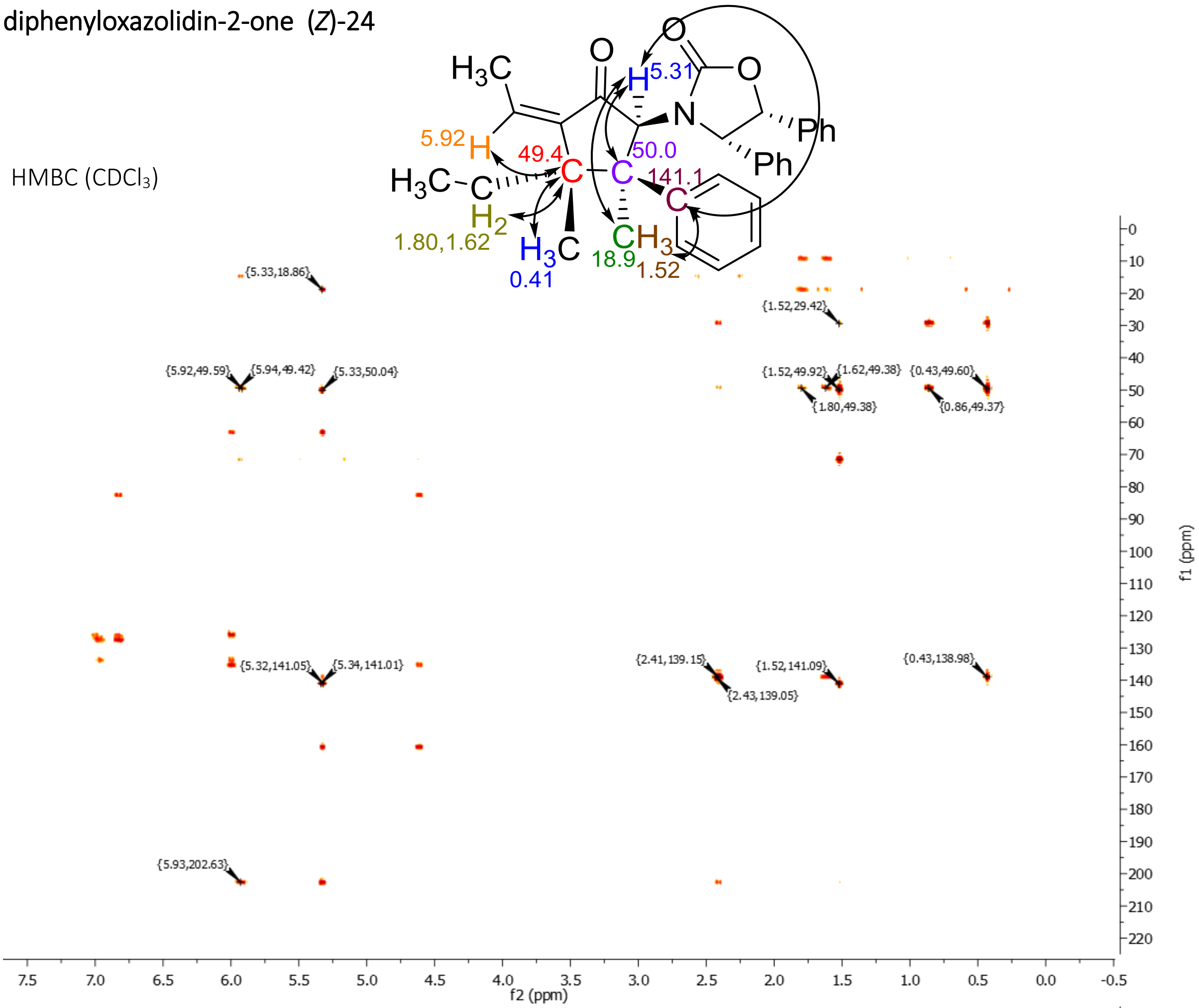

HSQC ($CDCl_3$)

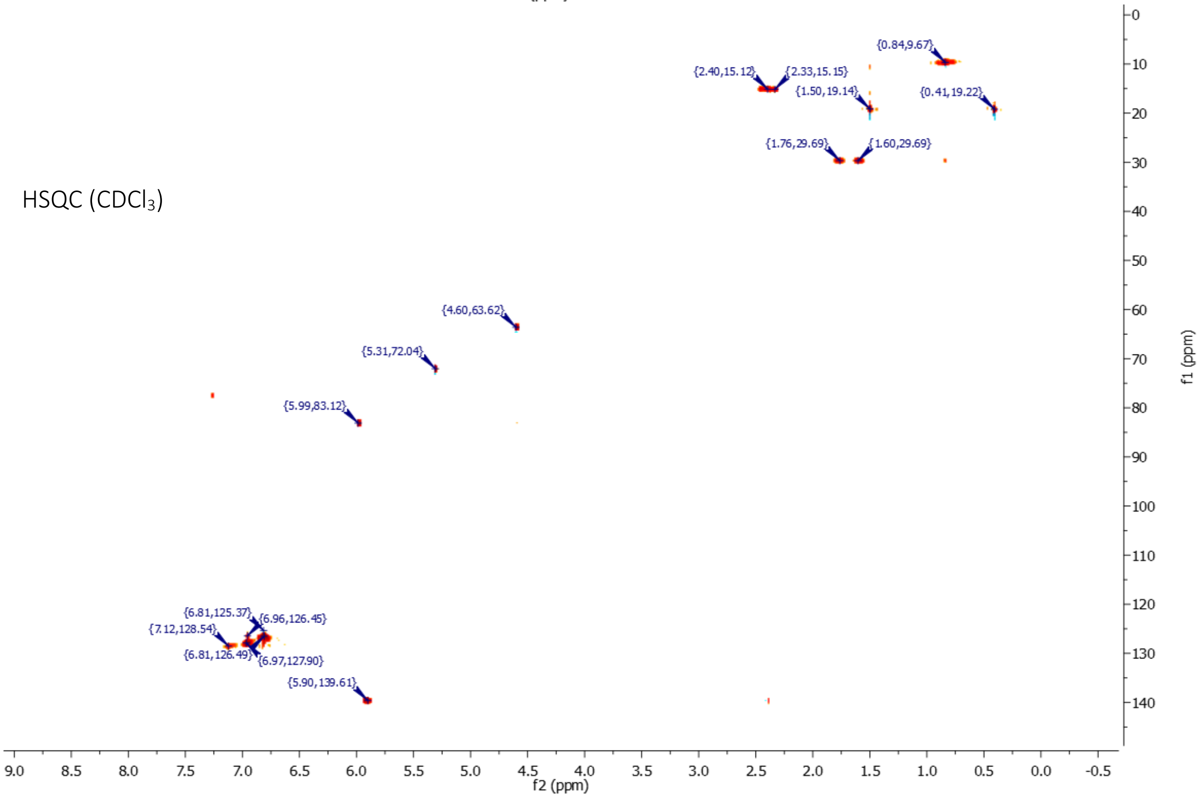

(4*S*,5*R*)-3-((1*S*,2*S*,3*S*,*Z*)-3-ethyl-4-ethylidene-2,3-dimethyl-5-oxo-2-phenylcyclopentyl)-4,5-diphenyloxazolidin-2-one (*Z*)-24

NOESY ($CDCl_3$, 400 MHz)

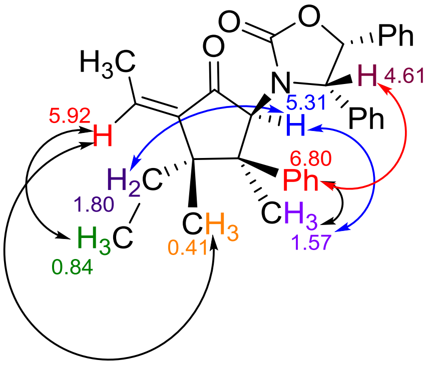

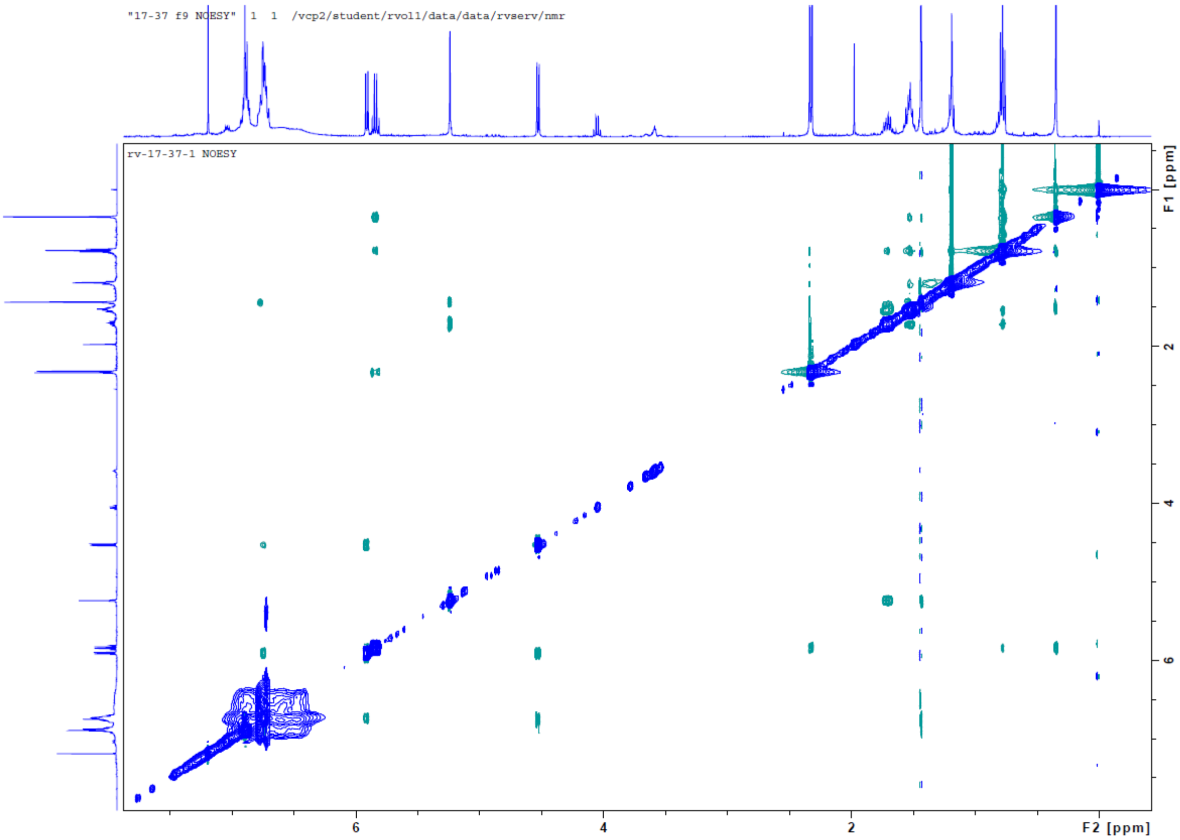

(4*S*,5*R*)-3-((1*S*,2*S*,3*S*,*E*)-3-ethyl-4-ethylidene-2,3-dimethyl-5-oxo-2-phenylcyclopentyl)-4,5-diphenyloxazolidin-2-one (*E*)-24

^{1}H NMR ($CDCl_3$, 400 MHz)

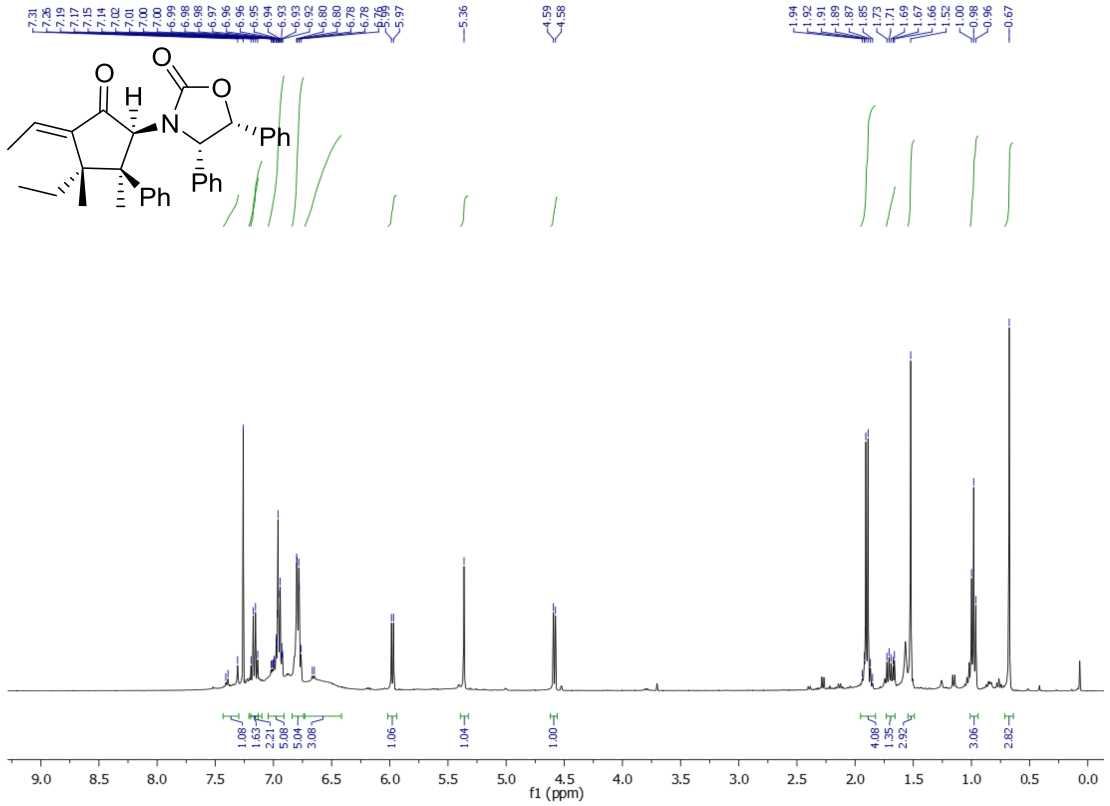

^{13}C NMR ($CDCl_3$, 101 MHz, DEPT-Q)

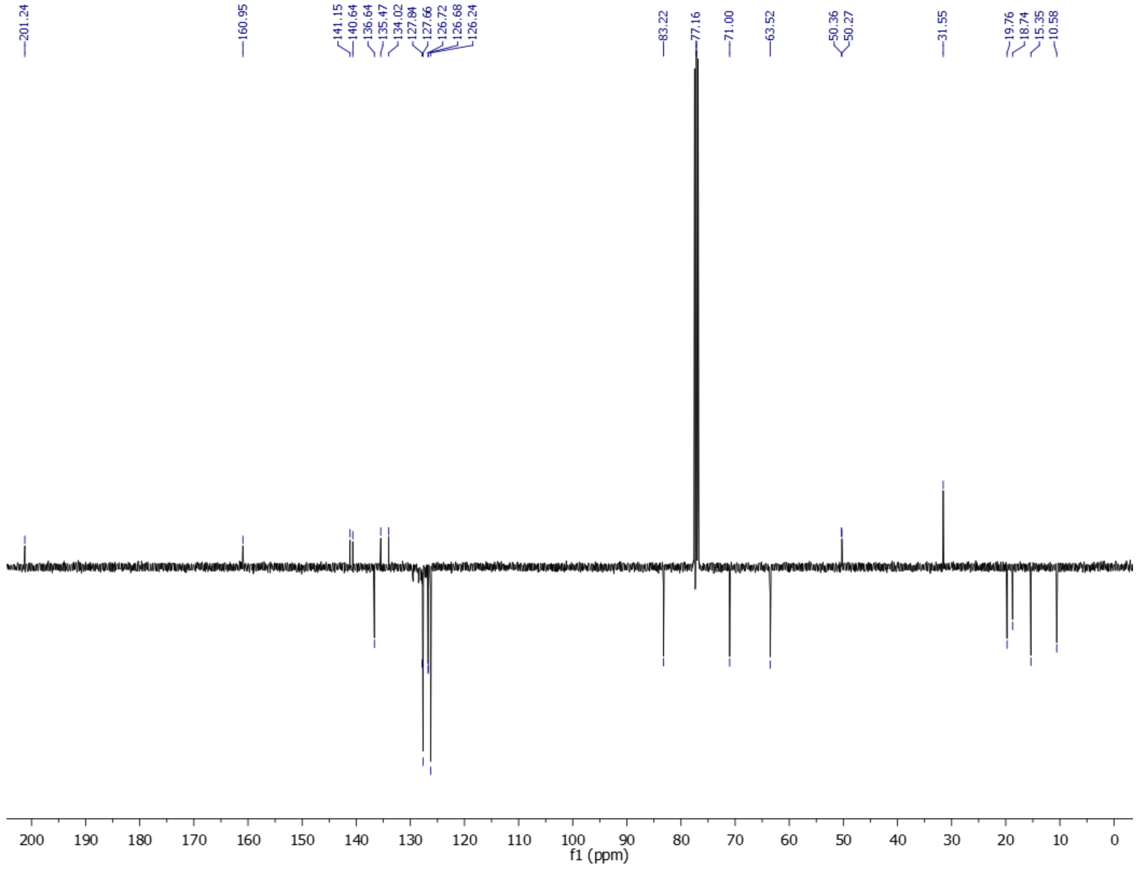

(4*S*,5*R*)-3-((1*S*,2*S*,3*S*,*E*)-3-ethyl-4-ethylidene-2,3-dimethyl-5-oxo-2-phenylcyclopentyl)-4,5-diphenyloxazolidin-2-one (*E*)-24

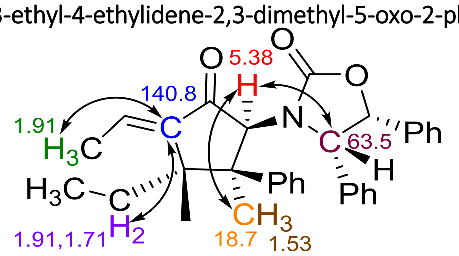

HMBC ($CDCl_3$)

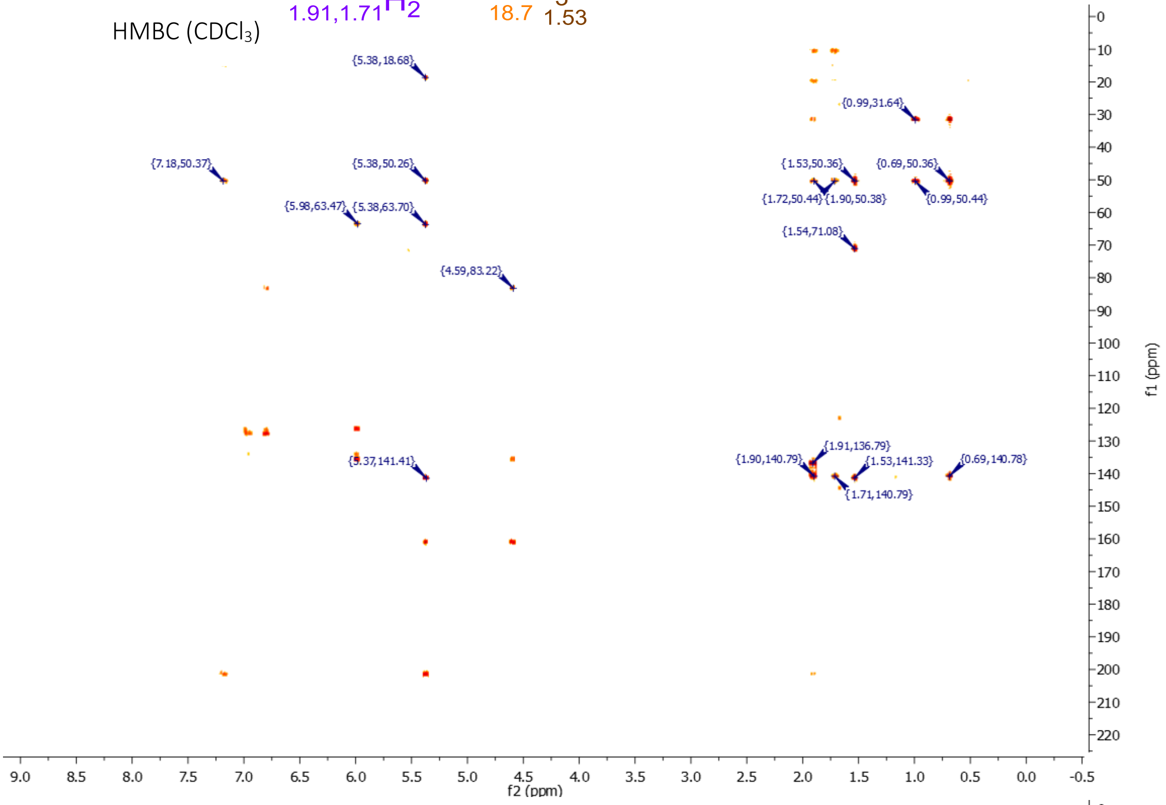

HSQC ($CDCl_3$)

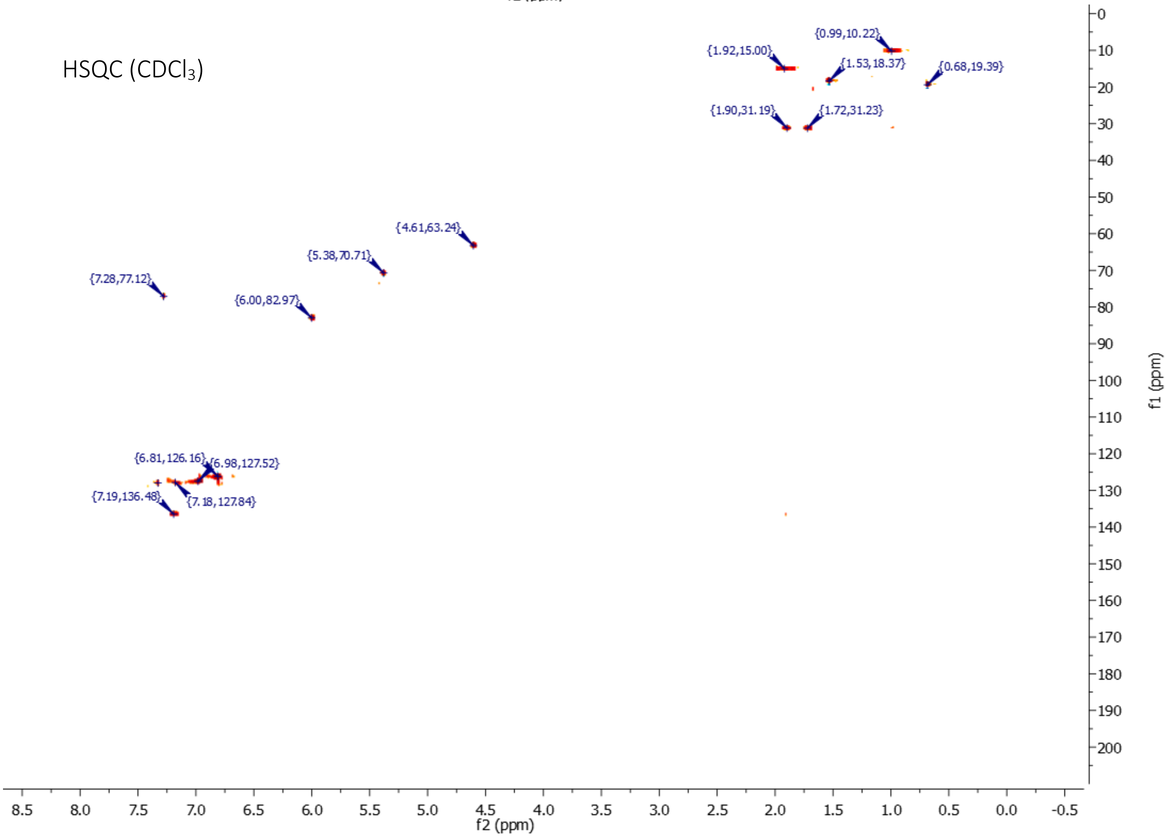

(4*S*,5*R*)-3-((1*S*,2*S*,3*S*,*E*)-3-ethyl-4-ethylidene-2,3-dimethyl-5-oxo-2-phenylcyclopentyl)-4,5-diphenyloxazolidin-2-one (*E*)-24

NOESY ($CDCl_3$, 400 MHz)

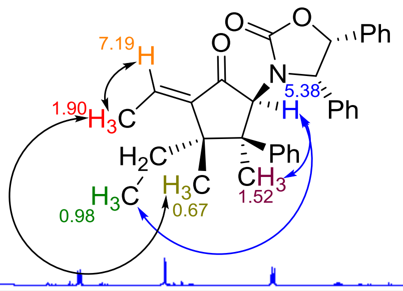

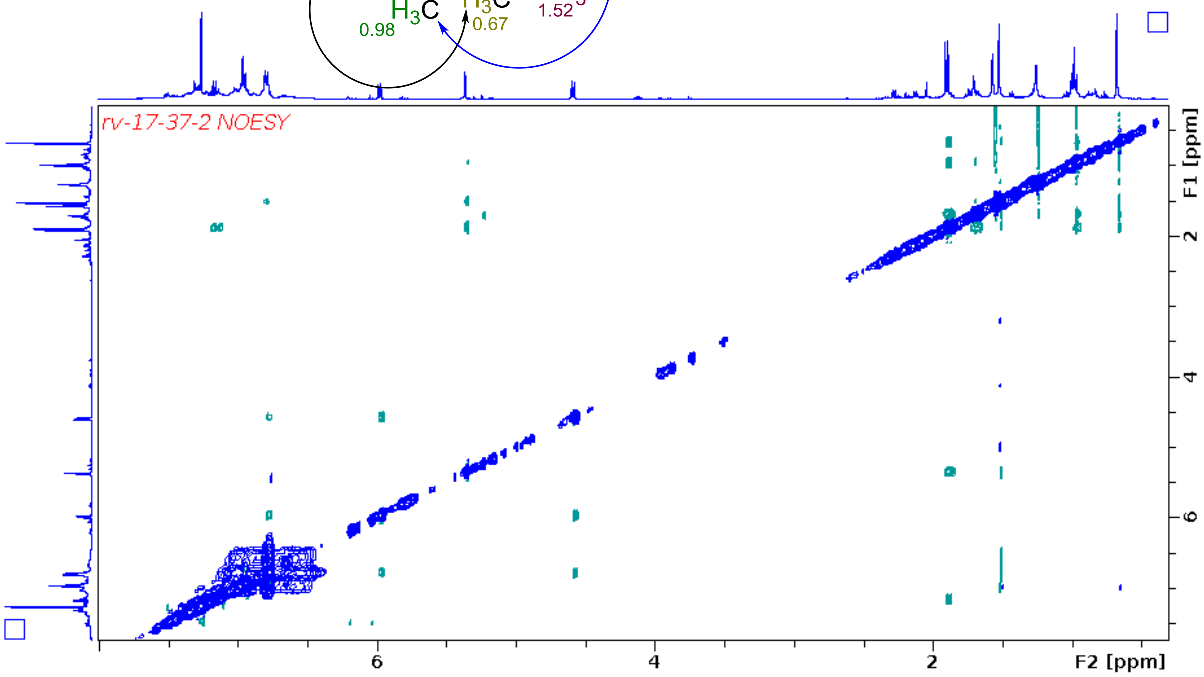

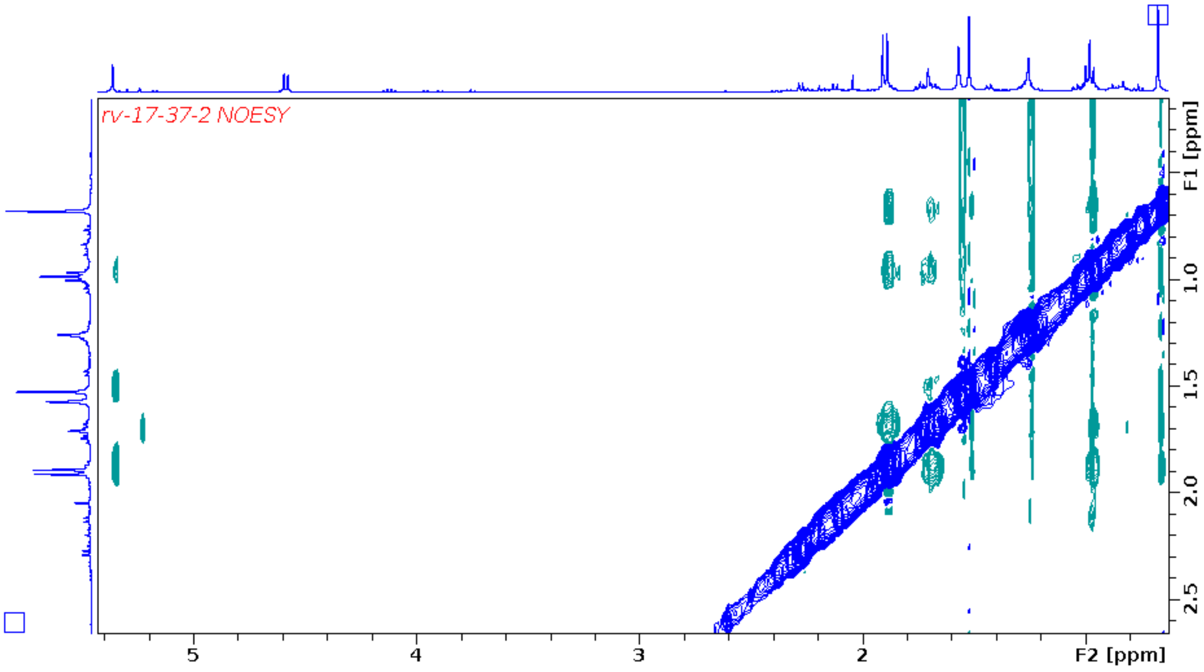

(4*S*,5*R*)-3-((2*Z*,5*E*)-7-(3,5-dimethoxyphenoxy)-5,6-dimethyl-4-oxo-2-phenylhepta-2,5-dien-3-yl)-4,5-diphenyloxazolidin-2-one 25

^{1}H NMR ($CDCl_3$, 400 MHz)

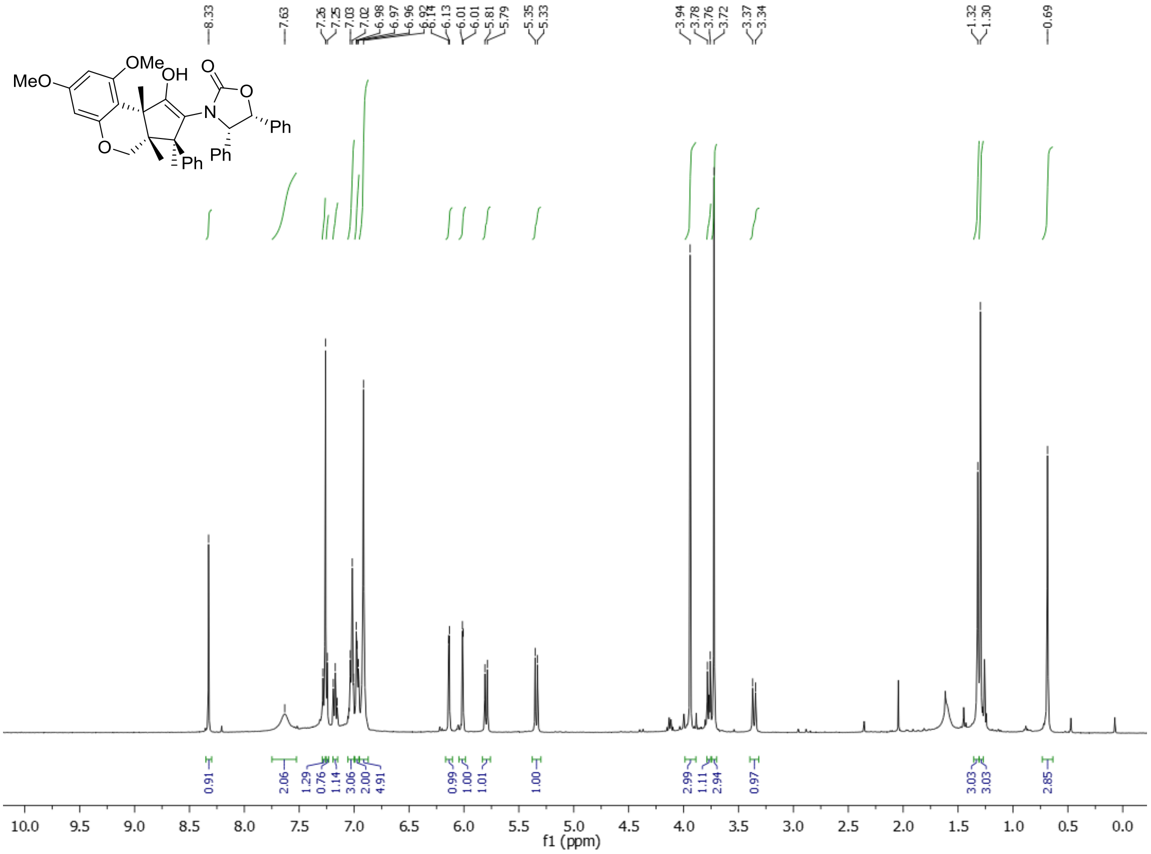

^{13}C NMR ($CDCl_3$, 101 MHz, DEPT-Q)

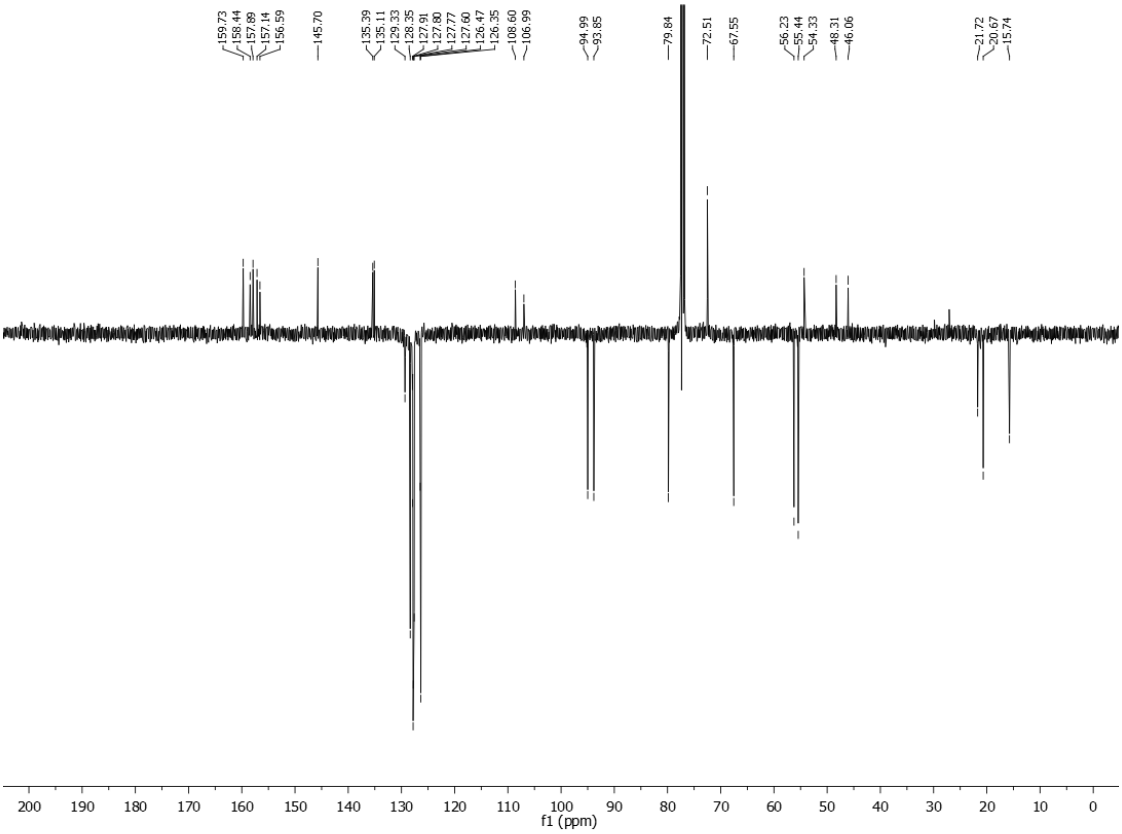

(4S,5*R*)-3-((2*Z*,5*E*)-7-(3,5-dimethoxyphenoxy)-5,6-dimethyl-4-oxo-2-phenylhepta-2,5-dien-3-yl)-4,5-diphenyloxazolidin-2-one 25

HMBC ($CDCl_3$)

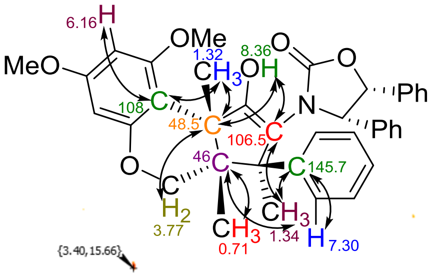

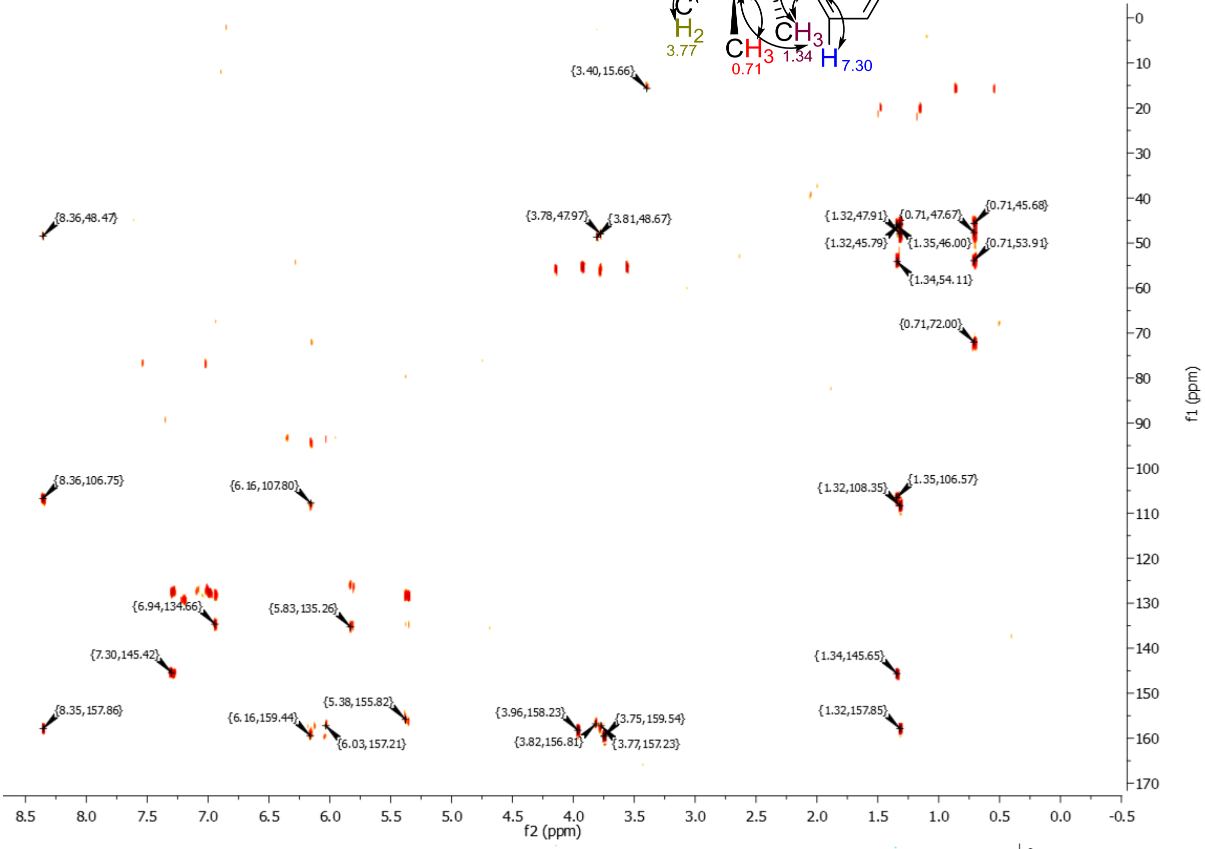

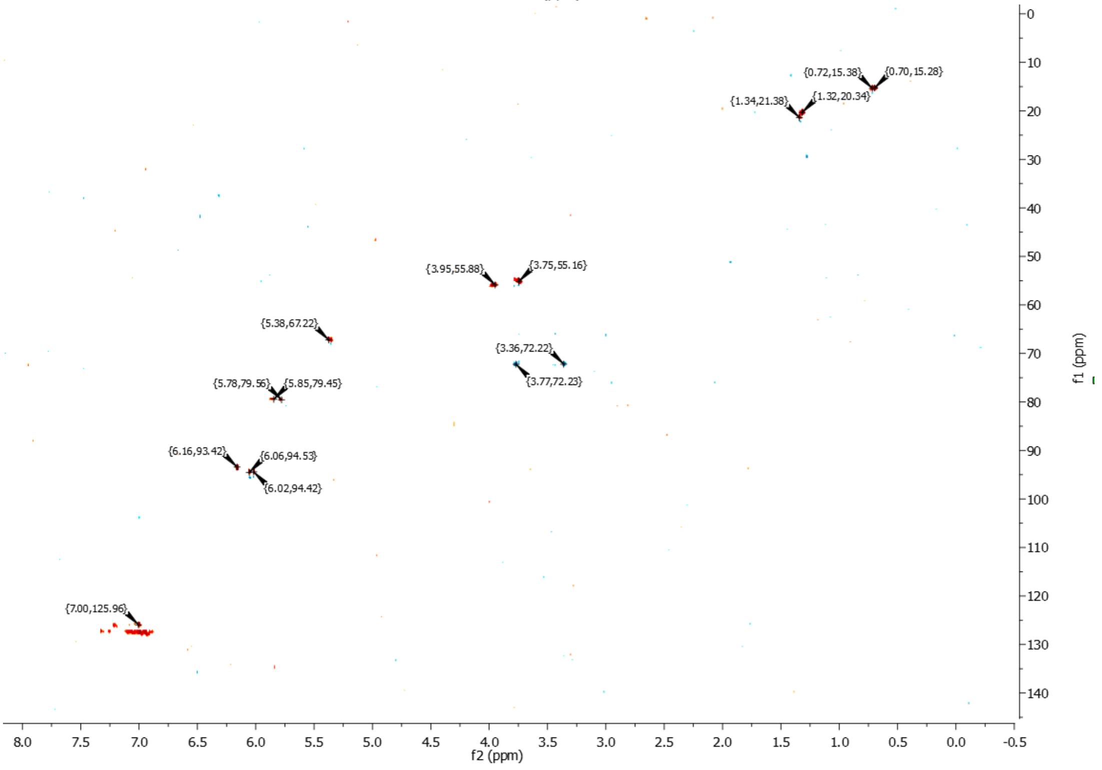

(4S,5R)-3-((2Z,5E)-7-(3,5-dimethoxyphenoxy)-5,6-dimethyl-4-oxo-2-phenylhepta-2,5-dien-3-yl)-4,5-diphenyloxazolidin-2-one 25

NOESY ($CDCl_3$, 400 MHz)

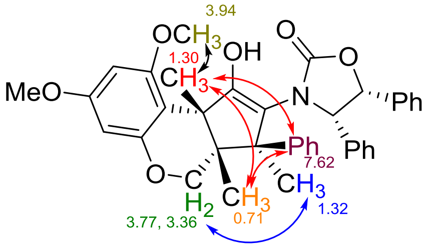

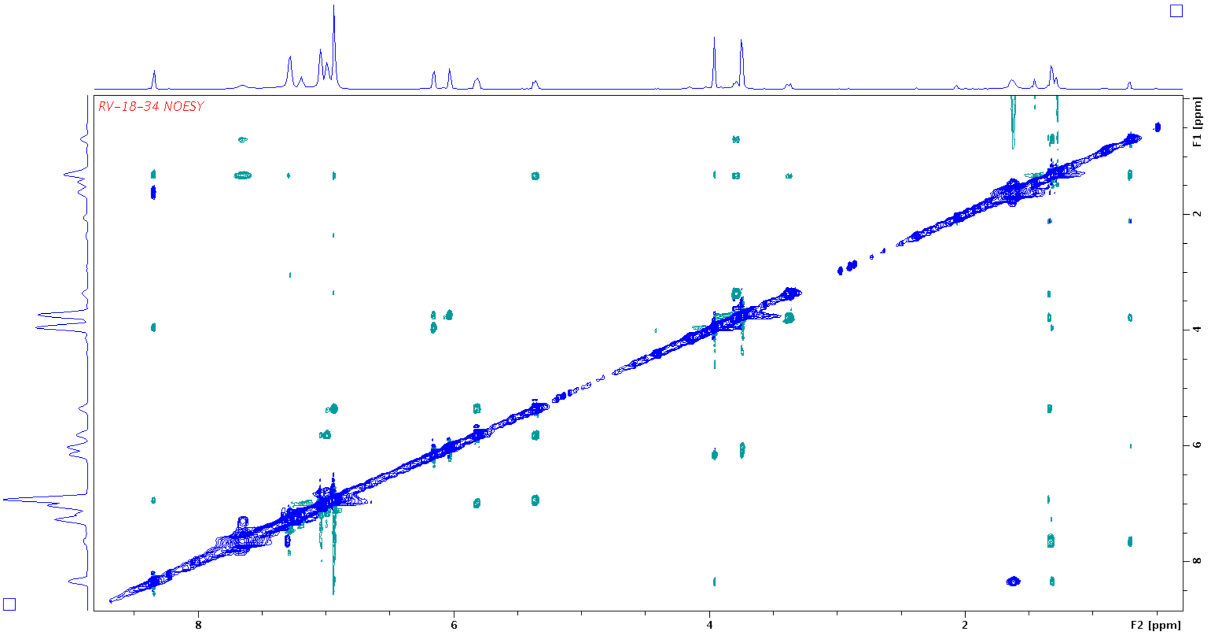

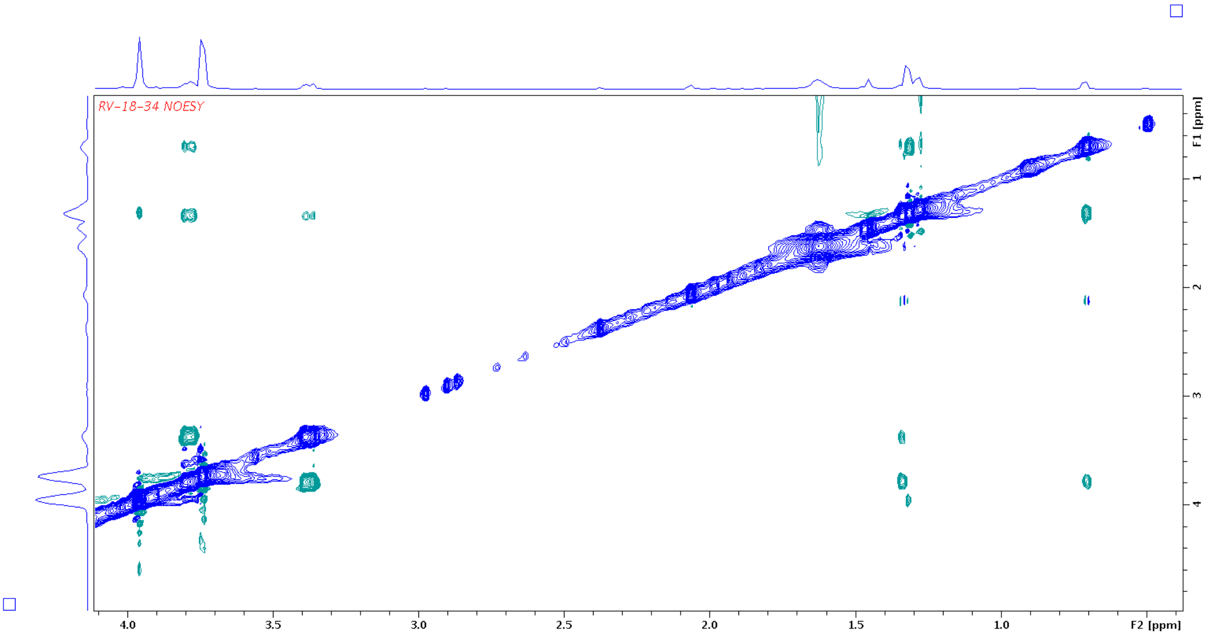

Chiral HPLC traces

(*S*)-1-methyl-1-phenyl-1,2,4,5-tetrahydro-3*H*-cyclopenta[*a*]naphthalen-3-one 12

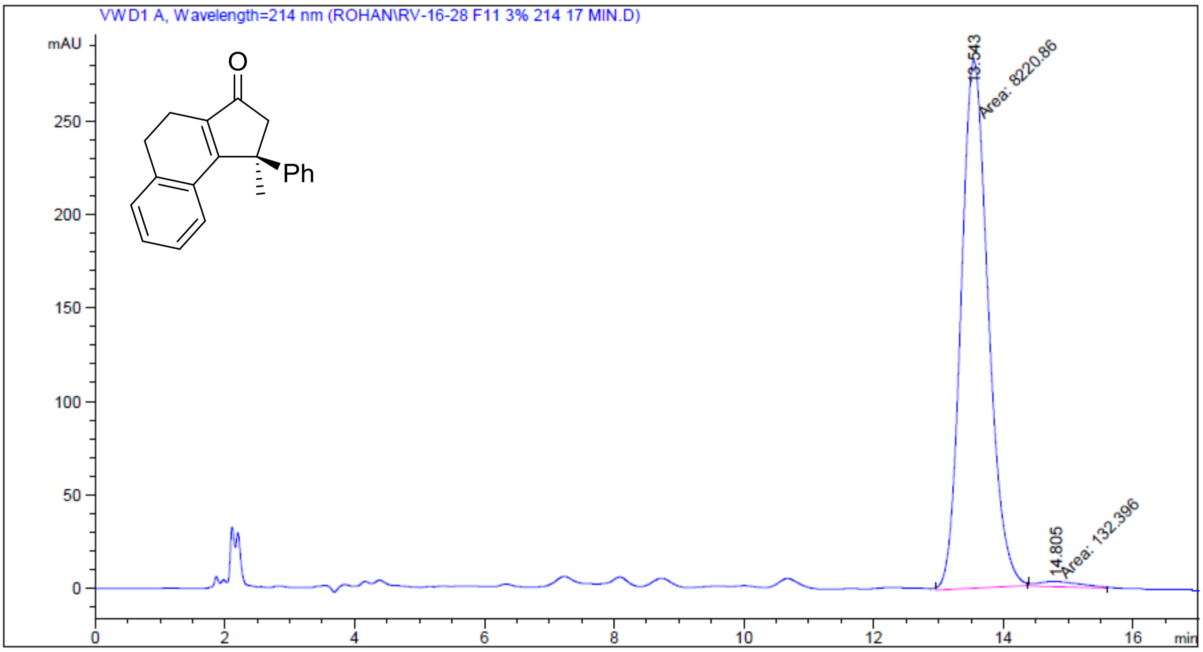

(*S*)-4-methyl-4-phenyl-4,5-dihydro-6*H*-cyclopenta[*b*]thiophen-6-one 13

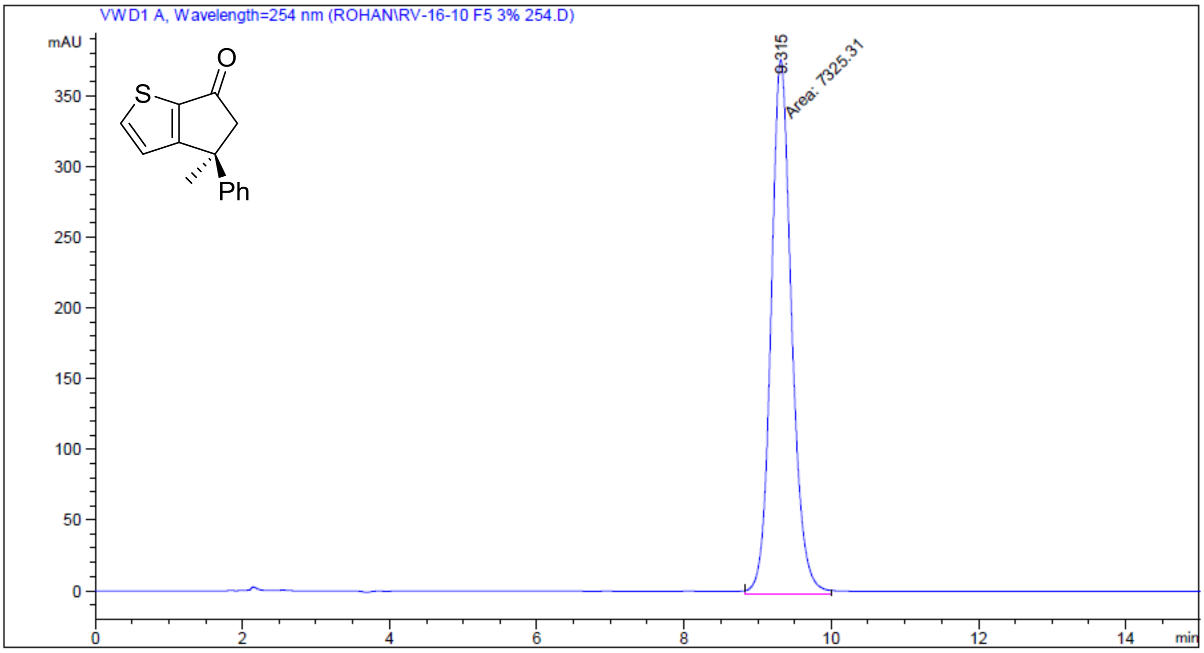

Supplement: Supplementary file 1 [file SC-009-C8SC00031J-s001.pdf]
